# Genetic variation of avian malaria in the tropical Andes: a relationship with the spatial distribution of hosts

**DOI:** 10.1186/s12936-019-2699-9

**Published:** 2019-04-11

**Authors:** Diana Lorena Gil-Vargas, Raul Ernesto Sedano-Cruz

**Affiliations:** 1 Grupo de Investigación en Ecología Animal, Departamento de Biología, Universidad del Valle, Calle 13 No 100-00, Edif. E20, Room 3120, Cali, Colombia; 2 Grupo de Investigación en Ecofisiología, Evolución y Biogeografía, Departamento de Biología, Universidad del Valle, Cali, Colombia

**Keywords:** Malaria, *Plasmodium*, *Haemoproteus*, *Leucocytozoon*, Tropical Andes

## Abstract

**Background:**

Avian haemosporidia are obligate blood parasites with an ample range of hosts worldwide. To understand how host communities may influence the diversity of parasites of the neotropics, the spatial genetic variation of avian *Plasmodium*, *Haemoproteus*, and *Leucocytozoon* was examined between areas of host endemism and along the elevational gradient in the tropical Andes.

**Methods:**

A total of 1686 accessions of the cytochrome *b* gene of avian haemosporidia were selected from 43 publications, that further provides additional information on 14.2% of bird species in the Neotropics. Haplotype groups were identified using a similarity-based clustering of sequences using a cut-off level ≥ 99.3% of sequence identity. Phylogenetic-based analyses were implemented to examine the spatial genetic structure of avian haemosporidia among areas of host endemism and the elevation gradient in the tropical Andes.

**Results:**

The areas of avian endemism, including the tropical Andes, can explain the differential distribution of the haemosporidia cytochrome *b* gene variation. In the tropical Andes region, the total number of avian haemosporidia haplotypes follows a unimodal pattern that peaks at mid-elevation between 2000 and 2500 m above sea level. Furthermore, the haplotype assemblages of obligate blood parasites tend to overlap towards mid-elevation, where avian host diversity tends to be maximized.

**Conclusions:**

Spatial analyses revealed that richness and turnover in haemosporidia suggest an association with montane host diversity, according to elevation in the tropical Andes. In addition, the spatial distribution of haemosporidia diversity is closely associated with patterns of host assemblages over large geographical scale in the tropical Andes and areas of avian endemism nearby.

**Electronic supplementary material:**

The online version of this article (10.1186/s12936-019-2699-9) contains supplementary material, which is available to authorized users.

## Background

Avian haemosporidia, such as the genera *Plasmodium*, *Haemoproteus* and *Leucocytozoon* (phylum Apicomplexa) are obligate blood parasites found in most bird species worldwide [1], transmitted by dipteran vectors [2]. Although genera of avian haemosporidia are not reciprocally monophyletic groups [3, 4], current taxonomy helps to understand that richness of parasite lineages may be highly underestimated [5]. Avian haemosporidians further exhibit high genetic diversity, but the understanding of its distribution across a large geographical scale is incipient.

Spatial distributional patterns of species diversity linking host-parasite relationships are an active topic of ecology and biogeography. The diversity of parasites varies considerably among ecologically distinct habitats [6], and environmental variables seem promising in predicting on large-scale spatial assessment of parasite prevalence [7, 8]. However, little is known about the influence of host assemblages as a change-effect factor of the evolution and spread of parasite diversity. The region of the tropical Andes is constituted by the northern and central Andes, sheltering *ca.* 20% of the global avian fauna [9]. In this region some studies have focused on local estimates of prevalence in multiple hosts species, as in Venezuela [10], Colombia [11, 12] and Ecuador [4]. Very few studies have used a more regional approach to examine the prevalence of avian haemosporidia, but this has been made on specific avian hosts [13, 14].

For studies of avian malaria parasites, the mitochondrial cytochrome *b* gene (cyt *b*) has become a popular molecular marker to rapidly gather phylogenetic information on *Plasmodium*, *Haemoproteus* and *Leucocytozoon* [15–17]. However, these studies are often not the subject of direct comparison because different criteria of cyt *b* divergence are used to identify lineages [18]. The lack of a uniform-criterion to identify lineages hinders any comprehensive, comparative analysis of the haemosporidians genetic variation in a broader geographic and host diversity context. An alternative to this is to establish the limits of haemosporidian lineages based on cyt *b* polymorphism, by defining operative taxonomic units to facilitate data comparison among studies of local variation of parasites [17].

Here a meta-analysis shows spatial patterns of avian malaria genetic diversity on a regional scale for the tropical Andes and areas of endemism nearby. Further, a methodological approach is provided for examining how richness and turnover of haemosporidian parasites may vary according to areas of host endemism and elevation following assemblages of montane avian fauna.

## Methods

### Dataset

To perform a spatial analysis of avian haemosporidia genetic diversity, a dataset was compiled by searching on the Web of Science and Google Scholar terms such as: “avian malaria, Andes, *Leucocytozoon*, *Haemoproteus*, *Plasmodium*” and the names of the countries in South America. Publications on the cyt *b* gene provided information on the taxonomic rank of the parasite, the host bird species and the sampling location, as they represent the effort of researchers to identify de novo haemosporidians for the Neotropics. The information on the cyt *b* gene was verified with those data reported in the GenBank [19] and MalAvi [20]. The sampling sites reported in the different publications were geo-referenced using the QGis program (QGIS Development Team Version 2.1.4. ‘Essen’. 2016) [21].

### Clustering sequences into haplotypes

Here, partial mitochondrial DNA (mtDNA) sequences of avian haemosporidia were used for determining haplotype groups as operative taxonomic units. Clustering of cyt *b* sequences was performed using USEARCH v8.1 [22], and each haplotype group was defined as a *centroid sequence* connecting other mtDNA sequences that make up its haplogroup by a ≥ 99.3% identity threshold. For this clustering procedure, the sequences were sorted into decreasing size in order to be aligned using a ‘semi-global’ method in which each of the group member sequence is aligned with the centroid by the identity threshold. Finally, if the sequences satisfied the identity threshold in the process, they were added as a single haplotypic group. Subsequently, the number of haplotypic groups between the total set of sequences, the number of unique occurrence sequences, and the number of sequences per group were calculated, using the command line: -*sortbylength*, -*cluster_fast*, -*id*, -*centroids*, -*uc*, -*sizeout* (see Additional file 1). To identify haplotype groups, the cyt *b* sequences belonging to *Leucocytozoon* sequences were examined as a separate set of *Plasmodium*–*Haemoproteus*, because these two sets of sequences do conform to independent monophyletic groups and furthermore, the taxa *Plasmodium* and *Haemoproteus* are unequivocally paraphyletic [4]. This methodological approach based on phylogenetics of avian malaria further acknowledges that these two sequence-rich clades harbour substantial variation among *Plasmodium*, *Haemoproteus*, and *Leucocytozoon* in terms of life cycles, their vectors and host specificity.

### Phylogenetics

Phylogenetic inference was performed with each identified haplotypic group to examine the possibility of defining clades using the tree topology. Sequences of 482 bp segment of cyt *b* were aligned using Sequencher v4.7 (Gene Codes Corporation, Ann Arbor, MI, USA). The size of this DNA segment equivalent to 42.3% of the total gene is of common use as amplicon in studies of avian haemosporidia (Additional file 2). Eighteen very short *centroid sequences* were omitted from the alignment, because of the small size between 186 and 356 base pairs. To determine the phylogenetic relationships among the remaining haplotypes, 7 independent Bayesian analyses were performed with BEAST v1.8.3 [23] in the platform CIPRES Science Gateway V. 3. [24]. The Bayesian phylogeny was inferred from using a nucleotide substitution model GTR + G + I [25], a fixed substitution rate of 0.012 sequence divergence per a million years [26], and a posterior probability value of 0.95 as minimum clade support. The consensus tree was generated in PAUP4 [27] using a 25% burn-in; further, the consensus tree was visualized in the ITOL platform [28].

### Spatial analyses of genetic variation

The genetic structure was calculated from genetic distance matrices between avian haemosporidia haplotypes. In order to mitigate the possibility of saturation effect due to multiple substitution hits at a site, the genetic distance matrix was corrected using a GTR substitution model in PAUP4 [27]. Thus, in this study were used a matrix for the *Plasmodium*–*Haemoproteus* centroid sequences, another for the *Leucocytozoon* centroid sequences and furthermore, a whole haemosporidia matrix for the sequences of the two clades that encompass the three genera. With each of these matrices, an analysis of molecular variance (AMOVA) was implemented with 10,000 permutations using the software Arlequin 3.5 [29]. With the AMOVA, the genetic structure of avian haemosporidia was estimated in three categories: (i) by areas of avian endemism in the Neotropics [9]; (ii) by elevation ranges; and, (iii) by localities in the tropical Andes, as listed in Additional file 3. For the first two categorical analyses, the information was grouped corresponding to the clade *Plasmodium* and *Haemoproteus*, while the clade *Leucocytozoon* was analysed separately.

For the spatial analysis of genetic variation according to elevation, the haplotypes belonging to the genera *Plasmodium*, *Haemoproteus*, and *Leucocytozoon* were assigned into 8 elevation zones from 0 to 4671 m above sea level (masl), with intervals of 500 m in altitude. These intervals along elevation constitute a methodological approach to examine the richness of bird species in the tropical Andes [30, 31]. Thus, a bias-corrected rarefaction analysis was implemented to estimate the haplotype richness for each interval of 500 m in altitude, further plotted using RStudio Team (2016) (RStudio, Inc., Boston, MA, USA). The rarefaction analysis was conducted by using a random haplotype sampling with replacement, to allow the variance of the estimates to be useful for comparison of richness among elevation zones [32].

An AMOVA was performed using Arlequin 3.5 [29] to examine patterns of genetic differentiation among areas of avian endemism in the Neotropical region [9]. But also, AMOVA was implemented in order to examine the genetic differentiation among localities within areas of avian endemism in the tropical Andes, for two groups within the tropical Andes, the first with 33 localities belonging to the Northern Andes and the second with 21 localities within the Central Andes. For this third category of spatial analysis, the haplotypes belonging to the three genera were organized in a matrix by localities for which was calculated the distance among them using the Geographic Distance Matrix Generator v1.2.3 [33].

### Correlation analysis of nucleotide diversity with elevation

A Shapiro–Wilk (α = 0.05) was carried to test for the normality of the genetic diversity and the elevation data sets. Because the Shapiro–Wilk test was rejected, the association between genetic diversity and elevation was examined using the Spearman rank correlation. The relationship between genetic variation and elevation was examined using the regression of genetic diversity of *Plasmodium*, *Haemoproteus*, and *Leucocytozoon* on the elevation of 54 localities in the tropical Andes. Genetic diversity is shown here as the probability that two randomly chosen homologous nucleotide sites in the cyt *b* sequence will be different, this is equivalent to nucleotide diversity for DNA data [29]. Additionally, the regression was performed only for the *Plasmodium*–*Haemoproteus* group in order to determine if the relationship between the variables was maintained independently of sample size. Statistical analyses and plots were performed with the statistical package RStudio Team (2016) (RStudio, Inc., Boston, MA, USA).

## Results

### Determination of haplotypes

**Fig. 1:**
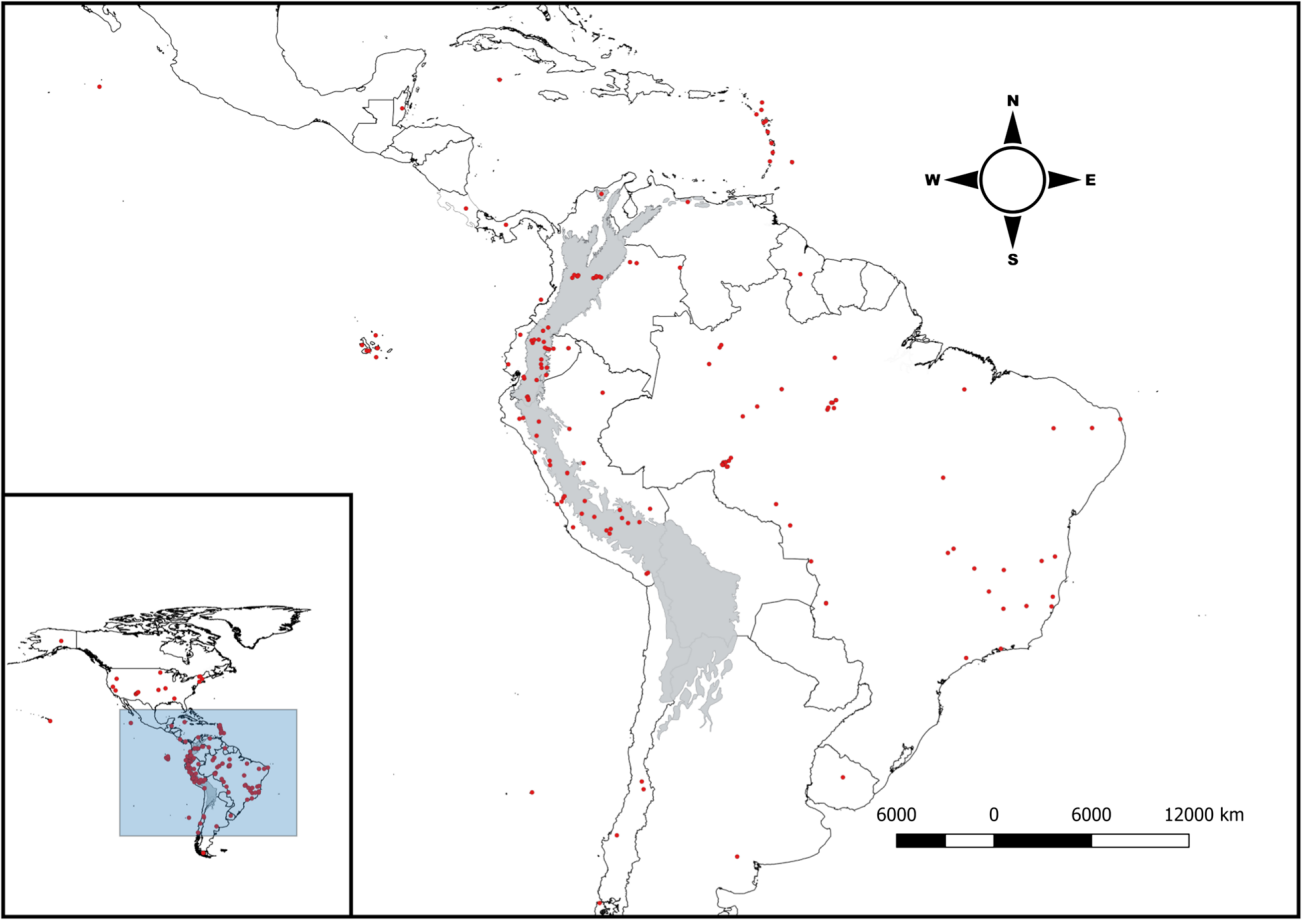
Map of sampling localities of the 43 studies. (Red dots) (see Additional file 3). In the lower left corner of the Neotropical region, the tropical Andes are shown in the grey area

In total, 619 haemosporidia haplotype groups from 1686 cyt *b* accessions of the GenBank and MalAvi were identified from 43 studies published between 2000 and 2017. Of the haemosporidia haplogroups, 13% corresponded to the genera *Leucocytozoon* and the remaining 87% to the haplotypes of the genera *Haemoproteus* and *Plasmodium*, as summarized in Additional file 4. The compiled dataset provides evidence of haemosporidian infection for 604 bird species of 67 avian families (Additional file 3), 540 are Neotropical bird species and a sub-set of 245 species restricted to the tropical Andes. For *Plasmodium* and *Haemoproteus* were found 498 haplotype groups of that occur in 16 of the 22 areas of endemism for the Neotropical avian fauna [9]. For *Leucocytozoon* there are 77 haplotypes restricted among four areas of host endemism. The GenBank accessions and host species were associated with 116 and 54 geo-referenced localities for the Neotropical region and the tropical Andes, respectively (Fig. 1, see geo-referenced data in Additional file 3).

**Fig. 2:**
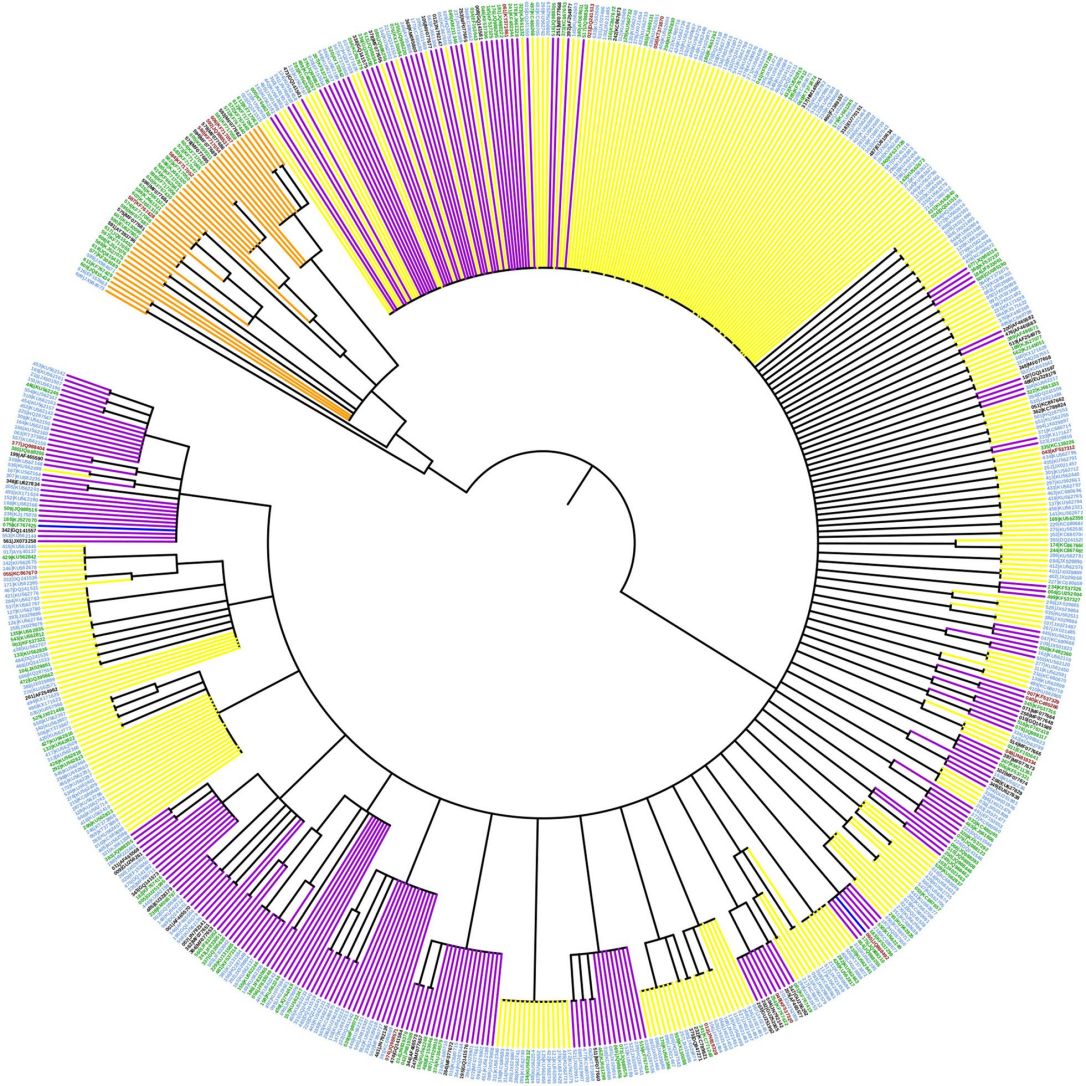
Bayesian inference of 601 haplotype groups of avian haemosporidia. Only posterior probability values ≥ 0.95 are shown as nodal support on the consensus tree. Branches represent the three genera: *Plasmodium* (yellow), *Haemoproteus* (violet), *Leucocytozoon* (orange) and *Plasmodium*–*Haemoproteus* undetermined haplotypes (blue). The colours of the outer ring correspond to the zoogeographic regions of endemism in Neotropical birds [9] for each tip of the tree: tropical Andes region (green), tropical Andes and other zoogeographic regions (brown), other zoogeographic regions, less in the tropical Andes (blue) and outside the neotropics (black) (see Additional file 3)

Avian haemosporidia haplotype richness continuously increases with the number of GenBank accessions of cyt *b*, as shown in the rarefaction plot in the Additional file 5. The single-occurrence haplotypes in the rarefaction plot further implies that the majority of sequences (62%) shared a sequence identity below criterion ≥ 99.3% for determining haplotypes. These single-occurrence haplotypes exhibit variation in size with a median of 482 bp per fragment for the genera *Haemoproteus* and *Plasmodium*, and a median size of 479 bp per fragment for the genera *Leucocytozoon*. For *Leucocytozoon* 77 sequences were found and equivalent to 88% of single-occurrences in the database. From a phylogenetic standpoint, the Bayesian-based inference representing each of the 601 haplotype groups (18 very short sequences were excluded) shown an extensive pattern of polytomies and only 168 nodes with high support of posterior probability (≥ 0.95) in the genealogy (Fig. 2).

### Genetic differentiation of avian haemosporidians

A differential distribution of the genetic variation within the clade *Plasmodium*–*Haemoproteus* was found among 16 areas of avian endemism (AMOVA, Fst_15, 2525__(GTR-corrected)_ = 0.0888, Exact Test of individual distribution P < 0.00001) (see areas of avian endemism in Additional file 3). For *Leucocytozoon*, there is also evidence of genetic differentiation among fewer areas of avian endemism as the Central Andes, Northern Andes, Southern Andes and the Subtropical Pacific (AMOVA, Fst_3,104__(GTR-corrected)_ = 0.229, Exact Test of individual distribution P < 0.00001).

### Altitudinal distribution of avian malaria in the Andes

**Fig. 3:**
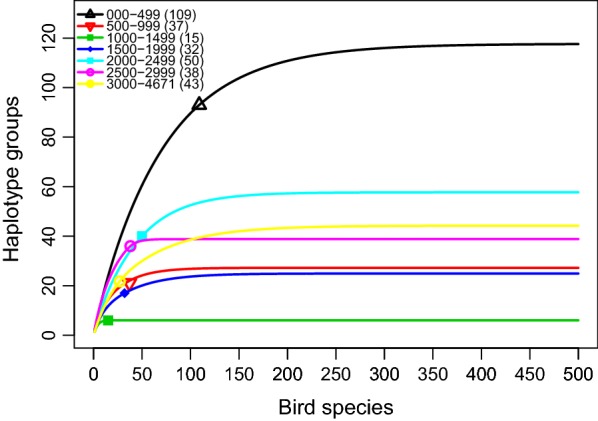
Rarefaction of avian haemosporidia haplogroups diversity by elevation ranges. The number of unique haplotypes estimated by groups of 500 m elevation range in the tropical Andes. Symbols on curves represent the observed number of host species for each of these ranges

**Fig. 4:**
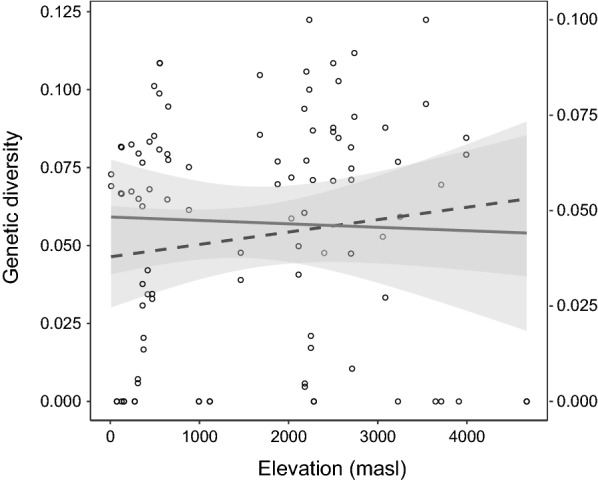
Regression of the genetic diversity of avian haemosporidia on elevation ranges in the tropical Andes. Regression of genetic diversity for *Plasmodium*, *Haemoproteus* and *Leucocytozoon* on the elevation of sampling localities (full dark-grey line). The upper and lower 95% confidence intervals (grey area). Genetic diversity = 3971 e^−06^ *(elevation) + 4638 e^−02^, R^2^ = 0.02035. Regression of genetic diversity for the genera *Plasmodium* and *Haemoproteus* (excluding *Leucocytozoon*) on elevation (dashed-line dark-grey). Genetic diversity = − 8980 e^−07^ *(elevation) + 4833 e^−02^, R^2^ = 0.001245

**Fig. 5:**
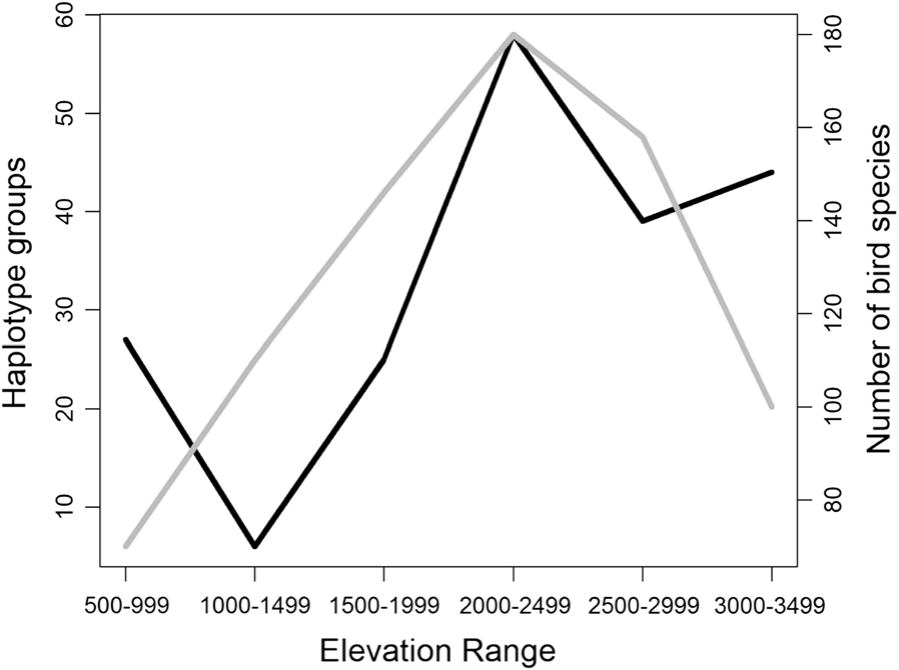
The number of avian haemosporidia haplogroups and host species by elevation range. Estimates of avian haemosporidia haplotype richness according to elevation (dark line) (see rarefaction in Fig. 3), and the number of montane bird species along elevational ranges (grey line) in the tropical Andes [31]

In the tropical Andes, only a fraction of the haplotype groups (0.16%) on the tree is widely distributed in all eight altitudinal zones (Fig. 2), the vast majority of the haplotype groups are distributed in fewer altitudinal zones (Additional file 3). Richness estimates of haplogroups by elevation zone are somewhat independent of sample size of the host species (Fig. 3). For all three haemosporidian genera there is a lack of correlation between nucleotide variation and elevation (Spearman, ρ = 0.2310, P = 0.0927), which further become an even weaker after excluding *Leucocytozoon* from this analysis (Fig. 4). This strongly suggests that the estimate of avian parasite nucleotide diversity is unlikely linearly correlated with elevation. Instead, it was found that richness of avian haemosporidian haplotypes followed a unimodal pattern that peaks at mid-elevation between 2000 and 2500 masl in the tropical Andes (Fig. 5).

### Turnover rates of haemosporidia haplotypes

**Fig. 6:**
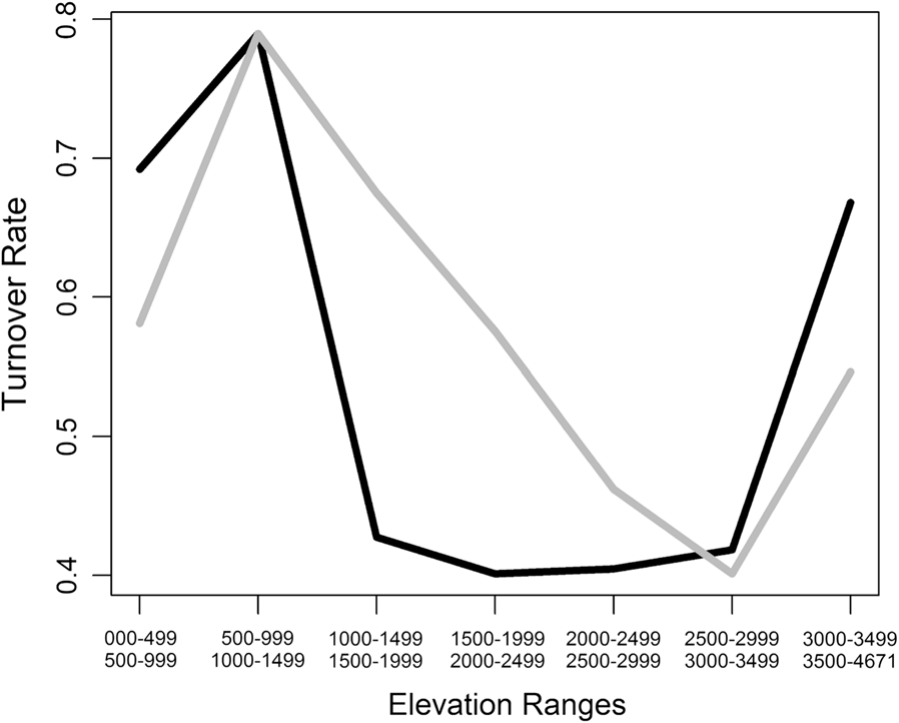
Turnover of avian haemosporidia haplogroups and hosts along elevation. Turnover of haemosporidia haplotypes (dark) between adjacent elevational ranges and turnover of avian host species (grey) in the tropical Andes

Turnover of haemosporidia haplotypes according to elevation shows the gains and losses of haplotypes between adjacent elevation zones; this clearly suggests an increasing overlap of parasite haplogroups around 2000–2500 masl (Fig. 6). Because of this pattern, a linear regression was used here to roughly estimate the average amount of genetic differentiation among elevation zones. For the set *Plasmodium*–*Haemoproteus*, there is evidence of genetic differentiation among altitudinal zones (AMOVA, Fst_7, 618__(GTR-corrected)_ = 0.0678, Exact test of individual distributions P = 0.000001), a pattern consistent with what found for *Leucocytozoon* mainly restricted to 2000–4671 masl, where they also exhibit genetic differentiation between intervals of 500 m along the elevation (AMOVA, Fst_7,97__(GTR-corrected)_ = 0.06504, Exact test of individual distributions P = 0.00444).

## Discussion

Avian haemosporidia accessions in the GenBank and MalAvi databases used here show infection for roughly 14.2% of bird species with distribution in the Neotropics and 26.8% of the haplogroups including the tropical Andes in their geographical range. The cut-off level ≥ 99.3% sequence similarity to determine haplogroups (or ≈ 0.7% of differentiation among sequences) used in this study accounts for variation in haemosporidia that could be considered intraspecific, interspecific or both. Overall, the cyt *b* gene can vary in sequence identity from 0.1 to 9.2% within well-supported haemosporidia species [17, 34]. The resulting set of haplotype groups after using the less stringent clustering method than that commonly applied for cyt *b* sequence divergence further suggest that this is a cautious approach to examine avian haemosporidia mtDNA variation. In contrast, the phylogeny shown here is of very limited use to determine operative taxonomical units, because: (i) as opposed to a *Leucocytozoon* well-supported clade, *Plasmodium* and *Haemoproteus* haplotypes exhibit a paraphyletic pattern; and further, (ii) strict clade support occurs only for 28% of the tree nodes if the phylogeny were fully resolved, a pattern consistent with previous phylogenetic inferences [4, 16].

The meta-analysis shown here contributes to the understanding of how assemblages of host communities may play a determinant role on the spatial distribution of parasites genetic diversity. It is important to emphasize that the nucleotide variation of *Plasmodium* and *Haemoproteus* distorts from a linear correlation with elevation. This suggests further support to the complex relationship between parasite richness and spatial distributions [35, 36]. In contrast, the total number of haemosporidia haplogroups peaks at mid-elevation, such a curve is strikingly consistent to the unimodal pattern for richness of montane avian fauna in the tropical Andes that also peaks towards 2000–2500 masl (see Fig. 5 in [31]). Therefore, the richness of avian haemosporidian forms at the mid-elevation may be influenced by the richness of montane birds susceptible to infection in this elevational zone [37].

In the tropical Andes, it was found that cyt *b* gene variation in avian haemosporidia is accumulated *within* rather than *among* elevation ranges. The distribution of haplogroups among areas of host endemism can explain roughly 9 and 23% of the cyt *b* variation for *Plasmodium*–*Haemoproteus* and *Leucocytozoon*, respectively. Clearly, areas that comprise avian fauna that exhibit phylogenetic and distributional congruence in the Neotropical region [38] are also congruent with the spatial distribution of the genetic variation of obligate blood parasites, as avian haemosporidia. In addition, the amplification of the avian haemosporidia genetic diversity in mid-elevation of the tropical Andes is consistent with prevalence peaks at mid-elevation documented on a local scale for the *Plasmodium* and *Haemoproteus* in the Andes of Colombia, Ecuador and Peru [4, 11, 13, 14]. It stands to reason that if the local array of hosts in a habitat is higher at mid-elevation as in the tropical Andes, the number of parasites would get amplified in order to exploit host opportunity [3, 14, 35]. Therefore, host opportunities for haemosporidian parasites may increase as the endemism of montane bird populations increases towards middle and upper elevations in the tropical Andes [39]. Collectively, reports of local prevalence of avian malaria parasites across the tropical Andes are consistent with how the large geographical scale pattern of haemosporidia genetic variation varies according to regional assemblages of avian hosts.

The phylogeny shows that 20.3% of the haplotype groups are distributed in two or more of the eight intervals of 500 m in altitude in the tropical Andes. These haemosporidian haplotypes forms are spatially generalists to infect avian hosts along elevation. Turnover of haemosporidians also shows a dramatic increasing overlap of haplotype assemblages among adjacent zones towards mid-elevation. This pattern minimally means that more homogeneous genetic assemblages of obligate blood parasites towards mid-elevation, where parasite diversity peaks. Such an assemblage of overlapping haplotypes succeeded in exploiting zones of the richest host habitats where also avian diversity tends to be maximized in the tropical Andes [30, 31]. This finding suggests that parasites that can exploit host diversity are likely a better fit to habitats rich in hosts [6]. Nevertheless, the groups of generalist haplotypes for elevation in the database are a minority of haemosporidia forms in the tropical Andes.

In contrast, 79.5% of the haplotype groups are restricted to one and only one elevational range among the eight intervals of 500 m in altitude in the tropical Andes; thus, more than three-quarters of haemosporidian haplotypes are elevation restricted to infect hosts. For instance, consistent with results here, it is well documented that *Leucocytozoon* is mainly restricted to the highlands, where they are likely prevailing at lower temperatures and where their vectors are common as well [40]. According to the results of this study, most haemosporidians could be restricted to certain elevation ranges to infect montane bird species because avian hosts also have a more or less limited distribution by altitudinal zones in the tropical Andes [41]. This spatial distribution pattern could also be limited by the dynamics of the vectors, as observed on Hawaii island [42]. Unfortunately for the tropical Andes, little is known about the influence of vectors on avian malaria infection yet.

## Conclusions

This study provides evidence of distributional congruence over a large geographical scale between obligate blood parasites in avian assemblages in the tropical Andes and areas of host endemism in the Neotropics. It provides evidence that there is a compelling association between parasite diversity according to the elevational distribution of montane avian diversity in the tropical Andes. Collectively these findings support the hypothesis that the diversity of avian malaria parasites increases due to the concomitant increase in host diversity. If findings are correct, research on the vector ecology is in need to understand disease transmission along elevation, a factor that is likely to be controlled by both hosts and parasites. This work might encourage efforts on the regional analysis of haemosporidia cyt *b* variation in a broader geographic and host diversity context; to widen understanding of how host and vector assemblages determine spatial patterns of parasite communities.

## Additional files

**Additional file 1.** Haplotype group determination. A centroid sequence is determined using medium to high-identity clustering of mtDNA sequences.

**Additional file 2.** References citing accessions of avian haemosporidia cytochrome *b* gene in America.

**Additional file 3.** Information associated to the accessions of avian haemosporidia cytochrome *b* gene. The zoogeographic regions following Parker et al. [9]. Sequences removed from alignment (*).

**Additional file 4.** Summary of clustering accessions of avian haemosporidia cytochrome *b* into haplotype groups. Haplotypes were determined using USEARCH v8.1 [22], implementing a cut-off level criterion > 99.3% similarity between sequences, for 1686 unique accessions in the database and further detailed in Additional file 3. The number of accessions refers to the unique sequences in the database used for the haplotype determination.

**Additional file 5.** Rarefaction plot of avian haemosporidia haplotype richness on the number of GenBank accessions. Rarefaction of the number of haplotype groups of avian haemosporidia as a function of the number of cyt *b* sequences in the dataset using EstimateS v 9.1.0 [32]. The grey area represents the 95% level of confidence interval. A total of 619 haplotypes (black circle) of 1686 cyt *b* sequences from avian malaria parasites was obtained; the dotted line represents the extrapolated rarefaction value up to 6000 sequences.

## Abbreviations

AMOVA: analysis of molecular variance
cyt *b*: cytochrome *b* gene
masl: meters above sea level
mtDNA: mitochondrial DNA
