# Supplementary material for: Genetic variation of avian malaria in the tropical Andes: a relationship with the spatial distribution of hosts
Source: Malar J. 2019 Apr 11;18:129. doi: 10.1186/s12936-019-2699-9 (PMC6458820; doi:10.1186/s12936-019-2699-9)
Supplement: Supplementary file 1 — Additional file 1. Haplotype group determination. A centroid sequence is determined using medium to high-identity clustering of mtDNA sequences. [file 12936_2019_2699_MOESM1_ESM.pptx]

## Slide 1
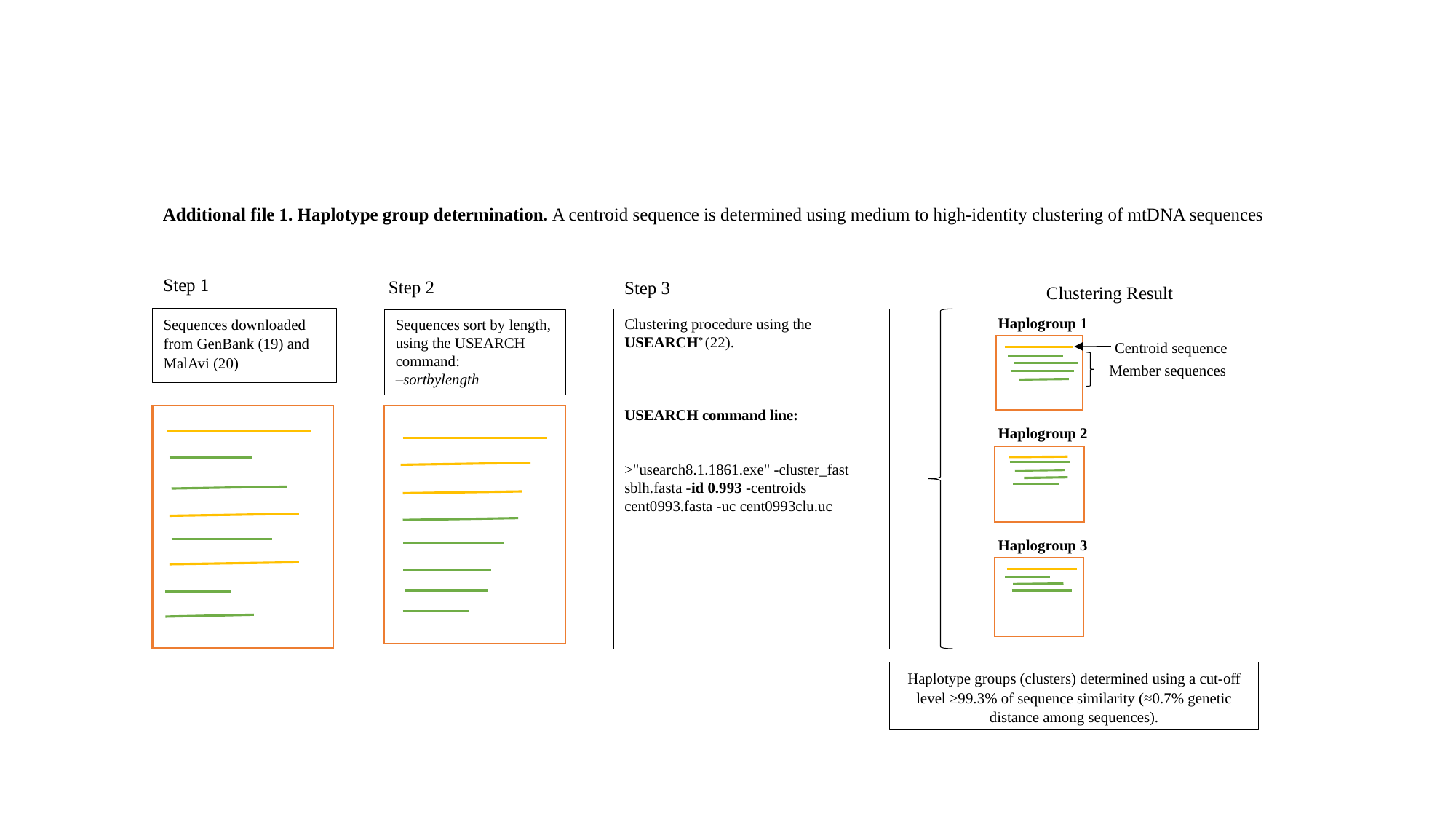

Additional file 1. Haplotype group determination. A centroid sequence is determined using medium to high-identity clustering of mtDNA sequences
Step 1
Step 2
Step 3
Clustering Result
Sequences downloaded from GenBank (19) and MalAvi (20)
Haplogroup 1
Clustering procedure using the USEARCH* (22).
USEARCH command line:
>"usearch8.1.1861.exe" -cluster_fast sblh.fasta -id 0.993 -centroids cent0993.fasta -uc cent0993clu.uc
Sequences sort by length, using the USEARCH command:
–sortbylength
Centroid sequence
Member sequences
Haplogroup 2
Haplogroup 3
Haplotype groups (clusters) determined using a cut-off level ≥99.3% of sequence similarity (≈0.7% genetic distance among sequences).
