# Supplementary material for: Genetic variation of avian malaria in the tropical Andes: a relationship with the spatial distribution of hosts
Source: Malar J. 2019 Apr 11;18:129. doi: 10.1186/s12936-019-2699-9 (PMC6458820; doi:10.1186/s12936-019-2699-9)
Supplement: Supplementary file 2 — Additional file 2. References citing accessions of avian haemosporidia cytochrome b gene in America. [file 12936_2019_2699_MOESM2_ESM.docx]

**Additional file 2.** **References citing accessions of avian haemosporidia cytochrome *b*  gene in America.**

Belo, N.O., Pinheiro, R.T., Reis, E.S., Ricklefs, R.E., Braga, É.M., 2011. Prevalence and lineage diversity of avian haemosporidians from three distinct cerrado habitats in Brazil. PLoS One 6, 1–8. https://doi.org/10.1371/journal.pone.0017654

Bensch, S., Stjernman, M., Hasselquist, D., Ústman, Ú., Hansson, B., Westerdahl, H., Pinheiro, R.T., 2000. Host specificity in avian blood parasites : a study of Plasmodium and Haemoproteus mitochondrial DNA amplified from birds. Proc. R. Soc. 267, 1583–1589. https://doi.org/10.1098/rspb.2000.1181

Bensch, S., Hellgren, O., Pérez-Tris, J., 2009. MalAvi: A public database of malaria parasites and related haemosporidians in avian hosts based on mitochondrial cytochrome b lineages. Mol. Ecol. Resour. 9, 1353–1358. https://doi.org/10.1111/j.1755-0998.2009.02692.x

Benson, D.A., Karsch-Mizrachi, I., Lipman, D.J., Ostell, J., Sayers, E.W., 2011. GenBank. Nucleic Acids Res. 39, D32–D37. https://doi.org/10.1093/nar/gkq1079

Bosholn, M., Fecchio, A., Silveira, P., Braga, É.M., Anciães, M., 2016. Effects of avian malaria on male behaviour and female visitation in lekking blue-crowned manakins. J. Avian Biol. 47, 457–465. https://doi.org/10.1111/jav.00864

Cadena Ortiz, H.F., 2015. Malaria Aviar en los Chingolos (Zonotrichia capensis) del Parque Recreacional-Bosque Protector Jerusalem, Pichincha, Ecuador.

Chagas, C.R.F., Guimarães, L. de O., Monteiro, E.F., Valkiūnas, G., Katayama, M.V., Santos, S.V., Guida, F.J.V., Simões, R.F., Kirchgatter, K., 2016. Hemosporidian parasites of free-living birds in the São Paulo Zoo, Brazil. Parasitol. Res. 115, 1443–1452. https://doi.org/10.1007/s00436-015-4878-0

Chagas, C.R.F., Valkiūnas, G., De Oliveira Guimarães, L., Monteiro, E.F., Guida, F.J., Simões, R.F., Rodrigues, P.T., De Albuquerque Luna, E.J., Kirchgatter, K., 2017. Diversity and distribution of avian malaria and related haemosporidian parasites in captive birds from a Brazilian megalopolis. Malar. J. 16, 1–20. https://doi.org/10.1186/s12936-017-1729-8

Coral, A.A., Valkiunas, G., González, A.D., Matta, N.E., 2015. In vitro development of Haemoproteus columbae (Haemosporida: Haemoproteidae), with perspectives for genomic studies of avian haemosporidian parasites. Exp. Parasitol. 157, 163–169. https://doi.org/10.1016/j.exppara.2015.08.003

Durrant, K.L., Beadell, J.S., Ishtiaq, F., Graves, G.R., Olson, S.L., Peirce, M.A., Milensky, C.M., Schmidt, B.K., Gebhard, C., Fleischer, R.C., Durrant, K.L., Beadell, J.S., Ishtiaq, F., Graves, G.R., Olson, S.L., Gering, E., Peirce, M.A., Milensky, C.M., Schmidt, B.K., Gebhard, C., Fleischer, R.C., 2006. CHAPTER 7 AVIAN HEMATOZOA IN SOUTH AMERICA : A COMPARISON OF TEMPERATE AND TROPICAL ZONES, in: American Ornitologis’ Union. pp. 98–111.

Fallon, S.M., Bermingham, E., Ricklefs, R.E., 2005. Host specialization and geographic localization of avian malaria parasites: a regional analysis in the Lesser Antilles. Am. Nat. 165, 466–480. https://doi.org/10.1086/428430

Fallon, S.M., Bermingham, E., Ricklefs, R.E., 2003. Island and taxon effects in parasitism revisited: avian malaria in the Lesser Antilles. Evolution 57, 606–615. https://doi.org/10.1554/0014-3820(2003)057

Galen, S.C., Witt, C.C., 2014. Diverse avian malaria and other haemosporidian parasites in Andean house wrens: Evidence for regional co-diversification by host-switching. J. Avian Biol. 45, 374–386. https://doi.org/10.1111/jav.00375

Gonzalez-Quevedo, C., Rivera-Gutierrez, H.F., Pabón, A., 2016. Prevalence of haemosporidians in a Neotropical endemic bird area. Avian Conserv. Ecol. 11, ZZ. https://doi.org/10.5751/ACE-00834-110107

González, A.D., Lotta, I.A., García, L.F., Moncada, L.I., Matta, N.E., 2015. Avian haemosporidians from Neotropical highlands: Evidence from morphological and molecular data. Parasitol. Int. 64, 48–59. https://doi.org/10.1016/j.parint.2015.01.007

Harrigan, R.J., Sedano, R., Chasar, A.C., Chaves, J. a., Nguyen, J.T., Whitaker, A., Smith, T.B., 2014. New host and lineage diversity of avian haemosporidia in the northern Andes. Evol. Appl. 1–13. https://doi.org/10.1111/eva.12176

Jones, M.R., Cheviron, Z. a, Carling, M.D., 2013. Spatial patterns of avian malaria prevalence in Zonotrichia capensis on the western slope of the Peruvian Andes. J. Parasitol. 99, 903–5. https://doi.org/10.1645/12-147.1

Lacorte, G. a., Flix, G.M.F., Pinheiro, R.R.B., Chaves, A. V., Almeida-Neto, G., Neves, F.S., Leite, L.O., Santos, F.R., Braga, É.M., 2013. Exploring the Diversity and Distribution of Neotropical Avian Malaria Parasites - A Molecular Survey from Southeast Brazil. PLoS One 8, 1–9. https://doi.org/10.1371/journal.pone.0057770

Lee-Cruz, L., Cunningham, A.A., Martínez, P., Cruz, M., Goodman, S.J., Hamer, K.C., 2016. Prevalence of Haemoproteus sp. in Galápagos blue-footed boobies: effects on health and reproduction. Parasitol. Open 2, e1. https://doi.org/10.1017/pao.2015.6

Levin, I.I., Valkiu-nas, G., Santiago-Alarcon, D., Cruz, L.L., Iezhova, T. a., O’Brien, S.L., Hailer, F., Dearborn, D., Schreiber, E. a., Fleischer, R.C., Ricklefs, R.E., Parker, P.G., 2011. Hippoboscid-transmitted Haemoproteus parasites (Haemosporida) infect Galapagos Pelecaniform birds: Evidence from molecular and morphological studies, with a description of Haemoproteus iwa. Int. J. Parasitol. 41, 1019–1027. https://doi.org/10.1016/j.ijpara.2011.03.014

Levin, I.I., Zwiers, P., Deem, S.L., Geest, E. a., Higashiguchi, J.M., Iezhova, T. a., Jiménez-Uzcátegui, G., Kim, D.H., Morton, J.P., Perlut, N.G., Renfrew, R.B., Sari, E.H.R., Valkiunas, G., Parker, P.G., 2013. Multiple Lineages of Avian Malaria Parasites (Plasmodium) in the Galapagos Islands and Evidence for Arrival via Migratory Birds. Conserv. Biol. 27, 1366–1377. https://doi.org/10.1111/cobi.12127

Lotta, I.A., Gonzalez, A.D., Pacheco, M.A., Escalante, A.A., Valkiūnas, G., Moncada, L.I., Matta, N.E., 2015. Leucocytozoon pterotenuis sp. nov. (Haemosporida, Leucocytozoidae): description of the morphologically unique species from the Grallariidae birds, with remarks on the distribution of Leucocytozoon parasites in the Neotropics. Parasitol. Res. 114, 1031–1044. https://doi.org/10.1007/s00436-014-4269-y

Lotta, I.A., Pacheco, M.A., Escalante, A.A., González, A.D., Mantilla, J.S., Moncada, L.I., Adler, P.H., Matta, N.E., 2016. Leucocytozoon Diversity and Possible Vectors in the Neotropical highlands of Colombia. Protist 167, 185–204. https://doi.org/10.1016/j.protis.2016.02.002

Mantilla, J.S., González, A.D., Lotta, I.A., Moens, M., Pacheco, M.A., Escalante, A.A., Valkiunas, G., Moncada, L.I., Pérez-Tris, J., Matta, N.E., 2016. Haemoproteus erythrogravidus n. sp. (Haemosporida, Haemoproteidae): Description and molecular characterization of a widespread blood parasite of birds in South America. Acta Trop. 159, 83–94. https://doi.org/10.1016/j.actatropica.2016.02.025

Mantilla, J.S., González, A.D., Valki, G., 2013. Description and molecular characterization of Plasmodium ( Novyella ) unalis sp . nov . from the Great Thrush ( Turdus fuscater ) in highland of Colombia. Parasitol. Res. Volume 112, 4193–4204. https://doi.org/10.1007/s00436-013-3611-0

Marroquin-Flores, R.A., Williamson, J.L., Chavez, A.N., Bauernfeind, S.M., Baumann, M.J., Gadek, C.R., Johnson, A.B., McCullough, J.M., Witt, C.C., Barrow, L.N., 2017. Diversity, abundance, and host relationships of avian malaria and related haemosporidians in New Mexico pine forests. PeerJ 5, e3700. https://doi.org/10.7717/peerj.3700

Martínez, J., Vásquez, R.A., Venegas, C., Merino, S., 2015. Molecular characterisation of haemoparasites in forest birds from Robinson Crusoe Island: Is the Austral Thrush a potential threat to endemic birds? Bird Conserv. Int. 25, 139–152. https://doi.org/10.1017/S0959270914000227

Marzal, A., García-Longoria, L., Cárdenas Callirgos, J.M., Sehgal, R.N., 2015. Invasive avian malaria as an emerging parasitic disease in native birds of Peru. Biol. Invasions 17, 39–45. https://doi.org/10.1007/s10530-014-0718-x

Matta, N.E., Pacheco, M.A., Escalante, A.A., Valkiunas, G., Ayerbe-Quiñones, F., Acevedo-Cendales, L.D., 2014. Description and molecular characterization of Haemoproteus macrovacuolatus n. sp. (Haemosporida, Haemoproteidae), a morphologically unique blood parasite of black-bellied whistling duck (Dendrocygna autumnalis) from South America. Parasitol. Res. 113, 2991–3000. https://doi.org/10.1007/s00436-014-3961-2

Merino, S., Moreno, J., Vásquez, R. a., Martínez, J., Sánchez-Monsálvez, I., Estades, C.F., Ippi, S., Sabat, P., Rozzi, R., Mcgehee, S., 2008. Haematozoa in forest birds from southern Chile: Latitudinal gradients in prevalence and parasite lineage richness. Austral Ecol. 33, 329–340. https://doi.org/10.1111/j.1442-9993.2008.01820.x

Mijares, A., Rosales, R., Silva-Iturriza, A., 2012. Hemosporidian Parasites in Forest Birds from Venezuela: Genetic Lineage Analyses. Avian Dis. Dig. 7, e56–e57. https://doi.org/10.1637/10223-1005812-DIGEST.1

Moens, M., 2017. Diversity and host specificity of symbionts in neotropical birds. Universidad Complutense de Madrid.

Moens, M.A.J., Pérez-Tris, J., 2016a. Discovering potential sources of emerging pathogens: South America is a reservoir of generalist avian blood parasites. Int. J. Parasitol. https://doi.org/10.1016/j.ijpara.2015.08.001

Moens, M.A.J., Pérez-Tris, J., 2016b. Discovering potential sources of emerging pathogens: South America is a reservoir of generalist avian blood parasites. Int. J. Parasitol. 46, 41–49. https://doi.org/10.1016/j.ijpara.2015.08.001

Ricklefs, R.E., Medeiros, M., Ellis, V.A., Svensson-Coelho, M., Blake, J.G., Loiselle, B.A., Soares, L., Fecchio, A., Outlaw, D., Marra, P.P., Latta, S.C., Valkiūnas, G., Hellgren, O., Bensch, S., 2017. Avian migration and the distribution of malaria parasites in New World passerine birds. J. Biogeogr. 44, 1113–1123. https://doi.org/10.1111/jbi.12928

Ricopa, L., Villa, Z., 2016. Prevalencia y diversidad de hemoparásitos en aves capturadas en la Reserva Nacional Allpahuayo Mishana (RNAM), Iquitos-Perú, 2013. UNIVERSIDAD NACIONAL DE LA AMAZONIA PERUANA.

Roos, F.L., Belo, N.O., Silveira, P., Braga, E.M., 2015. Prevalence and diversity of avian malaria parasites in migratory Black Skimmers (Rynchops niger, Laridae, Charadriiformes) from the Brazilian Amazon Basin. Parasitol. Res. 114, 3903–3911. https://doi.org/10.1007/s00436-015-4622-9

Sallaberry-Pincheira, N., Gonzalez-Acuña, D., Herrera-Tello, Y., Dantas, G.P.M., Luna-Jorquera, G., Frere, E., Valdés-Velasquez, A., Simeone, A., Vianna, J.A., 2015. Molecular Epidemiology of Avian Malaria in Wild Breeding Colonies of Humboldt and Magellanic Penguins in South America. Ecohealth 12, 267–277. https://doi.org/10.1007/s10393-014-0995-y

Smith, M.M., Ramey, A.M., 2015. Prevalence and genetic diversity of haematozoa in South American waterfowl and evidence for intercontinental redistribution of parasites by migratory birds. Int. J. Parasitol. Parasites Wildl. 4, 22–28. https://doi.org/10.1016/j.ijppaw.2014.12.007

Svensson-Coelho, M., Loiselle, B.A., Blake, J.G., Ricklefs, R.E., 2016. Resource predictability and specialization in avian malaria parasites. Mol. Ecol. 25, 4377–4391. https://doi.org/10.1111/mec.13758

Szymanski, M.M., Lovette, I.J., 2005. High lineage diversity and host sharing of malarial parasites in a local avian assemblage. J. Parasitol. 91, 768–774. https://doi.org/10.1645/GE-417R1.1

Walther, E.L., Valkiunas, G., González, A.D., Matta, N.E., Ricklefs, R.E., Cornel, A., Sehgal, R.N.M., 2014. Description, molecular characterization, and patterns of distribution of a widespread New World avian malaria parasite (Haemosporida: Plasmodiidae), Plasmodium (Novyella) homopolare sp. nov. Parasitol. Res. https://doi.org/10.1007/s00436-014-3995-5

Yohannes, E., Križanauskienè, A., Valcu, M., Bensch, S., Kempenaers, B., 2009. Prevalence of malaria and related haemosporidian parasites in two shorebird species with different winter habitat distribution. J. Ornithol. 150, 287–291. https://doi.org/10.1007/s10336-008-0349-z
