# Supplementary material for: Genetic variation of avian malaria in the tropical Andes: a relationship with the spatial distribution of hosts
Source: Malar J. 2019 Apr 11;18:129. doi: 10.1186/s12936-019-2699-9 (PMC6458820; doi:10.1186/s12936-019-2699-9)
Supplement: Supplementary file 3 — Additional file 3. Information associated to the accessions of avian haemosporidia cytochrome b gene. The zoogeographic regions following Parker et al. [9]. Sequences removed from alignment (*). [file 12936_2019_2699_MOESM3_ESM.docx]

**Additional file 3. Information associated to the accessions of avian haemosporidia cytochrome *b* gene.** The zoogeographic regions following Parker et al. (1996). Sequences removed from alignment (*).

| **GenBank** | **Latitude** | **Longitude** | **Genus or species of parasite** | **Avian Species** | **Continent or country** | **Locality** | **MalAvi Lineage** | **Haplotypic group** | **Geographic Region** | **Reference** |
| --- | --- | --- | --- | --- | --- | --- | --- | --- | --- | --- |
| KJ661265 | -0,599 | -77,89 | *Haemoproteus* sp. | *Diglossa cyanea* | Ecuador | Cerro Bosco | NA | 0 | NAN | (Harrigan *et al*., 2014) |
| KJ661267 | -0,599 | -77,89 | *Haemoproteus* sp. | *Diglossa cyanea* | Ecuador | Cerro Bosco | NA | 0 | NAN | (Harrigan *et al*., 2014) |
| KJ661268 | -0,599 | -77,89 | *Haemoproteus* sp. | *Diglossa cyanea* | Ecuador | Cerro Bosco | NA | 0 | NAN | (Harrigan *et al*., 2014) |
| KX130085 | 10,829 | -73,692 | *Haemoproteus* sp. | *Atlapetes melanocephalus* | Colombia | Sierra Nevada de Santa Marta, San Lorenzo ridge | NA | 0 | NAN | (Gonzalez-Quevedo, Rivera-Gutierrez & Pabón, 2016) |
| KT698210 | 4,711 | -74,072 | *Haemoproteus coatneyi* | *Arremon brunneinucha* | Colombia | NA | NA | 0 | NA | (Mantilla *et al*., 2016) |
| AF465570 | NA | NA | *Haemoproteus* sp. | *Tachycineta thalassina* | North America Continental | NA | TATHA01 | 1 | NA | (Galen and Witt, 2014) |
| AY640129 | 37,09 | -95,712 | *Haemoproteus* sp. | *Setophaga petechia* | United States of America | NA | NA | 2 | NA | (Szymanski and Lovette, 2005) |
| AY640129 | -3,944 | -73,607 | *Haemoproteus* sp. | *Lepidothrix coronata* | Peru | Allpahuayo Mishana National Reserve | NA | 2 | AMN | (Ricopa & Villa, 2016) |
| AY640129 | -3,944 | -73,607 | *Haemoproteus* sp. | *Cacicus cela* | Peru | Allpahuayo Mishana National Reserve | NA | 2 | AMN | (Ricopa & Villa, 2016) |
| KC680664 | -0,633 | -76,133 | *Haemoproteus* sp. | *Hafferia fortis* | Ecuador | Tiputini Biodiversity Station, Orellana Province | H1 | 2 | AMN | (Svensson-Coelho *et al*. 2016) |
| KC867678 | 43,265 | -118,845 | *Haemoproteus* sp. | *Dolichonyx oryzivorus* | United States of America | Oregon - Malheur National Wildlife Refuge | SIAMEX01 | 2 | NA | (Levin *et al*., 2013) |
| MF077667 | 35,532 | -107,355 | *Haemoproteus* sp. | *Setophaga nigrescens* | United States of America | Nuevo Mexico, Mesa Chivato | TABI02 | 2 | NA | (Marroquin-Flores *et al*., 2017) |
| MF077669 | 35,534 | -107,348 | *Haemoproteus* sp. | *Pipilo maculatus* | United States of America | Nuevo Mexico, Mesa Chivato | PIPMAC01 | 2 | NA | (Marroquin-Flores *et al*., 2017) |
| MF077678 | 35,524 | -107,350 | *Haemoproteus* sp. | *Spizella passerina* | United States of America | Nuevo Mexico, Mesa Chivato | SIAMEX01 | 2 | NA | (Marroquin-Flores *et al*., 2017) |
| GQ395668 | NA | NA | *Haemoproteus* sp. | *Spheniscus mendiculus* | North America Continental | NA | TABI02 | 2 | NA | (Galen and Witt, 2014) |
| DQ241532 | 4,86 | -58,93 | *Plasmodium* sp. | *Saltator coerulescens* | Guyana | NA | 25 | 3 | AMN | (Durrant *et al*., 2006) |
| DQ241532 | 4,86 | -58,93 | *Plasmodium* sp. | *Sicalis luteola* | Guyana | NA | 25 | 3 | AMN | (Durrant *et al*., 2006) |
| DQ241532 | 4,86 | -58,93 | *Plasmodium* sp. | *Sturnella militaris* | Guyana | NA | 25 | 3 | AMN | (Durrant *et al*., 2006) |
| KU562769 | -12,217 | -60,73 | *Plasmodium* sp. | *Rhytipterna simplex* | Brazil | Chupinguaia | NA | 3 | AMS | (Fecchio *et al*., 2017) |
| KU562770 | -12,217 | -60,73 | *Plasmodium* sp. | *Chlorophanes spiza* | Brazil | Chupinguaia | NA | 3 | AMS | (Fecchio *et al*., 2017) |
| KF537322 | 4,804 | -75,713 | *Plasmodium* sp. | *Vireo olivaceus* | Colombia | Pereira | VIOLI07 | 3 | NAN | (González *et al*., 2015) |
| DQ241545 | -32,522 | -55,765 | *Haemoproteus* sp. | *Pseudoleistes virescens* | Uruguay | NA | 38 | 4 | PAM | (Durrant *et al*., 2006) |
| DQ241545 | -32,522 | -55,765 | *Haemoproteus* sp. | *Colaptes campestris* | Uruguay | NA | 38 | 4 | PAM | (Durrant *et al*., 2006) |
| DQ241547 | -32,522 | -55,765 | *Haemoproteus* sp. | *Turdus amaurochalinus* | Uruguay | NA | 40 | 4 | PAM | (Durrant *et al*., 2006) |
| DQ241548 | 4,86 | -58,93 | *Haemoproteus* sp. | *Caryothraustes canadensis* | Guyana | NA | 41 | 4 | AMN | (Durrant *et al*., 2006) |
| DQ241548 | 4,86 | -58,93 | *Haemoproteus* sp. | *Saltator coerulescens* | Guyana | NA | 41 | 4 | AMN | (Durrant *et al*., 2006) |
| DQ241549 | 4,86 | -58,93 | *Haemoproteus* sp. | *Psarocolius decumanus* | Guyana | NA | 42 | 4 | AMN | (Durrant *et al*., 2006) |
| GQ141565 | NA | NA | *Parahaemoproteus* sp. | *Coereba flaveola* | Antilles | NA | PHAPAL02 | 4 | NA | (Galen and Witt, 2014) |
| HQ287537 | -10,249 | -48,324 | *Haemoproteus* sp. | *Tangara cayana* | Brazil | Palmas,Tocantins | Toc2 | 4 | CSA | (Belo *et al*., 2011) |
| HQ287537 | -15,842 | -47,972 | *Haemoproteus* sp. | *Tangara cayana* | Brazil | Vila Boa | NA | 4 | CSA | (Ricklefs *et al*., 2017) |
| JX029911 | -18,713 | -44,925 | *Haemoproteus* sp. | *Hemitriccus margaritaceiventer* | Brazil | Felixlândia | PACPEC02 | 4 | CSA | (Lacorte *et al*., 2013) |
| JX029911 | -18,713 | -44,925 | *Haemoproteus* sp. | *Nemosia pileata* | Brazil | Felixlândia | PACPEC02 | 4 | CSA | (Lacorte *et al*., 2013) |
| KF537317 | 4,804 | -75,713 | *Haemoproteus coatneyi* | *Piranga rubra* | Colombia | Pereira | LEPER01 | 4 | NAN | (González *et al*., 2015) |
| KJ661305 | -1,838 | -80,611 | *Haemoproteus* sp. | *Mionectes olivaceus* | Ecuador | Loma Alta | NA | 4 | NAN | (Harrigan *et al*., 2014) |
| KJ661311 | 0,362 | -79,716 | *Haemoproteus* sp. | *Euphonia xanthogaster* | Ecuador | Bilsa | NA | 4 | EPC | (Harrigan *et al*., 2014) |
| KJ661316 | -0,637 | -76,149 | *Haemoproteus* sp. | *Euphonia xanthogaster* | Ecuador | Tiputini | NA | 4 | AMN | (Harrigan *et al*., 2014) |
| KJ661317 | -0,637 | -76,149 | *Haemoproteus* sp. | *Euphonia xanthogaster* | Ecuador | Tiputini | NA | 4 | AMN | (Harrigan *et al*., 2014) |
| KT373859 | -2,117 | -77,733 | *Haemoproteus* sp. | *Euphonia xanthogaster* | Ecuador | Morona-Santiago Province, Wisui | PACPEC02 | 4 | AMN | (Moens & Pérez-Tris, 2016) |
| KT373859 | -2,117 | -77,733 | *Haemoproteus* sp. | *Saltator maximus* | Ecuador | Morona-Santiago Province, Wisui | PACPEC02 | 4 | AMN | (Moens & Pérez-Tris, 2016) |
| KT373859 | -2,087 | -77,751 | *Parahaemoproteus* sp. | *Saltator maximus* | Ecuador | Wisui reserve | NA | 4 | AMN | (Moens *et al*., 2017) |
| MF077652 | 35,533 | -107,349 | *Haemoproteus* sp. | *Piranga ludoviciana* | United States of America | Nuevo Mexico, Mesa Chivato | PIRLUD02 | 4 | NA | (Marroquin-Flores *et al*., 2017) |
| MF077671 | 34,836 | -108,215 | *Haemoproteus* sp. | *Piranga flava* | United States of America | Nuevo Mexico, El Malpais | PIRFLA01 | 4 | NA | (Marroquin-Flores *et al*., 2017) |
| MF077676 | 34,958 | -107,967 | *Haemoproteus* sp. | *Piranga flava* | United States of America | Nuevo Mexico, El Malpais | PACPEC02 | 4 | NA | (Marroquin-Flores *et al*., 2017) |
| MF990721 | 2,967 | -78,184 | *Haemoproteus* sp. | *Coereba flaveola* | Colombia | Cauca, PNN Gorgona, El Poblado | NA | 4 | CHO | In this studio |
| MF990730 | 2,967 | -78,184 | *Haemoproteus* sp. | *Coereba flaveola* | Colombia | Cauca, PNN Gorgona, El Poblado | NA | 4 | CHO | In this studio |
| GU252004 | NA | NA | *Parahaemoproteus* sp. | *Piranga rubra* | North America Continental | NA | NA | 4 | NA | (Galen and Witt, 2014) |
| AY167243 | 14,609 | -61,072 | *Haemoproteus* sp. | *Coereba flaveola* | Antilles | Lesser Antilles - Martinique Island | HD | 5 | LAN | (Fallon *et al*., 2005; Fallon *et al*., 2003) |
| AY167243 | 17,626 | -61,77 | *Haemoproteus* sp. | *Coereba flaveola* | Antilles | Antillas menores - Barbuda Island | HD | 5 | LAN | (Fallon *et al*., 2005; Fallon *et al*., 2003) |
| AY167243 | 13,909 | -60,978 | *Haemoproteus* sp. | *Coereba flaveola* | Antilles | Lesser Antilles - Saint Lucia Island | HD | 5 | LAN | (Fallon *et al*., 2005; Fallon *et al*., 2003) |
| AY167243 | 13,909 | -60,978 | *Haemoproteus* sp. | *Loxigilla noctis* | Antilles | Lesser Antilles - Saint Lucia Island | HD | 5 | LAN | (Fallon *et al*., 2005; Fallon *et al*., 2003) |
| AY167243 | 16,742 | -62,187 | *Haemoproteus* sp. | *Coereba flaveola* | Antilles | Lesser Antilles - Montserrat Island | HD | 5 | LAN | (Fallon *et al*., 2005; Fallon *et al*., 2003) |
| AY167243 | 16,742 | -62,187 | *Haemoproteus* sp. | *Tiaris bicolor* | Antilles | Lesser Antilles - Montserrat Island | HD | 5 | LAN | (Fallon *et al*., 2005; Fallon *et al*., 2003) |
| GQ141566 | 37,09 | -95,712 | *Parahaemoproteus* sp. | *Coereba flaveola* | United States of America | NA | NA | 5 | NA | (Ricklefs *et al*., 2017) |
| GQ141568 | NA | NA | *Parahaemoproteus* sp. | *Coereba flaveola* | Antilles | NA | COFLA06 | 5 | NA | (Galen and Witt, 2014) |
| GQ395638 | -0,829 | -90,982 | *Haemoproteus* sp. | *Spheniscus mendiculus* | Ecuador | Galápagos Islands | COFLA05 | 5 | NAN | (Galen and Witt, 2014) |
| GQ395652 | -0,829 | -90,982 | *Haemoproteus* sp. | *Spheniscus mendiculus* | Ecuador | Galápagos Islands | COFLA07 | 5 | NAN | (Galen and Witt, 2014) |
| GU251995 | NA | NA | *Parahaemoproteus* sp. | *Coereba flaveola* | Antilles | NA | NA | 5 | NA | (Galen and Witt, 2014) |
| KF537319 | 4,804 | -75,713 | *Parahaemoproteus vireonis* | *Vireo olivaceus* | Colombia | Pereira | ZOCAP09 | 6 | NAN | (González *et al*., 2015) |
| KF537321 | 4,804 | -75,713 | *Parahaemoproteus vireonis* | *Vireo olivaceus* | Colombia | Pereira | VIOLI08 | 6 | NAN | (González *et al*., 2015) |
| AY455658 | 16,235 | -61,488 | *Haemoproteus* sp. | *Loxigilla portoricensis* | Antilles | NA | HH | 7 | LAN | (Fallon *et al*., 2005) |
| EF153649 | -9,916 | -76,233 | *Haemoproteus* sp. | *Zonotrichia capensis* | Peru | Huánuco | ChH4 | 7 | CAN | (Marzal *et al*., 2015) |
| EF153649 | -0,0013 | -78,355 | *Haemoproteus* sp. | *Zonotrichia capensis* | Ecuador | Quito | ChH4 | 7 | NAN | (Cadena *et al*., 2015) |
| EF153649 | -53,163 | -70,917 | *Haemoproteus* sp. | *Zonotrichia capensis* | Chile | Punta Arenas | ChH4 | 7 | SAN | (Merino *et al*., 2008) |
| JQ764618 | 10,231 | -67,285 | *Haemoproteus* sp. | *Chlorospingus ophthalmicus* | Venezuela | Aragua | CHLOP01 | 7 | NAN | (Mijares *et al*., 2012) |
| JQ764618 | -9,916 | -76,233 | *Haemoproteus* sp. | *Zonotrichia capensis* | Peru | Huánuco | CHLOP01 | 7 | CAN | (Marzal *et al*., 2015) |
| JQ988106 | -13,249 | -72,169 | *Parahaemoproteus* sp. | *Chlorospingus flavigularis* | Peru | Cusco | NA | 7 | CAN | (Galen and Witt, 2014) |
| KF537309 | 4,804 | -75,713 | *Haemoproteus coatneyi* | *Atlapetes pallidinucha* | Colombia | Pereira | ATPLA02 | 7 | NAN | (González *et al*., 2015) |
| KF537318 | 4,804 | -75,713 | *Haemoproteus* sp. | *Zonotrichia capensis* | Colombia | Pereira | PACPEC02 | 7 | NAN | (González *et al*., 2015) |
| KF537329 | 4,804 | -75,713 | *Haemoproteus* sp. | *Zonotrichia capensis* | Colombia | Pereira | ZOCAP10 | 7 | NAN | (González *et al*., 2015) |
| KF537330 | 4,804 | -75,713 | *Haemoproteus* sp. | *Zonotrichia capensis* | Colombia | Pereira | ZOCAP10 | 7 | NAN | (González *et al*., 2015) |
| KF767417 | -5,833 | -79,505 | *Haemoproteus* sp. | *Troglodytes aedon* | Peru | Piura | TROAED15 | 7 | CAN | (Galen and Witt, 2014) |
| KJ661246 | -0,689 | -77,727 | *Haemoproteus* sp. | *Phaethornis* sp. | Ecuador | Hollín | NA | 7 | NAN | (Harrigan *et al*., 2014) |
| KJ661247 | -0,725 | -77,575 | *Haemoproteus* sp. | *Phaethornis* sp. | Ecuador | Guagua Sumaco | NA | 7 | NAN | (Harrigan *et al*., 2014) |
| KJ661251 | -0,637 | -76,149 | *Haemoproteus* sp. | *Phaethornis superciliosus* | Ecuador | Tiputini | NA | 7 | AMN | (Harrigan *et al*., 2014) |
| KJ661253 | -0,168 | -77,973 | *Haemoproteus* sp. | *Phaethornis* sp. | Ecuador | San Rafael | NA | 7 | NAN | (Harrigan *et al*., 2014) |
| KJ661254 | -0,168 | -77,973 | *Haemoproteus* sp. | *Phaethornis* sp. | Ecuador | San Rafael | NA | 7 | NAN | (Harrigan *et al*., 2014) |
| KJ661256 | -0,599 | -77,89 | *Haemoproteus* sp. | *Diglossa cyanea* | Ecuador | Yanayacu | NA | 7 | NAN | (Harrigan *et al*., 2014) |
| KJ661257 | -0,599 | -77,89 | *Haemoproteus* sp. | *Diglossa cyanea* | Ecuador | Yanayacu | NA | 7 | NAN | (Harrigan *et al*., 2014) |
| KJ661258 | -0,599 | -77,89 | *Haemoproteus* sp. | *Diglossa cyanea* | Ecuador | Yanayacu | NA | 7 | NAN | (Harrigan *et al*., 2014) |
| KJ661262 | -0,599 | -77,89 | *Haemoproteus* sp. | *Diglossa cyanea* | Ecuador | Cerro Bosco | NA | 7 | NAN | (Harrigan *et al*., 2014) |
| KJ661263 | -0,599 | -77,89 | *Haemoproteus* sp. | *Diglossa cyanea* | Ecuador | Cerro Bosco | NA | 7 | NAN | (Harrigan *et al*., 2014) |
| KJ661264 | -0,599 | -77,89 | *Haemoproteus* sp. | *Diglossa cyanea* | Ecuador | Cerro Bosco | NA | 7 | NAN | (Harrigan *et al*., 2014) |
| KJ661269 | -0,077 | -78,755 | *Haemoproteus* sp. | *Phaethornis syrmatophorus* | Ecuador | Mindo | NA | 7 | NAN | (Harrigan *et al*., 2014) |
| KJ661270 | -0,077 | -78,755 | *Haemoproteus* sp. | *Phaethornis syrmatophorus* | Ecuador | Mindo | NA | 7 | NAN | (Harrigan *et al*., 2014) |
| KJ661272 | -0,011 | -78,688 | *Haemoproteus* sp. | *Diglossa cyanea* | Ecuador | Bellavista | NA | 7 | NAN | (Harrigan *et al*., 2014) |
| KJ661273 | -0,011 | -78,688 | *Haemoproteus* sp. | *Diglossa cyanea* | Ecuador | Bellavista | NA | 7 | NAN | (Harrigan *et al*., 2014) |
| KJ661274 | -0,011 | -78,688 | *Haemoproteus* sp. | *Diglossa cyanea* | Ecuador | Bellavista | NA | 7 | NAN | (Harrigan *et al*., 2014) |
| KJ661275 | -4,48 | -79,12 | *Haemoproteus* sp. | *Diglossa cyanea* | Ecuador | Tapichalaca | NA | 7 | NAN | (Harrigan *et al*., 2014) |
| KJ661277 | -2,765 | -79,459 | *Haemoproteus* sp. | *Diglossa cyanea* | Ecuador | Sural | NA | 7 | NAN | (Harrigan *et al*., 2014) |
| KJ661278 | -2,765 | -79,459 | *Haemoproteus* sp. | *Diglossa cyanea* | Ecuador | Sural | NA | 7 | NAN | (Harrigan *et al*., 2014) |
| KJ661279 | -2,765 | -79,459 | *Haemoproteus* sp. | *Diglossa cyanea* | Ecuador | Sural | NA | 7 | NAN | (Harrigan *et al*., 2014) |
| KJ661280 | -2,765 | -79,459 | *Haemoproteus* sp. | *Diglossa cyanea* | Ecuador | Sural | NA | 7 | NAN | (Harrigan *et al*., 2014) |
| KJ661281 | -2,765 | -79,459 | *Haemoproteus* sp. | *Diglossa cyanea* | Ecuador | Sural | NA | 7 | NAN | (Harrigan *et al*., 2014) |
| KJ661282 | -2,765 | -79,459 | *Haemoproteus* sp. | *Diglossa cyanea* | Ecuador | Sural | NA | 7 | NAN | (Harrigan *et al*., 2014) |
| KJ661284 | -2,887 | -79,427 | *Haemoproteus* sp. | *Diglossa cyanea* | Ecuador | Chuacha | NA | 7 | NAN | (Harrigan *et al*., 2014) |
| KJ661286 | -2,887 | -79,429 | *Haemoproteus* sp. | *Diglossa cyanea* | Ecuador | Chuacha | NA | 7 | NAN | (Harrigan *et al*., 2014) |
| KJ661287 | -2,887 | -79,43 | *Haemoproteus* sp. | *Diglossa cyanea* | Ecuador | Chuacha | NA | 7 | NAN | (Harrigan *et al*., 2014) |
| KJ661288 | -0,245 | -78,802 | *Haemoproteus* sp. | *Diglossa cyanea* | Ecuador | Guajalito | NA | 7 | NAN | (Harrigan *et al*., 2014) |
| KJ661289 | -0,245 | -78,802 | *Haemoproteus* sp. | *Diglossa cyanea* | Ecuador | Guajalito | NA | 7 | NAN | (Harrigan *et al*., 2014) |
| KJ661291 | -0,011 | -78,688 | *Haemoproteus* sp. | *Diglossa cyanea* | Ecuador | Bellavista | NA | 7 | NAN | (Harrigan *et al*., 2014) |
| KJ661292 | -0,011 | -78,688 | *Haemoproteus* sp. | *Adelomyia melanogenys* | Ecuador | Bellavista | NA | 7 | NAN | (Harrigan *et al*., 2014) |
| KJ661293 | -0,011 | -78,688 | *Haemoproteus* sp. | *Diglossa cyanea* | Ecuador | Bellavista | NA | 7 | NAN | (Harrigan *et al*., 2014) |
| KJ661294 | 0,655 | -78,032 | *Haemoproteus* sp. | *Diglossa cyanea* | Ecuador | Guandera | NA | 7 | NAN | (Harrigan *et al*., 2014) |
| KJ661295 | 0,655 | -78,032 | *Haemoproteus* sp. | *Diglossa cyanea* | Ecuador | Guandera | NA | 7 | NAN | (Harrigan *et al*., 2014) |
| KJ661296 | 0,655 | -78,032 | *Haemoproteus* sp. | *Diglossa cyanea* | Ecuador | Guandera | NA | 7 | NAN | (Harrigan *et al*., 2014) |
| KJ661297 | 0,655 | -78,032 | *Haemoproteus* sp. | *Diglossa cyanea* | Ecuador | Guandera | NA | 7 | NAN | (Harrigan *et al*., 2014) |
| KJ661298 | 0,656 | -78,031 | *Haemoproteus* sp. | *Diglossa cyanea* | Ecuador | Guandera | NA | 7 | NAN | (Harrigan *et al*., 2014) |
| KJ661302 | -0,077 | -78,755 | *Haemoproteus* sp. | *Phaethornis syrmatophorus* | Ecuador | Mindo | NA | 7 | NAN | (Harrigan *et al*., 2014) |
| KJ661303 | -0,077 | -78,755 | *Haemoproteus* sp. | *Phaethornis syrmatophorus* | Ecuador | Mindo | NA | 7 | NAN | (Harrigan *et al*., 2014) |
| KJ661304 | -1,838 | -80,611 | *Haemoproteus* sp. | *Phaethornis baroni* | Ecuador | Loma Alta | NA | 7 | NAN | (Harrigan *et al*., 2014) |
| KJ661307 | 0,362 | -79,716 | *Haemoproteus* sp. | *Phaethornis yaruqui* | Ecuador | Bilsa | NA | 7 | EPC | (Harrigan *et al*., 2014) |
| KJ661309 | 0,362 | -79,716 | *Haemoproteus* sp. | *Phaethornis yaruqui* | Ecuador | Bilsa | NA | 7 | EPC | (Harrigan *et al*., 2014) |
| KJ661312 | -0,077 | -78,755 | *Haemoproteus* sp. | *Phaethornis yaruqui* | Ecuador | Mindo | NA | 7 | NAN | (Harrigan *et al*., 2014) |
| KJ661313 | -0,077 | -78,755 | *Haemoproteus* sp. | *Phaethornis striigularis* | Ecuador | Mindo | NA | 7 | NAN | (Harrigan *et al*., 2014) |
| KJ661314 | -0,077 | -78,755 | *Haemoproteus* sp. | *Phaethornis striigularis* | Ecuador | Mindo | NA | 7 | NAN | (Harrigan *et al*., 2014) |
| KJ661318 | -0,637 | -76,149 | *Haemoproteus* sp. | *Adelomyia melanogenys* | Ecuador | Tiputini | NA | 7 | AMN | (Harrigan *et al*., 2014) |
| KJ661324 | -2,765 | -79,459 | *Haemoproteus* sp. | *Diglossa cyanea* | Ecuador | Sural | NA | 7 | NAN | (Harrigan *et al*., 2014) |
| KJ661325 | -2,765 | -79,459 | *Haemoproteus* sp. | *Diglossa cyanea* | Ecuador | Sural | NA | 7 | NAN | (Harrigan *et al*., 2014) |
| KT698209 | 4,633 | -74,083 | *Haemoproteus erythrogravidus* | *Zonotrichia capensis* | Colombia | Bogotá, D.C. | NA | 7 | NAN | (Mantilla *et al*., 2016) |
| KU364580 | -4,387 | -79,146 | *Haemoproteus witti* | *Coeligena iris* | Ecuador | Podocarpus National Park | TROAED20 | 7 | NAN | (Moens *et al*., 2016) |
| KU364581 | -4,258 | -79,217 | *Haemoproteus witti* | *Basileuterus trifasciatus* | Ecuador | Podocarpus National Park | TROAED20 | 7 | NAN | (Moens *et al*., 2016) |
| KU364582 | -4,258 | -79,217 | *Haemoproteus witti* | *Myiothlypis fraseri* | Ecuador | Podocarpus National Park | TROAED20 | 7 | NAN | (Moens *et al*., 2016) |
| KU364583 | -4,258 | -79,217 | *Haemoproteus witti* | *Pachyramphus albogriseus* | Ecuador | Podocarpus National Park | TROAED20 | 7 | NAN | (Moens *et al*., 2016) |
| KU364584 | -4,258 | -79,217 | *Haemoproteus witti* | *Tyrannus melancholicus* | Ecuador | Podocarpus National Park | TROAED20 | 7 | NAN | (Moens *et al*., 2016) |
| KF537329 | 4,711 | -74,072 | *Haemoproteus* sp. | *Zonotrichia capensis* | Colombia | Bogotá, D.C. | ZOCAP10 | 7 | NAN | (Mantilla *et al*., 2016) |
| GQ141581 | NA | NA | *Parahaemoproteus* sp. | *Cardinalis cardinalis* | North America Continental | NA | CARCAR01 | 8 | NA | (Galen and Witt, 2014) |
| GQ141563 | NA | NA | *Parahaemoproteus* sp. | *Spindalis dominicensis* | Antilles | NA | SPIDOM01 | 9 | NA | (Galen and Witt, 2014) |
| GU256261 | NA | NA | *Parahaemoproteus* sp. | *Spindalis dominicensis* | Antilles | NA | NA | 9 | NA | (Galen and Witt, 2014) |
| KJ910306 | 37,09 | -95,712 | *Haemoproteus* sp. | *Spizella passerina* | United States of America | NA | NA | 10 | NA | (Ricklefs *et al*., 2017) |
| MF077649 | 35,827 | -106,896 | *Haemoproteus* sp. | *Spizella passerina* | United States of America | Nuevo Mexico, Elk Springs | SPIPAS02 | 10 | NA | (Marroquin-Flores *et al*., 2017) |
| MF077649 | 34,836 | -108,223 | *Haemoproteus* sp. | *Tyrannus vociferans* | United States of America | Nuevo Mexico, El Malpais | SPIPAS02 | 10 | NA | (Marroquin-Flores *et al*., 2017) |
| MF077650 | 35,533 | -107,349 | *Haemoproteus* sp. | *Spizella passerina* | United States of America | Nuevo Mexico, Mesa Chivato | SPIPAS01 | 10 | NA | (Marroquin-Flores *et al*., 2017) |
| MF077650 | 35,533 | -107,350 | *Haemoproteus* sp. | *Spinus psaltria* | United States of America | Nuevo Mexico, Mesa Chivato | SPIPAS01 | 10 | NA | (Marroquin-Flores *et al*., 2017) |
| GQ141589 | NA | NA | *Parahaemoproteus* sp. | *Dendroica pensylvanica* | North America Continental | NA | DENPEN02 | 10 | NA | (Galen and Witt, 2014) |
| AF465568 | NA | NA | *Haemoproteus* sp. | *Tiaris bicolor* | Sur America | NA | TIABIC01 | 11 | NA | (Galen and Witt, 2014) |
| JN792147 | 36,778 | -119,418 | *Haemoproteus* sp. | *Catharus ustulatus* | United States of America | California | NA | 12 | NA | (Galen and Witt, 2014) |
| AF465548 | 64,2 | -149,493 | *Plasmodium* sp. | *Turdus migratorius* | United States of America | Alaska | TUMIG03 | 13 | NA | (Mantilla *et al*., 2013) |
| DQ241523 | -32,522 | -55,765 | *Plasmodium* sp. | *Turdus rufiventris* | Uruguay | NA | 16 | 13 | PAM | (Durrant *et al*., 2006) |
| DQ241524 | -32,522 | -55,765 | *Plasmodium* sp. | *Turdus rufiventris* | Uruguay | NA | 17 | 13 | PAM | (Durrant *et al*., 2006) |
| EF153639 | -41,867 | -73,828 | *Plasmodium* sp. | *Turdus falcklandii* | Chile | Ancud | TUMIG03 | 13 | SAN | (Mantilla *et al*., 2013) |
| EF153639 | -41,867 | -73,828 | *Plasmodium* sp. | *Anairetes fernandezianus* | Chile | Ancud | TUMIG03 | 13 | SAN | (Mantilla *et al*., 2013) |
| HM222478 | 32,318 | -86,902 | *Plasmodium* sp. | *Hylocichla mustelina* | United States of America | Alabama | OZ35 | 13 | NA | (Mantilla *et al*., 2013; Marzal *et al*., 2015) |
| HM222479 | 32,318 | -86,902 | *Plasmodium* sp. | *Hylocichla mustelina* | United States of America | Alabama | OZ35 | 13 | NA | (Mantilla *et al*., 2013; Marzal *et al*., 2015) |
| JN792135 | 64,2 | -149,493 | *Plasmodium* sp. | *Catharus ustulatus* | United States of America | Alaska | TUMIG03 | 13 | NA | (Mantilla *et al*., 2013) |
| JN819328 | 9,748 | -83,753 | *Plasmodium* sp. | *Turdus assimilis* | Costa Rica | NA | NA | 13 | CDH | (Mantilla *et al*., 2013) |
| JN819328 | 10,829 | -73,692 | *Plasmodium* sp. | *Turdus flavipes* | Colombia | Sierra Nevada de Santa Marta, San Lorenzo ridge | NA | 13 | NAN | (Gonzalez-Quevedo, Rivera-Gutierrez & Pabón, 2016) |
| JN819328 | 10,829 | -73,692 | *Plasmodium* sp. | *Turdus olivater* | Colombia | Sierra Nevada de Santa Marta, San Lorenzo ridge | NA | 13 | NAN | (Gonzalez-Quevedo, Rivera-Gutierrez & Pabón, 2016) |
| JN819340 | 9,748 | -83,753 | *Plasmodium* sp. | *Tangara icterocephala* | Costa Rica | NA | NA | 13 | CDH | (Mantilla *et al*., 2013) |
| JN819343 | 9,748 | -83,753 | *Plasmodium* sp. | *Turdus assimilis* | Costa Rica | NA | NA | 13 | CDH | (Mantilla *et al*., 2013) |
| JN819347 | 9,748 | -83,753 | *Plasmodium* sp. | *Turdus assimilis* | Costa Rica | NA | NA | 13 | CDH | (Mantilla *et al*., 2013) |
| JX021462 | -17,111 | -43,82 | *Plasmodium* sp. | *Turdus amaurochalinus* | Brazil | Bocaiúva | TUMIG03 | 13 | CSA | (Lacorte *et al*., 2013) |
| JX021462 | -18,713 | -44,925 | *Plasmodium* sp. | *Turdus amaurochalinus* | Brazil | Felixlândia | TUMIG03 | 13 | CSA | (Lacorte *et al*., 2013) |
| JX021462 | -19,993 | -43,848 | *Plasmodium* sp. | *Turdus amaurochalinus* | Brazil | Nova Lima | TUMIG03 | 13 | CSA | (Lacorte *et al*., 2013) |
| JX021473 | -19,789 | -42,141 | *Plasmodium* sp. | *Turdus rufiventris* | Brazil | Caratinga | TURUF01 | 13 | CSA | (Lacorte *et al*., 2013) |
| JX021473 | -19,993 | -43,848 | *Plasmodium* sp. | *Turdus rufiventris* | Brazil | Nova Lima | TURUF01 | 13 | CSA | (Lacorte *et al*., 2013) |
| KC771247 | 4,711 | -74,072 | *Plasmodium unalis* | *Turdus fuscater* | Colombia | Bogotá, D.C. | TFUS06 | 13 | NAN | (Mantilla *et al*., 2013) |
| KF182186 | 4,711 | -74,072 | *Plasmodium unalis* | *Turdus fuscater* | Colombia | Bogotá, D.C. | TFUS06 | 13 | NAN | (Mantilla *et al*., 2013) |
| KF537293 | 4,804 | -75,713 | *Plasmodium unalis* | *Turdus fuscater* | Colombia | Pereira | TFUS06 | 13 | NAN | (González *et al*., 2015) |
| KF537324 | 4,804 | -75,713 | *Plasmodium unalis* | *Turdus fuscater* | Colombia | Pereira | TFUS06 | 13 | NAN | (González *et al*., 2015) |
| KF767414 | -17,32 | -70,247 | *Plasmodium* sp. | *Troglodytes aedon* | Peru | Tacna | TUMIG03 | 13 | CAN | (Galen and Witt, 2014; Bensch *et al*., 2009) |
| KF767415 | -17,39 | -70,345 | *Plasmodium* sp. | *Troglodytes aedon* | Peru | Tacna | TUMIG03 | 13 | CAN | (Galen and Witt, 2014; Bensch *et al*., 2009) |
| KU562788 | -13,8 | -59,683 | *Plasmodium* sp. | *Turdus amaurochalinus* | Brazil | Comodoro | NA | 13 | CSA | (Fecchio *et al*., 2017) |
| JN819328 | 9,748 | -83,753 | *Plasmodium* sp. | *Tangara icterocephala* | Costa Rica | NA | NA | 13 | CDH | (Mantilla *et al*., 2013) |
| JN819383 | 9,748 | -83,753 | *Haemoproteus* sp. | *Turdus assimilis* | Costa Rica | NA | TURDUS2 | 14 | CDH | (Galen and Witt, 2014) |
| JN819399 | 9,748 | -83,753 | *Haemoproteus* sp. | *Turdus assimilis* | Costa Rica | NA | NA | 14 | CDH | (Galen and Witt, 2014) |
| JQ988577 | -13,249 | -72,169 | *Parahaemoproteus* sp. | *Ramphocelus carbo* | Peru | Cusco | NA | 14 | CAN | (Galen and Witt, 2014) |
| JN819378 | 9,748 | -83,753 | *Haemoproteus* sp. | *Turdus assimilis* | Costa Rica | NA | NA | 14 | CDH | (Galen and Witt, 2014) |
| KC680715 | -0,633 | -76,133 | *Haemoproteus* sp. | *Philydor erythropterus* | Ecuador | Tiputini Biodiversity Station, Orellana Province | H14 | 15 | AMN | (Svensson-Coelho *et al*. 2016) |
| KC680674 | -0,633 | -76,133 | *Plasmodium* sp. | *Gymnopithys leucaspis* | Ecuador | Tiputini Biodiversity Station, Orellana Province | NA | 16 | AMN | (Ricklefs *et al*., 2017) |
| KC680703 | -0,633 | -76,133 | *Plasmodium* sp. | *Lepidothrix coronata* | Ecuador | Tiputini Biodiversity Station, Orellana Province | P30 | 16 | AMN | (Svensson-Coelho *et al*. 2016) |
| KC680703 | -0,633 | -76,133 | *Plasmodium* sp. | *Lepidothrix coronata* | Ecuador | Tiputini Biodiversity Station, Orellana Province | NA | 16 | AMN | (Ricklefs *et al*., 2017) |
| KC680699 | -0,633 | -76,133 | *Plasmodium* sp. | *Gymnopithys leucaspis* | Ecuador | Tiputini Biodiversity Station, Orellana Province | P32L | 16 | AMN | (Svensson-Coelho *et al*. 2016) |
| AY167250 | 13,909 | -60,978 | *Plasmodium* sp. | *Vireo altiloquus* | Antilles | Lesser Antilles - Saint Lucia Island | PU1 | 17 | LAN | (Fallon *et al*., 2005) |
| AY640137 | -23,65 | -46,617 | *Plasmodium nucleophilum* | *Alopochen aegyptiaca* | Brazil | São Paulo Zoo | NA | 17 | ATL | (Chagas *et al*., 2017) |
| AY640137 | -23,65 | -46,617 | *Plasmodium nucleophilum* | *Cygnus atratus* | Brazil | São Paulo Zoo | NA | 17 | ATL | (Chagas *et al*., 2017) |
| AY640137 | -23,65 | -46,617 | *Plasmodium nucleophilum* | *Netta erythrophthalma* | Brazil | São Paulo Zoo | NA | 17 | ATL | (Chagas *et al*., 2017) |
| AY640137 | -23,65 | -46,617 | *Plasmodium nucleophilum* | *Pipile jacutinga* | Brazil | São Paulo Zoo | NA | 17 | ATL | (Chagas *et al*., 2017) |
| AY640137 | -23,65 | -46,617 | *Plasmodium nucleophilum* | *Psarocolius decumanus* | Brazil | São Paulo Zoo | NA | 17 | ATL | (Chagas *et al*., 2017) |
| AY640137 | -23,65 | -46,617 | *Plasmodium nucleophilum* | *Phoenicopterus chilensis* | Brazil | São Paulo Zoo | NA | 17 | ATL | (Chagas *et al*., 2017) |
| AY640137 | -23,65 | -46,617 | *Plasmodium nucleophilum* | *Ramphastos toco* | Brazil | São Paulo Zoo | NA | 17 | ATL | (Chagas *et al*., 2017) |
| AY640137 | -23,65 | -46,617 | *Plasmodium nucleophilum* | *Guarouba guarouba* | Brazil | São Paulo Zoo | NA | 17 | ATL | (Chagas *et al*., 2017) |
| AY640137 | -3,944 | -73,607 | *Plasmodium* sp. | *Cacicus cela* | Peru | Allpahuayo Mishana National Reserve | NA | 17 | AMN | (Ricopa & Villa, 2016) |
| AY640137 | -3,944 | -73,607 | *Plasmodium* sp. | *Manacus manacus* | Peru | Allpahuayo Mishana National Reserve | NA | 17 | AMN | (Ricopa & Villa, 2016) |
| AY640137 | -3,944 | -73,607 | *Plasmodium* sp. | *Willisornis poecilinotus* | Peru | Allpahuayo Mishana National Reserve | NA | 17 | AMN | (Ricopa & Villa, 2016) |
| AY640137 | -3,944 | -73,607 | *Plasmodium* sp. | *Myiarchus ferox* | Peru | Allpahuayo Mishana National Reserve | NA | 17 | AMN | (Ricopa & Villa, 2016) |
| HQ287545 | -10,249 | -48,324 | *Plasmodium* sp. | *Tachyphonus luctuosus* | Brazil | Palmas,Tocantins | Toc-15 | 17 | CSA | (Belo *et al*., 2011) |
| AY640137 | NA | NA | *Plasmodium* sp. | *Dendroica petechia* | USA, Guyana, Uruguay, Brazil | NA | NA | 17 | NA | (Szymanski and Lovette, 2005) |
| AY167247 | 14,609 | -61,072 | *Plasmodium* sp. | *Coereba flaveola* | Antilles | Lesser Antilles - Martinique Island | PA | 18 | LAN | (Fallon *et al*., 2005; Fallon *et al*., 2003) |
| AY167247 | 13,909 | -60,978 | *Plasmodium* sp. | *Coereba flaveola* | Antilles | Lesser Antilles - Saint Lucia Island | PA | 18 | LAN | (Fallon *et al*., 2005; Fallon *et al*., 2003) |
| AY167247 | 13,909 | -60,978 | *Plasmodium* sp. | *Loxigilla noctis* | Antilles | Lesser Antilles - Saint Lucia Island | PA | 18 | LAN | (Fallon *et al*., 2005; Fallon *et al*., 2003) |
| AY167247 | 16,742 | -62,187 | *Plasmodium* sp. | *Loxigilla noctis* | Antilles | Lesser Antilles - Montserrat Island | PA | 18 | LAN | (Fallon *et al*., 2005; Fallon *et al*., 2003) |
| AY167247 | 15,439 | -61,346 | *Plasmodium* sp. | *Coereba flaveola* | Antilles | Lesser Antilles - Dominica Island | PA | 18 | LAN | (Fallon *et al*., 2005; Fallon *et al*., 2003) |
| AY167247 | 15,439 | -61,346 | *Plasmodium* sp. | *Loxigilla noctis* | Antilles | Lesser Antilles - Dominica Island | PA | 18 | LAN | (Fallon *et al*., 2005; Fallon *et al*., 2003) |
| AY167247 | 16,129 | -61,653 | *Plasmodium* sp. | *Coereba flaveola* | Antilles | Lesser Antilles - Guadeloupe Island | PA | 18 | LAN | (Fallon *et al*., 2005; Fallon *et al*., 2003) |
| HQ287549 | -10,249 | -48,324 | *Plasmodium* sp. | *Saltator atricollis* | Brazil | Palmas,Tocantins | Toc-32 | 18 | CSA | (Belo *et al*., 2011) |
| KC680698 | -0,633 | -76,133 | *Plasmodium* sp. | *Gymnopithys leucaspis* | Ecuador | Tiputini Biodiversity Station, Orellana Province | P37L | 18 | AMN | (Svensson-Coelho *et al*. 2016) |
| KC680691 | -0,633 | -76,133 | *Plasmodium* sp. | *Myrmotherula longipennis* | Ecuador | Tiputini Biodiversity Station, Orellana Province | P24L | 19 | AMN | (Svensson-Coelho *et al*. 2016) |
| EF153650 | -32,835 | -70,701 | *Haemoproteus* sp. | *Phrygilus alaudinus* | Chile | Rinconada | ZOCAP02 | 20 | SAN | (Galen and Witt, 2014) |
| DQ241512 | 4,86 | -58,93 | *Plasmodium* sp. | *Emberizoides herbicola* | Guyana | NA | 5 | 21 | AMN | (Durrant *et al*., 2006) |
| DQ241512 | 4,86 | -58,93 | *Plasmodium* sp. | *Sturnella militaris* | Guyana | NA | 5 | 21 | AMN | (Durrant *et al*., 2006) |
| DQ241513 | -32,522 | -55,765 | *Plasmodium* sp. | *Gnorimopsar chopi* | Uruguay | NA | 6 | 21 | PAM | (Durrant *et al*., 2006) |
| DQ241513 | -32,522 | -55,765 | *Plasmodium* sp. | *Pseudoleistes guirahuro* | Uruguay | NA | 6 | 21 | PAM | (Durrant *et al*., 2006) |
| DQ241513 | -32,522 | -55,765 | *Plasmodium* sp. | *Tangara preciosa* | Uruguay | NA | 6 | 21 | PAM | (Durrant *et al*., 2006) |
| DQ241513 | -32,522 | -55,765 | *Plasmodium* sp. | *Stephanophorus diadematus* | Uruguay | NA | 6 | 21 | PAM | (Durrant *et al*., 2006) |
| DQ241513 | -32,522 | -55,765 | *Plasmodium* sp. | *Troglodytes aedon* | Uruguay | NA | 6 | 21 | PAM | (Durrant *et al*., 2006) |
| DQ241513 | -32,522 | -55,765 | *Plasmodium* sp. | *Troglodytes aedon* | Uruguay | NA | 6 | 21 | PAM | (Durrant *et al*., 2006) |
| DQ241513 | -32,522 | -55,765 | *Plasmodium* sp. | *Troglodytes aedon* | Uruguay | NA | 6 | 21 | PAM | (Durrant *et al*., 2006) |
| DQ241513 | -32,522 | -55,765 | *Plasmodium* sp. | *Turdus rufiventris* | Uruguay | NA | 6 | 21 | PAM | (Durrant *et al*., 2006) |
| JX021461 | -17,0003 | -46,008 | *Plasmodium* sp. | *Myiodynastes maculatus* | Brazil | Brasilandia de Minas | MYITYR01 | 21 | CSA | (Lacorte *et al*., 2013) |
| JX021461 | -18,713 | -44,925 | *Plasmodium* sp. | *Myiodynastes maculatus* | Brazil | Felixlândia | MYITYR01 | 21 | CSA | (Lacorte *et al*., 2013) |
| JX021461 | -16,11 | -40,022 | *Plasmodium* sp. | *Myiarchus tuberculifer* | Brazil | Salto da Divisa | MYITYR01 | 21 | CSA | (Lacorte *et al*., 2013) |
| JX021461 | -16,11 | -40,022 | *Plasmodium* sp. | *Myiarchus tyrannulus* | Brazil | Salto da Divisa | MYITYR01 | 21 | CSA | (Lacorte *et al*., 2013) |
| JX025076 | -19,82 | -40,276 | *Plasmodium* sp. | *Troglodytes musculus* | Brazil | Aracruz | PADOM09 | 21 | ATL | (Lacorte *et al*., 2013) |
| JX025076 | -19,82 | -40,276 | *Plasmodium* sp. | *Tyrannus melancholicus* | Brazil | Aracruz | PADOM09 | 21 | ATL | (Lacorte *et al*., 2013) |
| JX025076 | -17,111 | -43,82 | *Plasmodium* sp. | *Basileuterus culicivorus* | Brazil | Bocaiúva | PADOM09 | 21 | CSA | (Lacorte *et al*., 2013) |
| JX025076 | -17,111 | -43,82 | *Plasmodium* sp. | *Lathrotriccus euleri* | Brazil | Bocaiúva | PADOM09 | 21 | CSA | (Lacorte *et al*., 2013) |
| JX025076 | -17,0003 | -46,008 | *Plasmodium* sp. | *Cnemotriccus fuscatus* | Brazil | Brasilandia de Minas | PADOM09 | 21 | CSA | (Lacorte *et al*., 2013) |
| JX025076 | -17,0003 | -46,008 | *Plasmodium* sp. | *Myiopagis viridicata* | Brazil | Brasilandia de Minas | PADOM09 | 21 | CSA | (Lacorte *et al*., 2013) |
| JX025076 | -19,789 | -42,141 | *Plasmodium* sp. | *Trichothraupis melanops* | Brazil | Caratinga | PADOM09 | 21 | CSA | (Lacorte *et al*., 2013) |
| JX025076 | -18,713 | -44,925 | *Plasmodium* sp. | *Cnemotriccus fuscatus* | Brazil | Felixlândia | PADOM09 | 21 | CSA | (Lacorte *et al*., 2013) |
| JX025076 | -18,713 | -44,925 | *Plasmodium* sp. | *Elaenia cristata* | Brazil | Felixlândia | PADOM09 | 21 | CSA | (Lacorte *et al*., 2013) |
| JX025076 | -18,713 | -44,925 | *Plasmodium* sp. | *Lathrotriccus euleri* | Brazil | Felixlândia | PADOM09 | 21 | CSA | (Lacorte *et al*., 2013) |
| JX025076 | -16,437 | -41,012 | *Plasmodium* sp. | *Cnemotriccus fuscatus* | Brazil | Jequitinhonha | PADOM09 | 21 | CSA | (Lacorte *et al*., 2013) |
| JX025076 | -16,437 | -41,012 | *Plasmodium* sp. | *Myiopagis viridicata* | Brazil | Jequitinhonha | PADOM09 | 21 | CSA | (Lacorte *et al*., 2013) |
| JX025076 | -16,11 | -40,022 | *Plasmodium* sp. | *Myiophobus fasciatus* | Brazil | Salto da Divisa | PADOM09 | 21 | CSA | (Lacorte *et al*., 2013) |
| JX025076 | -16,11 | -40,022 | *Plasmodium* sp. | *Pheugopedius genibarbis* | Brazil | Salto da Divisa | PADOM09 | 21 | CSA | (Lacorte *et al*., 2013) |
| JX025076 | -16,11 | -40,022 | *Plasmodium* sp. | *Pitangus sulphuratus* | Brazil | Salto da Divisa | PADOM09 | 21 | CSA | (Lacorte *et al*., 2013) |
| JX029887 | -17,0003 | -46,008 | *Plasmodium* sp. | *Furnarius rufus* | Brazil | Brasilandia de Minas | FURUF01 | 21 | CSA | (Lacorte *et al*., 2013) |
| JX029893 | -19,82 | -40,276 | *Plasmodium* sp. | *Pheugopedius genibarbis* | Brazil | Aracruz | THAMB09 | 21 | ATL | (Lacorte *et al*., 2013) |
| JX029893 | -19,82 | -40,276 | *Plasmodium* sp. | *Thamnophilus ambiguus* | Brazil | Aracruz | THAMB09 | 21 | ATL | (Lacorte *et al*., 2013) |
| KC867648 | 43,193 | -71,572 | *Plasmodium* sp. | *Dolichonyx oryzivorus* | United States of America | New Hampshire - Platte River | PADOM09 | 21 | NA | (Levin *et al*., 2013) |
| KC867649 | 43,265 | -118,845 | *Plasmodium* sp. | *Dolichonyx oryzivorus* | United States of America | Oregon - Malheur National Wildlife Refuge | PADOM09 | 21 | NA | (Levin *et al*., 2013) |
| KC867650 | 44,451 | -73,122 | *Plasmodium* sp. | *Dolichonyx oryzivorus* | United States of America | Vermont - northwestern Vermont | MYITYR01 | 21 | NA | (Levin *et al*., 2013) |
| KC867651 | -0,412 | -91,482 | *Plasmodium* sp. | *Setophaga petechia* | Ecuador | Galápagos Islands - Fernandina Island | MYITYR01 | 21 | NAN | (Levin *et al*., 2013) |
| KC867652 | 44,451 | -73,122 | *Plasmodium* sp. | *Dolichonyx oryzivorus* | United States of America | Vermont - northwestern Vermont | DOLORY01 | 21 | NA | (Levin *et al*., 2013) |
| KF767409 | -8,386 | -78,645 | *Plasmodium* sp. | *Troglodytes aedon* | Peru | La Libertad | PADOM09 P4 | 21 | CAN | (Galen and Witt, 2014) |
| KF767410 | -5,896 | -79,785 | *Plasmodium* sp. | *Troglodytes aedon* | Peru | Lambayeque | PADOM09 P4 | 21 | CAN | (Galen and Witt, 2014) |
| KF767411 | -8,386 | -78,645 | *Plasmodium* sp. | *Troglodytes aedon* | Peru | La Libertad | PADOM09 P4 | 21 | CAN | (Galen and Witt, 2014) |
| KU562526 | -6,602 | -40,124 | *Plasmodium* sp. | *Lepidocolaptes angustirostris* | Brazil | Aiuaba | NA | 21 | CSA | (Fecchio *et al*., 2017) |
| KU562554 | -15,53 | -47,55 | *Plasmodium elongatum* | *Elaenia cristata* | Brazil | Planaltina | NA | 21 | CSA | (Fecchio *et al*., 2017) |
| KU562555 | -15,53 | -47,55 | *Plasmodium elongatum* | *Elaenia cristata* | Brazil | Planaltina | NA | 21 | CSA | (Fecchio *et al*., 2017) |
| KU562556 | -15,53 | -47,55 | *Plasmodium elongatum* | *Neothraupis fasciata* | Brazil | Planaltina | NA | 21 | CSA | (Fecchio *et al*., 2017) |
| KU562557 | -5,923 | -35,175 | *Plasmodium elongatum* | *Myiarchus tyrannulus* | Brazil | Ponta Negra | NA | 21 | ATL | (Fecchio *et al*., 2017) |
| KU562558 | -19,567 | -57,017 | *Plasmodium elongatum* | *Paroaria capitata* | Brazil | Corumbá | NA | 21 | CSA | (Fecchio *et al*., 2017) |
| KU562559 | -19,567 | -57,017 | *Plasmodium elongatum* | *Saltator coerulescens* | Brazil | Corumbá | NA | 21 | CSA | (Fecchio *et al*., 2017) |
| KU562560 | -19,567 | -57,017 | *Plasmodium elongatum* | *Donacobius atricapilla* | Brazil | Corumbá | NA | 21 | CSA | (Fecchio *et al*., 2017) |
| KU562561 | -3,7 | -46,75 | *Plasmodium elongatum* | *Tachyphonus cristatus* | Brazil | Gurupi | NA | 21 | AMS | (Fecchio *et al*., 2017) |
| KU562562 | -3,7 | -46,75 | *Plasmodium elongatum* | *Pheugopedius genibarbis* | Brazil | Gurupi | NA | 21 | AMS | (Fecchio *et al*., 2017) |
| KU562563 | -1,35 | -56,367 | *Plasmodium elongatum* | *Dendrocolaptes certhia* | Brazil | Porto Trombetas | NA | 21 | AMN | (Fecchio *et al*., 2017) |
| KU562564 | -12,217 | -60,73 | *Plasmodium elongatum* | *Cnemotriccus fuscatus* | Brazil | Chupinguaia | NA | 21 | AMS | (Fecchio *et al*., 2017) |
| KU562565 | -12,217 | -60,73 | *Plasmodium elongatum* | *Rhytipterna simplex* | Brazil | Chupinguaia | NA | 21 | AMS | (Fecchio *et al*., 2017) |
| KU562566 | -9,133 | -64,633 | *Plasmodium elongatum* | *Ramphotrigon ruficauda* | Brazil | Madeira River | NA | 21 | AMS | (Fecchio *et al*., 2017) |
| KU562574 | -15,53 | -47,55 | *Plasmodium* sp. | *Neothraupis fasciata* | Brazil | Planaltina | NA | 21 | CSA | (Fecchio *et al*., 2017) |
| KU562594 | -0,583 | -64,917 | *Plasmodium* sp. | *Automolus infuscatus* | Brazil | Negro River | NA | 21 | AMN | (Fecchio *et al*., 2017) |
| KU562608 | -0,583 | -64,917 | *Plasmodium* sp. | *Myrmotherula longipennis* | Brazil | Negro River | NA | 21 | AMN | (Fecchio *et al*., 2017) |
| KU562609 | -5,717 | -63,2 | *Plasmodium* sp. | *Myrmoborus myotherinus* | Brazil | Purus River | NA | 21 | AMN | (Fecchio *et al*., 2017) |
| KU562654 | -5,717 | -63,2 | *Plasmodium* sp. | *Thamnophilus aethiops* | Brazil | Purus River | NA | 21 | AMN | (Fecchio *et al*., 2017) |
| KU562655 | -6,583 | -37,267 | *Plasmodium* sp. | *Myiarchus tyrannulus* | Brazil | Jucurutu | NA | 21 | CSA | (Fecchio *et al*., 2017) |
| KU562656 | -6,583 | -37,267 | *Plasmodium* sp. | *Myiarchus tyrannulus* | Brazil | Jucurutu | NA | 21 | CSA | (Fecchio *et al*., 2017) |
| KU562657 | -6,583 | -37,267 | *Plasmodium* sp. | *Myiarchus tyrannulus* | Brazil | Jucurutu | NA | 21 | CSA | (Fecchio *et al*., 2017) |
| KU562658 | -3,7 | -46,75 | *Plasmodium* sp. | *Rhytipterna simplex* | Brazil | Gurupi | NA | 21 | AMS | (Fecchio *et al*., 2017) |
| KU562659 | -3,7 | -46,75 | *Plasmodium* sp. | *Campylorhynchus turdinus* | Brazil | Gurupi | NA | 21 | AMS | (Fecchio *et al*., 2017) |
| KU562680 | -5,923 | -35,175 | *Plasmodium* sp. | *Schistochlamys ruficapillus* | Brazil | Ponta Negra | NA | 21 | ATL | (Fecchio *et al*., 2017) |
| KU562681 | -12,567 | -70,083 | *Plasmodium* sp. | *Myrmoborus myotherinus* | Peru | Manu | NA | 21 | CAN | (Fecchio *et al*., 2017) |
| DQ241513 | -32,522 | -55,765 | *Plasmodium* sp. | *Gnorimopsar chopi* | Uruguay | NA | 6 | 21 | PAM | (Durrant *et al*., 2006) |
| DQ241529 | 4,86 | -58,93 | *Plasmodium* sp. | *Cyanocompsa cyanoides* | Guyana | NA | 22 | 22 | AMN | (Durrant *et al*., 2006) |
| DQ241529 | 4,86 | -58,93 | *Plasmodium* sp. | *Icterus cayanensis* | Guyana | NA | 22 | 22 | AMN | (Durrant *et al*., 2006) |
| JX029894 | -19,098 | -40,186 | *Plasmodium* sp. | *Formicarius colma* | Brazil | Sooretama | FOCOL01 | 22 | ATL | (Lacorte *et al*., 2013) |
| JX029895 | -19,098 | -40,186 | *Plasmodium* sp. | *Formicarius colma* | Brazil | Sooretama | FOCOL02 | 22 | ATL | (Lacorte *et al*., 2013) |
| KU236432 | -3,684 | -60,314 | *Plasmodium* sp. | *Lepidothrix coronata* | Brazil | Careiro-Castanho | LECOR02 | 22 | AMN | (Bosholn *et al*., 2016) |
| KU562328 | -5,1 | -56,43 | *Plasmodium* sp. | *Thamnomanes caesius* | Brazil | Jamanxim River | NA | 22 | AMS | (Fecchio *et al*., 2017) |
| KU562329 | -4,500 | -56,283 | *Plasmodium* sp. | *Thamnomanes caesius* | Brazil | Tapajόs River | NA | 22 | AMS | (Fecchio *et al*., 2017) |
| KU562330 | -4,700 | -56,53 | *Plasmodium* sp. | *Hypocnemis striata* | Brazil | Tapajόs River | NA | 22 | AMS | (Fecchio *et al*., 2017) |
| KU562331 | -5,217 | -56,917 | *Plasmodium* sp. | *Thamnomanes saturninus* | Brazil | Tapajόs River | NA | 22 | AMS | (Fecchio *et al*., 2017) |
| KU562332 | -4,683 | -56,63 | *Plasmodium* sp. | *Myrmornis torquata* | Brazil | Tapajόs River | NA | 22 | AMS | (Fecchio *et al*., 2017) |
| KU562333 | -4,683 | -56,63 | *Plasmodium* sp. | *Myrmoborus myotherinus* | Brazil | Tapajόs River | NA | 22 | AMS | (Fecchio *et al*., 2017) |
| KU562334 | -0,583 | -64,917 | *Plasmodium* sp. | *Hypocnemis striata* | Brazil | Negro River | NA | 22 | AMN | (Fecchio *et al*., 2017) |
| KU562335 | -0,583 | -64,917 | *Plasmodium* sp. | *Lepidothrix coronata* | Brazil | Negro River | NA | 22 | AMN | (Fecchio *et al*., 2017) |
| KU562336 | -0,583 | -64,917 | *Plasmodium* sp. | *Sclerurus caudacutus* | Brazil | Negro River | NA | 22 | AMN | (Fecchio *et al*., 2017) |
| KU562337 | -0,583 | -64,917 | *Plasmodium* sp. | *Microbates collaris* | Brazil | Negro River | NA | 22 | AMN | (Fecchio *et al*., 2017) |
| KU562338 | -1,35 | -56,367 | *Plasmodium* sp. | *Geotrygon montana* | Brazil | Porto Trombetas | NA | 22 | AMN | (Fecchio *et al*., 2017) |
| KU562339 | -1,35 | -56,367 | *Plasmodium* sp. | *Dixiphia pipra* | Brazil | Porto Trombetas | NA | 22 | AMN | (Fecchio *et al*., 2017) |
| KU562340 | -1,35 | -56,367 | *Plasmodium* sp. | *Dixiphia pipra* | Brazil | Porto Trombetas | NA | 22 | AMN | (Fecchio *et al*., 2017) |
| KU562341 | -1,35 | -56,367 | *Plasmodium* sp. | *Dixiphia pipra* | Brazil | Porto Trombetas | NA | 22 | AMN | (Fecchio *et al*., 2017) |
| KU562342 | -1,35 | -56,367 | *Plasmodium* sp. | *Hylexetastes perrotii* | Brazil | Porto Trombetas | NA | 22 | AMN | (Fecchio *et al*., 2017) |
| KU562343 | -1,35 | -56,367 | *Plasmodium* sp. | *Dixiphia pipra* | Brazil | Porto Trombetas | NA | 22 | AMN | (Fecchio *et al*., 2017) |
| KU562344 | -1,35 | -56,367 | *Plasmodium* sp. | *Pithys albifrons* | Brazil | Porto Trombetas | NA | 22 | AMN | (Fecchio *et al*., 2017) |
| KU562345 | -1,35 | -56,367 | *Plasmodium* sp. | *Pithys albifrons* | Brazil | Porto Trombetas | NA | 22 | AMN | (Fecchio *et al*., 2017) |
| KU562429 | -4,683 | -56,63 | *Plasmodium* sp. | *Hypocnemis striata* | Brazil | Tapajόs River | NA | 22 | AMS | (Fecchio *et al*., 2017) |
| KU562430 | -4,683 | -56,63 | *Plasmodium* sp. | *Willisornis poecilinotus* | Brazil | Tapajόs River | NA | 22 | AMS | (Fecchio *et al*., 2017) |
| KU562431 | -5,717 | -63,2 | *Plasmodium* sp. | *Gymnopithys salvini* | Brazil | Purus River | NA | 22 | AMN | (Fecchio *et al*., 2017) |
| KU562601 | -0,583 | -64,917 | *Plasmodium* sp. | *Thamnomanes caesius* | Brazil | Negro River | NA | 22 | AMN | (Fecchio *et al*., 2017) |
| KU562644 | -4,983 | -62,13 | *Plasmodium* sp. | *Lepidothrix coronata* | Brazil | Purus River | NA | 22 | AMN | (Fecchio *et al*., 2017) |
| DQ241528 | 4,86 | -58,93 | *Plasmodium* sp. | *Butorides striata* | Guyana | NA | 21 | 22 | AMN | (Durrant *et al*., 2006) |
| DQ241515 | -32,522 | -55,765 | *Plasmodium* sp. | *Embernagra platensis* | Uruguay | NA | 8 | 23 | PAM | (Durrant *et al*., 2006) |
| DQ241515 | -32,522 | -55,765 | *Plasmodium* sp. | *Embernagra platensis* | Uruguay | NA | 8 | 23 | PAM | (Durrant *et al*., 2006) |
| DQ241519 | -32,522 | -55,765 | *Plasmodium* sp. | *Coryphistera alaudina* | Uruguay | NA | 12 | 24 | PAM | (Durrant *et al*., 2006) |
| DQ241519 | -32,522 | -55,765 | *Plasmodium* sp. | *Limnornis curvirostris* | Uruguay | NA | 12 | 24 | PAM | (Durrant *et al*., 2006) |
| DQ241519 | -32,522 | -55,765 | *Plasmodium* sp. | *Gnorimopsar chopi* | Uruguay | NA | 12 | 24 | PAM | (Durrant *et al*., 2006) |
| DQ241519 | -32,522 | -55,765 | *Plasmodium* sp. | *Mimus saturninus* | Uruguay | NA | 12 | 24 | PAM | (Durrant *et al*., 2006) |
| DQ241519 | -32,522 | -55,765 | *Plasmodium* sp. | *Stephanophorus diadematus* | Uruguay | NA | 12 | 24 | PAM | (Durrant *et al*., 2006) |
| DQ241520 | 4,86 | -58,93 | *Plasmodium* sp. | *Icterus nigrogularis* | Guyana | NA | 13 | 24 | AMN | (Durrant *et al*., 2006) |
| DQ241520 | 4,86 | -58,93 | *Plasmodium* sp. | *Cacicus cela* | Guyana | NA | 13 | 24 | AMN | (Durrant *et al*., 2006) |
| KC867664 | 43,193 | -71,572 | *Plasmodium* sp. | *Dolichonyx oryzivorus* | United States of America | New Hampshire - Platte River | COLL4 | 24 | NA | (Levin *et al*., 2013) |
| KU562839 | -12,567 | -70,083 | *Plasmodium* sp. | *Glyphorynchus spirurus* | Peru | Manu | NA | 24 | CAN | (Fecchio *et al*., 2017) |
| DQ241519 | -32,522 | -55,765 | *Plasmodium* sp. | *Microspingus lateralis* | Uruguay | NA | 12 | 24 | PAM | (Durrant *et al*., 2006) |
| DQ241550 | -32,522 | -55,765 | *Haemoproteus* sp. | *Paroaria coronata* | Uruguay | NA | MONGUT02 | 25 | PAM | (Galen and Witt, 2014; Durrant *et al*., 2006) |
| HQ287542 | -10,249 | -48,324 | *Plasmodium* sp. | *Turdus leucomelas* | Brazil | Palmas,Tocantins | Toc-9 | 26 | CSA | (Belo *et al*., 2011) |
| AY455660 | 16,235 | -61,488 | *Plasmodium* sp. | *Elaenia martinica* | Antilles | NA | PD | 27 | LAN | (Fallon *et al*., 2005) |
| HQ287539 | -10,249 | -48,324 | *Plasmodium* sp. | *Pipra fasciicauda* | Brazil | Palmas,Tocantins | Toc4 | 27 | CSA | (Belo *et al*., 2011) |
| JX501902 | -15,53 | -47,55 | *Haemoproteus* sp. | *Elaenia chiriquensis* | Brazil | Águas Emendadas Station Ecologic, Distrito Federal | NA | 28 | CSA | (Ricklefs *et al*., 2017) |
| KC680711 | -0,633 | -76,133 | *Haemoproteus* sp. | *Phlegopsis erythroptera* | Ecuador | Tiputini Biodiversity Station, Orellana Province | H10 | 28 | AMN | (Svensson-Coelho *et al*. 2016) |
| KC680711 | -0,633 | -76,133 | *Haemoproteus* sp. | *Phlegopsis erythroptera* | Ecuador | Tiputini Biodiversity Station, Orellana Province | NA | 28 | AMN | (Ricklefs *et al*., 2017) |
| HQ287536 | -10,249 | -48,324 | *Haemoproteus* sp. | *Hemitriccus margaritaceiventer* | Brazil | Palmas,Tocantins | Toc1 | 28 | CSA | (Belo *et al*., 2011) |
| JX501789 | -15,53 | -47,55 | *Haemoproteus* sp. | *Cypsnagra hirundinacea* | Brazil | Águas Emendadas Station Ecologic, Distrito Federal | NA | 29 | CSA | (Ricklefs *et al*., 2017) |
| HQ287538 | -10,249 | -48,324 | *Haemoproteus* sp. | *Volatinia jacarina* | Brazil | Palmas,Tocantins | Toc3 | 29 | CSA | (Belo *et al*., 2011) |
| EF153654 | -32,835 | -70,701 | *Haemoproteus* sp. | *Phrygilus fruticeti* | Chile | Rinconada | PHFRU01 | 30 | SAN | (Galen and Witt, 2014) |
| EF153641 | -36,833 | -72,55 | *Plasmodium* sp. | *Troglodytes musculus* | Chile | Pantanillo | ChP4 | 31 | SAN | (Cadena *et al*., 2015; Merino *et al*., 2008) |
| EF153641 | -36,833 | -72,55 | *Plasmodium* sp. | *Elaenia albiceps* | Chile | Pantanillo | ChP4 | 31 | SAN | (Cadena *et al*., 2015; Merino *et al*., 2008) |
| EF153641 | -36,833 | -72,55 | *Plasmodium* sp. | *Zonotrichia capensis* | Chile | Pantanillo | ChP4 | 31 | SAN | (Cadena *et al*., 2015; Merino *et al*., 2008) |
| EF153641 | -0,0013 | -78,355 | *Plasmodium* sp. | *Zonotrichia capensis* | Ecuador | Quito | ChP4 | 31 | NAN | (Cadena *et al*., 2015) |
| KF482349 | -3,944 | -73,607 | *Plasmodium* sp. | *Ammodramus aurifrons* | Peru | Allpahuayo Mishana National Reserve | NA | 31 | AMN | (Ricopa & Villa, 2016) |
| KF537286 | 4,804 | -75,713 | *Plasmodium homopolare* | *Zonotrichia capensis* | Colombia | Pereira | BAEBIC02 | 31 | NAN | (Walther *et al*., 2014; González *et al*., 2015) |
| KF537289 | 4,804 | -75,713 | *Plasmodium homopolare* | *Anisognathus somptuosus* | Colombia | Pereira | BAEBIC02 | 31 | NAN | (Walther *et al*., 2014; González *et al*., 2015) |
| KF537289 | 4,804 | -75,713 | *Plasmodium homopolare* | *Zonotrichia capensis* | Colombia | Pereira | BAEBIC02 | 31 | NAN | (Walther *et al*., 2014; González *et al*., 2015) |
| EF153641 | -36,833 | -72,55 | *Plasmodium* sp. | *Turdus falcklandii* | Chile | Pantanillo | ChP4 | 31 | SAN | (Cadena *et al*., 2015; Merino *et al*., 2008) |
| KC680667 | -0,633 | -76,133 | *Plasmodium* sp. | *Myrmoborus myotherinus* | Ecuador | Tiputini Biodiversity Station, Orellana Province | NA | 32 | AMN | (Ricklefs *et al*., 2017) |
| EF153648 | -33,408 | -70,567 | *Haemoproteus* sp. | *Zonotrichia capensis* | Chile | Navarino | ChH3 | 33 | SAN | (Galen and Witt, 2014) |
| EF153648 | -0,0013 | -78,355 | *Haemoproteus* sp. | *Zonotrichia capensis* | Ecuador | Quito | ChH3 | 33 | NAN | (Cadena *et al*., 2015) |
| EF153652 | -0,0013 | -78,355 | *Haemoproteus* sp. | *Zonotrichia capensis* | Ecuador | Quito | ChH6 | 33 | NAN | (Cadena *et al*., 2015) |
| EF153652 | -32,835 | -70,701 | *Haemoproteus* sp. | *Troglodytes musculus* | Chile | Rinconada | ChH6 | 33 | SAN | (Cadena *et al*., 2015; Merino *et al*., 2008) |
| EF153652 | -32,835 | -70,701 | *Haemoproteus* sp. | *Elaenia albiceps* | Chile | Rinconada | ChH6 | 33 | SAN | (Cadena *et al*., 2015; Merino *et al*., 2008) |
| EF153652 | -32,835 | -70,701 | *Haemoproteus* sp. | *Turdus falcklandii* | Chile | Rinconada | ChH6 | 33 | SAN | (Cadena *et al*., 2015; Merino *et al*., 2008) |
| EF153652 | -32,835 | -70,701 | *Haemoproteus* sp. | *Zonotrichia capensis* | Chile | Rinconada | ChH6 | 33 | SAN | (Cadena *et al*., 2015; Merino *et al*., 2008) |
| EF153652 | -32,835 | -70,701 | *Haemoproteus* sp. | *Aphrastura spinicauda* | Chile | Rinconada | ChH6 | 33 | SAN | (Cadena *et al*., 2015; Merino *et al*., 2008) |
| KC480265 | -0,0013 | -78,355 | *Haemoproteus* sp. | *Zonotrichia capensis* | Ecuador | Quito | ZOCAP08 | 33 | NAN | (Cadena *et al*., 2015) |
| KC480265 | -13,937 | -75,8 | *Haemoproteus* sp. | *Zonotrichia capensis* | Peru | Ica | ZOCAP08 | 33 | STP | (Galen and Witt, 2014; Jones *et al*., 2013) |
| EF153643 | -32,835 | -70,701 | *Plasmodium* sp. | *Aphrastura spinicauda* | Chile | Rinconada | ChP6 | 34 | SAN | (Cadena *et al*., 2015; Merino *et al*., 2008) |
| EF153643 | -32,835 | -70,701 | *Plasmodium* sp. | *Elaenia albiceps* | Chile | Rinconada | ChP6 | 34 | SAN | (Cadena *et al*., 2015; Merino *et al*., 2008) |
| EF153643 | -32,835 | -70,701 | *Plasmodium* sp. | *Zonotrichia capensis* | Chile | Rinconada | ChP6 | 34 | SAN | (Cadena *et al*., 2015; Merino *et al*., 2008) |
| KU236434 | -3,686 | -60,316 | *Plasmodium* sp. | *Lepidothrix coronata* | Brazil | Careiro-Castanho | LECOR04 | 34 | AMN | (Bosholn *et al*., 2016) |
| EF153643 | -0,0013 | -78,355 | *Plasmodium* sp. | *Zonotrichia capensis* | Ecuador | Quito | ChP6 | 34 | NAN | (Cadena *et al*., 2015) |
| KU057966 | -23,65 | -46,617 | *Plasmodium* sp. | *Dendrocygna viduata* | Brazil | São Paulo Zoo | NA | 35 | ATL | (Chagas *et al*., 2016) |
| KU057966 | -23,65 | -46,617 | *Plasmodium* sp. | *Cygnus atratus* | Brazil | São Paulo Zoo | NA | 35 | ATL | (Chagas *et al*., 2017) |
| KU057966 | -23,65 | -46,617 | *Plasmodium* sp. | *Tadorna ferruginea* | Brazil | São Paulo Zoo | NA | 35 | ATL | (Chagas *et al*., 2017) |
| KU057966 | -23,65 | -46,617 | *Plasmodium* sp. | *Pavo cristatus* | Brazil | São Paulo Zoo | NA | 35 | ATL | (Chagas *et al*., 2017) |
| KU057966 | -23,65 | -46,617 | *Plasmodium* sp. | *Pavo muticus* | Brazil | São Paulo Zoo | NA | 35 | ATL | (Chagas *et al*., 2017) |
| KU057966 | -23,65 | -46,617 | *Plasmodium* sp. | *Alopochen aegyptiaca* | Brazil | São Paulo Zoo | NA | 35 | ATL | (Chagas *et al*., 2017) |
| DQ241526 | 4,86 | -58,93 | *Plasmodium* sp. | *Volatinia jacarina* | Guyana | NA | 19 | 36 | AMN | (Durrant *et al*., 2006) |
| JX501809 | -15,53 | -47,55 | *Plasmodium* sp. | *Turdus amaurochalinus* | Brazil | Águas Emendadas Station Ecologic, Distrito Federal | NA | 36 | CSA | (Ricklefs *et al*., 2017) |
| KX171626 | -23,65 | -46,617 | *Plasmodium* sp. | *Nothocrax urumutum* | Brazil | São Paulo Zoo | NA | 37 | ATL | (Chagas *et al*., 2017) |
| KC680686 | -0,633 | -76,133 | *Haemoproteus* sp. | *Pithys albifrons* | Ecuador | Tiputini Biodiversity Station, Orellana Province | H11 | 38 | AMN | (Svensson-Coelho *et al*. 2016) |
| MF990712 | 2,967 | -78,184 | *Haemoproteus* sp. | *Cyanerpes cyaneus* | Colombia | Cauca, PNN Gorgona, El Poblado | NA | 39 | CHO | In this studio |
| MF990713 | 2,967 | -78,184 | *Haemoproteus* sp. | *Cyanerpes cyaneus* | Colombia | Cauca, PNN Gorgona, El Poblado | NA | 39 | CHO | In this studio |
| MF990714 | 2,967 | -78,184 | *Haemoproteus* sp. | *Coereba flaveola* | Colombia | Cauca, PNN Gorgona, El Poblado | NA | 39 | CHO | In this studio |
| MF990715 | 2,967 | -78,184 | *Haemoproteus* sp. | *Cyanerpes cyaneus* | Colombia | Cauca, PNN Gorgona, El Poblado | NA | 39 | CHO | In this studio |
| MF990716 | 2,967 | -78,184 | *Haemoproteus* sp. | *Cyanerpes cyaneus* | Colombia | Cauca, PNN Gorgona, El Poblado | NA | 39 | CHO | In this studio |
| MF990717 | 4,59 | -75,86 | *Haemoproteus* sp. | *Thamnophilus atrinucha* | Colombia | Quindio, Quimbaya, Reserva Natural La montaña del Ocaso | NA | 39 | NAN | In this studio |
| MF990718 | 2,967 | -78,184 | *Haemoproteus* sp. | *Thamnophilus atrinucha* | Colombia | Cauca, PNN Gorgona, El Poblado | NA | 39 | CHO | In this studio |
| MF990719 | 2,967 | -78,184 | *Haemoproteus* sp. | *Cyanerpes cyaneus* | Colombia | Cauca, PNN Gorgona, El Poblado | NA | 39 | CHO | In this studio |
| MF990720 | 2,967 | -78,184 | *Haemoproteus* sp. | *Cyanerpes cyaneus* | Colombia | Cauca, PNN Gorgona, El Poblado | NA | 39 | CHO | In this studio |
| MF990722 | 2,967 | -78,184 | *Haemoproteus* sp. | *Cyanerpes cyaneus* | Colombia | Cauca, PNN Gorgona, El Poblado | NA | 39 | CHO | In this studio |
| MF990723 | 2,967 | -78,184 | *Haemoproteus* sp. | *Cyanerpes cyaneus* | Colombia | Cauca, PNN Gorgona, El Poblado | NA | 39 | CHO | In this studio |
| MF990724 | 2,967 | -78,184 | *Haemoproteus* sp. | *Thamnophilus atrinucha* | Colombia | Cauca, PNN Gorgona, El Poblado | NA | 39 | CHO | In this studio |
| MF990725 | 2,967 | -78,184 | *Haemoproteus* sp. | *Cyanerpes cyaneus* | Colombia | Cauca, PNN Gorgona, El Poblado | NA | 39 | CHO | In this studio |
| MF990726 | 2,967 | -78,184 | *Haemoproteus* sp. | *Cyanerpes cyaneus* | Colombia | Cauca, PNN Gorgona, El Poblado | NA | 39 | CHO | In this studio |
| MF990728 | 2,967 | -78,184 | *Haemoproteus* sp. | *Cyanerpes cyaneus* | Colombia | Cauca, PNN Gorgona, El Poblado | NA | 39 | CHO | In this studio |
| MF990729 | 2,967 | -78,184 | *Haemoproteus* sp. | *Tyrannus melancholicus* | Colombia | Cauca, PNN Gorgona, El Poblado | NA | 39 | CHO | In this studio |
| MF990731 | 2,967 | -78,184 | *Haemoproteus* sp. | *Cyanerpes cyaneus* | Colombia | Cauca, PNN Gorgona, El Poblado | NA | 39 | CHO | In this studio |
| MF990733 | 2,967 | -78,184 | *Haemoproteus* sp. | *Coereba flaveola* | Colombia | Cauca, PNN Gorgona, El Poblado | NA | 39 | CHO | In this studio |
| MF990734 | 2,967 | -78,184 | *Haemoproteus* sp. | *Actitis macularia* | Colombia | Cauca, PNN Gorgona, El Poblado | NA | 39 | CHO | In this studio |
| MF990732 | 2,967 | -78,184 | *Haemoproteus* sp. | *Cyanerpes cyaneus* | Colombia | Cauca, PNN Gorgona, El Poblado | NA | 39 | CHO | In this studio |
| KC480266 | -0,0013 | -78,355 | *Haemoproteus* sp. | *Zonotrichia capensis* | Ecuador | Quito | ZOCAP01 | 40 | NAN | (Cadena *et al*., 2015) |
| KM211348 | 4,804 | -75,713 | *Haemoproteus* sp. | *Diglossa caerulescens* | Colombia | Pereira | DICER01 | 40 | NAN | (González *et al*., 2015) |
| KC480266 | -12,928 | -75,154 | *Haemoproteus* sp. | *Zonotrichia capensis* | Peru | Huancavelica | ZOCAP01 | 40 | CAN | (Jones *et al*., 2013) |
| KF537290 | 4,804 | -75,713 | *Plasmodium* sp. | *Trogon personatus* | Colombia | Pereira | TRPER01 | 41 | NAN | (González *et al*., 2015) |
| JN819393 | 9,748 | -83,753 | *Haemoproteus* sp. | *Turdus assimilis* | Costa Rica | NA | COLL2 | 42 | CDH | (Galen and Witt, 2014) |
| KJ661259 | -0,599 | -77,89 | *Haemoproteus* sp. | *Adelomyia melanogenys* | Ecuador | Yanayacu | NA | 42 | NAN | (Harrigan *et al*., 2014) |
| JN819388 | 9,748 | -83,753 | *Haemoproteus* sp. | *Tangara icterocephala* | Costa Rica | NA | TURDUS2 | 42 | CDH | (Galen and Witt, 2014) |
| KF537276 | 4,804 | -75,713 | *Plasmodium lutzi* | *Diglossa lafresnayii* | Colombia | Pereira | DIALB01 | 43 | NAN | (González *et al*., 2015) |
| KF537277 | 4,804 | -75,713 | *Plasmodium lutzi* | *Diglossa lafresnayii* | Colombia | Pereira | DILAF01 | 43 | NAN | (González *et al*., 2015) |
| KF537284 | 4,804 | -75,713 | *Plasmodium lutzi* | *Anisognathus lacrymosus* | Colombia | Pereira | DIALB01 | 43 | NAN | (González *et al*., 2015) |
| KF537305 | 4,804 | -75,713 | *Plasmodium lutzi* | *Diglossa cyanea* | Colombia | Pereira | DIALB01 | 43 | NAN | (González *et al*., 2015) |
| KF537307 | 4,804 | -75,713 | *Plasmodium lutzi* | *Diglossa cyanea* | Colombia | Pereira | DIALB01 | 43 | NAN | (González *et al*., 2015) |
| KF537313 | 4,804 | -75,713 | *Plasmodium lutzi* | *Diglossa albilatera* | Colombia | Pereira | DIALB01 | 43 | NAN | (González *et al*., 2015) |
| KF767406 | -17,32 | -70,247 | *Plasmodium* sp. | *Troglodytes aedon* | Peru | Tacna | CATUST05 P5 | 43 | CAN | (Galen and Witt, 2014) |
| KF767407 | -17,32 | -70,247 | *Plasmodium* sp. | *Troglodytes aedon* | Peru | Tacna | CATUST05 P5 | 43 | CAN | (Galen and Witt, 2014) |
| KF767408 | -17,32 | -70,247 | *Plasmodium* sp. | *Troglodytes aedon* | Peru | Tacna | CATUST05 P5 | 43 | CAN | (Galen and Witt, 2014) |
| KJ527079 | -9,190 | -75,015 | *Plasmodium* sp. | *Merganetta armata* | Peru | NA | NA | 43 | NAN | (Smith and Ramey 2015) |
| KJ661266 | -0,599 | -77,89 | *Plasmodium* sp. | *Diglossa cyanea* | Ecuador | Cerro Bosco | NA | 43 | NAN | (Harrigan *et al*., 2014) |
| KJ661276 | -4,48 | -79,12 | *Plasmodium* sp. | *Diglossa cyanea* | Ecuador | Tapichalaca | NA | 43 | NAN | (Harrigan *et al*., 2014) |
| KJ780795 | 4,804 | -75,713 | *Plasmodium lutzi* | *Diglossa cyanea* | Colombia | Pereira | DIALB01 | 43 | NAN | (González *et al*., 2015) |
| KJ780795 | 10,829 | -73,692 | *Plasmodium* sp. | *Diglossa humeralis* | Colombia | Sierra Nevada de Santa Marta, San Lorenzo ridge | NA | 43 | NAN | (Gonzalez-Quevedo, Rivera-Gutierrez & Pabón, 2016) |
| KM211353 | 4,804 | -75,713 | *Plasmodium lutzi* | *Hemispingus verticalis* | Colombia | Pereira | DIALB01 | 43 | NAN | (González *et al*., 2015) |
| KF537312 | 4,804 | -75,713 | *Plasmodium lutzi* | *Turdus fuscater* | Colombia | Pereira | TFUS05 | 43 | NAN | (González *et al*., 2015) |
| KX171622 | -23,65 | -46,617 | *Plasmodium* sp. | *Aramides cajaneus* | Brazil | São Paulo Zoo | NA | 44 | ATL | (Chagas *et al*., 2017) |
| KM211349 | 4,804 | -75,713 | *Haemoproteus* sp. | *Buthraupis montana* | Colombia | Pereira | DICER01 | 45 | NAN | (González *et al*., 2015) |
| KM211349 | 10,829 | -73,692 | *Haemoproteus* sp. | *Atlapetes melanocephalus* | Colombia | Sierra Nevada de Santa Marta, San Lorenzo ridge | NA | 45 | NAN | (Gonzalez-Quevedo, Rivera-Gutierrez & Pabón, 2016) |
| KM211352 | 4,804 | -75,713 | *Haemoproteus* sp. | *Hemispingus atropileus* | Colombia | Pereira | HEMATRO01 | 45 | NAN | (González *et al*., 2015) |
| KM211346 | 4,804 | -75,713 | *Haemoproteus* sp. | *Buthraupis montana* | Colombia | Pereira | BUTMON02 | 45 | NAN | (González *et al*., 2015) |
| KF537300 | 4,804 | -75,713 | *Haemoproteus* sp. | *Anisognathus igniventris* | Colombia | Pereira | ANIGN01 | 46 | NAN | (González *et al*., 2015) |
| KF537301 | 4,804 | -75,713 | *Haemoproteus* sp. | *Anisognathus igniventris* | Colombia | Pereira | ANIGN01 | 46 | NAN | (González *et al*., 2015) |
| KF537302 | 4,804 | -75,713 | *Haemoproteus* sp. | *Anisognathus igniventris* | Colombia | Pereira | ANIGN01 | 46 | NAN | (González *et al*., 2015) |
| KF537303 | 4,804 | -75,713 | *Haemoproteus* sp. | *Anisognathus igniventris* | Colombia | Pereira | ANIGN01 | 46 | NAN | (González *et al*., 2015) |
| KF537311 | 4,804 | -75,713 | *Haemoproteus* sp. | *Anisognathus igniventris* | Colombia | Pereira | ANIGN01 | 46 | NAN | (González *et al*., 2015) |
| KF537306 | 4,804 | -75,713 | *Haemoproteus* sp. | *Anisognathus igniventris* | Colombia | Pereira | ANIGN01 | 46 | NAN | (González *et al*., 2015) |
| KC680668 | -0,633 | -76,133 | *Haemoproteus* sp. | *Myrmotherula axillaris* | Ecuador | Tiputini Biodiversity Station, Orellana Province | H8 | 47 | AMN | (Svensson-Coelho *et al*. 2016) |
| AF465555 | 9,748 | -83,753 | *Plasmodium* sp. | *Tangara icterocephala* | Costa Rica | NA | BAEBIC02 | 48 | CDH | (Walther *et al*., 2014) |
| AF465555 | 9,748 | -83,753 | *Plasmodium* sp. | *Baeolophus bicolor* | Costa Rica | NA | BAEBIC02 | 48 | CDH | (Walther *et al*., 2014) |
| AF465555 | -9,916 | -76,233 | *Plasmodium* sp. | *Zonotrichia capensis* | Peru | Huánuco | BAEBIC02 | 48 | CAN | (Marzal *et al*., 2015) |
| JQ764622 | 10,231 | -67,285 | *Plasmodium* sp. | *Seiurus noveboracensis* | Venezuela | Aragua | PVE5 | 48 | NAN | (Mijares *et al*., 2012) |
| JQ988537 | -6,649 | -76,072 | *Plasmodium* sp. | *Troglodytes aedon* | Peru | San Martín | BAEBIC02 P1 | 48 | CAN | (Galen and Witt, 2014) |
| JQ988539 | -14,173 | -73,323 | *Plasmodium* sp. | *Troglodytes aedon* | Peru | Apurímac | BAEBIC02 P1 | 48 | CAN | (Galen and Witt, 2014) |
| JQ988540 | -6,104 | -78,341 | *Plasmodium* sp. | *Troglodytes aedon* | Peru | Amazonas | BAEBIC02 P2 | 48 | CAN | (Galen and Witt, 2014) |
| JQ988540 | -14,173 | -73,323 | *Plasmodium* sp. | *Troglodytes aedon* | Peru | Apurímac | BAEBIC02 P2 | 48 | CAN | (Galen and Witt, 2014) |
| JQ988540 | -13,249 | -72,169 | *Plasmodium* sp. | *Troglodytes aedon* | Peru | Cusco | BAEBIC02 P2 | 48 | CAN | (Galen and Witt, 2014) |
| JQ988540 | -11,983 | -74,933 | *Plasmodium* sp. | *Troglodytes aedon* | Peru | Junín | BAEBIC02 P2 | 48 | CAN | (Galen and Witt, 2014) |
| JQ988540 | -6,649 | -76,072 | *Plasmodium* sp. | *Troglodytes aedon* | Peru | San Martín | BAEBIC02 P2 | 48 | CAN | (Galen and Witt, 2014) |
| JQ988550 | -13,249 | -72,169 | *Plasmodium* sp. | *Troglodytes aedon* | Peru | Cusco | BAEBIC02 P1 | 48 | CAN | (Galen and Witt, 2014) |
| JQ988551 | -14,06 | -73,008 | *Plasmodium* sp. | *Troglodytes aedon* | Peru | Apurímac | TROAED21 | 48 | CAN | (Galen and Witt, 2014) |
| KC867674 | -1,308 | -90,432 | *Plasmodium* sp. | *Geospiza fuliginosa* | Ecuador | Galápagos Islands - Floreana Island | LAIRI01 | 48 | NAN | (Levin *et al*., 2013) |
| KC867675 | -1,308 | -90,432 | *Plasmodium* sp. | *Geospiza fuliginosa* | Ecuador | Galápagos Islands - Floreana Island | LAIRI01 | 48 | NAN | (Levin *et al*., 2013) |
| KC867676 | -1,308 | -90,432 | *Plasmodium* sp. | *Geospiza fuliginosa* | Ecuador | Galápagos Islands - Floreana Island | LAIRI01 | 48 | NAN | (Levin *et al*., 2013) |
| KC867677 | 43,193 | -71,572 | *Plasmodium* sp. | *Dolichonyx oryzivorus* | United States of America | New Hampshire - Platte River | LAIRI01 | 48 | NA | (Levin *et al*., 2013) |
| KF482353 | -3,944 | -73,607 | *Plasmodium* sp. | *Ammodramus aurifrons* | Peru | Allpahuayo Mishana National Reserve | NA | 48 | AMN | (Ricopa & Villa, 2016) |
| KF537278 | 4,804 | -75,713 | *Plasmodium homopolare* | *Myiothlypis coronatus* | Colombia | Pereira | BAEBIC02 | 48 | NAN | (Walther *et al*., 2014; González *et al*., 2015) |
| KF537279 | 4,804 | -75,713 | *Plasmodium homopolare* | *Zonotrichia capensis* | Colombia | Pereira | BAEBIC02 | 48 | NAN | (González *et al*., 2015) |
| KF537280 | 4,804 | -75,713 | *Plasmodium homopolare* | *Atlapetes albinucha* | Colombia | Pereira | BAEBIC02 | 48 | NAN | (González *et al*., 2015) |
| KF537281 | 4,804 | -75,713 | *Plasmodium homopolare* | *Zonotrichia capensis* | Colombia | Pereira | BAEBIC02 | 48 | NAN | (Walther *et al*., 2014; González *et al*., 2015) |
| KF537287 | 4,804 | -75,713 | *Plasmodium homopolare* | *Atlapetes albinucha* | Colombia | Pereira | BAEBIC02 | 48 | NAN | (González *et al*., 2015) |
| KF537288 | 4,804 | -75,713 | *Plasmodium homopolare* | *Myiothlypis coronatus* | Colombia | Pereira | BAEBIC02 | 48 | NAN | (Walther *et al*., 2014; González *et al*., 2015) |
| KF537291 | 4,804 | -75,713 | *Plasmodium homopolare* | *Zonotrichia capensis* | Colombia | Pereira | BAEBIC02 | 48 | NAN | (Walther *et al*., 2014; González *et al*., 2015) |
| KF537294 | 4,804 | -75,713 | *Plasmodium homopolare* | *Myiothlypis coronatus* | Colombia | Pereira | BAEBIC02 | 48 | NAN | (Walther *et al*., 2014; González *et al*., 2015) |
| KF767413 | -6,104 | -78,341 | *Plasmodium* sp. | *Troglodytes aedon* | Peru | Amazonas | BAEBIC02 P1 | 48 | CAN | (Galen and Witt, 2014) |
| KJ482708 | 36,778 | -119,418 | *Plasmodium homopolare* | *Passerella iliaca* | United States of America | California | BAEBIC02 | 48 | NA | (Walther *et al*., 2014) |
| KJ482708 | 36,778 | -119,418 | *Plasmodium homopolare* | *Pipilo maculatus* | United States of America | California | BAEBIC02 | 48 | NA | (Walther *et al*., 2014) |
| KJ482708 | 36,778 | -119,418 | *Plasmodium homopolare* | *Geothlypis trichas* | United States of America | California | BAEBIC02 | 48 | NA | (Walther *et al*., 2014) |
| KJ482708 | 36,778 | -119,418 | *Plasmodium homopolare* | *Molothrus ater* | United States of America | California | BAEBIC02 | 48 | NA | (Walther *et al*., 2014) |
| KJ482708 | 36,778 | -119,418 | *Plasmodium homopolare* | *Wilsonia pusilla* | United States of America | California | BAEBIC02 | 48 | NA | (Walther *et al*., 2014) |
| KJ482708 | 36,778 | -119,418 | *Plasmodium homopolare* | *Sitta carolinensis* | United States of America | California | BAEBIC02 | 48 | NA | (Walther *et al*., 2014) |
| KJ482708 | 36,778 | -119,418 | *Plasmodium homopolare* | *Troglodytes aedon* | United States of America | California | BAEBIC02 | 48 | NA | (Walther *et al*., 2014) |
| KJ482708 | 36,778 | -119,418 | *Plasmodium homopolare* | *Melospiza melodia* | United States of America | California | BAEBIC02 | 48 | NA | (Walther *et al*., 2014) |
| KJ482708 | 4,804 | -75,713 | *Plasmodium homopolare* | *Atlapetes albinucha* | Colombia | Pereira | BAEBIC02 | 48 | NAN | (Bensch *et al*., 2009) |
| KJ482708 | 4,804 | -75,713 | *Plasmodium homopolare* | *Zonotrichia capensis* | Colombia | Pereira | BAEBIC02 | 48 | NAN | (Bensch *et al*., 2009) |
| KJ482708 | 4,804 | -75,713 | *Plasmodium homopolare* | *Myiothlypis coronatus* | Colombia | Pereira | BAEBIC02 | 48 | NAN | (Bensch *et al*., 2009) |
| KJ482708 | 4,804 | -75,713 | *Plasmodium homopolare* | *Anisognathus somptuosus* | Colombia | Pereira | BAEBIC02 | 48 | NAN | (Bensch *et al*., 2009) |
| KT373876 | -2,117 | -77,733 | *Plasmodium* sp. | *Phaeothlypis fulvicauda* | Ecuador | Morona-Santiago Province, Wisui | PHAFUL01 | 48 | AMN | (Moens & Pérez-Tris, 2016) |
| KT373876 | -2,087 | -77,751 | *Plasmodium* sp. | *Phaeothlypis fulvicauda* | Ecuador | Wisui reserve | NA | 48 | AMN | (Moens *et al*., 2017) |
| MF077656 | 34,958 | -107,967 | *Plasmodium* sp. | *Spinus psaltria* | United States of America | Nuevo Mexico, El Malpais | LAIRI01 | 48 | NA | (Marroquin-Flores *et al*., 2017) |
| MF077656 | 34,805 | -108,208 | *Plasmodium* sp. | *Tyrannus vociferans* | United States of America | Nuevo Mexico, El Malpais | LAIRI01 | 48 | NA | (Marroquin-Flores *et al*., 2017) |
| MF077656 | 34,812 | -108,208 | *Plasmodium* sp. | *Gymnorhinus cyanocephalus* | United States of America | Nuevo Mexico, El Malpais | LAIRI01 | 48 | NA | (Marroquin-Flores *et al*., 2017) |
| MF077656 | 34,812 | -108,213 | *Plasmodium* sp. | *Thryomanes bewickii* | United States of America | Nuevo Mexico, El Malpais | LAIRI01 | 48 | NA | (Marroquin-Flores *et al*., 2017) |
| MF077656 | 35,534 | -107,350 | *Plasmodium* sp. | *Sialia mexicana* | United States of America | Nuevo Mexico, Mesa Chivato | LAIRI01 | 48 | NA | (Marroquin-Flores *et al*., 2017) |
| MF077656 | 34,836 | -108,225 | *Plasmodium* sp. | *Turdus migratorius* | United States of America | Nuevo Mexico, El Malpais | LAIRI01 | 48 | NA | (Marroquin-Flores *et al*., 2017) |
| JN819334 | 9,748 | -83,753 | *Plasmodium* sp. | *Tangara icterocephala* | Costa Rica | NA | NA | 48 | CDH | (Walther *et al*., 2014) |
| JQ988135 | -6,104 | -78,341 | *Parahaemoproteus* sp. | *Vireo olivaceus* | Peru | Amazonas | NA | 49 | CAN | (Galen and Witt, 2014) |
| KF537331 | 4,804 | -75,713 | *Parahaemoproteus vireonis* | *Vireo olivaceus* | Colombia | Pereira | VIOLI06 | 49 | NAN | (González *et al*., 2015) |
| KF537331 | 10,829 | -73,692 | *Parahaemoproteus vireonis* | *Vireo leucophrys* | Colombia | Sierra Nevada de Santa Marta, San Lorenzo ridge | NA | 49 | NAN | (Gonzalez-Quevedo, Rivera-Gutierrez & Pabón, 2016) |
| KU562167 | -6,602 | -40,124 | *Parahaemoproteus vireonis* | *Cyclarhis gujanensis* | Brazil | Aiuaba | NA | 49 | CSA | (Fecchio *et al*., 2017) |
| KU562168 | -6,602 | -40,124 | *Parahaemoproteus vireonis* | *Lepidocolaptes angustirostris* | Brazil | Aiuaba | NA | 49 | CSA | (Fecchio *et al*., 2017) |
| KU562169 | -6,602 | -40,124 | *Parahaemoproteus vireonis* | *Cyclarhis gujanensis* | Brazil | Aiuaba | NA | 49 | CSA | (Fecchio *et al*., 2017) |
| KU562170 | -6,582 | -37,267 | *Parahaemoproteus vireonis* | *Cyclarhis gujanensis* | Brazil | Serra Negra do Norte | NA | 49 | ATL | (Fecchio *et al*., 2017) |
| KF537320 | 4,804 | -75,713 | *Parahaemoproteus vireonis* | *Vireo olivaceus* | Colombia | Pereira | VIOLI08 | 49 | NAN | (González *et al*., 2015) |
| KF482360 | -3,944 | -73,607 | *Haemoproteus* sp. | *Malacoptila fusca* | Peru | Allpahuayo Mishana National Reserve | NA | 50 | AMN | (Ricopa & Villa, 2016) |
| KF482360 | -3,944 | -73,607 | *Haemoproteus* sp. | *Pithys albifrons* | Peru | Allpahuayo Mishana National Reserve | NA | 50 | AMN | (Ricopa & Villa, 2016) |
| KF482360 | -9,190 | -75,015 | *Haemoproteus* sp. | *Myiopagis flavivertex* | Peru | NA | NA | 50 | NAN | (Marzal, Sehgal & Cardenas, 2013. Sin publicar) |
| MF077662 | 35,533 | -107,350 | *Plasmodium* sp. | *Spizella passerina* | United States of America | Nuevo Mexico, Mesa Chivato | WW3 | 51 | NA | (Marroquin-Flores *et al*., 2017) |
| KC867662 | 43,193 | -71,572 | *Plasmodium* sp. | *Dolichonyx oryzivorus* | United States of America | New Hampshire - Platte River | WW3 | 51 | NA | (Levin *et al*., 2013) |
| DQ241510 | 4,86 | -58,93 | *Plasmodium* sp. | *Cyanocompsa cyanoides* | Guyana | NA | 3 | 52 | AMN | (Durrant *et al*., 2006) |
| KC867654 | 43,193 | -71,572 | *Plasmodium* sp. | *Dolichonyx oryzivorus* | United States of America | New Hampshire - Platte River | DOLORY02 | 52 | NA | (Levin *et al*., 2013) |
| KC867656 | 43,265 | -118,845 | *Plasmodium* sp. | *Dolichonyx oryzivorus* | United States of America | Oregon - Malheur National Wildlife Refuge | SEIAUR01 | 52 | NA | (Levin *et al*., 2013) |
| KC867655 | 44,451 | -73,122 | *Plasmodium* sp. | *Dolichonyx oryzivorus* | United States of America | Vermont - northwestern Vermont | SEIAUR01 | 52 | NA | (Levin *et al*., 2013) |
| JX021470 | -16,11 | -40,022 | *Plasmodium* sp. | *Tiaris fuliginosus* | Brazil | Salto da Divisa | PADOM17 | 53 | CSA | (Lacorte *et al*., 2013) |
| KC867658 | 43,193 | -71,572 | *Plasmodium* sp. | *Dolichonyx oryzivorus* | United States of America | New Hampshire - Platte River | PADOM17 | 53 | NA | (Levin *et al*., 2013) |
| KC867659 | 43,265 | -118,845 | *Plasmodium* sp. | *Dolichonyx oryzivorus* | United States of America | Oregon - Malheur National Wildlife Refuge | PADOM17 | 53 | NA | (Levin *et al*., 2013) |
| KU562532 | -6,602 | -40,124 | *Plasmodium* sp. | *Furnarius leucopus* | Brazil | Aiuaba | NA | 53 | CSA | (Fecchio *et al*., 2017) |
| KU562533 | -15,53 | -47,55 | *Plasmodium* sp. | *Neothraupis fasciata* | Brazil | Planaltina | NA | 53 | CSA | (Fecchio *et al*., 2017) |
| KU562534 | -5,717 | -63,2 | *Plasmodium* sp. | *Automolus infuscatus* | Brazil | Purus River | NA | 53 | AMN | (Fecchio *et al*., 2017) |
| KU562535 | -5,717 | -63,2 | *Plasmodium* sp. | *Galbula cyanicollis* | Brazil | Purus River | NA | 53 | AMN | (Fecchio *et al*., 2017) |
| KU562536 | -5,923 | -35,175 | *Plasmodium* sp. | *Tachyphonus rufus* | Brazil | Ponta Negra | NA | 53 | ATL | (Fecchio *et al*., 2017) |
| KU562537 | -19,567 | -57,017 | *Plasmodium* sp. | *Ramphocelus carbo* | Brazil | Corumbá | NA | 53 | CSA | (Fecchio *et al*., 2017) |
| KU562538 | -19,567 | -57,017 | *Plasmodium* sp. | *Ramphocelus carbo* | Brazil | Corumbá | NA | 53 | CSA | (Fecchio *et al*., 2017) |
| KU562539 | -6,582 | -37,267 | *Plasmodium* sp. | *Coryphospingus pileatus* | Brazil | Serra Negra do Norte | NA | 53 | ATL | (Fecchio *et al*., 2017) |
| KU562540 | -9,33 | -64,683 | *Plasmodium* sp. | *Arremon taciturnus* | Brazil | Madeira River | NA | 53 | AMS | (Fecchio *et al*., 2017) |
| KU562541 | -12,567 | -70,083 | *Plasmodium* sp. | *Gymnopithys salvini* | Peru | Manu | NA | 53 | CAN | (Fecchio *et al*., 2017) |
| KU562708 | -6,582 | -37,267 | *Plasmodium* sp. | *Coryphospingus pileatus* | Brazil | Serra Negra do Norte | NA | 53 | ATL | (Fecchio *et al*., 2017) |
| KU562831 | -12,567 | -70,083 | *Plasmodium* sp. | *Pipra fasciicauda* | Peru | Manu | NA | 53 | CAN | (Fecchio *et al*., 2017) |
| KC867657 | 44,451 | -73,122 | *Plasmodium* sp. | *Dolichonyx oryzivorus* | United States of America | Vermont - northwestern Vermont | PADOM17 | 53 | NA | (Levin *et al*., 2013) |
| KC867680 | 43,193 | -71,572 | *Haemoproteus* sp. | *Dolichonyx oryzivorus* | United States of America | New Hampshire - Platte River | ICTLEU01 | 54 | NA | (Levin *et al*., 2013) |
| KJ561807 | -9,190 | -75,015 | *Haemoproteus* sp. | *Spheniscus humboldti* | Peru | NA | NA | 54 | NAN | (Sallaberry-Pincheira *et al*. 2015) |
| KC867679 | 44,451 | -73,122 | *Haemoproteus* sp. | *Dolichonyx oryzivorus* | United States of America | Vermont - northwestern Vermont | ICTLEU01 | 54 | NA | (Levin *et al*., 2013) |
| DQ241530 | -32,522 | -55,765 | *Plasmodium* sp. | *Zonotrichia capensis* | Uruguay | NA | 23 | 55 | PAM | (Durrant *et al*., 2006) |
| DQ241530 | -32,522 | -55,765 | *Plasmodium* sp. | *Cranioleuca pyrrhophia* | Uruguay | NA | 23 | 55 | PAM | (Durrant *et al*., 2006) |
| DQ241530 | -32,522 | -55,765 | *Plasmodium* sp. | *Gnorimopsar chopi* | Uruguay | NA | 23 | 55 | PAM | (Durrant *et al*., 2006) |
| DQ241530 | -32,522 | -55,765 | *Plasmodium* sp. | *Basileuterus culicivorus* | Uruguay | NA | 23 | 55 | PAM | (Durrant *et al*., 2006) |
| DQ241530 | -32,522 | -55,765 | *Plasmodium* sp. | *Myiothlypis leucoblephara* | Uruguay | NA | 23 | 55 | PAM | (Durrant *et al*., 2006) |
| DQ241530 | -32,522 | -55,765 | *Plasmodium* sp. | *Myiothlypis leucoblephara* | Uruguay | NA | 23 | 55 | PAM | (Durrant *et al*., 2006) |
| DQ241530 | 4,86 | -58,93 | *Plasmodium* sp. | *Volatinia jacarina* | Guyana | NA | B23 | 55 | AMN | (Cadena *et al*., 2015; Durrant *et al*., 2006) |
| DQ241530 | 4,86 | -58,93 | *Plasmodium* sp. | *Cacicus cela* | Guyana | NA | B23 | 55 | AMN | (Cadena *et al*., 2015; Durrant *et al*., 2006) |
| DQ241530 | 4,86 | -58,93 | *Plasmodium* sp. | *Cacicus haemorrhous* | Guyana | NA | B23 | 55 | AMN | (Cadena *et al*., 2015; Durrant *et al*., 2006) |
| DQ241530 | 4,86 | -58,93 | *Plasmodium* sp. | *Diopsittaca nobilis* | Guyana | NA | B23 | 55 | AMN | (Cadena *et al*., 2015; Durrant *et al*., 2006) |
| DQ241530 | 4,86 | -58,93 | *Plasmodium* sp. | *Volatinia jacarina* | Guyana | NA | 23 | 55 | AMN | (Durrant *et al*., 2006) |
| DQ241530 | 4,86 | -58,93 | *Plasmodium* sp. | *Cacicus cela* | Guyana | NA | 23 | 55 | AMN | (Durrant *et al*., 2006) |
| DQ241530 | 4,86 | -58,93 | *Plasmodium* sp. | *Cacicus haemorrhous* | Guyana | NA | 23 | 55 | AMN | (Durrant *et al*., 2006) |
| DQ241530 | 4,86 | -58,93 | *Plasmodium* sp. | *Diopsittaca nobilis* | Guyana | NA | 23 | 55 | AMN | (Durrant *et al*., 2006) |
| DQ241530 | -0,0013 | -78,355 | *Plasmodium* sp. | *Zonotrichia capensis* | Ecuador | Quito | B23 | 55 | NAN | (Cadena *et al*., 2015) |
| JX021474 | -19,82 | -40,276 | *Plasmodium* sp. | *Zonotrichia capensis* | Brazil | Aracruz | DENPET03 | 55 | ATL | (Lacorte *et al*., 2013) |
| JX021474 | -17,111 | -43,82 | *Plasmodium* sp. | *Formicivora melanogaster* | Brazil | Bocaiúva | DENPET03 | 55 | CSA | (Lacorte *et al*., 2013) |
| JX021474 | -17,0003 | -46,008 | *Plasmodium* sp. | *Myiothlypis flaveola* | Brazil | Brasilandia de Minas | DENPET03 | 55 | CSA | (Lacorte *et al*., 2013) |
| JX021474 | -17,0003 | -46,008 | *Plasmodium* sp. | *Coereba flaveola* | Brazil | Brasilandia de Minas | DENPET03 | 55 | CSA | (Lacorte *et al*., 2013) |
| JX021474 | -17,0003 | -46,008 | *Plasmodium* sp. | *Setophaga pitiayumi* | Brazil | Brasilandia de Minas | DENPET03 | 55 | CSA | (Lacorte *et al*., 2013) |
| JX021474 | -17,0003 | -46,008 | *Plasmodium* sp. | *Turdus leucomelas* | Brazil | Brasilandia de Minas | DENPET03 | 55 | CSA | (Lacorte *et al*., 2013) |
| JX021474 | -19,789 | -42,141 | *Plasmodium* sp. | *Trichothraupis melanops* | Brazil | Caratinga | DENPET03 | 55 | CSA | (Lacorte *et al*., 2013) |
| JX021474 | -18,713 | -44,925 | *Plasmodium* sp. | *Myiothlypis flaveola* | Brazil | Felixlândia | DENPET03 | 55 | CSA | (Lacorte *et al*., 2013) |
| JX021474 | -18,713 | -44,925 | *Plasmodium* sp. | *Coereba flaveola* | Brazil | Felixlândia | DENPET03 | 55 | CSA | (Lacorte *et al*., 2013) |
| JX021474 | -18,713 | -44,925 | *Plasmodium* sp. | *Pachyramphus polychopterus* | Brazil | Felixlândia | DENPET03 | 55 | CSA | (Lacorte *et al*., 2013) |
| JX021474 | -22,959 | -44,041 | *Plasmodium* sp. | *Cantorchilus longirostris* | Brazil | Manga | DENPET03 | 55 | ATL | (Lacorte *et al*., 2013) |
| JX021474 | -16,11 | -40,022 | *Plasmodium* sp. | *Turdus rufiventris* | Brazil | Salto da Divisa | DENPET03 | 55 | CSA | (Lacorte *et al*., 2013) |
| JX021474 | -0,0013 | -78,355 | *Plasmodium* sp. | *Zonotrichia capensis* | Ecuador | Quito | DENPET03 | 55 | NAN | (Cadena *et al*., 2015) |
| JX021476 | -17,111 | -43,82 | *Plasmodium* sp. | *Basileuterus culicivorus* | Brazil | Bocaiúva | BAFLA03 | 55 | CSA | (Lacorte *et al*., 2013) |
| JX021476 | -17,111 | -43,82 | *Plasmodium* sp. | *Myiothlypis flaveola* | Brazil | Bocaiúva | BAFLA03 | 55 | CSA | (Lacorte *et al*., 2013) |
| JX021476 | -17,0003 | -46,008 | *Plasmodium* sp. | *Celeus flavescens* | Brazil | Brasilandia de Minas | BAFLA03 | 55 | CSA | (Lacorte *et al*., 2013) |
| JX021476 | -17,0003 | -46,008 | *Plasmodium* sp. | *Dryocopus lineatus* | Brazil | Brasilandia de Minas | BAFLA03 | 55 | CSA | (Lacorte *et al*., 2013) |
| JX021476 | -19,789 | -42,141 | *Plasmodium* sp. | *Hylophilus amaurocephalus* | Brazil | Caratinga | BAFLA03 | 55 | CSA | (Lacorte *et al*., 2013) |
| JX021476 | -19,789 | -42,141 | *Plasmodium* sp. | *Leptopogon amaurocephalus* | Brazil | Caratinga | BAFLA03 | 55 | CSA | (Lacorte *et al*., 2013) |
| JX021476 | -19,789 | -42,141 | *Plasmodium* sp. | *Trichothraupis melanops* | Brazil | Caratinga | BAFLA03 | 55 | CSA | (Lacorte *et al*., 2013) |
| JX021476 | -19,789 | -42,141 | *Plasmodium* sp. | *Turdus rufiventris* | Brazil | Caratinga | BAFLA03 | 55 | CSA | (Lacorte *et al*., 2013) |
| JX021476 | -16,437 | -41,012 | *Plasmodium* sp. | *Myiothlypis flaveola* | Brazil | Jequitinhonha | BAFLA03 | 55 | CSA | (Lacorte *et al*., 2013) |
| JX021476 | -22,959 | -44,041 | *Plasmodium* sp. | *Hylophilus amaurocephalus* | Brazil | Manga | BAFLA03 | 55 | ATL | (Lacorte *et al*., 2013) |
| JX021476 | -22,959 | -44,041 | *Plasmodium* sp. | *Myiarchus tyrannulus* | Brazil | Manga | BAFLA03 | 55 | ATL | (Lacorte *et al*., 2013) |
| JX021476 | -16,11 | -40,022 | *Plasmodium* sp. | *Euphonia violacea* | Brazil | Salto da Divisa | BAFLA03 | 55 | CSA | (Lacorte *et al*., 2013) |
| JX029888 | -17,0003 | -46,008 | *Plasmodium* sp. | *Pachyramphus polychopterus* | Brazil | Brasilandia de Minas | PAPOL06 | 55 | CSA | (Lacorte *et al*., 2013) |
| KC867669 | 43,193 | -71,572 | *Plasmodium* sp. | *Dolichonyx oryzivorus* | United States of America | New Hampshire - Platte River | VOLJAC02 | 55 | NA | (Levin *et al*., 2013) |
| KC867671 | 43,193 | -71,572 | *Plasmodium* sp. | *Dolichonyx oryzivorus* | United States of America | New Hampshire - Platte River | DENPET03 | 55 | NA | (Levin *et al*., 2013) |
| KJ469132 | -1,817 | -65,7 | *Plasmodium* sp. | *Rynchops niger* | Brazil | Amazonas | NA | 55 | AMN | (Roos *et al*. 2015) |
| KU562434 | -4,683 | -56,63 | *Plasmodium* sp. | *Schiffornis turdina* | Brazil | Tapajόs River | NA | 55 | AMS | (Fecchio *et al*., 2017) |
| KU562435 | -4,683 | -56,63 | *Plasmodium* sp. | *Arremon taciturnus* | Brazil | Tapajόs River | NA | 55 | AMS | (Fecchio *et al*., 2017) |
| KU562436 | -4,683 | -56,63 | *Plasmodium* sp. | *Arremon taciturnus* | Brazil | Tapajόs River | NA | 55 | AMS | (Fecchio *et al*., 2017) |
| KU562437 | -5,717 | -63,2 | *Plasmodium* sp. | *Megastictus margaritatus* | Brazil | Purus River | NA | 55 | AMN | (Fecchio *et al*., 2017) |
| KU562438 | -9,33 | -64,683 | *Plasmodium* sp. | *Arremon taciturnus* | Brazil | Madeira River | NA | 55 | AMS | (Fecchio *et al*., 2017) |
| KU562446 | -4,683 | -56,63 | *Plasmodium* sp. | *Tachyphonus cristatus* | Brazil | Tapajόs River | NA | 55 | AMS | (Fecchio *et al*., 2017) |
| KU562447 | -12,567 | -70,083 | *Plasmodium* sp. | *Dendrocincla merula* | Peru | Manu | NA | 55 | CAN | (Fecchio *et al*., 2017) |
| KU562448 | -12,567 | -70,083 | *Plasmodium* sp. | *Leptopogon amaurocephalus* | Peru | Manu | NA | 55 | CAN | (Fecchio *et al*., 2017) |
| KU562464 | -4,5 | -56,267 | *Plasmodium nucleophilum* | *Ramphocelus carbo* | Brazil | Tapajόs River | NA | 55 | AMS | (Fecchio *et al*., 2017) |
| KU562465 | -4,5 | -56,267 | *Plasmodium nucleophilum* | *Thamnophilus nigrocinereus* | Brazil | Tapajόs River | NA | 55 | AMS | (Fecchio *et al*., 2017) |
| KU562466 | -15,53 | -47,55 | *Plasmodium nucleophilum* | *Hemithraupis guira* | Brazil | Planaltina | NA | 55 | CSA | (Fecchio *et al*., 2017) |
| KU562467 | -15,53 | -47,55 | *Plasmodium nucleophilum* | *Cypsnagra hirundinacea* | Brazil | Planaltina | NA | 55 | CSA | (Fecchio *et al*., 2017) |
| KU562468 | -15,53 | -47,55 | *Plasmodium nucleophilum* | *Cypsnagra hirundinacea* | Brazil | Planaltina | NA | 55 | CSA | (Fecchio *et al*., 2017) |
| KU562469 | -15,53 | -47,55 | *Plasmodium nucleophilum* | *Cypsnagra hirundinacea* | Brazil | Planaltina | NA | 55 | CSA | (Fecchio *et al*., 2017) |
| KU562470 | -15,53 | -47,55 | *Plasmodium nucleophilum* | *Mimus saturninus* | Brazil | Planaltina | NA | 55 | CSA | (Fecchio *et al*., 2017) |
| KU562471 | -5,717 | -63,2 | *Plasmodium nucleophilum* | *Ramphocelus carbo* | Brazil | Purus River | NA | 55 | AMN | (Fecchio *et al*., 2017) |
| KU562472 | -5,923 | -35,175 | *Plasmodium nucleophilum* | *Cacicus solitarius* | Brazil | Ponta Negra | NA | 55 | ATL | (Fecchio *et al*., 2017) |
| KU562473 | -19,567 | -57,017 | *Plasmodium nucleophilum* | *Ramphocelus carbo* | Brazil | Corumbá | NA | 55 | CSA | (Fecchio *et al*., 2017) |
| KU562474 | -19,567 | -57,017 | *Plasmodium nucleophilum* | *Ramphocelus carbo* | Brazil | Corumbá | NA | 55 | CSA | (Fecchio *et al*., 2017) |
| KU562475 | -19,567 | -57,017 | *Plasmodium nucleophilum* | *Saltator coerulescens* | Brazil | Corumbá | NA | 55 | CSA | (Fecchio *et al*., 2017) |
| KU562476 | -19,567 | -57,017 | *Plasmodium nucleophilum* | *Ramphocelus carbo* | Brazil | Corumbá | NA | 55 | CSA | (Fecchio *et al*., 2017) |
| KU562477 | -12,567 | -70,083 | *Plasmodium nucleophilum* | *Arremon taciturnus* | Peru | Manu | NA | 55 | CAN | (Fecchio *et al*., 2017) |
| KU562478 | -12,567 | -70,083 | *Plasmodium nucleophilum* | *Hypocnemis subflava* | Peru | Manu | NA | 55 | CAN | (Fecchio *et al*., 2017) |
| KU562479 | -12,567 | -70,083 | *Plasmodium nucleophilum* | *Automolus rufipileatus* | Peru | Manu | NA | 55 | CAN | (Fecchio *et al*., 2017) |
| KU562480 | -12,567 | -70,083 | *Plasmodium nucleophilum* | *Pipra fasciicauda* | Peru | Manu | NA | 55 | CAN | (Fecchio *et al*., 2017) |
| KU562481 | -12,567 | -70,083 | *Plasmodium nucleophilum* | *Turdus hauxwelli* | Peru | Manu | NA | 55 | CAN | (Fecchio *et al*., 2017) |
| KU562482 | -12,567 | -70,083 | *Plasmodium nucleophilum* | *Pipra fasciicauda* | Peru | Manu | NA | 55 | CAN | (Fecchio *et al*., 2017) |
| KU562483 | -12,567 | -70,083 | *Plasmodium nucleophilum* | *Cyphorhinus arada* | Peru | Manu | NA | 55 | CAN | (Fecchio *et al*., 2017) |
| KU562484 | -12,567 | -70,083 | *Plasmodium nucleophilum* | *Cyphorhinus arada* | Peru | Manu | NA | 55 | CAN | (Fecchio *et al*., 2017) |
| KU562485 | -12,567 | -70,083 | *Plasmodium nucleophilum* | *Xiphorhynchus ocellatus* | Peru | Manu | NA | 55 | CAN | (Fecchio *et al*., 2017) |
| KU562486 | -12,567 | -70,083 | *Plasmodium nucleophilum* | *Pipra fasciicauda* | Peru | Manu | NA | 55 | CAN | (Fecchio *et al*., 2017) |
| KU562487 | -12,567 | -70,083 | *Plasmodium nucleophilum* | *Ramphocelus carbo* | Peru | Manu | NA | 55 | CAN | (Fecchio *et al*., 2017) |
| KU562488 | -12,567 | -70,083 | *Plasmodium nucleophilum* | *Turdus hauxwelli* | Peru | Manu | NA | 55 | CAN | (Fecchio *et al*., 2017) |
| KU562489 | -12,567 | -70,083 | *Plasmodium nucleophilum* | *Arremon taciturnus* | Peru | Manu | NA | 55 | CAN | (Fecchio *et al*., 2017) |
| KU562490 | -12,567 | -70,083 | *Plasmodium nucleophilum* | *Ramphocelus carbo* | Peru | Manu | NA | 55 | CAN | (Fecchio *et al*., 2017) |
| KU562491 | -12,567 | -70,083 | *Plasmodium nucleophilum* | *Ramphocelus carbo* | Peru | Manu | NA | 55 | CAN | (Fecchio *et al*., 2017) |
| KU562553 | -15,53 | -47,55 | *Plasmodium* sp. | *Nystalus chacuru* | Brazil | Planaltina | NA | 55 | CSA | (Fecchio *et al*., 2017) |
| KU562603 | -0,583 | -64,917 | *Plasmodium* sp. | *Automolus infuscatus* | Brazil | Negro River | NA | 55 | AMN | (Fecchio *et al*., 2017) |
| KU562679 | -5,717 | -63,2 | *Plasmodium* sp. | *Ramphocelus carbo* | Brazil | Purus River | NA | 55 | AMN | (Fecchio *et al*., 2017) |
| KU562689 | -19,567 | -57,017 | *Plasmodium* sp. | *Paroaria capitata* | Brazil | Corumbá | NA | 55 | CSA | (Fecchio *et al*., 2017) |
| KU562690 | -19,567 | -57,017 | *Plasmodium* sp. | *Paroaria capitata* | Brazil | Corumbá | NA | 55 | CSA | (Fecchio *et al*., 2017) |
| KU562699 | -6,582 | -37,267 | *Plasmodium* sp. | *Zonotrichia capensis* | Brazil | Serra Negra do Norte | NA | 55 | ATL | (Fecchio *et al*., 2017) |
| KU562700 | -6,582 | -37,267 | *Plasmodium* sp. | *Zonotrichia capensis* | Brazil | Serra Negra do Norte | NA | 55 | ATL | (Fecchio *et al*., 2017) |
| KU562734 | -3,7 | -46,75 | *Plasmodium* sp. | *Psarocolius bifasciatus* | Brazil | Gurupi | NA | 55 | AMS | (Fecchio *et al*., 2017) |
| KU562811 | -12,567 | -70,083 | *Plasmodium* sp. | *Pheugopedius genibarbis* | Peru | Manu | NA | 55 | CAN | (Fecchio *et al*., 2017) |
| KU562820 | -12,567 | -70,083 | *Plasmodium* sp. | *Myrmoborus myotherinus* | Peru | Manu | NA | 55 | CAN | (Fecchio *et al*., 2017) |
| KU562834 | -12,567 | -70,083 | *Plasmodium* sp. | *Hemitriccus griseipectus* | Peru | Manu | NA | 55 | CAN | (Fecchio *et al*., 2017) |
| KC867670 | 44,451 | -73,122 | *Plasmodium* sp. | *Dolichonyx oryzivorus* | United States of America | Vermont - northwestern Vermont | DENPET03 | 55 | NA | (Levin *et al*., 2013) |
| JF833043 | -0,829 | -90,982 | *Haemoproteus* sp. | *Fregata magnificens* | Ecuador | Galápagos Islands | CY19 | 56 | NAN | (Levin *et al*., 2011) |
| JF833043 | -0,829 | -90,982 | *Haemoproteus* sp. | *Fregata minor* | Ecuador | Galápagos Islands | CY19 | 56 | NAN | (Levin *et al*., 2011) |
| JF833043 | 19,313 | -81,255 | *Haemoproteus* sp. | *Fregata magnificens* | Cayman Islands | NA | CY19 | 56 | GAN | (Levin *et al*., 2011) |
| JF833043 | 17,189 | -88,497 | *Haemoproteus* sp. | *Fregata magnificens* | Belize | NA | CY19 | 56 | GCS | (Levin *et al*., 2011) |
| JF833043 | 8,537 | -80,782 | *Haemoproteus* sp. | *Fregata magnificens* | Panama | NA | CY19 | 56 | CDH | (Levin *et al*., 2011) |
| JF833043 | 19,896 | -155,582 | *Haemoproteus* sp. | *Fregata minor* | United States of America | Hawaii | CY19 | 56 | NA | (Levin *et al*., 2011) |
| JF833044 | -0,829 | -90,982 | *Haemoproteus* sp. | *Fregata minor* | Ecuador | Galápagos Islands | E75 | 56 | NAN | (Levin *et al*., 2011) |
| JF833044 | -0,829 | -90,982 | *Haemoproteus* sp. | *Fregata magnificens* | Ecuador | Galápagos Islands | E75 | 56 | NAN | (Levin *et al*., 2011) |
| JF833045 | -0,829 | -90,982 | *Haemoproteus* sp. | *Fregata minor* | Ecuador | Galápagos Islands | FMAG15 | 56 | NAN | (Levin *et al*., 2011) |
| JF833045 | -0,829 | -90,982 | *Haemoproteus* sp. | *Fregata magnificens* | Ecuador | Galápagos Islands | FMAG15 | 56 | NAN | (Levin *et al*., 2011) |
| JF833048 | -0,829 | -90,982 | *Haemoproteus* sp. | *Fregata minor* | Ecuador | Galápagos Islands | HC02 | 56 | NAN | (Levin *et al*., 2011) |
| JF833048 | -0,829 | -90,982 | *Haemoproteus* sp. | *Fregata magnificens* | Ecuador | Galápagos Islands | HC02 | 56 | NAN | (Levin *et al*., 2011) |
| JF833049 | -0,829 | -90,982 | *Haemoproteus* sp. | *Fregata magnificens* | Ecuador | Galápagos Islands | HIPP5 | 56 | NAN | (Levin *et al*., 2011) |
| JF833050 | -0,829 | -90,982 | *Haemoproteus iwa* | *Fregata minor* | Ecuador | Galápagos Islands | FMINGAL1 | 56 | NAN | (Levin *et al*., 2011) |
| JF833050 | -0,829 | -90,982 | *Haemoproteus iwa* | *Fregata magnificens* | Ecuador | Galápagos Islands | FMINGAL1 | 56 | NAN | (Levin *et al*., 2011) |
| JF833058 | 8,537 | -80,782 | *Haemoproteus* sp. | *Fregata magnificens* | Panama | NA | GI20 | 56 | CDH | (Levin *et al*., 2011) |
| JF833049 | -0,829 | -90,982 | *Haemoproteus* sp. | *Fregata minor* | Ecuador | Galápagos Islands | HIPP5 | 56 | NAN | (Levin *et al*., 2011) |
| JN792139 | 36,778 | -119,418 | *Haemoproteus* sp. | *Catharus ustulatus* | United States of America | California | NA | 57 | NA | (Galen and Witt, 2014) |
| JN792141 | 36,778 | -119,418 | *Haemoproteus* sp. | *Catharus ustulatus* | United States of America | California | NA | 57 | NA | (Galen and Witt, 2014) |
| JX029873 | -17,0003 | -46,008 | *Plasmodium* sp. | *Antilophia galeata* | Brazil | Brasilandia de Minas | ANGAL01 | 58 | CSA | (Lacorte *et al*., 2013) |
| KF482355 | -9,190 | -75,015 | *Plasmodium* sp. | *Lepidothrix coronata* | Peru | NA | NA | 58 | NAN | (Marzal, Sehgal & Cardenas, 2013. Sin publicar) |
| KF482355 | -3,944 | -73,607 | *Plasmodium* sp. | *Cacicus cela* | Peru | Allpahuayo Mishana National Reserve | NA | 58 | AMN | (Ricopa & Villa, 2016) |
| KF482356 | -12,217 | -76,985 | *Plasmodium* sp. | *Tachuris rubrigastra* | Peru | Pantanos de Villa wetland Reserve | TACHURIS01 | 58 | STP | (Marzal *et al*., 2015) |
| KT373870 | -2,087 | -77,751 | *Plasmodium* sp. | *Ceratopipra erythrocephala* | Ecuador | Wisui reserve | NA | 58 | AMN | (Moens *et al*., 2017) |
| KT373871 | -2,117 | -77,733 | *Plasmodium* sp. | *Lepidothrix coronata* | Ecuador | Morona-Santiago Province, Wisui | LEPCOR01 | 58 | AMN | (Moens & Pérez-Tris, 2016) |
| KT373871 | -2,087 | -77,751 | *Plasmodium* sp. | *Lepidothrix coronata* | Ecuador | Wisui reserve | NA | 58 | AMN | (Moens *et al*., 2017) |
| KU236431 | -3,683 | -60,313 | *Plasmodium* sp. | *Lepidothrix coronata* | Brazil | Careiro-Castanho | LECOR01 | 58 | AMN | (Bosholn *et al*., 2016) |
| KU562628 | -4,983 | -62,13 | *Plasmodium* sp. | *Lepidothrix coronata* | Brazil | Purus River | NA | 58 | AMN | (Fecchio *et al*., 2017) |
| KU562629 | -5,717 | -63,2 | *Plasmodium* sp. | *Lepidothrix coronata* | Brazil | Purus River | NA | 58 | AMN | (Fecchio *et al*., 2017) |
| KU562630 | -5,717 | -63,2 | *Plasmodium* sp. | *Lepidothrix coronata* | Brazil | Purus River | NA | 58 | AMN | (Fecchio *et al*., 2017) |
| KU562631 | -5,717 | -63,2 | *Plasmodium* sp. | *Lepidothrix coronata* | Brazil | Purus River | NA | 58 | AMN | (Fecchio *et al*., 2017) |
| KU562632 | -5,717 | -63,2 | *Plasmodium* sp. | *Lepidothrix coronata* | Brazil | Purus River | NA | 58 | AMN | (Fecchio *et al*., 2017) |
| KU562633 | -12,217 | -60,73 | *Plasmodium* sp. | *Lepidothrix nattereri* | Brazil | Chupinguaia | NA | 58 | AMS | (Fecchio *et al*., 2017) |
| KU562634 | -12,217 | -60,73 | *Plasmodium* sp. | *Lepidothrix nattereri* | Brazil | Chupinguaia | NA | 58 | AMS | (Fecchio *et al*., 2017) |
| KU562635 | -12,217 | -60,73 | *Plasmodium* sp. | *Lepidothrix nattereri* | Brazil | Chupinguaia | NA | 58 | AMS | (Fecchio *et al*., 2017) |
| KU562636 | -12,217 | -60,73 | *Plasmodium* sp. | *Lepidothrix nattereri* | Brazil | Chupinguaia | NA | 58 | AMS | (Fecchio *et al*., 2017) |
| KU562637 | -12,217 | -60,73 | *Plasmodium* sp. | *Lepidothrix nattereri* | Brazil | Chupinguaia | NA | 58 | AMS | (Fecchio *et al*., 2017) |
| KU562638 | -12,217 | -60,73 | *Plasmodium* sp. | *Lepidothrix nattereri* | Brazil | Chupinguaia | NA | 58 | AMS | (Fecchio *et al*., 2017) |
| KU562639 | -13,8 | -59,683 | *Plasmodium* sp. | *Lepidothrix nattereri* | Brazil | Comodoro | NA | 58 | CSA | (Fecchio *et al*., 2017) |
| KU562640 | -12,217 | -60,73 | *Plasmodium* sp. | *Lepidothrix nattereri* | Brazil | Chupinguaia | NA | 58 | AMS | (Fecchio *et al*., 2017) |
| KU562641 | -13,8 | -59,683 | *Plasmodium* sp. | *Lepidothrix nattereri* | Brazil | Comodoro | NA | 58 | CSA | (Fecchio *et al*., 2017) |
| KU562642 | -9,3 | -64,717 | *Plasmodium* sp. | *Ceratopipra rubrocapilla* | Brazil | Porto Velho | NA | 58 | AMS | (Fecchio *et al*., 2017) |
| KU562643 | -9,33 | -64,683 | *Plasmodium* sp. | *Ceratopipra rubrocapilla* | Brazil | Madeira River | NA | 58 | AMS | (Fecchio *et al*., 2017) |
| KU562824 | -12,567 | -70,083 | *Plasmodium* sp. | *Cyphorhinus arada* | Peru | Manu | NA | 58 | CAN | (Fecchio *et al*., 2017) |
| KU562828 | -12,567 | -70,083 | *Plasmodium* sp. | *Dendrocincla fuliginosa* | Peru | Manu | NA | 58 | CAN | (Fecchio *et al*., 2017) |
| KU562829 | -12,567 | -70,083 | *Plasmodium* sp. | *Corythopis torquatus* | Peru | Manu | NA | 58 | CAN | (Fecchio *et al*., 2017) |
| KT373870 | -2,117 | -77,733 | *Plasmodium* sp. | *Ceratopipra erythrocephala* | Ecuador | Morona-Santiago Province, Wisui | TACRUB01 | 58 | AMN | (Moens & Pérez-Tris, 2016) |
| KT373869 | -2,087 | -77,751 | *Plasmodium* sp. | *Gymnopithys leucaspis* | Ecuador | Wisui reserve | NA | 59 | AMN | (Moens *et al*., 2017) |
| KT373869 | -2,117 | -77,733 | *Plasmodium* sp. | *Gymnopithys leucaspis* | Ecuador | Morona-Santiago Province, Wisui | GYMLEU01 | 59 | AMN | (Moens & Pérez-Tris, 2016) |
| JX029883 | -19,789 | -42,141 | *Plasmodium* sp. | *Myiopagis caniceps* | Brazil | Caratinga | MYCAN01 | 60 | CSA | (Lacorte *et al*., 2013) |
| KT373874 | -2,087 | -77,751 | *Plasmodium* sp. | *Lepidothrix coronata* | Ecuador | Wisui reserve | NA | 60 | AMN | (Moens *et al*., 2017) |
| KU236433 | -3,685 | -60,315 | *Plasmodium* sp. | *Lepidothrix coronata* | Brazil | Careiro-Castanho | LECOR03 | 60 | AMN | (Bosholn *et al*., 2016) |
| KU562376 | -5,217 | -56,917 | *Plasmodium* sp. | *Leptopogon amaurocephalus* | Brazil | Tapajόs River | NA | 60 | AMS | (Fecchio *et al*., 2017) |
| KU562581 | -0,4 | -64,8 | *Plasmodium* sp. | *Lepidothrix coronata* | Brazil | Negro River | NA | 60 | AMN | (Fecchio *et al*., 2017) |
| KU562582 | -0,4 | -64,8 | *Plasmodium* sp. | *Ceratopipra erythrocephala* | Brazil | Negro River | NA | 60 | AMN | (Fecchio *et al*., 2017) |
| KU562583 | -12,217 | -60,73 | *Plasmodium* sp. | *Lepidothrix nattereri* | Brazil | Chupinguaia | NA | 60 | AMS | (Fecchio *et al*., 2017) |
| KU562584 | -9,33 | -64,683 | *Plasmodium* sp. | *Lepidothrix nattereri* | Brazil | Madeira River | NA | 60 | AMS | (Fecchio *et al*., 2017) |
| KU562585 | -9,317 | -64,717 | *Plasmodium* sp. | *Pipra fasciicauda* | Brazil | Porto Velho | NA | 60 | AMS | (Fecchio *et al*., 2017) |
| KU562586 | -9,33 | -64,67 | *Plasmodium* sp. | *Thamnophilus aethiops* | Brazil | Porto Velho | NA | 60 | AMS | (Fecchio *et al*., 2017) |
| KU562587 | -12,567 | -70,083 | *Plasmodium* sp. | *Turdus hauxwelli* | Peru | Manu | NA | 60 | CAN | (Fecchio *et al*., 2017) |
| KU562588 | -12,567 | -70,083 | *Plasmodium* sp. | *Pipra fasciicauda* | Peru | Manu | NA | 60 | CAN | (Fecchio *et al*., 2017) |
| KU562792 | -9,33 | -64,7 | *Plasmodium* sp. | *Chloroceryle aenea* | Brazil | Porto Velho | NA | 60 | AMS | (Fecchio *et al*., 2017) |
| KU562805 | -9,133 | -64,5 | *Plasmodium* sp. | *Pipra fasciicauda* | Brazil | Porto Velho | NA | 60 | AMS | (Fecchio *et al*., 2017) |
| KU562806 | -9,3 | -64,717 | *Plasmodium* sp. | *Ceratopipra rubrocapilla* | Brazil | Porto Velho | NA | 60 | AMS | (Fecchio *et al*., 2017) |
| KU562810 | -9,283 | -64,73 | *Plasmodium* sp. | *Xiphorhynchus ocellatus perplexo* | Brazil | Porto Velho | NA | 60 | AMS | (Fecchio *et al*., 2017) |
| KU562827 | -12,567 | -70,083 | *Plasmodium* sp. | *Pipra fasciicauda* | Peru | Manu | NA | 60 | CAN | (Fecchio *et al*., 2017) |
| KT373874 | -2,117 | -77,733 | *Plasmodium* sp. | *Lepidothrix coronata* | Ecuador | Morona-Santiago Province, Wisui | LEPCOR04 | 60 | AMN | (Moens & Pérez-Tris, 2016) |
| JN819345 | 9,748 | -83,753 | *Haemoproteus* sp. | *Tangara icterocephala* | Costa Rica | NA | TASCH01 | 61 | CDH | (Galen and Witt, 2014) |
| JN819385 | 9,748 | -83,753 | *Haemoproteus* sp. | *Tangara icterocephala* | Costa Rica | NA | TASCH01 | 61 | CDH | (Galen and Witt, 2014) |
| JQ988150 | -6,104 | -78,341 | *Parahaemoproteus* sp. | *Tangara viridicollis* | Peru | Amazonas | NA | 61 | CAN | (Galen and Witt, 2014) |
| JQ988167 | -6,104 | -78,341 | *Parahaemoproteus* sp. | *Tangara nigroviridis* | Peru | Amazonas | NA | 61 | CAN | (Galen and Witt, 2014) |
| JQ988254 | -6,649 | -76,072 | *Parahaemoproteus* sp. | *Thalurania furcata* | Peru | San Martín | NA | 61 | CAN | (Galen and Witt, 2014) |
| JQ988745 | -14,06 | -73,008 | *Parahaemoproteus* sp. | *Xenodacnis parina* | Peru | Apurímac | NA | 61 | CAN | (Witt & McNew, 2012. Sin publicar) |
| JQ988745 | -3,944 | -73,607 | *Parahaemoproteus* sp. | *Myiozetetes granadensis* | Peru | Allpahuayo Mishana National Reserve | NA | 61 | AMN | (Ricopa & Villa, 2016) |
| KM211350 | 4,804 | -75,713 | *Haemoproteus coatneyi* | *Anisognathus somptuosus* | Colombia | Pereira | ANSOM01 | 61 | NAN | (González *et al*., 2015) |
| KT373861 | -2,117 | -77,733 | *Haemoproteus* sp. | *Tangara gyrola* | Ecuador | Morona-Santiago Province, Wisui | TANSCH01 | 61 | AMN | (Moens & Pérez-Tris, 2016) |
| KT373861 | -2,117 | -77,733 | *Haemoproteus* sp. | *Tangara chilensis* | Ecuador | Morona-Santiago Province, Wisui | TANSCH01 | 61 | AMN | (Moens & Pérez-Tris, 2016) |
| KT373861 | -2,087 | -77,751 | *Parahaemoproteus* sp. | *Tangara chilensis* | Ecuador | Wisui reserve | NA | 61 | AMN | (Moens *et al*., 2017) |
| KU364585 | -4,387 | -79,146 | *Haemoproteus witti* | *Eriocnemis vestita* | Ecuador | Podocarpus National Park | TROAED20 | 61 | NAN | (Moens *et al*., 2016) |
| KU364586 | -4,387 | -79,146 | *Haemoproteus witti* | *Tangara vassorii* | Ecuador | Podocarpus National Park | TROAED20 | 61 | NAN | (Moens *et al*., 2016) |
| KT373861 | -2,117 | -77,733 | *Haemoproteus* sp. | *Tangara schrankii* | Ecuador | Morona-Santiago Province, Wisui | TANSCH01 | 61 | AMN | (Moens & Pérez-Tris, 2016) |
| KT373858 | -2,117 | -77,733 | *Haemoproteus* sp. | *Gymnopithys leucaspis* | Ecuador | Morona-Santiago Province, Wisui | LEPCOR03 | 62 | AMN | (Moens & Pérez-Tris, 2016) |
| KT373858 | -2,117 | -77,733 | *Haemoproteus* sp. | *Hypocnemis cantator* | Ecuador | Morona-Santiago Province, Wisui | LEPCOR03 | 62 | AMN | (Moens & Pérez-Tris, 2016) |
| KT373858 | -2,117 | -77,733 | *Haemoproteus* sp. | *Chiroxiphia pareola* | Ecuador | Morona-Santiago Province, Wisui | LEPCOR03 | 62 | AMN | (Moens & Pérez-Tris, 2016) |
| KT373858 | -2,117 | -77,733 | *Haemoproteus* sp. | *Epinecrophylla erythrura* | Ecuador | Morona-Santiago Province, Wisui | LEPCOR03 | 62 | AMN | (Moens & Pérez-Tris, 2016) |
| KT373858 | -2,117 | -77,733 | *Haemoproteus* sp. | *Pithys albifrons* | Ecuador | Morona-Santiago Province, Wisui | LEPCOR03 | 62 | AMN | (Moens & Pérez-Tris, 2016) |
| KT373858 | -2,117 | -77,733 | *Haemoproteus* sp. | *Thamnomanes caesius* | Ecuador | Morona-Santiago Province, Wisui | LEPCOR03 | 62 | AMN | (Moens & Pérez-Tris, 2016) |
| KT373858 | -2,087 | -77,751 | *Haemoproteus* sp. | *Lepidothrix coronata* | Ecuador | Wisui reserve | NA | 62 | AMN | (Moens *et al*., 2017) |
| KU562237 | -13,8 | -59,683 | *Haemoproteus* sp. | *Ceratopipra rubrocapilla* | Brazil | Comodoro | NA | 62 | CSA | (Fecchio *et al*., 2017) |
| KU562238 | -13,8 | -59,683 | *Haemoproteus* sp. | *Elaenia parvirostris* | Brazil | Comodoro | NA | 62 | CSA | (Fecchio *et al*., 2017) |
| KU562239 | -13,8 | -59,683 | *Haemoproteus* sp. | *Elaenia parvirostris* | Brazil | Comodoro | NA | 62 | CSA | (Fecchio *et al*., 2017) |
| KU562240 | -13,8 | -59,683 | *Haemoproteus* sp. | *Machaeropterus pyrocephalus* | Brazil | Comodoro | NA | 62 | CSA | (Fecchio *et al*., 2017) |
| KT373858 | -2,117 | -77,733 | *Haemoproteus* sp. | *Lepidothrix coronata* | Ecuador | Morona-Santiago Province, Wisui | LEPCOR03 | 62 | AMN | (Moens & Pérez-Tris, 2016) |
| KT373864 | -2,087 | -77,751 | *Parahaemoproteus* sp. | *Malacoptila fusca* | Ecuador | Wisui reserve | NA | 63 | AMN | (Moens *et al*., 2017) |
| KU562151 | -4,683 | -56,63 | *Haemoproteus* sp. | *Dendrocolaptes certhia* | Brazil | Tapajόs River | NA | 63 | AMS | (Fecchio *et al*., 2017) |
| KU562152 | -4,5 | -56,283 | *Haemoproteus* sp. | *Hypocnemis striata* | Brazil | Tapajόs River | NA | 63 | AMS | (Fecchio *et al*., 2017) |
| KU562153 | -4,5 | -56,283 | *Haemoproteus sp* | *Galbula cyanicollis* | Brazil | Tapajόs River | NA | 63 | AMS | (Fecchio *et al*., 2017) |
| KU562154 | -4,5 | -56,283 | *Haemoproteus* sp. | *Malacoptila rufa* | Brazil | Tapajόs River | NA | 63 | AMS | (Fecchio *et al*., 2017) |
| KU562155 | -4,5 | -56,283 | *Haemoproteus* sp. | *Galbula cyanicollis* | Brazil | Tapajόs River | NA | 63 | AMS | (Fecchio *et al*., 2017) |
| KU562156 | -4,5 | -56,283 | *Haemoproteus* sp. | *Saltator coerulescens* | Brazil | Tapajόs River | NA | 63 | AMS | (Fecchio *et al*., 2017) |
| KU562162 | -4,5 | -56,267 | *Haemoproteus* sp. | *Cantorchilus leucotis* | Brazil | Tapajόs River | NA | 63 | AMS | (Fecchio *et al*., 2017) |
| KU562165 | -4,5 | -56,267 | *Haemoproteus* sp. | *Knipolegus poecilocercus* | Brazil | Tapajόs River | NA | 63 | AMS | (Fecchio *et al*., 2017) |
| KT373864 | -2,117 | -77,733 | *Haemoproteus* sp. | *Malacoptila fusca* | Ecuador | Morona-Santiago Province, Wisui | MALFUS01 | 63 | AMN | (Moens & Pérez-Tris, 2016) |
| KT373875 | -2,087 | -77,751 | *Plasmodium* sp. | *Myrmoborus myotherinus* | Ecuador | Wisui reserve | NA | 64 | AMN | (Moens *et al*., 2017) |
| KT373875 | -2,117 | -77,733 | *Plasmodium* sp. | *Myrmoborus myotherinus* | Ecuador | Morona-Santiago Province, Wisui | MYRMYO02 | 64 | AMN | (Moens & Pérez-Tris, 2016) |
| JQ988105 | -13,249 | -72,169 | *Parahaemoproteus* sp. | *Ocreatus underwoodii* | Peru | Cusco | NA | 65 | CAN | (Galen and Witt, 2014) |
| JQ988147 | -6,104 | -78,341 | *Parahaemoproteus* sp. | *Coeligena coeligena* | Peru | Amazonas | NA | 65 | CAN | (Galen and Witt, 2014) |
| JQ988371 | -11,983 | -74,933 | *Parahaemoproteus* sp. | *Coeligena torquata* | Peru | Junín | NA | 65 | CAN | (Galen and Witt, 2014) |
| JQ988384 | -11,983 | -74,933 | *Parahaemoproteus* sp. | *Coeligena torquata* | Peru | Junín | NA | 65 | CAN | (Galen and Witt, 2014) |
| JQ988406 | -6,104 | -78,341 | *Parahaemoproteus* sp. | *Troglodytes aedon* | Peru | Amazonas | TROAED20 | 65 | CAN | (Galen and Witt, 2014) |
| JQ988426 | -12,029 | -76,65 | *Parahaemoproteus* sp. | *Patagona gigas* | Peru | Lima | NA | 65 | STP | (Galen and Witt, 2014) |
| JQ988430 | -12,029 | -76,65 | *Parahaemoproteus* sp. | *Oreotrochilus melanogaster* | Peru | Lima | NA | 65 | STP | (Galen and Witt, 2014) |
| JQ988447 | -11,983 | -74,933 | *Parahaemoproteus* sp. | *Metallura tyrianthina* | Peru | Junín | NA | 65 | CAN | (Galen and Witt, 2014) |
| JQ988487 | -11,627 | -76,434 | *Parahaemoproteus* sp. | *Troglodytes aedon* | Peru | Lima | TROAED20 | 65 | CAN | (Galen and Witt, 2014) |
| JQ988488 | -11,627 | -76,434 | *Parahaemoproteus* sp. | *Troglodytes aedon* | Peru | Lima | TROAED20 | 65 | CAN | (Galen and Witt, 2014) |
| JQ988521 | -9,916 | -76,233 | *Parahaemoproteus* sp. | *Turdus fuscater* | Peru | Huánuco | NA | 65 | CAN | (Galen and Witt, 2014) |
| JQ988538 | -13,249 | -72,169 | *Parahaemoproteus* sp. | *Troglodytes aedon* | Peru | Cusco | TROAED20 | 65 | CAN | (Galen and Witt, 2014) |
| JQ988563 | -13,249 | -72,169 | *Parahaemoproteus* sp. | *Pyriglena leuconota* | Peru | Cusco | NA | 65 | CAN | (Galen and Witt, 2014) |
| JQ988570 | -13,249 | -72,169 | *Parahaemoproteus* sp. | *Diglossa glauca* | Peru | Cusco | NA | 65 | CAN | (Galen and Witt, 2014) |
| JQ988744 | -14,173 | -73,323 | *Parahaemoproteus* sp. | *Synallaxis courseni* | Peru | Apurímac | NA | 65 | CAN | (Galen and Witt, 2014) |
| JX029915 | -17,0003 | -46,008 | *Haemoproteus* sp. | *Tolmomyias sulphurescens* | Brazil | Brasilandia de Minas | TOSUL01 | 65 | CSA | (Lacorte *et al*., 2013) |
| JX029915 | -18,713 | -44,925 | *Haemoproteus* sp. | *Tolmomyias sulphurescens* | Brazil | Felixlândia | TOSUL01 | 65 | CSA | (Lacorte *et al*., 2013; Galen & Witt, 2014) |
| KC121053 | 10,829 | -73,692 | *Haemoproteus witti* | *Vermivora peregrina* | Colombia | Sierra Nevada de Santa Marta, San Lorenzo ridge | NA | 65 | NAN | (Gonzalez-Quevedo, Rivera-Gutierrez & Pabón, 2016) |
| KC121053 | 10,829 | -73,692 | *Haemoproteus witti* | *Metallura tyrianthina* | Colombia | Sierra Nevada de Santa Marta, San Lorenzo ridge | NA | 65 | NAN | (Gonzalez-Quevedo, Rivera-Gutierrez & Pabón, 2016) |
| KF537304 | 4,804 | -75,713 | *Haemoproteus witti* | *Eriocnemis vestitus* | Colombia | Pereira | TROAED20 | 65 | NAN | (González *et al*., 2015) |
| KF767420 | -11,627 | -76,434 | *Haemoproteus* sp. | *Troglodytes aedon* | Peru | Lima | TROAED20 | 65 | CAN | (Galen and Witt, 2014) |
| KJ661301 | -2,099 | -78,135 | *Haemoproteus* sp. | *Phaethornis* sp. | Ecuador | Nueva Alianza | NA | 65 | NAN | (Harrigan *et al*., 2014) |
| KT373863 | -2,117 | -77,733 | *Haemoproteus* sp. | *Heliodoxa schreibersii* | Ecuador | Morona-Santiago Province, Wisui | HELSCH01 | 65 | AMN | (Moens & Pérez-Tris, 2016) |
| KT373863 | -2,087 | -77,751 | *Parahaemoproteus* sp. | *Heliodoxa schreibersii* | Ecuador | Wisui reserve | NA | 65 | AMN | (Moens *et al*., 2017) |
| KU364540 | -4,235 | -79,174 | *Haemoproteus witti* | *Adelomyia melanogenys* | Ecuador | Podocarpus National Park | TROAED20 | 65 | NAN | (Moens *et al*., 2016) |
| KU364541 | -4,258 | -79,217 | *Haemoproteus witti* | *Amazilia alticola* | Ecuador | Podocarpus National Park | TROAED20 | 65 | NAN | (Moens *et al*., 2016) |
| KU364542 | -4,235 | -79,174 | *Haemoproteus witti* | *Boissonneaua matthewsii* | Ecuador | Podocarpus National Park | TROAED20 | 65 | NAN | (Moens *et al*., 2016) |
| KU364543 | -4,235 | -79,174 | *Haemoproteus witti* | *Coeligena iris* | Ecuador | Podocarpus National Park | TROAED20 | 65 | NAN | (Moens *et al*., 2016) |
| KU364544 | -4,235 | -79,174 | *Haemoproteus witti* | *Coeligena torquata* | Ecuador | Podocarpus National Park | TROAED20 | 65 | NAN | (Moens *et al*., 2016) |
| KU364545 | -4,235 | -79,174 | *Haemoproteus witti* | *Colibri thalassinus* | Ecuador | Podocarpus National Park | TROAED20 | 65 | NAN | (Moens *et al*., 2016) |
| KU364546 | -4,235 | -79,174 | *Haemoproteus witti* | *Heliangelus viola* | Ecuador | Podocarpus National Park | TROAED20 | 65 | NAN | (Moens *et al*., 2016) |
| KU364547 | -4,235 | -79,174 | *Haemoproteus witti* | *Lafresnaya lafresnayi* | Ecuador | Podocarpus National Park | TROAED20 | 65 | NAN | (Moens *et al*., 2016) |
| KU364548 | -4,387 | -79,146 | *Haemoproteus witti* | *Metallura tyrianthina* | Ecuador | Podocarpus National Park | TROAED20 | 65 | NAN | (Moens *et al*., 2016) |
| KU364549 | -4,235 | -79,174 | *Haemoproteus witti* | *Phaethornis griseogularis* | Ecuador | Podocarpus National Park | TROAED20 | 65 | NAN | (Moens *et al*., 2016) |
| KU364550 | -4,386 | -79,122 | *Haemoproteus witti* | *Amblycercus holosericeus* | Ecuador | Podocarpus National Park | TROAED20 | 65 | NAN | (Moens *et al*., 2016) |
| KU364551 | -4,235 | -79,174 | *Haemoproteus witti* | *Arremon torquatus* | Ecuador | Podocarpus National Park | TROAED20 | 65 | NAN | (Moens *et al*., 2016) |
| KU364552 | -4,235 | -79,174 | *Haemoproteus witti* | *Basileuterus trifasciatus* | Ecuador | Podocarpus National Park | TROAED20 | 65 | NAN | (Moens *et al*., 2016) |
| KU364553 | -4,258 | -79,217 | *Haemoproteus witti* | *Campylorhynchus fasciatus* | Ecuador | Podocarpus National Park | TROAED20 | 65 | NAN | (Moens *et al*., 2016) |
| KU364554 | -4,235 | -79,174 | *Haemoproteus witti* | *Cinnycerthia unirufa* | Ecuador | Podocarpus National Park | TROAED20 | 65 | NAN | (Moens *et al*., 2016) |
| KU364555 | -4,235 | -79,174 | *Haemoproteus witti* | *Cranioleuca antisiensis* | Ecuador | Podocarpus National Park | TROAED20 | 65 | NAN | (Moens *et al*., 2016) |
| KU364556 | -4,387 | -79,146 | *Haemoproteus witti* | *Diglossa caerulescens* | Ecuador | Podocarpus National Park | TROAED20 | 65 | NAN | (Moens *et al*., 2016) |
| KU364557 | -4,235 | -79,174 | *Haemoproteus witti* | *Elaenia albiceps* | Ecuador | Podocarpus National Park | TROAED20 | 65 | NAN | (Moens *et al*., 2016) |
| KU364558 | -4,235 | -79,174 | *Haemoproteus witti* | *Elaenia pallatangae* | Ecuador | Podocarpus National Park | TROAED20 | 65 | NAN | (Moens *et al*., 2016) |
| KU364559 | -4,258 | -79,217 | *Haemoproteus witti* | *Furnarius leucopus* | Ecuador | Podocarpus National Park | TROAED20 | 65 | NAN | (Moens *et al*., 2016) |
| KU364560 | -4,386 | -79,122 | *Haemoproteus witti* | *Hellmayrea gularis* | Ecuador | Podocarpus National Park | TROAED20 | 65 | NAN | (Moens *et al*., 2016) |
| KU364561 | -4,258 | -79,217 | *Haemoproteus witti* | *Icterus mesomelas* | Ecuador | Podocarpus National Park | TROAED20 | 65 | NAN | (Moens *et al*., 2016) |
| KU364562 | -4,235 | -79,174 | *Haemoproteus witti* | *Lepidocolaptes lacrymiger* | Ecuador | Podocarpus National Park | TROAED20 | 65 | NAN | (Moens *et al*., 2016) |
| KU364563 | -4,235 | -79,174 | *Haemoproteus witti* | *Mionectes striaticollis* | Ecuador | Podocarpus National Park | TROAED20 | 65 | NAN | (Moens *et al*., 2016) |
| KU364564 | -4,235 | -79,174 | *Haemoproteus witti* | *Myadestes ralloides* | Ecuador | Podocarpus National Park | TROAED20 | 65 | NAN | (Moens *et al*., 2016) |
| KU364565 | -4,235 | -79,174 | *Haemoproteus witti* | *Myioborus miniatus* | Ecuador | Podocarpus National Park | TROAED20 | 65 | NAN | (Moens *et al*., 2016) |
| KU364566 | -4,235 | -79,174 | *Haemoproteus witti* | *Myiothlypis coronata* | Ecuador | Podocarpus National Park | TROAED20 | 65 | NAN | (Moens *et al*., 2016) |
| KU364567 | -4,258 | -79,217 | *Haemoproteus witti* | *Myiothlypis fraseri* | Ecuador | Podocarpus National Park | TROAED20 | 65 | NAN | (Moens *et al*., 2016) |
| KU364568 | -4,235 | -79,174 | *Haemoproteus witti* | *Myiothlypis nigrocristatus* | Ecuador | Podocarpus National Park | TROAED20 | 65 | NAN | (Moens *et al*., 2016) |
| KU364569 | -4,387 | -79,146 | *Haemoproteus witti* | *Ochthoeca rufipectoralis* | Ecuador | Podocarpus National Park | TROAED20 | 65 | NAN | (Moens *et al*., 2016) |
| KU364570 | -4,235 | -79,174 | *Haemoproteus witti* | *Pipreola riefferii* | Ecuador | Podocarpus National Park | TROAED20 | 65 | NAN | (Moens *et al*., 2016) |
| KU364571 | -4,235 | -79,174 | *Haemoproteus witti* | *Synallaxis azarae* | Ecuador | Podocarpus National Park | TROAED20 | 65 | NAN | (Moens *et al*., 2016) |
| KU364572 | -4,235 | -79,174 | *Haemoproteus witti* | *Thraupis cyanocephala* | Ecuador | Podocarpus National Park | TROAED20 | 65 | NAN | (Moens *et al*., 2016) |
| KU364573 | -4,258 | -79,217 | *Haemoproteus witti* | *Tiaris obscura* | Ecuador | Podocarpus National Park | TROAED20 | 65 | NAN | (Moens *et al*., 2016) |
| KU364574 | -4,235 | -79,174 | *Haemoproteus witti* | *Turdus nigriceps* | Ecuador | Podocarpus National Park | TROAED20 | 65 | NAN | (Moens *et al*., 2016) |
| KU364575 | -4,235 | -79,174 | *Haemoproteus witti* | *Turdus serranus* | Ecuador | Podocarpus National Park | TROAED20 | 65 | NAN | (Moens *et al*., 2016) |
| KX130087 | 10,829 | -73,692 | *Plasmodium* sp. | *Vermivora peregrina* | Colombia | Sierra Nevada de Santa Marta, San Lorenzo ridge | NA | 65 | NAN | (Gonzalez-Quevedo, Rivera-Gutierrez & Pabón, 2016) |
| JQ988492 | -13,249 | -72,169 | *Parahaemoproteus* sp. | *Pyrrhomyias cinnamomeus* | Peru | Cusco | NA | 65 | CAN | (Galen and Witt, 2014) |
| JQ988144 | -6,104 | -78,341 | *Parahaemoproteus* sp. | *Creurgops verticalis* | Peru | Amazonas | NA | 66 | CAN | (Galen and Witt, 2014) |
| JQ988323 | -13,249 | -72,169 | *Parahaemoproteus* sp. | *Coeligena violifer* | Peru | Cusco | NA | 66 | CAN | (Galen and Witt, 2014) |
| JQ988370 | -11,983 | -74,933 | *Parahaemoproteus* sp. | *Coeligena torquata* | Peru | Junín | NA | 66 | CAN | (Galen and Witt, 2014) |
| JQ988393 | -13,249 | -72,169 | *Parahaemoproteus* sp. | *Coeligena torquata* | Peru | Cusco | NA | 66 | CAN | (Galen and Witt, 2014) |
| JX021453 | -16,437 | -41,012 | *Plasmodium* sp. | *Conopophaga lineata* | Brazil | Jequitinhonha | BAFLA01 | 67 | CSA | (Lacorte *et al*., 2013) |
| JX021453 | -16,437 | -41,012 | *Plasmodium* sp. | *Myiothlypis flaveola* | Brazil | Jequitinhonha | BAFLA01 | 67 | CSA | (Lacorte *et al*., 2013) |
| JX021453 | -19,993 | -43,848 | *Plasmodium* sp. | *Myiothlypis flaveola* | Brazil | Nova Lima | BAFLA01 | 67 | CSA | (Lacorte *et al*., 2013) |
| JX021478 | -19,993 | -43,848 | *Plasmodium* sp. | *Ilicura militaris* | Brazil | Nova Lima | ILMIL01 | 67 | CSA | (Lacorte *et al*., 2013) |
| JX021478 | -19,993 | -43,848 | *Plasmodium* sp. | *Thamnophilus caerulescens* | Brazil | Nova Lima | ILMIL01 | 67 | CSA | (Lacorte *et al*., 2013) |
| JX021489 | -19,82 | -40,276 | *Plasmodium* sp. | *Thraupis sayaca* | Brazil | Aracruz | COLIN05 | 67 | ATL | (Lacorte *et al*., 2013) |
| JX021489 | -19,789 | -42,141 | *Plasmodium* sp. | *Conopophaga lineata* | Brazil | Caratinga | COLIN05 | 67 | CSA | (Lacorte *et al*., 2013) |
| JX021489 | -19,789 | -42,141 | *Plasmodium* sp. | *Trichothraupis melanops* | Brazil | Caratinga | COLIN05 | 67 | CSA | (Lacorte *et al*., 2013) |
| JX021489 | -16,437 | -41,012 | *Plasmodium* sp. | *Tolmomyias flaviventris* | Brazil | Jequitinhonha | COLIN05 | 67 | CSA | (Lacorte *et al*., 2013) |
| JX021489 | -22,959 | -44,041 | *Plasmodium* sp. | *Polioptila plumbea* | Brazil | Manga | COLIN05 | 67 | ATL | (Lacorte *et al*., 2013) |
| JX021489 | -19,993 | -43,848 | *Plasmodium* sp. | *Conopophaga lineata* | Brazil | Nova Lima | COLIN05 | 67 | CSA | (Lacorte *et al*., 2013) |
| JX021489 | -19,993 | -43,848 | *Plasmodium* sp. | *Leptopogon amaurocephalus* | Brazil | Nova Lima | COLIN05 | 67 | CSA | (Lacorte *et al*., 2013) |
| JX021489 | -19,993 | -43,848 | *Plasmodium* sp. | *Philydor rufum* | Brazil | Nova Lima | COLIN05 | 67 | CSA | (Lacorte *et al*., 2013) |
| JX021451 | -19,993 | -43,848 | *Plasmodium* sp. | *Conopophaga lineata* | Brazil | Nova Lima | COLIN15 | 67 | CSA | (Lacorte *et al*., 2013) |
| JX021452 | -16,437 | -41,012 | *Plasmodium* sp. | *Conopophaga lineata* | Brazil | Jequitinhonha | COLIN16 | 68 | CSA | (Lacorte *et al*., 2013) |
| JX021452 | -19,993 | -43,848 | *Plasmodium* sp. | *Conopophaga lineata* | Brazil | Nova Lima | COLIN16 | 68 | CSA | (Lacorte *et al*., 2013) |
| JX021495 | -19,993 | -43,848 | *Plasmodium* sp. | *Conopophaga lineata* | Brazil | Nova Lima | COLIN12 | 68 | CSA | (Lacorte *et al*., 2013) |
| JX021452 | -16,437 | -41,012 | *Plasmodium* sp. | *Myiothlypis flaveola* | Brazil | Jequitinhonha | COLIN16 | 68 | CSA | (Lacorte *et al*., 2013) |
| JX029900 | -17,0003 | -46,008 | *Haemoproteus* sp. | *Dendrocolaptes platyrostris* | Brazil | Brasilandia de Minas | ELALB01 | 69 | CSA | (Lacorte *et al*., 2013) |
| JX029900 | 9,748 | -83,753 | *Haemoproteus* sp. | *Tangara icterocephala* | Costa Rica | NA | ELALB01 | 69 | CDH | (Galen and Witt, 2014) |
| JX029900 | -16,437 | -41,012 | *Haemoproteus* sp. | *Casiornis fuscus* | Brazil | Jequitinhonha | ELALB01 | 69 | CSA | (Lacorte *et al*., 2013) |
| JX029900 | -16,11 | -40,022 | *Haemoproteus* sp. | *Capsiempis flaveola* | Brazil | Salto da Divisa | ELALB01 | 69 | CSA | (Lacorte *et al*., 2013) |
| JX029900 | -16,11 | -40,022 | *Haemoproteus* sp. | *Casiornis fuscus* | Brazil | Salto da Divisa | ELALB01 | 69 | CSA | (Lacorte *et al*., 2013) |
| JX029900 | -16,11 | -40,022 | *Haemoproteus* sp. | *Myiodynastes maculatus* | Brazil | Salto da Divisa | ELALB01 | 69 | CSA | (Lacorte *et al*., 2013) |
| JX029900 | -16,11 | -40,022 | *Haemoproteus* sp. | *Pitangus sulphuratus* | Brazil | Salto da Divisa | ELALB01 | 69 | CSA | (Lacorte *et al*., 2013) |
| JX029900 | -16,11 | -40,022 | *Haemoproteus* sp. | *Tolmomyias flaviventris* | Brazil | Salto da Divisa | ELALB01 | 69 | CSA | (Lacorte *et al*., 2013) |
| JX029900 | -19,098 | -40,186 | *Haemoproteus* sp. | *Myiarchus tuberculifer* | Brazil | Sooretama | ELALB01 | 69 | ATL | (Lacorte *et al*., 2013) |
| JX029903 | -18,713 | -44,925 | *Haemoproteus* sp. | *Myiothlypis flaveola* | Brazil | Felixlândia | BAFLA02 | 69 | CSA | (Lacorte *et al*., 2013) |
| JX029903 | -16,11 | -40,022 | *Haemoproteus* sp. | *Tyrannus melancholicus* | Brazil | Salto da Divisa | BAFLA02 | 69 | CSA | (Lacorte *et al*., 2013) |
| JX029920 | -19,789 | -42,141 | *Haemoproteus* sp. | *Turdus rufiventris* | Brazil | Caratinga | TURUF02 | 69 | CSA | (Lacorte *et al*., 2013) |
| KU562174 | -15,53 | -47,55 | *Haemoproteus* sp. | *Myiarchus swainsoni* | Brazil | Planaltina | NA | 69 | CSA | (Fecchio *et al*., 2017) |
| KU562175 | -15,53 | -47,55 | *Haemoproteus* sp. | *Myiarchus swainsoni* | Brazil | Planaltina | NA | 69 | CSA | (Fecchio *et al*., 2017) |
| KU562176 | -15,53 | -47,55 | *Haemoproteus* sp. | *Phaeomyias murina* | Brazil | Planaltina | NA | 69 | CSA | (Fecchio *et al*., 2017) |
| KU562177 | -15,53 | -47,55 | *Haemoproteus* sp. | *Elaenia chiriquensis* | Brazil | Planaltina | NA | 69 | CSA | (Fecchio *et al*., 2017) |
| KU562178 | -15,53 | -47,55 | *Haemoproteus* sp. | *Elaenia chiriquensis* | Brazil | Planaltina | NA | 69 | CSA | (Fecchio *et al*., 2017) |
| KU562183 | -15,53 | -47,55 | *Haemoproteus* sp. | *Suiriri suiriri* | Brazil | Planaltina | NA | 69 | CSA | (Fecchio *et al*., 2017) |
| KU562184 | -15,53 | -47,55 | *Haemoproteus* sp. | *Suiriri suiriri* | Brazil | Planaltina | NA | 69 | CSA | (Fecchio *et al*., 2017) |
| KU562185 | -15,53 | -47,55 | *Haemoproteus* sp. | *Suiriri suiriri* | Brazil | Planaltina | NA | 69 | CSA | (Fecchio *et al*., 2017) |
| KU562186 | -15,53 | -47,55 | *Haemoproteus* sp. | *Suiriri suiriri* | Brazil | Planaltina | NA | 69 | CSA | (Fecchio *et al*., 2017) |
| KU562187 | -15,53 | -47,55 | *Haemoproteus* sp. | *Suiriri suiriri* | Brazil | Planaltina | NA | 69 | CSA | (Fecchio *et al*., 2017) |
| JQ988656 | -6,104 | -78,341 | *Parahaemoproteus* sp. | *Zimmerius viridiflavus* | Peru | Amazonas | NA | 69 | CAN | (Galen and Witt, 2014) |
| JX021455 | -16,11 | -40,022 | *Plasmodium* sp. | *Conopophaga melanops* | Brazil | Salto da Divisa | COMEL01 | 70 | CSA | (Lacorte *et al*., 2013) |
| JX021455 | -19,789 | -42,141 | *Plasmodium* sp. | *Conopophaga melanops* | Brazil | Caratinga | COMEL01 | 70 | CSA | (Lacorte *et al*., 2013) |
| MF077664 | 35,536 | -107,349 | *Haemoproteus* sp. | *Chondestes grammacus* | United States of America | Nuevo Mexico, Mesa Chivato | CHOGRA01 | 71 | NA | (Marroquin-Flores *et al*., 2017) |
| JX029917 | -19,82 | -40,276 | *Haemoproteus* sp. | *Elaenia flavogaster* | Brazil | Aracruz | ELFLA01 | 72 | ATL | (Lacorte *et al*., 2013) |
| JX029917 | -19,82 | -40,276 | *Haemoproteus* sp. | *Tyrannus melancholicus* | Brazil | Aracruz | ELFLA01 | 72 | ATL | (Lacorte *et al*., 2013) |
| MF077661 | 35,533 | -107,350 | *Haemoproteus* sp. | *Contopus sordidulus* | United States of America | Nuevo Mexico, Mesa Chivato | ELFLA01 | 72 | NA | (Marroquin-Flores *et al*., 2017) |
| JQ988575 | -13,249 | -72,169 | *Parahaemoproteus* sp. | *Mionectes striaticollis* | Peru | Cusco | NA | 73 | CAN | (Galen and Witt, 2014) |
| KJ661283 | -2,765 | -79,459 | *Haemoproteus* sp. | *Diglossa cyanea* | Ecuador | Sural | NA | 74 | NAN | (Harrigan *et al*., 2014) |
| KU562241 | -13,8 | -59,683 | *Haemoproteus* sp. | *Cyanerpes cyaneus* | Brazil | Comodoro | NA | 74 | CSA | (Fecchio *et al*., 2017) |
| JQ988571 | -13,249 | -72,169 | *Parahaemoproteus* sp. | *Tangara arthus* | Peru | Cusco | NA | 74 | CAN | (Galen and Witt, 2014) |
| DQ241534 | 4,86 | -58,93 | *Plasmodium* sp. | *Icterus nigrogularis* | Guyana | NA | 27 | 75 | AMN | (Durrant *et al*., 2006) |
| DQ241534 | 4,86 | -58,93 | *Plasmodium* sp. | *Sturnella militaris* | Guyana | NA | 27 | 75 | AMN | (Durrant *et al*., 2006) |
| DQ241553 | 4,86 | -58,93 | *Haemoproteus* sp. | *Eupsittula pertinax* | Guyana | NA | ARAPER01 | 75 | AMN | (Galen and Witt, 2014; Durrant *et al*., 2006) |
| DQ241554 | 4,86 | -58,93 | *Haemoproteus* sp. | *Paroaria gularis* | Guyana | NA | 47 | 75 | AMN | (Durrant *et al*., 2006) |
| KF767425 | -13,625 | -71,718 | *Haemoproteus* sp. | *Troglodytes aedon* | Peru | Cusco | TROAED19 | 75 | CAN | (Galen and Witt, 2014) |
| KX130084 | 10,829 | -73,692 | *Haemoproteus* sp. | *Atlapetes melanocephalus* | Colombia | Sierra Nevada de Santa Marta, San Lorenzo ridge | NA | 76 | NAN | (Gonzalez-Quevedo, Rivera-Gutierrez & Pabón, 2016) |
| KX130084 | 10,829 | -73,692 | *Haemoproteus* sp. | *Atlapetes melanocephalus* | Colombia | Sierra Nevada de Santa Marta, San Lorenzo ridge | NA | 76 | NAN | (Gonzalez-Quevedo, Rivera-Gutierrez & Pabón, 2016) |
| JQ988107 | -13,249 | -72,169 | *Parahaemoproteus* sp. | *Chlorospingus flavigularis* | Peru | Cusco | NA | 77 | CAN | (Galen and Witt, 2014) |
| JQ988136 | -6,104 | -78,341 | *Parahaemoproteus* sp. | *Drymophila caudata* | Peru | Amazonas | NA | 77 | CAN | (Galen and Witt, 2014) |
| JQ988134 | -6,104 | -78,341 | *Parahaemoproteus* sp. | *Zonotrichia capensis* | Peru | Amazonas | NA | 77 | CAN | (Galen and Witt, 2014) |
| JQ988489 | -13,249 | -72,169 | *Parahaemoproteus* sp. | *Diglossa sittoides* | Peru | Cusco | NA | 78 | CAN | (Galen and Witt, 2014) |
| JQ988117 | -5,896 | -79,785 | *Parahaemoproteus* sp. | *Diglossa humeralis* | Peru | Lambayeque | NA | 78 | CAN | (Galen and Witt, 2014) |
| JQ988123 | -5,896 | -79,785 | *Parahaemoproteus* sp. | *Arremon assimilis* | Peru | Lambayeque | NA | 79 | CAN | (Galen and Witt, 2014) |
| JQ988256 | -6,649 | -76,072 | *Parahaemoproteus* sp. | *Thalurania furcata* | Peru | San Martín | NA | 80 | CAN | (Galen and Witt, 2014) |
| MF077663 | 35,526 | -107,387 | *Haemoproteus* sp. | *Vireo gilvus* | United States of America | Nuevo Mexico, Mesa Chivato | VIGIL05 | 81 | NA | (Marroquin-Flores *et al*., 2017) |
| KF767419 | -12,651 | -72,323 | *Haemoproteus* sp. | *Troglodytes aedon* | Peru | Cusco | TROAED13 | 81 | CAN | (Galen and Witt, 2014) |
| JX021460 | -19,789 | -42,141 | *Plasmodium* sp. | *Turdus leucomelas* | Brazil | Caratinga | TULEU01 | 82 | CSA | (Lacorte *et al*., 2013) |
| JX021460 | -18,713 | -44,925 | *Plasmodium* sp. | *Turdus leucomelas* | Brazil | Felixlândia | TULEU01 | 82 | CSA | (Lacorte *et al*., 2013) |
| JX021460 | -18,713 | -44,925 | *Plasmodium* sp. | *Tyrannus melancholicus* | Brazil | Felixlândia | TULEU01 | 82 | CSA | (Lacorte *et al*., 2013) |
| JX021460 | -16,437 | -41,012 | *Plasmodium* sp. | *Turdus amaurochalinus* | Brazil | Jequitinhonha | TULEU01 | 82 | CSA | (Lacorte *et al*., 2013) |
| JX021460 | -16,437 | -41,012 | *Plasmodium* sp. | *Turdus leucomelas* | Brazil | Jequitinhonha | TULEU01 | 82 | CSA | (Lacorte *et al*., 2013) |
| JX021460 | -16,11 | -40,022 | *Plasmodium* sp. | *Thamnophilus ambiguus* | Brazil | Salto da Divisa | TULEU01 | 82 | CSA | (Lacorte *et al*., 2013) |
| JX029872 | -18,713 | -44,925 | *Plasmodium* sp. | *Turdus leucomelas* | Brazil | Felixlândia | TULEU04 | 82 | CSA | (Lacorte *et al*., 2013) |
| JX021460 | -17,0003 | -46,008 | *Plasmodium* sp. | *Turdus leucomelas* | Brazil | Brasilandia de Minas | TULEU01 | 82 | CSA | (Lacorte *et al*., 2013) |
| JX029885 | -17,111 | -43,82 | *Plasmodium* sp. | *Myiobius barbatus* | Brazil | Bocaiúva | MYSWA01 | 83 | CSA | (Lacorte *et al*., 2013) |
| JX029885 | -17,0003 | -46,008 | *Plasmodium* sp. | *Myiarchus ferox* | Brazil | Brasilandia de Minas | MYSWA01 | 83 | CSA | (Lacorte *et al*., 2013) |
| JX029891 | -17,111 | -43,82 | *Plasmodium* sp. | *Thraupis sayaca* | Brazil | Bocaiúva | THSAY01 | 83 | CSA | (Lacorte *et al*., 2013) |
| JX029891 | -17,111 | -43,82 | *Plasmodium* sp. | *Thlypopsis sordida* | Brazil | Bocaiúva | THSAY01 | 83 | CSA | (Lacorte *et al*., 2013) |
| KU562768 | -1,35 | -56,367 | *Plasmodium* sp. | *Dendrocincla fuliginosa* | Brazil | Porto Trombetas | NA | 83 | AMN | (Fecchio *et al*., 2017) |
| JX029885 | -17,111 | -43,82 | *Plasmodium* sp. | *Myiarchus swainsoni* | Brazil | Bocaiúva | MYSWA01 | 83 | CSA | (Lacorte *et al*., 2013) |
| JX021475 | -17,0003 | -46,008 | *Plasmodium* sp. | *Tachyphonus rufus* | Brazil | Brasilandia de Minas | TARUF01 | 84 | CSA | (Lacorte *et al*., 2013) |
| JX021475 | -19,789 | -42,141 | *Plasmodium* sp. | *Tangara cayana* | Brazil | Caratinga | TARUF01 | 84 | CSA | (Lacorte *et al*., 2013) |
| JX021475 | -18,713 | -44,925 | *Plasmodium* sp. | *Myiothlypis flaveola* | Brazil | Felixlândia | TARUF01 | 84 | CSA | (Lacorte *et al*., 2013) |
| JX021475 | -18,713 | -44,925 | *Plasmodium* sp. | *Saltator similis* | Brazil | Felixlândia | TARUF01 | 84 | CSA | (Lacorte *et al*., 2013) |
| JX029875 | -17,0003 | -46,008 | *Plasmodium* sp. | *Basileuterus culicivorus* | Brazil | Brasilandia de Minas | BAHYP01 | 84 | CSA | (Lacorte *et al*., 2013) |
| JX029875 | -18,713 | -44,925 | *Plasmodium* sp. | *Coereba flaveola* | Brazil | Felixlândia | BAHYP01 | 84 | CSA | (Lacorte *et al*., 2013) |
| JX029875 | -18,713 | -44,925 | *Plasmodium* sp. | *Coryphospingus pileatus* | Brazil | Felixlândia | BAHYP01 | 84 | CSA | (Lacorte *et al*., 2013) |
| JX029890 | -17,0003 | -46,008 | *Plasmodium* sp. | *Myiothlypis flaveola* | Brazil | Brasilandia de Minas | BFLA05 | 84 | CSA | (Lacorte *et al*., 2013) |
| JX029876 | -16,437 | -41,012 | *Plasmodium* sp. | *Malacoptila striata* | Brazil | Jequitinhonha | MASTR01 | 85 | CSA | (Lacorte *et al*., 2013) |
| JX029878 | -17,111 | -43,82 | *Plasmodium* sp. | *Turdus leucomelas* | Brazil | Bocaiúva | TULEU06 | 85 | CSA | (Lacorte *et al*., 2013) |
| JX029878 | -17,0003 | -46,008 | *Plasmodium* sp. | *Pachyramphus viridis* | Brazil | Brasilandia de Minas | TULEU06 | 85 | CSA | (Lacorte *et al*., 2013) |
| JX029878 | -16,437 | -41,012 | *Plasmodium* sp. | *Turdus leucomelas* | Brazil | Jequitinhonha | TULEU06 | 85 | CSA | (Lacorte *et al*., 2013) |
| JX029881 | -18,713 | -44,925 | *Plasmodium* sp. | *Turdus amaurochalinus* | Brazil | Felixlândia | TULEU07 | 85 | CSA | (Lacorte *et al*., 2013) |
| JX029881 | -18,713 | -44,925 | *Plasmodium* sp. | *Turdus leucomelas* | Brazil | Felixlândia | TULEU07 | 85 | CSA | (Lacorte *et al*., 2013) |
| KU562570 | -15,53 | -47,55 | *Plasmodium* sp. | *Turdus leucomelas* | Brazil | Planaltina | NA | 85 | CSA | (Fecchio *et al*., 2017) |
| JX029874 | -18,713 | -44,925 | *Plasmodium* sp. | *Turdus leucomelas* | Brazil | Felixlândia | TULEU05 | 85 | CSA | (Lacorte *et al*., 2013) |
| JX029880 | -17,0003 | -46,008 | *Plasmodium* sp. | *Galbula ruficauda* | Brazil | Brasilandia de Minas | GARUF01 | 86 | CSA | (Lacorte *et al*., 2013) |
| JQ988585 | -13,249 | -72,169 | *Parahaemoproteus* sp. | *Thripadectes melanorhynchus* | Peru | Cusco | NA | 87 | CAN | (Galen and Witt, 2014) |
| JX029910 | -16,437 | -41,012 | *Haemoproteus* sp. | *Synallaxis cinerea* | Brazil | Jequitinhonha | SYCIN01 | 87 | CSA | (Lacorte *et al*., 2013) |
| JX029907 | -16,11 | -40,022 | *Haemoproteus* sp. | *Pachyramphus polychopterus* | Brazil | Salto da Divisa | PAPOL02 | 88 | CSA | (Lacorte *et al*., 2013) |
| DQ241552 | 4,86 | -58,93 | *Haemoproteus* sp. | *Streptoprocne zonaris* | Guyana | NA | 45 | 89 | AMN | (Durrant *et al*., 2006) |
| JX029908 | -16,11 | -40,022 | *Haemoproteus* sp. | *Myiarchus tyrannulus* | Brazil | Salto da Divisa | TOFLA02 | 89 | CSA | (Lacorte *et al*., 2013) |
| JX029912 | -22,959 | -44,041 | *Haemoproteus* sp. | *Pachyramphus polychopterus* | Brazil | Manga | PAPOL03 | 89 | ATL | (Lacorte *et al*., 2013) |
| JX029912 | -16,11 | -40,022 | *Haemoproteus* sp. | *Pachyramphus polychopterus* | Brazil | Salto da Divisa | PAPOL03 | 89 | CSA | (Lacorte *et al*., 2013) |
| KF482354 | -3,944 | -73,607 | *Haemoproteus* sp. | *Pachyramphus polychopterus* | Peru | Allpahuayo Mishana National Reserve | NA | 89 | AMN | (Ricopa & Villa, 2016) |
| KU562171 | -6,602 | -40,124 | *Haemoproteus* sp. | *Pachyramphus marginatus* | Brazil | Aiuaba | NA | 89 | CSA | (Fecchio *et al*., 2017) |
| KU562172 | -6,602 | -40,124 | *Haemoproteus* sp. | *Pachyramphus polychopterus* | Brazil | Aiuaba | NA | 89 | CSA | (Fecchio *et al*., 2017) |
| KU562173 | -15,53 | -47,55 | *Haemoproteus* sp. | *Pachyramphus marginatus* | Brazil | Planaltina | NA | 89 | CSA | (Fecchio *et al*., 2017) |
| JX029908 | -16,437 | -41,012 | *Haemoproteus* sp. | *Tolmomyias flaviventris* | Brazil | Jequitinhonha | TOFLA02 | 89 | CSA | (Lacorte *et al*., 2013) |
| JX029909 | -22,959 | -44,041 | *Haemoproteus* sp. | *Leptotila verreauxi* | Brazil | Manga | LERUF01 | 90 | ATL | (Lacorte *et al*., 2013) |
| KU562145 | -4,5 | -56,267 | *Haemoproteus* sp. | *Leptotila rufaxilla* | Brazil | Tapajόs River | NA | 90 | AMS | (Fecchio *et al*., 2017) |
| KU562146 | -6,602 | -40,124 | *Haemoproteus* sp. | *Leptotila rufaxilla* | Brazil | Aiuaba | NA | 90 | CSA | (Fecchio *et al*., 2017) |
| JX029909 | -16,437 | -41,012 | *Haemoproteus* sp. | *Leptotila rufaxilla* | Brazil | Jequitinhonha | LERUF01 | 90 | CSA | (Lacorte *et al*., 2013) |
| JX029914 | -17,0003 | -46,008 | *Haemoproteus* sp. | *Myiodynastes maculatus* | Brazil | Brasilandia de Minas | MYMAC01 | 91 | CSA | (Lacorte *et al*., 2013) |
| JX029898 | -19,789 | -42,141 | *Plasmodium* sp. | *Trichothraupis melanops* | Brazil | Caratinga | THSAY02 | 92 | CSA | (Lacorte *et al*., 2013) |
| JX029898 | -19,82 | -40,276 | *Plasmodium* sp. | *Thraupis sayaca* | Brazil | Aracruz | THSAY02 | 92 | ATL | (Lacorte *et al*., 2013) |
| JX029899 | -19,993 | -43,848 | *Plasmodium* sp. | *Conopophaga lineata* | Brazil | Nova Lima | COLIN11 | 93 | CSA | (Lacorte *et al*., 2013) |
| JX029897 | -19,82 | -40,276 | *Plasmodium* sp. | *Troglodytes musculus* | Brazil | Aracruz | VIOLI01 | 94 | ATL | (Lacorte *et al*., 2013) |
| JX029897 | -19,82 | -40,276 | *Plasmodium* sp. | *Vireo olivaceus* | Brazil | Aracruz | VIOLI01 | 94 | ATL | (Lacorte *et al*., 2013) |
| JX029921 | -17,0003 | -46,008 | *Haemoproteus* sp. | *Claravis pretiosa* | Brazil | Brasilandia de Minas | COSQU01 | 95 | CSA | (Lacorte *et al*., 2013) |
| JX029921 | -18,713 | -44,925 | *Haemoproteus* sp. | *Columbina squammata* | Brazil | Felixlândia | COSQU01 | 95 | CSA | (Lacorte *et al*., 2013) |
| JX029921 | -22,959 | -44,041 | *Haemoproteus* sp. | *Columbina squammata* | Brazil | Manga | COSQU01 | 95 | ATL | (Lacorte *et al*., 2013) |
| JX029921 | -17,111 | -43,82 | *Haemoproteus* sp. | *Claravis pretiosa* | Brazil | Bocaiúva | COSQU01 | 95 | CSA | (Lacorte *et al*., 2013) |
| JX029902 | -16,11 | -40,022 | *Haemoproteus* sp. | *Coryphospingus pileatus* | Brazil | Salto da Divisa | CARUF01 | 96 | CSA | (Lacorte *et al*., 2013) |
| JX029902 | -18,713 | -44,925 | *Haemoproteus* sp. | *Casiornis rufus* | Brazil | Felixlândia | CARUF01 | 96 | CSA | (Lacorte *et al*., 2013) |
| JX021480 | -19,789 | -42,141 | *Plasmodium* sp. | *Conopophaga lineata* | Brazil | Caratinga | COLIN02 | 97 | CSA | (Lacorte *et al*., 2013) |
| JX021482 | -19,789 | -42,141 | *Plasmodium* sp. | *Conopophaga lineata* | Brazil | Caratinga | COLIN04 | 98 | CSA | (Lacorte *et al*., 2013) |
| JX021467 | -19,789 | -42,141 | *Plasmodium* sp. | *Trichothraupis melanops* | Brazil | Caratinga | TRMEL01 | 99 | CSA | (Lacorte *et al*., 2013) |
| JX021465 | -19,789 | -42,141 | *Plasmodium* sp. | *Drymophila squamata* | Brazil | Caratinga | FOSER01 | 100 | CSA | (Lacorte *et al*., 2013) |
| JX021465 | -19,789 | -42,141 | *Plasmodium* sp. | *Formicivora serrana* | Brazil | Caratinga | FOSER01 | 100 | CSA | (Lacorte *et al*., 2013) |
| JX029870 | -17,111 | -43,82 | *Plasmodium* sp. | *Formicivora melanogaster* | Brazil | Bocaiúva | FOMEL01 | 100 | CSA | (Lacorte *et al*., 2013) |
| KU562703 | -6,582 | -37,267 | *Plasmodium* sp. | *Formicivora melanogaster* | Brazil | Serra Negra do Norte | NA | 100 | ATL | (Fecchio *et al*., 2017) |
| JX021465 | -17,111 | -43,82 | *Plasmodium* sp. | *Formicivora melanogaster* | Brazil | Bocaiúva | FOSER01 | 100 | CSA | (Lacorte *et al*., 2013) |
| JX021464 | -16,437 | -41,012 | *Plasmodium* sp. | *Chamaeza campanisona* | Brazil | Jequitinhonha | CHCAM01 | 101 | CSA | (Lacorte *et al*., 2013) |
| MF077675 | 35,533 | -107,349 | *Haemoproteus* sp. | *Vireo plumbeus* | United States of America | Nuevo Mexico, Mesa Chivato | VIRPLU03 | 102 | NA | (Marroquin-Flores *et al*., 2017) |
| MF077674 | 35,529 | -107,356 | *Haemoproteus* sp. | *Vireo plumbeus* | United States of America | Nuevo Mexico, Mesa Chivato | VIRPLU04 | 102 | NA | (Marroquin-Flores *et al*., 2017) |
| DQ241538 | -32,522 | -55,765 | *Plasmodium* sp. | *Chrysomus ruficapillus* | Uruguay | NA | 31 | 103 | PAM | (Durrant *et al*., 2006) |
| DQ241538 | -32,522 | -55,765 | *Plasmodium* sp. | *Polioptila dumicola* | Uruguay | NA | 31 | 103 | PAM | (Durrant *et al*., 2006) |
| DQ241538 | -32,522 | -55,765 | *Plasmodium* sp. | *Polioptila dumicola* | Uruguay | NA | 31 | 103 | PAM | (Durrant *et al*., 2006) |
| DQ241538 | 4,86 | -58,93 | *Plasmodium* sp. | *Pitylus grossus* | Guyana | NA | 31 | 103 | AMN | (Durrant *et al*., 2006) |
| DQ241538 | 4,86 | -58,93 | *Plasmodium* sp. | *Saltator maximus* | Guyana | NA | 31 | 103 | AMN | (Durrant *et al*., 2006) |
| DQ241538 | 4,86 | -58,93 | *Plasmodium* sp. | *Saltator maximus* | Guyana | NA | 31 | 103 | AMN | (Durrant *et al*., 2006) |
| DQ241538 | 4,86 | -58,93 | *Plasmodium* sp. | *Cyanocompsa cyanoides* | Guyana | NA | 31 | 103 | AMN | (Durrant *et al*., 2006) |
| DQ241538 | 4,86 | -58,93 | *Plasmodium* sp. | *Cacicus cela* | Guyana | NA | 31 | 103 | AMN | (Durrant *et al*., 2006) |
| DQ368381 | NA | NA | *Plasmodium* sp. | *Acrocephalus arundinaceus* | No se reporta en el Genbank | NA | SYAT05 | 103 | NA | (Walther *et al*., 2014) |
| DQ368381 | -23,65 | -46,617 | *Plasmodium elongatum* | *Coscoroba coscoroba* | Brazil | São Paulo Zoo | NA | 103 | ATL | (Chagas *et al*., 2017) |
| DQ368381 | -23,65 | -46,617 | *Plasmodium elongatum* | *Cygnus atratus* | Brazil | São Paulo Zoo | NA | 103 | ATL | (Chagas *et al*., 2017) |
| DQ368381 | -23,65 | -46,617 | *Plasmodium elongatum* | *Pavo cristatus* | Brazil | São Paulo Zoo | NA | 103 | ATL | (Chagas *et al*., 2017) |
| JQ764620 | 10,231 | -67,285 | *Plasmodium* sp. | *Formicarius analis* | Venezuela | Aragua | PVE3 | 103 | NAN | (Mijares *et al*., 2012) |
| JQ764621 | 10,231 | -67,285 | *Plasmodium* sp. | *Chamaeza campanisona* | Venezuela | Aragua | PVE4 | 103 | NAN | (Mijares *et al*., 2012) |
| JX021463 | -17,0003 | -46,008 | *Plasmodium* sp. | *Dacnis cayana* | Brazil | Brasilandia de Minas | PADOM11 | 103 | CSA | (Lacorte *et al*., 2013) |
| JX021463 | -17,0003 | -46,008 | *Plasmodium* sp. | *Sittasomus griseicapillus* | Brazil | Brasilandia de Minas | PADOM11 | 103 | CSA | (Lacorte *et al*., 2013) |
| JX021463 | -18,713 | -44,925 | *Plasmodium* sp. | *Myiothlypis flaveola* | Brazil | Felixlândia | PADOM11 | 103 | CSA | (Lacorte *et al*., 2013) |
| JX021463 | -18,713 | -44,925 | *Plasmodium* sp. | *Coryphospingus pileatus* | Brazil | Felixlândia | PADOM11 | 103 | CSA | (Lacorte *et al*., 2013) |
| JX021463 | -16,437 | -41,012 | *Plasmodium* sp. | *Trichothraupis melanops* | Brazil | Jequitinhonha | PADOM11 | 103 | CSA | (Lacorte *et al*., 2013) |
| JX021463 | -19,993 | -43,848 | *Plasmodium* sp. | *Saltator similis* | Brazil | Nova Lima | PADOM11 | 103 | CSA | (Lacorte *et al*., 2013) |
| JX021471 | -17,111 | -43,82 | *Plasmodium* sp. | *Coryphospingus pileatus* | Brazil | Bocaiúva | COPIL01 | 103 | CSA | (Lacorte *et al*., 2013) |
| JX021471 | -17,0003 | -46,008 | *Plasmodium* sp. | *Nonnula rubecula* | Brazil | Brasilandia de Minas | COPIL01 | 103 | CSA | (Lacorte *et al*., 2013) |
| JX021471 | -16,11 | -40,022 | *Plasmodium* sp. | *Troglodytes musculus* | Brazil | Salto da Divisa | COPIL01 | 103 | CSA | (Lacorte *et al*., 2013) |
| JX021471 | -16,11 | -40,022 | *Plasmodium* sp. | *Volatinia jacarina* | Brazil | Salto da Divisa | COPIL01 | 103 | CSA | (Lacorte *et al*., 2013) |
| JX029877 | -19,82 | -40,276 | *Plasmodium* sp. | *Malacoptila striata* | Brazil | Aracruz | GRW06 | 103 | ATL | (Lacorte *et al*., 2013) |
| JX029877 | -19,82 | -40,276 | *Plasmodium* sp. | *Turdus leucomelas* | Brazil | Aracruz | GRW06 | 103 | ATL | (Lacorte *et al*., 2013) |
| JX029877 | -17,0003 | -46,008 | *Plasmodium* sp. | *Basileuterus hypoleucus* | Brazil | Brasilandia de Minas | GRW06 | 103 | CSA | (Lacorte *et al*., 2013) |
| JX029877 | -16,437 | -41,012 | *Plasmodium* sp. | *Turdus leucomelas* | Brazil | Jequitinhonha | GRW06 | 103 | CSA | (Lacorte *et al*., 2013) |
| KC867660 | 44,451 | -73,122 | *Plasmodium* sp. | *Dolichonyx oryzivorus* | United States of America | Vermont - northwestern Vermont | PADOM11 | 103 | NA | (Levin *et al*., 2013) |
| KC867661 | 43,193 | -71,572 | *Plasmodium* sp. | *Dolichonyx oryzivorus* | United States of America | New Hampshire - Platte River | PADOM11 | 103 | NA | (Levin *et al*., 2013) |
| KJ527078 | -9,190 | -75,015 | *Plasmodium* sp. | *Merganetta armata* | Peru | NA | NA | 103 | NAN | (Smith and Ramey 2015) |
| KJ527081 | -9,190 | -75,015 | *Plasmodium* sp. | *Merganetta armata* | Peru | NA | NA | 103 | NAN | (Smith and Ramey 2015) |
| KU057965 | -23,65 | -46,617 | *Plasmodium elongatum* | *Dendrocygna viduata* | Brazil | São Paulo Zoo | NA | 103 | ATL | (Chagas *et al*., 2016) |
| KU562396 | -4,5 | -56,283 | *Plasmodium* sp. | *Galbula cyanicollis* | Brazil | Tapajόs River | NA | 103 | AMS | (Fecchio *et al*., 2017) |
| KU562397 | -4,983 | -62,13 | *Plasmodium* sp. | *Galbula cyanicollis* | Brazil | Purus River | NA | 103 | AMN | (Fecchio *et al*., 2017) |
| KU562398 | -9,283 | -64,73 | *Plasmodium* sp. | *Galbula cyanicollis* | Brazil | Madeira River | NA | 103 | AMS | (Fecchio *et al*., 2017) |
| KU562399 | -4,5 | -56,283 | *Plasmodium* sp. | *Galbula cyanicollis* | Brazil | Tapajόs River | NA | 103 | AMS | (Fecchio *et al*., 2017) |
| KU562548 | -15,53 | -47,55 | *Plasmodium* sp. | *Volatinia jacarina* | Brazil | Planaltina | NA | 103 | CSA | (Fecchio *et al*., 2017) |
| KU562549 | -15,53 | -47,55 | *Plasmodium* sp. | *Neothraupis fasciata* | Brazil | Planaltina | NA | 103 | CSA | (Fecchio *et al*., 2017) |
| KU562550 | -15,53 | -47,55 | *Plasmodium* sp. | *Neothraupis fasciata* | Brazil | Planaltina | NA | 103 | CSA | (Fecchio *et al*., 2017) |
| KU562551 | -3,7 | -46,75 | *Plasmodium* sp. | *Tachyphonus rufus* | Brazil | Gurupi | NA | 103 | AMS | (Fecchio *et al*., 2017) |
| KU562552 | -3,7 | -46,75 | *Plasmodium* sp. | *Coereba flaveola* | Brazil | Gurupi | NA | 103 | AMS | (Fecchio *et al*., 2017) |
| KU562666 | -5,717 | -63,2 | *Plasmodium elongatum* | *Dendrocincla merula* | Brazil | Purus River | NA | 103 | AMN | (Fecchio *et al*., 2017) |
| KU562667 | -5,717 | -63,2 | *Plasmodium elongatum* | *Glyphorynchus spirurus* | Brazil | Purus River | NA | 103 | AMN | (Fecchio *et al*., 2017) |
| KU562668 | -19,597 | -57,017 | *Plasmodium elongatum* | *Donacobius atricapilla* | Brazil | Corumbá | NA | 103 | CSA | (Fecchio *et al*., 2017) |
| KU562669 | -3,7 | -46,75 | *Plasmodium elongatum* | *Sporophila americana* | Brazil | Gurupi | NA | 103 | AMS | (Fecchio *et al*., 2017) |
| KU562670 | -9,1167 | -64,467 | *Plasmodium elongatum* | *Dendrocincla merula* | Brazil | Porto Velho | NA | 103 | AMS | (Fecchio *et al*., 2017) |
| KU562671 | -9,15 | -64,5 | *Plasmodium elongatum* | *Dendrocincla merula* | Brazil | Porto Velho | NA | 103 | AMS | (Fecchio *et al*., 2017) |
| KU562676 | -5,717 | -63,2 | *Plasmodium* sp. | *Cyanocompsa cyanoides* | Brazil | Purus River | NA | 103 | AMN | (Fecchio *et al*., 2017) |
| KU562704 | -6,582 | -37,267 | *Plasmodium* sp. | *Coryphospingus pileatus* | Brazil | Serra Negra do Norte | NA | 103 | ATL | (Fecchio *et al*., 2017) |
| JX021463 | -17,111 | -43,82 | *Plasmodium* sp. | *Polioptila plumbea* | Brazil | Bocaiúva | PADOM11 | 103 | CSA | (Lacorte *et al*., 2013) |
| JX029861 | -16,11 | -40,022 | *Plasmodium* sp. | *Tiaris fuliginosus* | Brazil | Salto da Divisa | BAFLA04 | 104 | CSA | (Lacorte *et al*., 2013) |
| JX029861 | -16,11 | -40,022 | *Plasmodium* sp. | *Paroaria dominicana* | Brazil | Salto da Divisa | BAFLA04 | 104 | CSA | (Lacorte *et al*., 2013) |
| KU562527 | -6,602 | -40,124 | *Plasmodium* sp. | *Thamnophilus pelzelni* | Brazil | Aiuaba | NA | 104 | CSA | (Fecchio *et al*., 2017) |
| KU562528 | -6,602 | -40,124 | *Plasmodium* sp. | *Coereba flaveola* | Brazil | Aiuaba | NA | 104 | CSA | (Fecchio *et al*., 2017) |
| KU562529 | -5,923 | -35,175 | *Plasmodium* sp. | *Tachyphonus rufus* | Brazil | Ponta Negra | NA | 104 | ATL | (Fecchio *et al*., 2017) |
| KU562530 | -5,923 | -35,175 | *Plasmodium* sp. | *Tachyphonus rufus* | Brazil | Ponta Negra | NA | 104 | ATL | (Fecchio *et al*., 2017) |
| KU562531 | -6,582 | -37,267 | *Plasmodium* sp. | *Coryphospingus pileatus* | Brazil | Serra Negra do Norte | NA | 104 | ATL | (Fecchio *et al*., 2017) |
| KU562692 | -19,567 | -57,017 | *Plasmodium* sp. | *Paroaria capitata* | Brazil | Corumbá | NA | 104 | CSA | (Fecchio *et al*., 2017) |
| KU562693 | -12,567 | -70,083 | *Plasmodium* sp. | *Habia rubica* | Peru | Manu | NA | 104 | CAN | (Fecchio *et al*., 2017) |
| KU562711 | -3,7 | -46,75 | *Plasmodium* sp. | *Thamnophilus amazonicus* | Brazil | Gurupi | NA | 104 | AMS | (Fecchio *et al*., 2017) |
| KU562713 | -3,7 | -46,75 | *Plasmodium* sp. | *Xenops minutus* | Brazil | Gurupi | NA | 104 | AMS | (Fecchio *et al*., 2017) |
| KU562722 | -3,7 | -46,75 | *Plasmodium* sp. | *Piculus flavigula* | Brazil | Gurupi | NA | 104 | AMS | (Fecchio *et al*., 2017) |
| KU562818 | -12,567 | -70,083 | *Plasmodium* sp. | *Habia rubica* | Peru | Manu | NA | 104 | CAN | (Fecchio *et al*., 2017) |
| JX029861 | -18,713 | -44,925 | *Plasmodium* sp. | *Myiothlypis flaveola* | Brazil | Felixlândia | BAFLA04 | 104 | CSA | (Lacorte *et al*., 2013) |
| MF077677 | 35,532 | -107,350 | *Haemoproteus* sp. | *Setophaga coronata* | United States of America | Nuevo Mexico, Mesa Chivato | SETCOR01 | 105 | NA | (Marroquin-Flores *et al*., 2017) |
| JX021494 | -19,993 | -43,848 | *Plasmodium* sp. | *Conopophaga lineata* | Brazil | Nova Lima | COLIN10 | 106 | CSA | (Lacorte *et al*., 2013) |
| JX021487 | -16,11 | -40,022 | *Plasmodium* sp. | *Pyriglena leucoptera* | Brazil | Salto da Divisa | PYLEU04 | 107 | CSA | (Lacorte *et al*., 2013) |
| JX021491 | -19,993 | -43,848 | *Plasmodium* sp. | *Conopophaga lineata* | Brazil | Nova Lima | COLIN07 | 108 | CSA | (Lacorte *et al*., 2013) |
| JX021490 | -19,993 | -43,848 | *Plasmodium* sp. | *Conopophaga lineata* | Brazil | Nova Lima | COLIN06 | 109 | CSA | (Lacorte *et al*., 2013) |
| DQ241508 | -32,522 | -55,765 | *Plasmodium* sp. | *Troglodytes aedon* | Uruguay | NA | 1 | 110 | PAM | (Durrant *et al*., 2006) |
| DQ241508 | 4,86 | -58,93 | *Plasmodium* sp. | *Dolospingus fringilloides* | Guyana | NA | 1 | 110 | AMN | (Durrant *et al*., 2006) |
| DQ241508 | 4,86 | -58,93 | *Plasmodium* sp. | *Icterus nigrogularis* | Guyana | NA | 1 | 110 | AMN | (Durrant *et al*., 2006) |
| DQ241508 | 4,86 | -58,93 | *Plasmodium* sp. | *Icterus chrysocephalus* | Guyana | NA | 1 | 110 | AMN | (Durrant *et al*., 2006) |
| KU562545 | -15,53 | -47,55 | *Plasmodium* sp. | *Volatinia jacarina* | Brazil | Planaltina | NA | 110 | CSA | (Fecchio *et al*., 2017) |
| KU562546 | -15,53 | -47,55 | *Plasmodium* sp. | *Volatinia jacarina* | Brazil | Planaltina | NA | 110 | CSA | (Fecchio *et al*., 2017) |
| KU562547 | -3,7 | -46,75 | *Plasmodium* sp. | *Polioptila paraensis* | Brazil | Gurupi | NA | 110 | AMS | (Fecchio *et al*., 2017) |
| KU562685 | -19,567 | -57,017 | *Plasmodium* sp. | *Paroaria capitata* | Brazil | Corumbá | NA | 110 | CSA | (Fecchio *et al*., 2017) |
| KU562686 | -19,567 | -57,017 | *Plasmodium* sp. | *Paroaria capitata* | Brazil | Corumbá | NA | 110 | CSA | (Fecchio *et al*., 2017) |
| KU562687 | -19,567 | -57,017 | *Plasmodium* sp. | *Paroaria capitata* | Brazil | Corumbá | NA | 110 | CSA | (Fecchio *et al*., 2017) |
| KU562739 | -3,7 | -46,75 | *Plasmodium* sp. | *Tachyphonus rufus* | Brazil | Gurupi | NA | 110 | AMS | (Fecchio *et al*., 2017) |
| KU562793 | -9,33 | -64,7 | *Plasmodium* sp. | *Cyanocompsa cyanoides* | Brazil | Porto Velho | NA | 110 | AMS | (Fecchio *et al*., 2017) |
| JX021483 | -19,993 | -43,848 | *Plasmodium* sp. | *Basileuterus hypoleucus* | Brazil | Nova Lima | ICTCAY01 | 110 | CSA | (Lacorte *et al*., 2013) |
| KU562577 | -0,4 | -64,8 | *Plasmodium* sp. | *Formicarius colma* | Brazil | Negro River | NA | 111 | AMN | (Fecchio *et al*., 2017) |
| KU562578 | -0,4 | -64,8 | *Plasmodium* sp. | *Formicarius colma* | Brazil | Negro River | NA | 111 | AMN | (Fecchio *et al*., 2017) |
| KU562646 | -4,983 | -62,13 | *Plasmodium* sp. | *Formicarius colma* | Brazil | Purus River | NA | 111 | AMN | (Fecchio *et al*., 2017) |
| KU562647 | -4,983 | -62,13 | *Plasmodium* sp. | *Formicarius colma* | Brazil | Purus River | NA | 111 | AMN | (Fecchio *et al*., 2017) |
| KU562648 | -9,283 | -64,73 | *Plasmodium* sp. | *Formicarius colma* | Brazil | Madeira River | NA | 111 | AMS | (Fecchio *et al*., 2017) |
| KU562649 | -9,283 | -64,73 | *Plasmodium* sp. | *Formicarius colma* | Brazil | Madeira River | NA | 111 | AMS | (Fecchio *et al*., 2017) |
| KU562650 | -9,133 | -64,617 | *Plasmodium* sp. | *Formicarius colma* | Brazil | Porto Velho | NA | 111 | AMS | (Fecchio *et al*., 2017) |
| KU562591 | -0,4 | -64,8 | *Plasmodium* sp. | *Formicarius colma* | Brazil | Negro River | NA | 111 | AMN | (Fecchio *et al*., 2017) |
| KU562427 | -4,683 | -56,63 | *Plasmodium* sp. | *Pipra fasciicauda* | Brazil | Tapajόs River | NA | 112 | AMS | (Fecchio *et al*., 2017) |
| KU562589 | -0,4 | -64,8 | *Plasmodium* sp. | *Dixiphia pipra* | Brazil | Negro River | NA | 112 | AMN | (Fecchio *et al*., 2017) |
| KU562613 | -4,983 | -62,13 | *Plasmodium* sp. | *Ceratopipra rubrocapilla* | Brazil | Purus River | NA | 113 | AMN | (Fecchio *et al*., 2017) |
| KU562612 | -0,583 | -64,917 | *Plasmodium* sp. | *Ceratopipra erythrocephala* | Brazil | Negro River | NA | 113 | AMN | (Fecchio *et al*., 2017) |
| KU562433 | -4,683 | -56,63 | *Plasmodium* sp. | *Xenops minutus* | Brazil | Tapajόs River | NA | 114 | AMS | (Fecchio *et al*., 2017) |
| JX029892 | -19,098 | -40,186 | *Plasmodium* sp. | *Dendrocincla turdina* | Brazil | Sooretama | DETUR01 | 115 | ATL | (Lacorte *et al*., 2013) |
| KU562452 | -4,5 | -56,283 | *Plasmodium* sp. | *Myrmotherula axillaris* | Brazil | Tapajόs River | NA | 115 | AMS | (Fecchio *et al*., 2017) |
| KU562453 | -4,5 | -56,283 | *Plasmodium* sp. | *Galbula cyanicollis* | Brazil | Tapajόs River | NA | 115 | AMS | (Fecchio *et al*., 2017) |
| KU562454 | -9,1167 | -64,467 | *Plasmodium* sp. | *Myrmotherula axillaris* | Brazil | Porto Velho | NA | 115 | AMS | (Fecchio *et al*., 2017) |
| KU562455 | -9,133 | -64,633 | *Plasmodium* sp. | *Myrmotherula axillaris* | Brazil | Madeira River | NA | 115 | AMS | (Fecchio *et al*., 2017) |
| KU562802 | -9,283 | -64,73 | *Plasmodium* sp. | *Malacoptila rufa* | Brazil | Porto Velho | NA | 115 | AMS | (Fecchio *et al*., 2017) |
| KU562451 | -4,5 | -56,283 | *Plasmodium* sp. | *Myrmoborus myotherinus* | Brazil | Tapajόs River | NA | 115 | AMS | (Fecchio *et al*., 2017) |
| KU562400 | -4,7 | -56,53 | *Plasmodium* sp. | *Phaethornis* sp. | Brazil | Tapajόs River | NA | 116 | AMS | (Fecchio *et al*., 2017) |
| KU562402 | -4,7 | -56,53 | *Plasmodium* sp. | *Myrmoborus myotherinus* | Brazil | Tapajόs River | NA | 117 | AMS | (Fecchio *et al*., 2017) |
| KU562492 | -4,5 | -56,267 | *Plasmodium* sp. | *Monasa nigrifrons* | Brazil | Tapajόs River | NA | 118 | AMS | (Fecchio *et al*., 2017) |
| KU562493 | -4,683 | -56,63 | *Plasmodium* sp. | *Thamnomanes saturninus* | Brazil | Tapajόs River | NA | 118 | AMS | (Fecchio *et al*., 2017) |
| KU562593 | -0,4 | -64,8 | *Plasmodium* sp. | *Formicarius colma* | Brazil | Negro River | NA | 118 | AMN | (Fecchio *et al*., 2017) |
| KU562494 | -0,4 | -64,8 | *Plasmodium* sp. | *Ramphastos tucanus* | Brazil | Negro River | NA | 118 | AMN | (Fecchio *et al*., 2017) |
| KU562498 | -4,5 | -56,283 | *Plasmodium* sp. | *Hypocnemis striata* | Brazil | Tapajόs River | NA | 119 | AMS | (Fecchio *et al*., 2017) |
| KU562501 | -4,5 | -56,283 | *Plasmodium* sp. | *Hypocnemis striata* | Brazil | Tapajόs River | NA | 120 | AMS | (Fecchio *et al*., 2017) |
| KU562250 | -5,067 | -56,85 | *Plasmodium* sp. | *Phaethornis malaris* | Brazil | Tapajόs River | NA | 121 | AMS | (Fecchio *et al*., 2017) |
| KU562251 | -4,7 | -56,53 | *Plasmodium* sp. | *Hypocnemis striata* | Brazil | Tapajόs River | NA | 121 | AMS | (Fecchio *et al*., 2017) |
| KU562252 | -4,5 | -56,283 | *Plasmodium* sp. | *Myiobius barbatus* | Brazil | Tapajόs River | NA | 121 | AMS | (Fecchio *et al*., 2017) |
| KU562253 | -5,067 | -56,85 | *Plasmodium* sp. | *Terenotriccus erythrurus* | Brazil | Tapajόs River | NA | 121 | AMS | (Fecchio *et al*., 2017) |
| KU562259 | -5,067 | -56,85 | *Plasmodium* sp. | *Galbula cyanicollis* | Brazil | Tapajόs River | NA | 121 | AMS | (Fecchio *et al*., 2017) |
| KU562260 | -5,067 | -56,85 | *Plasmodium* sp. | *Thamnophilus amazonicus* | Brazil | Tapajόs River | NA | 121 | AMS | (Fecchio *et al*., 2017) |
| KU562261 | -5,217 | -56,917 | *Plasmodium* sp. | *Automolus paraensis* | Brazil | Tapajόs River | NA | 121 | AMS | (Fecchio *et al*., 2017) |
| KU562262 | -5,217 | -56,917 | *Plasmodium* sp. | *Attila spadiceus* | Brazil | Tapajόs River | NA | 121 | AMS | (Fecchio *et al*., 2017) |
| KU562263 | -5,217 | -56,917 | *Plasmodium* sp. | *Thamnophilus schistaceus* | Brazil | Tapajόs River | NA | 121 | AMS | (Fecchio *et al*., 2017) |
| KU562264 | -4,7 | -56,53 | *Plasmodium* sp. | *Epinecrophylla haematonota* | Brazil | Tapajόs River | NA | 121 | AMS | (Fecchio *et al*., 2017) |
| KU562265 | -4,7 | -56,53 | *Plasmodium* sp. | *Thamnomanes saturninus* | Brazil | Tapajόs River | NA | 121 | AMS | (Fecchio *et al*., 2017) |
| KU562266 | -5,1 | -56,43 | *Plasmodium* sp. | *Phlegopsis nigromaculata* | Brazil | Jamanxim River | NA | 121 | AMS | (Fecchio *et al*., 2017) |
| KU562267 | -5,217 | -56,917 | *Plasmodium* sp. | *Thamnophilus schistaceus* | Brazil | Tapajόs River | NA | 121 | AMS | (Fecchio *et al*., 2017) |
| KU562268 | -5,1 | -56,43 | *Plasmodium* sp. | *Willisornis poecilinotus* | Brazil | Jamanxim River | NA | 121 | AMS | (Fecchio *et al*., 2017) |
| KU562269 | -4,5 | -56,283 | *Plasmodium* sp. | *Phlegopsis nigromaculata* | Brazil | Tapajόs River | NA | 121 | AMS | (Fecchio *et al*., 2017) |
| KU562270 | -5,717 | -63,2 | *Plasmodium* sp. | *Willisornis poecilinotus* | Brazil | Purus River | NA | 121 | AMN | (Fecchio *et al*., 2017) |
| KU562271 | -5,717 | -63,2 | *Plasmodium* sp. | *Gymnopithys salvini* | Brazil | Purus River | NA | 121 | AMN | (Fecchio *et al*., 2017) |
| KU562272 | -19,567 | -57,017 | *Plasmodium* sp. | *Paroaria capitata* | Brazil | Corumbá | NA | 121 | CSA | (Fecchio *et al*., 2017) |
| KU562273 | -3,7 | -46,75 | *Plasmodium* sp. | *Rhynchocyclus olivaceus* | Brazil | Gurupi | NA | 121 | AMS | (Fecchio *et al*., 2017) |
| KU562274 | -5,067 | -56,85 | *Plasmodium* sp. | *Hypocnemis striata* | Brazil | Tapajόs River | NA | 121 | AMS | (Fecchio *et al*., 2017) |
| KU562276 | -5,067 | -56,85 | *Plasmodium* sp. | *Ramphocelus carbo* | Brazil | Tapajόs River | NA | 121 | AMS | (Fecchio *et al*., 2017) |
| KU562277 | -5,217 | -56,917 | *Plasmodium* sp. | *Dendrocolaptes certhia* | Brazil | Tapajόs River | NA | 121 | AMS | (Fecchio *et al*., 2017) |
| KU562360 | -4,5 | -56,283 | *Plasmodium* sp. | *Galbula cyanicollis* | Brazil | Tapajόs River | NA | 121 | AMS | (Fecchio *et al*., 2017) |
| KU562372 | -5,217 | -56,917 | *Plasmodium* sp. | *Mionectes macconnelli* | Brazil | Tapajόs River | NA | 121 | AMS | (Fecchio *et al*., 2017) |
| KU562378 | -4,7 | -56,53 | *Plasmodium* sp. | *Thamnomanes saturninus* | Brazil | Tapajόs River | NA | 121 | AMS | (Fecchio *et al*., 2017) |
| KU562510 | -4,5 | -56,267 | *Plasmodium* sp. | *Cranioleuca vulpina* | Brazil | Tapajόs River | NA | 121 | AMS | (Fecchio *et al*., 2017) |
| KU562662 | -5,717 | -63,2 | *Plasmodium* sp. | *Myrmotherula axillaris* | Brazil | Purus River | NA | 121 | AMN | (Fecchio *et al*., 2017) |
| KU562665 | -5,717 | -63,2 | *Plasmodium* sp. | *Willisornis poecilinotus* | Brazil | Purus River | NA | 121 | AMN | (Fecchio *et al*., 2017) |
| KU562688 | -19,567 | -57,017 | *Plasmodium* sp. | *Paroaria capitata* | Brazil | Corumbá | NA | 121 | CSA | (Fecchio *et al*., 2017) |
| KU562698 | -19,567 | -57,017 | *Plasmodium* sp. | *Paroaria coronata* | Brazil | Corumbá | NA | 121 | CSA | (Fecchio *et al*., 2017) |
| KU562728 | -3,7 | -46,75 | *Plasmodium* sp. | *Philydor erythropterum* | Brazil | Gurupi | NA | 121 | AMS | (Fecchio *et al*., 2017) |
| KU562745 | -3,7 | -46,75 | *Plasmodium* sp. | *Pachyramphus rufus* | Brazil | Gurupi | NA | 121 | AMS | (Fecchio *et al*., 2017) |
| KU562746 | -3,7 | -46,75 | *Plasmodium* sp. | *Formicivora grisea* | Brazil | Gurupi | NA | 121 | AMS | (Fecchio *et al*., 2017) |
| KU562747 | -3,7 | -46,75 | *Plasmodium* sp. | *Formicivora grisea* | Brazil | Gurupi | NA | 121 | AMS | (Fecchio *et al*., 2017) |
| KU562505 | -4,5 | -56,283 | *Plasmodium* sp. | *Myrmotherula axillaris* | Brazil | Tapajόs River | NA | 121 | AMS | (Fecchio *et al*., 2017) |
| KU562507 | -4,5 | -56,283 | *Plasmodium* sp. | *Hypocnemis striata* | Brazil | Tapajόs River | NA | 122 | AMS | (Fecchio *et al*., 2017) |
| KU562508 | -4,5 | -56,283 | *Plasmodium* sp. | *Hypocnemis striata* | Brazil | Tapajόs River | NA | 122 | AMS | (Fecchio *et al*., 2017) |
| KU562798 | -9,45 | -64,33 | *Plasmodium* sp. | *Hypocnemis ochrogyna* | Brazil | Porto Velho | NA | 122 | AMS | (Fecchio *et al*., 2017) |
| KU562504 | -4,5 | -56,283 | *Plasmodium* sp. | *Hypocnemis striata* | Brazil | Tapajόs River | NA | 122 | AMS | (Fecchio *et al*., 2017) |
| KU562503 | -4,5 | -56,283 | *Plasmodium* sp. | *Hypocnemis striata* | Brazil | Tapajόs River | NA | 123 | AMS | (Fecchio *et al*., 2017) |
| KU562458 | -4,5 | -56,283 | *Plasmodium* sp. | *Hylophylax punctulatus* | Brazil | Tapajόs River | NA | 124 | AMS | (Fecchio *et al*., 2017) |
| KU562459 | -4,5 | -56,283 | *Plasmodium* sp. | *Mionectes macconnelli* | Brazil | Tapajόs River | NA | 125 | AMS | (Fecchio *et al*., 2017) |
| KU562784 | -13,8 | -59,683 | *Plasmodium* sp. | *Machaeropterus pyrocephalus* | Brazil | Comodoro | NA | 126 | CSA | (Fecchio *et al*., 2017) |
| KU562779 | -13,8 | -59,683 | *Plasmodium* sp. | *Tachyphonus phoenicius* | Brazil | Comodoro | NA | 127 | CSA | (Fecchio *et al*., 2017) |
| KU562780 | -13,8 | -59,683 | *Plasmodium* sp. | *Tachyphonus phoenicius* | Brazil | Comodoro | NA | 127 | CSA | (Fecchio *et al*., 2017) |
| KU562716 | -3,7 | -46,75 | *Plasmodium* sp. | *Willisornis poecilinotus* | Brazil | Gurupi | NA | 128 | AMS | (Fecchio *et al*., 2017) |
| KU562717 | -3,7 | -46,75 | *Plasmodium* sp. | *Willisornis poecilinotus* | Brazil | Gurupi | NA | 128 | AMS | (Fecchio *et al*., 2017) |
| KU562718 | -3,7 | -46,75 | *Plasmodium* sp. | *Piprites chloris* | Brazil | Gurupi | NA | 128 | AMS | (Fecchio *et al*., 2017) |
| KU562719 | -3,7 | -46,75 | *Plasmodium* sp. | *Xiphorhynchus spixii* | Brazil | Gurupi | NA | 128 | AMS | (Fecchio *et al*., 2017) |
| KU562720 | -3,7 | -46,75 | *Plasmodium* sp. | *Willisornis poecilinotus* | Brazil | Gurupi | NA | 128 | AMS | (Fecchio *et al*., 2017) |
| KU562721 | -3,7 | -46,75 | *Plasmodium* sp. | *Piaya cayana* | Brazil | Gurupi | NA | 128 | AMS | (Fecchio *et al*., 2017) |
| KU562726 | -3,7 | -46,75 | *Plasmodium* sp. | *Willisornis poecilinotus* | Brazil | Gurupi | NA | 128 | AMS | (Fecchio *et al*., 2017) |
| KU562743 | -3,7 | -46,75 | *Plasmodium* sp. | *Pyrrhura lepida* | Brazil | Gurupi | NA | 128 | AMS | (Fecchio *et al*., 2017) |
| KU562814 | -12,567 | -70,083 | *Plasmodium* sp. | *Sciaphylax hemimelaena* | Peru | Manu | NA | 129 | CAN | (Fecchio *et al*., 2017) |
| KU562732 | -3,7 | -46,75 | *Plasmodium* sp. | *Willisornis poecilinotus* | Brazil | Gurupi | NA | 129 | AMS | (Fecchio *et al*., 2017) |
| KU562748 | -3,7 | -46,75 | *Plasmodium* sp. | *Formicivora grisea* | Brazil | Gurupi | NA | 130 | AMS | (Fecchio *et al*., 2017) |
| DQ241521 | -32,522 | -55,765 | *Plasmodium* sp. | *Leptotila verreauxi* | Uruguay | NA | 14 | 131 | PAM | (Durrant *et al*., 2006) |
| EU684543 | 37,09 | -95,712 | *Plasmodium* sp. | *Calidris melanotos* | United States of America | NA | NA | 131 | NA | (Yohannes *et al*., 2009) |
| EU684543 | -23,65 | -46,617 | *Plasmodium* sp. | *Buteogallus urubitinga* | Brazil | São Paulo Zoo | NA | 131 | ATL | (Chagas *et al*., 2017) |
| EU684543 | -23,65 | -46,617 | *Plasmodium* sp. | *Nycticorax nycticorax* | Brazil | São Paulo Zoo | NA | 131 | ATL | (Chagas *et al*., 2016) |
| EU684543 | -23,65 | -46,617 | *Plasmodium* sp. | *Cygnus atratus* | Brazil | São Paulo Zoo | NA | 131 | ATL | (Chagas *et al*., 2017) |
| EU684543 | -23,65 | -46,617 | *Plasmodium* sp. | *Cygnus melanocoryphus* | Brazil | São Paulo Zoo | NA | 131 | ATL | (Chagas *et al*., 2017) |
| JX029862 | -16,11 | -40,022 | *Plasmodium* sp. | *Phaeomyias murina* | Brazil | Salto da Divisa | PESA01 | 131 | CSA | (Lacorte *et al*., 2013) |
| KJ527080 | -38,416 | -63,617 | *Plasmodium* sp. | *Dendrocygna bicolor* | Argentina | NA | NA | 131 | PAM | (Smith and Ramey 2015) |
| KU057967 | -23,65 | -46,617 | *Plasmodium* sp. | *Cyanopsitta spixii* | Brazil | São Paulo Zoo | NA | 131 | ATL | (Chagas *et al*., 2017) |
| KU057967 | -23,65 | -46,617 | *Plasmodium* sp. | *Nycticorax nycticorax* | Brazil | São Paulo Zoo | NA | 131 | ATL | (Chagas *et al*., 2016) |
| KU057967 | -23,65 | -46,617 | *Plasmodium* sp. | *Sarcoramphus papa* | Brazil | São Paulo Zoo | NA | 131 | ATL | (Chagas *et al*., 2017) |
| KU057967 | -23,65 | -46,617 | *Plasmodium* sp. | *Alopochen aegyptiaca* | Brazil | São Paulo Zoo | NA | 131 | ATL | (Chagas *et al*., 2017) |
| KU057967 | -23,65 | -46,617 | *Plasmodium* sp. | *Amazonetta brasiliensis* | Brazil | São Paulo Zoo | NA | 131 | ATL | (Chagas *et al*., 2017) |
| KU057967 | -23,65 | -46,617 | *Plasmodium* sp. | *Anser cygnoides* | Brazil | São Paulo Zoo | NA | 131 | ATL | (Chagas *et al*., 2017) |
| KU057967 | -23,65 | -46,617 | *Plasmodium* sp. | *Cygnus atratus* | Brazil | São Paulo Zoo | NA | 131 | ATL | (Chagas *et al*., 2017) |
| KU057967 | -23,65 | -46,617 | *Plasmodium* sp. | *Cygnus melanocoryphus* | Brazil | São Paulo Zoo | NA | 131 | ATL | (Chagas *et al*., 2017) |
| KU057967 | -23,65 | -46,617 | *Plasmodium* sp. | *Dendrocygna viduata* | Brazil | São Paulo Zoo | NA | 131 | ATL | (Chagas *et al*., 2017) |
| KU057967 | -23,65 | -46,617 | *Plasmodium* sp. | *Plectropterus gambensis* | Brazil | São Paulo Zoo | NA | 131 | ATL | (Chagas *et al*., 2017) |
| KU057967 | -23,65 | -46,617 | *Plasmodium* sp. | *Tadorna ferruginea* | Brazil | São Paulo Zoo | NA | 131 | ATL | (Chagas *et al*., 2017) |
| KU057967 | -23,65 | -46,617 | *Plasmodium* sp. | *Tadorna variegata* | Brazil | São Paulo Zoo | NA | 131 | ATL | (Chagas *et al*., 2017) |
| KU057967 | -23,65 | -46,617 | *Plasmodium* sp. | *Pipile jacutinga* | Brazil | São Paulo Zoo | NA | 131 | ATL | (Chagas *et al*., 2017) |
| KU057967 | -23,65 | -46,617 | *Plasmodium* sp. | *Ramphastos toco* | Brazil | São Paulo Zoo | NA | 131 | ATL | (Chagas *et al*., 2017) |
| KU057967 | -23,65 | -46,617 | *Plasmodium* sp. | *Amazona aestiva* | Brazil | São Paulo Zoo | NA | 131 | ATL | (Chagas *et al*., 2017) |
| KU057967 | -23,65 | -46,617 | *Plasmodium* sp. | *Struthio camelus* | Brazil | São Paulo Zoo | NA | 131 | ATL | (Chagas *et al*., 2017) |
| KU057967 | -23,65 | -46,617 | *Plasmodium* sp. | *Anodorhynchus hyacinthinus* | Brazil | São Paulo Zoo | NA | 131 | ATL | (Chagas *et al*., 2017) |
| KU562663 | -5,717 | -63,2 | *Plasmodium* sp. | *Glyphorynchus spirurus* | Brazil | Purus River | NA | 131 | AMN | (Fecchio *et al*., 2017) |
| KU562787 | -13,8 | -59,683 | *Plasmodium* sp. | *Piprites chloris* | Brazil | Comodoro | NA | 131 | CSA | (Fecchio *et al*., 2017) |
| KU562822 | -12,567 | -70,083 | *Plasmodium* sp. | *Automolus ochrolaemus* | Peru | Manu | NA | 132 | CAN | (Fecchio *et al*., 2017) |
| KU562701 | -6,582 | -37,267 | *Plasmodium* sp. | *Coryphospingus pileatus* | Brazil | Serra Negra do Norte | NA | 133 | ATL | (Fecchio *et al*., 2017) |
| KU562702 | -6,582 | -37,267 | *Plasmodium* sp. | *Coryphospingus pileatus* | Brazil | Serra Negra do Norte | NA | 133 | ATL | (Fecchio *et al*., 2017) |
| KU562836 | -12,567 | -70,083 | *Plasmodium* sp. | *Dacnis cayana* | Peru | Manu | NA | 133 | CAN | (Fecchio *et al*., 2017) |
| KU562832 | -12,567 | -70,083 | *Plasmodium* sp. | *Pipra fasciicauda* | Peru | Manu | NA | 134 | CAN | (Fecchio *et al*., 2017) |
| KU562835 | -12,567 | -70,083 | *Plasmodium* sp. | *Euphonia xanthogaster* | Peru | Manu | NA | 135 | CAN | (Fecchio *et al*., 2017) |
| KU562800 | -9,45 | -64,33 | *Plasmodium* sp. | *Lepidothrix nattereri* | Brazil | Porto Velho | NA | 136 | AMS | (Fecchio *et al*., 2017) |
| KU562794 | -9,3 | -64,717 | *Plasmodium* sp. | *Dendrocincla merula* | Brazil | Porto Velho | NA | 137 | AMS | (Fecchio *et al*., 2017) |
| KU562801 | -9,45 | -64,383 | *Plasmodium* sp. | *Lepidothrix nattereri* | Brazil | Porto Velho | NA | 138 | AMS | (Fecchio *et al*., 2017) |
| KU562375 | -5,217 | -56,917 | *Plasmodium* sp. | *Willisornis poecilinotus* | Brazil | Tapajόs River | NA | 139 | AMS | (Fecchio *et al*., 2017) |
| KU562377 | -5,217 | -56,917 | *Plasmodium* sp. | *Willisornis poecilinotus* | Brazil | Tapajόs River | NA | 139 | AMS | (Fecchio *et al*., 2017) |
| KU562626 | -4,983 | -62,13 | *Plasmodium* sp. | *Hafferia fortis* | Brazil | Purus River | NA | 139 | AMN | (Fecchio *et al*., 2017) |
| KU562809 | -9,1167 | -64,467 | *Plasmodium* sp. | *Willisornis poecilinotus* | Brazil | Porto Velho | NA | 139 | AMS | (Fecchio *et al*., 2017) |
| KT373866 | -2,117 | -77,733 | *Plasmodium* sp. | *Myrmoborus myotherinus* | Ecuador | Morona-Santiago Province, Wisui | MYRMYO01 | 140 | AMN | (Moens & Pérez-Tris, 2016) |
| KT373866 | -2,117 | -77,733 | *Plasmodium* sp. | *Hylophylax naevius* | Ecuador | Morona-Santiago Province, Wisui | MYRMYO01 | 140 | AMN | (Moens & Pérez-Tris, 2016) |
| KT373866 | -2,117 | -77,733 | *Plasmodium* sp. | *Hafferia fortis* | Ecuador | Morona-Santiago Province, Wisui | MYRMYO01 | 140 | AMN | (Moens & Pérez-Tris, 2016) |
| KT373866 | -2,117 | -77,733 | *Plasmodium* sp. | *Gymnopithys leucaspis* | Ecuador | Morona-Santiago Province, Wisui | MYRMYO01 | 140 | AMN | (Moens & Pérez-Tris, 2016) |
| KT373866 | -2,117 | -77,733 | *Plasmodium* sp. | *Thamnomanes ardesiacus* | Ecuador | Morona-Santiago Province, Wisui | MYRMYO01 | 140 | AMN | (Moens & Pérez-Tris, 2016) |
| KT373866 | -2,087 | -77,751 | *Plasmodium* sp. | *Myrmoborus myotherinus* | Ecuador | Wisui reserve | NA | 140 | AMN | (Moens *et al*., 2017) |
| KU562322 | -5,1 | -56,43 | *Plasmodium* sp. | *Conopophaga aurita* | Brazil | Jamanxim River | NA | 140 | AMS | (Fecchio *et al*., 2017) |
| KU562323 | -4,683 | -56,63 | *Plasmodium* sp. | *Hypocnemis striata* | Brazil | Tapajόs River | NA | 140 | AMS | (Fecchio *et al*., 2017) |
| KU562324 | -4,683 | -56,63 | *Plasmodium* sp. | *Hypocnemis striata* | Brazil | Tapajόs River | NA | 140 | AMS | (Fecchio *et al*., 2017) |
| KU562325 | -4,5 | -56,283 | *Plasmodium* sp. | *Glyphorynchus spirurus* | Brazil | Tapajόs River | NA | 140 | AMS | (Fecchio *et al*., 2017) |
| KU562326 | -4,5 | -56,283 | *Plasmodium* sp. | *Myrmoborus myotherinus* | Brazil | Tapajόs River | NA | 140 | AMS | (Fecchio *et al*., 2017) |
| KU562327 | -4,5 | -56,283 | *Plasmodium* sp. | *Mionectes oleagineus* | Brazil | Tapajόs River | NA | 140 | AMS | (Fecchio *et al*., 2017) |
| KU562432 | -4,683 | -56,63 | *Plasmodium* sp. | *Myrmoborus myotherinus* | Brazil | Tapajόs River | NA | 140 | AMS | (Fecchio *et al*., 2017) |
| KU562807 | -9,283 | -64,73 | *Plasmodium* sp. | *Thamnophilus aethiops* | Brazil | Porto Velho | NA | 140 | AMS | (Fecchio *et al*., 2017) |
| KU562278 | -5,217 | -56,917 | *Plasmodium* sp. | *Myrmotherula longipennis* | Brazil | Tapajόs River | NA | 141 | AMS | (Fecchio *et al*., 2017) |
| KU562279 | -5,1 | -56,43 | *Plasmodium* sp. | *Myrmotherula longipennis* | Brazil | Jamanxim River | NA | 141 | AMS | (Fecchio *et al*., 2017) |
| KU562280 | -5,1 | -56,43 | *Plasmodium* sp. | *Myrmotherula longipennis* | Brazil | Jamanxim River | NA | 141 | AMS | (Fecchio *et al*., 2017) |
| KU562281 | -4,683 | -56,63 | *Plasmodium* sp. | *Myrmotherula longipennis* | Brazil | Tapajόs River | NA | 141 | AMS | (Fecchio *et al*., 2017) |
| KU562282 | -4,5 | -56,283 | *Plasmodium* sp. | *Myrmotherula axillaris* | Brazil | Tapajόs River | NA | 141 | AMS | (Fecchio *et al*., 2017) |
| KU562283 | -15,53 | -47,55 | *Plasmodium* sp. | *Neothraupis fasciata* | Brazil | Planaltina | NA | 141 | CSA | (Fecchio *et al*., 2017) |
| KU562672 | -5,717 | -63,2 | *Plasmodium* sp. | *Myrmotherula longipennis* | Brazil | Purus River | NA | 141 | AMN | (Fecchio *et al*., 2017) |
| KU562675 | -5,717 | -63,2 | *Plasmodium* sp. | *Myrmotherula axillaris* | Brazil | Purus River | NA | 142 | AMN | (Fecchio *et al*., 2017) |
| KT373877 | -2,117 | -77,733 | *Plasmodium* sp. | *Thamnomanes caesius* | Ecuador | Morona-Santiago Province, Wisui | THACAE01 | 143 | AMN | (Moens & Pérez-Tris, 2016) |
| KT373877 | -2,117 | -77,733 | *Plasmodium* sp. | *Hylophylax naevius* | Ecuador | Morona-Santiago Province, Wisui | THACAE01 | 143 | AMN | (Moens & Pérez-Tris, 2016) |
| KT373877 | -2,087 | -77,751 | *Plasmodium* sp. | *Thamnomanes caesius* | Ecuador | Wisui reserve | NA | 143 | AMN | (Moens *et al*., 2017) |
| KU562412 | -4,683 | -56,63 | *Plasmodium* sp. | *Hypocnemis striata* | Brazil | Tapajόs River | NA | 143 | AMS | (Fecchio *et al*., 2017) |
| KU562413 | -4,5 | -56,267 | *Plasmodium* sp. | *Leptotila rufaxilla* | Brazil | Tapajόs River | NA | 143 | AMS | (Fecchio *et al*., 2017) |
| KU562414 | -4,5 | -56,283 | *Plasmodium* sp. | *Phlegopsis nigromaculata* | Brazil | Tapajόs River | NA | 143 | AMS | (Fecchio *et al*., 2017) |
| KU562415 | -4,5 | -56,283 | *Plasmodium* sp. | *Thamnophilus aethiops* | Brazil | Tapajόs River | NA | 143 | AMS | (Fecchio *et al*., 2017) |
| KU562416 | -4,5 | -56,267 | *Plasmodium* sp. | *Thamnophilus nigrocinereus* | Brazil | Tapajόs River | NA | 143 | AMS | (Fecchio *et al*., 2017) |
| KU562417 | -4,983 | -62,13 | *Plasmodium* sp. | *Myrmotherula axillaris* | Brazil | Purus River | NA | 143 | AMN | (Fecchio *et al*., 2017) |
| KU562418 | -5,717 | -63,2 | *Plasmodium* sp. | *Myrmotherula axillaris* | Brazil | Purus River | NA | 143 | AMN | (Fecchio *et al*., 2017) |
| KU562419 | -5,717 | -63,2 | *Plasmodium* sp. | *Myrmotherula axillaris* | Brazil | Purus River | NA | 143 | AMN | (Fecchio *et al*., 2017) |
| KU562420 | -5,717 | -63,2 | *Plasmodium* sp. | *Willisornis poecilinotus* | Brazil | Purus River | NA | 143 | AMN | (Fecchio *et al*., 2017) |
| KU562421 | -3,7 | -46,75 | *Plasmodium* sp. | *Dysithamnus mentalis* | Brazil | Gurupi | NA | 143 | AMS | (Fecchio *et al*., 2017) |
| KU562422 | -1,35 | -56,367 | *Plasmodium* sp. | *Thamnophilus murinus* | Brazil | Porto Trombetas | NA | 143 | AMN | (Fecchio *et al*., 2017) |
| KU562423 | -1,35 | -56,367 | *Plasmodium* sp. | *Thamnophilus murinus* | Brazil | Porto Trombetas | NA | 143 | AMN | (Fecchio *et al*., 2017) |
| KU562424 | -1,35 | -56,367 | *Plasmodium* sp. | *Thamnophilus murinus* | Brazil | Porto Trombetas | NA | 143 | AMN | (Fecchio *et al*., 2017) |
| KU562425 | -12,567 | -70,083 | *Plasmodium* sp. | *Akletos goeldii* | Peru | Manu | NA | 143 | CAN | (Fecchio *et al*., 2017) |
| KU562426 | -12,567 | -70,083 | *Plasmodium* sp. | *Sciaphylax hemimelaena* | Peru | Manu | NA | 143 | CAN | (Fecchio *et al*., 2017) |
| KU562624 | -4,983 | -62,13 | *Plasmodium* sp. | *Dendrocincla merula* | Brazil | Purus River | NA | 143 | AMN | (Fecchio *et al*., 2017) |
| KU562625 | -5,717 | -63,2 | *Plasmodium* sp. | *Thamnophilus aethiops* | Brazil | Purus River | NA | 143 | AMN | (Fecchio *et al*., 2017) |
| KU562691 | -19,567 | -57,017 | *Plasmodium* sp. | *Paroaria capitata* | Brazil | Corumbá | NA | 143 | CSA | (Fecchio *et al*., 2017) |
| KU562813 | -12,567 | -70,083 | *Plasmodium* sp. | *Thamnophilus schistaceus* | Peru | Manu | NA | 143 | CAN | (Fecchio *et al*., 2017) |
| KU562821 | -12,567 | -70,083 | *Plasmodium* sp. | *Arremon taciturnus* | Peru | Manu | NA | 143 | CAN | (Fecchio *et al*., 2017) |
| KU562673 | -5,717 | -63,2 | *Plasmodium* sp. | *Myrmotherula longipennis* | Brazil | Purus River | NA | 143 | AMN | (Fecchio *et al*., 2017) |
| KU562627 | -4,983 | -62,13 | *Plasmodium* sp. | *Hafferia fortis* | Brazil | Purus River | NA | 144 | AMN | (Fecchio *et al*., 2017) |
| KU562683 | -19,567 | -57,017 | *Plasmodium* sp. | *Paroaria capitata* | Brazil | Corumbá | NA | 145 | CSA | (Fecchio *et al*., 2017) |
| KU562678 | -5,717 | -63,2 | *Plasmodium* sp. | *Ramphocelus carbo* | Brazil | Purus River | NA | 146 | AMN | (Fecchio *et al*., 2017) |
| KU562214 | -5,923 | -35,175 | *Haemoproteus* sp. | *Tachyphonus rufus* | Brazil | Ponta Negra | NA | 147 | ATL | (Fecchio *et al*., 2017) |
| KU562215 | -5,923 | -35,175 | *Haemoproteus* sp. | *Tachyphonus rufus* | Brazil | Ponta Negra | NA | 147 | ATL | (Fecchio *et al*., 2017) |
| KU562216 | -5,923 | -35,175 | *Haemoproteus* sp. | *Tachyphonus rufus* | Brazil | Ponta Negra | NA | 147 | ATL | (Fecchio *et al*., 2017) |
| KU562213 | -5,923 | -35,175 | *Haemoproteus* sp. | *Tachyphonus rufus* | Brazil | Ponta Negra | NA | 147 | ATL | (Fecchio *et al*., 2017) |
| KU562208 | -4,983 | -62,13 | *Haemoproteus* sp. | *Myrmoborus myotherinus* | Brazil | Purus River | NA | 148 | AMN | (Fecchio *et al*., 2017) |
| KU562209 | -4,983 | -62,13 | *Haemoproteus* sp. | *Gymnopithys salvini* | Brazil | Purus River | NA | 148 | AMN | (Fecchio *et al*., 2017) |
| KU562210 | -5,717 | -63,2 | *Haemoproteus* sp. | *Dendrocincla merula* | Brazil | Purus River | NA | 148 | AMN | (Fecchio *et al*., 2017) |
| KU562211 | -5,717 | -63,2 | *Haemoproteus* sp. | *Myrmoborus myotherinus* | Brazil | Purus River | NA | 148 | AMN | (Fecchio *et al*., 2017) |
| KU562212 | -4,983 | -62,13 | *Haemoproteus* sp. | *Gymnopithys salvini* | Brazil | Purus River | NA | 148 | AMN | (Fecchio *et al*., 2017) |
| DQ241559 | 4,86 | -58,93 | *Haemoproteus* sp. | *Columbina passerina* | Guyana | NA | 52 | 149 | AMN | (Durrant *et al*., 2006) |
| DQ241559 | 4,86 | -58,93 | *Haemoproteus* sp. | *Columbina talpacoti* | Guyana | NA | 52 | 149 | AMN | (Durrant *et al*., 2006) |
| KU562204 | -0,583 | -64,917 | *Haemoproteus* sp. | *Automolus infuscatus* | Brazil | Santa Isabel do Rio Negro | NA | 149 | AMN | (Fecchio *et al*., 2017) |
| KU562205 | -19,567 | -57,017 | *Haemoproteus* sp. | *Columbina talpacoti* | Brazil | Corumbá | NA | 149 | CSA | (Fecchio *et al*., 2017) |
| KU562206 | -3,7 | -46,75 | *Haemoproteus* sp. | *Myiophobus fasciatus* | Brazil | Gurupi | NA | 149 | AMS | (Fecchio *et al*., 2017) |
| KU562220 | -6,582 | -37,267 | *Haemoproteus* sp. | *Coryphospingus pileatus* | Brazil | Serra Negra do Norte | NA | 150 | ATL | (Fecchio *et al*., 2017) |
| KU562223 | -6,582 | -37,267 | *Haemoproteus* sp. | *Coryphospingus pileatus* | Brazil | Serra Negra do Norte | NA | 150 | ATL | (Fecchio *et al*., 2017) |
| KU562222 | -6,582 | -37,267 | *Haemoproteus* sp. | *Coryphospingus pileatus* | Brazil | Serra Negra do Norte | NA | 150 | ATL | (Fecchio *et al*., 2017) |
| KU562179 | -15,53 | -47,55 | *Haemoproteus* sp. | *Nystalus maculatus* | Brazil | Planaltina | NA | 151 | CSA | (Fecchio *et al*., 2017) |
| KU562180 | -15,53 | -47,55 | *Haemoproteus* sp. | *Nystalus maculatus* | Brazil | Planaltina | NA | 151 | CSA | (Fecchio *et al*., 2017) |
| KU562181 | -15,53 | -47,55 | *Haemoproteus* sp. | *Nystalus chacuru* | Brazil | Planaltina | NA | 151 | CSA | (Fecchio *et al*., 2017) |
| KU562196 | -15,53 | -47,55 | *Haemoproteus* sp. | *Nystalus chacuru* | Brazil | Planaltina | NA | 151 | CSA | (Fecchio *et al*., 2017) |
| KU562191 | -15,53 | -47,55 | *Haemoproteus* sp. | *Nystalus chacuru* | Brazil | Planaltina | NA | 152 | CSA | (Fecchio *et al*., 2017) |
| KU562192 | -15,53 | -47,55 | *Haemoproteus* sp. | *Nystalus chacuru* | Brazil | Planaltina | NA | 152 | CSA | (Fecchio *et al*., 2017) |
| KU562193 | -15,53 | -47,55 | *Haemoproteus* sp. | *Nystalus chacuru* | Brazil | Planaltina | NA | 152 | CSA | (Fecchio *et al*., 2017) |
| KU562194 | -15,53 | -47,55 | *Haemoproteus* sp. | *Nystalus chacuru* | Brazil | Planaltina | NA | 152 | CSA | (Fecchio *et al*., 2017) |
| KU562195 | -6,582 | -37,267 | *Haemoproteus* sp. | *Nystalus maculatus* | Brazil | Serra Negra do Norte | NA | 152 | ATL | (Fecchio *et al*., 2017) |
| KU562219 | -6,582 | -37,267 | *Haemoproteus* sp. | *Nystalus maculatus* | Brazil | Serra Negra do Norte | NA | 152 | ATL | (Fecchio *et al*., 2017) |
| KU562243 | -9,15 | -64,617 | *Haemoproteus* sp. | *Cercomacra cinerascens* | Brazil | Madeira River | NA | 152 | AMS | (Fecchio *et al*., 2017) |
| KU562190 | -15,53 | -47,55 | *Haemoproteus* sp. | *Nystalus maculatus* | Brazil | Planaltina | NA | 152 | CSA | (Fecchio *et al*., 2017) |
| KU562189 | -15,53 | -47,55 | *Haemoproteus* sp. | *Suiriri suiriri* | Brazil | Planaltina | NA | 153 | CSA | (Fecchio *et al*., 2017) |
| KU562202 | -15,53 | -47,55 | *Haemoproteus* sp. | *Neothraupis fasciata* | Brazil | Planaltina | NA | 154 | CSA | (Fecchio *et al*., 2017) |
| DQ241558 | 4,86 | -58,93 | *Haemoproteus* sp. | *Columbina passerina* | Guyana | NA | 51 | 155 | AMN | (Durrant *et al*., 2006) |
| DQ241558 | 4,86 | -58,93 | *Haemoproteus* sp. | *Columbina passerina* | Guyana | NA | 51 | 155 | AMN | (Durrant *et al*., 2006) |
| DQ241558 | 4,86 | -58,93 | *Haemoproteus* sp. | *Columbina passerina* | Guyana | NA | 51 | 155 | AMN | (Durrant *et al*., 2006) |
| DQ241558 | 4,86 | -58,93 | *Haemoproteus* sp. | *Columbina passerina* | Guyana | NA | 51 | 155 | AMN | (Durrant *et al*., 2006) |
| KU562226 | -3,7 | -46,75 | *Haemoproteus* sp. | *Columbina passerina* | Brazil | Gurupi | NA | 155 | AMS | (Fecchio *et al*., 2017) |
| KU562227 | -3,7 | -46,75 | *Haemoproteus paramultipigmentatus* | *Columbina passerina* | Brazil | Gurupi | NA | 155 | AMS | (Fecchio *et al*., 2017) |
| KU562228 | -3,7 | -46,75 | *Haemoproteus paramultipigmentatus* | *Columbina passerina* | Brazil | Gurupi | NA | 155 | AMS | (Fecchio *et al*., 2017) |
| KU562229 | -3,7 | -46,75 | *Haemoproteus paramultipigmentatus* | *Thamnophilus doliatus* | Brazil | Gurupi | NA | 155 | AMS | (Fecchio *et al*., 2017) |
| KU562230 | -3,7 | -46,75 | *Haemoproteus paramultipigmentatus* | *Columbina passerina* | Brazil | Gurupi | NA | 155 | AMS | (Fecchio *et al*., 2017) |
| KU562246 | -12,567 | -70,083 | *Haemoproteus* sp. | *Lathrotriccus euleri* | Peru | Manu | NA | 155 | CAN | (Fecchio *et al*., 2017) |
| KU562248 | -12,567 | -70,083 | *Haemoproteus* sp. | *Deconychura longicauda* | Peru | Manu | NA | 156 | CAN | (Fecchio *et al*., 2017) |
| KU562247 | -12,567 | -70,083 | *Haemoproteus* sp. | *Automolus infuscatus* | Peru | Manu | NA | 157 | CAN | (Fecchio *et al*., 2017) |
| DQ241556 | 4,86 | -58,93 | *Haemoproteus* sp. | *Geotrygon montana* | Guyana | NA | 49 | 158 | AMN | (Durrant *et al*., 2006) |
| KT373865 | -2,117 | -77,733 | *Haemoproteus* sp. | *Geotrygon montana* | Ecuador | Morona-Santiago Province, Wisui | GEOTRY01 | 158 | AMN | (Moens & Pérez-Tris, 2016) |
| KT373865 | -2,087 | -77,751 | *Haemoproteus* sp. | *Geotrygon montana* | Ecuador | Wisui reserve | NA | 158 | AMN | (Moens *et al*., 2017) |
| KU562129 | -5,217 | -56,917 | *Haemoproteus* sp. | *Geotrygon montana* | Brazil | Tapajόs River | NA | 158 | AMS | (Fecchio *et al*., 2017) |
| KU562130 | -5,1 | -56,43 | *Haemoproteus* sp. | *Geotrygon montana* | Brazil | Jamanxim River | NA | 158 | AMS | (Fecchio *et al*., 2017) |
| KU562131 | -4,983 | -62,13 | *Haemoproteus* sp. | *Geotrygon montana* | Brazil | Purus River | NA | 158 | AMN | (Fecchio *et al*., 2017) |
| KU562207 | -4,983 | -62,13 | *Haemoproteus* sp. | *Geotrygon montana* | Brazil | Purus River | NA | 158 | AMN | (Fecchio *et al*., 2017) |
| KU562244 | -12,567 | -70,083 | *Haemoproteus* sp. | *Thamnophilus schistaceus* | Peru | Manu | NA | 158 | CAN | (Fecchio *et al*., 2017) |
| KU562224 | -6,582 | -37,267 | *Haemoproteus* sp. | *Coryphospingus pileatus* | Brazil | Serra Negra do Norte | NA | 159 | ATL | (Fecchio *et al*., 2017) |
| KU562236 | -1,35 | -56,367 | *Haemoproteus* sp. | *Micrastur gilvicollis* | Brazil | Porto Trombetas | NA | 160 | AMN | (Fecchio *et al*., 2017) |
| KU562137 | -4,7 | -56,63 | *Haemoproteus* sp. | *Thamnomanes saturninus* | Brazil | Tapajόs River | NA | 161 | AMS | (Fecchio *et al*., 2017) |
| KU562121 | -5,217 | -56,917 | *Haemoproteus* sp. | *Hypocnemis striata* | Brazil | Tapajόs River | NA | 162 | AMS | (Fecchio *et al*., 2017) |
| KU562122 | -5,066 | -56,851 | *Haemoproteus* sp. | *Hypocnemoides maculicauda* | Brazil | Tapajόs River | NA | 162 | AMS | (Fecchio *et al*., 2017) |
| KU562123 | -4,7 | -56,63 | *Haemoproteus* sp. | *Phlegopsis nigromaculata* | Brazil | Tapajόs River | NA | 162 | AMS | (Fecchio *et al*., 2017) |
| KU562124 | -5,066 | -56,851 | *Haemoproteus* sp. | *Xenops minutus* | Brazil | Tapajόs River | NA | 162 | AMS | (Fecchio *et al*., 2017) |
| KU562125 | -5,066 | -56,851 | *Haemoproteus* sp. | *Dendrocincla fuliginosa* | Brazil | Tapajόs River | NA | 162 | AMS | (Fecchio *et al*., 2017) |
| KU562126 | -4,7 | -56,63 | *Haemoproteus* sp. | *Phlegopsis nigromaculata* | Brazil | Tapajόs River | NA | 162 | AMS | (Fecchio *et al*., 2017) |
| KU562127 | -5,066 | -56,851 | *Haemoproteus* sp. | *Tolmomyias flaviventris* | Brazil | Tapajόs River | NA | 162 | AMS | (Fecchio *et al*., 2017) |
| KU562128 | -5,217 | -56,917 | *Haemoproteus* sp. | *Sclerurus caudacutus* | Brazil | Tapajόs River | NA | 162 | AMS | (Fecchio *et al*., 2017) |
| KU562132 | -5,217 | -56,917 | *Haemoproteus* sp. | *Myrmotherula axillaris* | Brazil | Tapajόs River | NA | 162 | AMS | (Fecchio *et al*., 2017) |
| KU562133 | -4,7 | -56,63 | *Haemoproteus* sp. | *Automolus ochrolaemus* | Brazil | Tapajόs River | NA | 162 | AMS | (Fecchio *et al*., 2017) |
| KU562134 | -4,7 | -56,63 | *Haemoproteus* sp. | *Schiffornis turdina* | Brazil | Tapajόs River | NA | 162 | AMS | (Fecchio *et al*., 2017) |
| KU562119 | -5,066 | -56,851 | *Haemoproteus* sp. | *Cyanocompsa cyanoides* | Brazil | Tapajόs River | NA | 162 | AMS | (Fecchio *et al*., 2017) |
| KU562182 | -15,53 | -47,55 | *Haemoproteus* sp. | *Tangara cayana* | Brazil | Planaltina | NA | 163 | CSA | (Fecchio *et al*., 2017) |
| KU562158 | -4,5 | -56,283 | *Haemoproteus* sp. | *Xiphorhynchus elegans* | Brazil | Tapajόs River | NA | 164 | AMS | (Fecchio *et al*., 2017) |
| KU562161 | -4,5 | -56,283 | *Haemoproteus* sp. | *Hypocnemis striata* | Brazil | Tapajόs River | NA | 165 | AMS | (Fecchio *et al*., 2017) |
| KU562160 | -4,5 | -56,283 | *Haemoproteus* sp. | *Hypocnemis striata* | Brazil | Tapajόs River | NA | 166 | AMS | (Fecchio *et al*., 2017) |
| KU562164 | -4,5 | -56,267 | *Haemoproteus* sp. | *Cantorchilus leucotis* | Brazil | Tapajόs River | NA | 167 | AMS | (Fecchio *et al*., 2017) |
| KU562166 | -4,5 | -56,267 | *Haemoproteus* sp. | *Xiphorhynchus obsoletus* | Brazil | Tapajόs River | NA | 168 | AMS | (Fecchio *et al*., 2017) |
| KU562357 | -4,5 | -56,283 | *Plasmodium* sp. | *Isleria hauxwelli* | Brazil | Tapajόs River | NA | 169 | AMS | (Fecchio *et al*., 2017) |
| KU562358 | -3,7 | -46,75 | *Plasmodium* sp. | *Isleria hauxwelli* | Brazil | Gurupi | NA | 169 | AMS | (Fecchio *et al*., 2017) |
| KU562359 | -12,567 | -70,083 | *Plasmodium* sp. | *Pipra fasciicauda* | Peru | Manu | NA | 169 | CAN | (Fecchio *et al*., 2017) |
| KU562381 | -4,5 | -56,283 | *Plasmodium* sp. | *Thamnomanes saturninus* | Brazil | Tapajόs River | NA | 170 | AMS | (Fecchio *et al*., 2017) |
| KU562382 | -4,5 | -56,283 | *Plasmodium* sp. | *Myrmoborus myotherinus* | Brazil | Tapajόs River | NA | 170 | AMS | (Fecchio *et al*., 2017) |
| KU562383 | -4,5 | -56,283 | *Plasmodium* sp. | *Phlegopsis nigromaculata* | Brazil | Tapajόs River | NA | 170 | AMS | (Fecchio *et al*., 2017) |
| KU562384 | -4,5 | -56,283 | *Plasmodium* sp. | *Hypocnemis striata* | Brazil | Tapajόs River | NA | 170 | AMS | (Fecchio *et al*., 2017) |
| KU562385 | -4,5 | -56,267 | *Plasmodium* sp. | *Thamnophilus nigrocinereus* | Brazil | Tapajόs River | NA | 170 | AMS | (Fecchio *et al*., 2017) |
| KU562387 | -4,5 | -56,267 | *Plasmodium* sp. | *Cantorchilus leucotis* | Brazil | Tapajόs River | NA | 170 | AMS | (Fecchio *et al*., 2017) |
| KU562388 | -4,5 | -56,267 | *Plasmodium* sp. | *Turdus fumigatus* | Brazil | Tapajόs River | NA | 170 | AMS | (Fecchio *et al*., 2017) |
| KU562389 | -4,5 | -56,267 | *Plasmodium* sp. | *Thamnophilus nigrocinereus* | Brazil | Tapajόs River | NA | 170 | AMS | (Fecchio *et al*., 2017) |
| KU562390 | -12,567 | -70,083 | *Plasmodium* sp. | *Pipra fasciicauda* | Peru | Manu | NA | 170 | CAN | (Fecchio *et al*., 2017) |
| KU562506 | -4,5 | -56,283 | *Plasmodium* sp. | *Hypocnemis striata* | Brazil | Tapajόs River | NA | 170 | AMS | (Fecchio *et al*., 2017) |
| KU562512 | -4,5 | -56,267 | *Plasmodium* sp. | *Cantorchilus leucotis* | Brazil | Tapajόs River | NA | 170 | AMS | (Fecchio *et al*., 2017) |
| KU562386 | -4,5 | -56,267 | *Plasmodium* sp. | *Turdus fumigatus* | Brazil | Tapajόs River | NA | 170 | AMS | (Fecchio *et al*., 2017) |
| JX546135 | -23,65 | -46,617 | *Plasmodium* sp. | *Cygnus atratus* | Brazil | São Paulo Zoo | NA | 171 | ATL | (Chagas *et al*., 2017) |
| JX546135 | -23,65 | -46,617 | *Plasmodium* sp. | *Phoenicopterus chilensis* | Brazil | São Paulo Zoo | NA | 171 | ATL | (Chagas *et al*., 2017) |
| KU562365 | -4,7 | -56,53 | *Plasmodium* sp. | *Micrastur semitorquatus* | Brazil | Tapajόs River | NA | 171 | AMS | (Fecchio *et al*., 2017) |
| KU562275 | -5,067 | -56,85 | *Plasmodium* sp. | *Hypocnemis striata* | Brazil | Tapajόs River | NA | 172 | AMS | (Fecchio *et al*., 2017) |
| KU562297 | -5,1 | -56,43 | *Plasmodium* sp. | *Willisornis poecilinotus* | Brazil | Jamanxim River | NA | 173 | AMS | (Fecchio *et al*., 2017) |
| JF833046 | -0,829 | -90,982 | *Plasmodium* sp. | *Spheniscus mendiculus* | Ecuador | Galápagos Islands | GAPE75 | 174 | NAN | (Levin *et al*., 2011) |
| JF833047 | -0,829 | -90,982 | *Plasmodium* sp. | *Spheniscus mendiculus* | Ecuador | Galápagos Islands | GAPE78 | 174 | NAN | (Levin *et al*., 2011) |
| KC867667 | -0,639 | -90,337 | *Plasmodium* sp. | *Geospiza fortis* | Ecuador | Galápagos Islands - Santa Cruz Island | SPMEN03 | 174 | NAN | (Levin *et al*., 2013) |
| KC867668 | -0,829 | -90,982 | *Plasmodium* sp. | *Setophaga petechia* | Ecuador | Galápagos Islands | SETPET02 | 174 | NAN | (Levin *et al*., 2013) |
| KC867666 | -0,829 | -91,135 | *Plasmodium* sp. | *Setophaga petechia* | Ecuador | Galápagos Island -Isabela Island | SPMEN03 | 174 | NAN | (Levin *et al*., 2013) |
| JQ988310 | -6,104 | -78,341 | *Parahaemoproteus* sp. | *Coeligena torquata* | Peru | Amazonas | NA | 175 | CAN | (Galen and Witt, 2014) |
| JQ988295 | -6,104 | -78,341 | *Parahaemoproteus* sp. | *Coeligena coeligena* | Peru | Amazonas | NA | 176 | CAN | (Galen and Witt, 2014) |
| KC680680 | -0,633 | -76,133 | *Haemoproteus* sp. | *Glyphorynchus spirurus* | Ecuador | Tiputini Biodiversity Station, Orellana Province | H4 | 177 | AMN | (Svensson-Coelho *et al*. 2016) |
| KJ661326 | -0,077 | -78,755 | *Haemoproteus* sp. | *Phaethornis syrmatophorus* | Ecuador | Mindo | NA | 178 | NAN | (Harrigan *et al*., 2014) |
| KJ661328 | -0,077 | -78,755 | *Haemoproteus* sp. | *Phaethornis striigularis* | Ecuador | Mindo | NA | 178 | NAN | (Harrigan *et al*., 2014) |
| KJ661271 | -0,011 | -78,688 | *Plasmodium* sp. | *Mionectes striaticollis* | Ecuador | Bellavista | NA | 179 | NAN | (Harrigan *et al*., 2014) |
| KJ661285 | -2,887 | -79,428 | *Plasmodium* sp. | *Mionectes striaticollis* | Ecuador | Chuacha | NA | 179 | NAN | (Harrigan *et al*., 2014) |
| KJ661255 | -2,636 | -77,798 | *Plasmodium* sp. | *Eutoxeres aquila* | Ecuador | Maizal | NA | 180 | NAN | (Harrigan *et al*., 2014) |
| KJ661290 | -0,067 | -78,883 | *Plasmodium* sp. | *Myiothlypis fulvicauda* | Ecuador | La Joya | NA | 180 | NAN | (Harrigan *et al*., 2014) |
| KJ661300 | -1,477 | -78,16 | *Plasmodium* sp. | *Phaethornis* sp. | Ecuador | Cumandá | NA | 180 | NAN | (Harrigan *et al*., 2014) |
| KJ661315 | -2,887 | -79,427 | *Plasmodium* sp. | *Phaethornis yaruqui* | Ecuador | Chuacha | NA | 180 | NAN | (Harrigan *et al*., 2014) |
| KJ661327 | -0,077 | -78,755 | *Plasmodium* sp. | *Phaethornis syrmatophorus* | Ecuador | Mindo | NA | 180 | NAN | (Harrigan *et al*., 2014) |
| KJ661329 | -0,077 | -78,755 | *Plasmodium* sp. | *Phaethornis striigularis* | Ecuador | Mindo | NA | 180 | NAN | (Harrigan *et al*., 2014) |
| KJ661330 | -2,099 | -78,135 | *Plasmodium* sp. | *Phaethornis guy* | Ecuador | Nueva Alianza | NA | 180 | NAN | (Harrigan *et al*., 2014) |
| KJ661331 | -2,099 | -78,135 | *Plasmodium* sp. | *Phaethornis guy* | Ecuador | Nueva Alianza | NA | 180 | NAN | (Harrigan *et al*., 2014) |
| KJ661261 | -0,599 | -77,89 | *Plasmodium* sp. | *Mionectes striaticollis* | Ecuador | Cerro Bosco | NA | 180 | NAN | (Harrigan *et al*., 2014) |
| JQ988222 | -6,649 | -76,072 | *Parahaemoproteus* sp. | *Microcerculus marginatu* | Peru | San Martín | NA | 181 | CAN | (Galen and Witt, 2014) |
| KF767416 | -14,173 | -73,323 | *Haemoproteus* sp. | *Troglodytes aedon* | Peru | Apurímac | TROAED17 | 182 | CAN | (Galen and Witt, 2014) |
| KF537296 | 4,804 | -75,713 | *Haemoproteus witti* | *Eriocnemis derbyi* | Colombia | Pereira | TROAED20 | 183 | NAN | (González *et al*., 2015) |
| KT373862 | -2,117 | -77,733 | *Haemoproteus* sp. | *Xenopipo holochlora* | Ecuador | Morona-Santiago Province, Wisui | XENHOL01 | 183 | AMN | (Moens & Pérez-Tris, 2016) |
| KT373862 | -2,087 | -77,751 | *Parahaemoproteus* sp. | *Xenopipo holochlora* | Ecuador | Wisui reserve | NA | 183 | AMN | (Moens *et al*., 2017) |
| KU562136 | -5,217 | -56,917 | *Haemoproteus* sp. | *Automolus rufipileatus* | Brazil | Tapajόs River | NA | 183 | AMS | (Fecchio *et al*., 2017) |
| KF537299 | 4,804 | -75,713 | *Haemoproteus witti* | *Eriocnemis cupreoventris* | Colombia | Pereira | TROAED20 | 183 | NAN | (González *et al*., 2015) |
| JF833061 | -0,829 | -90,982 | *Haemoproteus* sp. | *Sula nebouxii* | Ecuador | Galápagos Islands | NA | 184 | NAN | (Lee-Cruz *et al*., 2016) |
| JF833060 | -0,829 | -90,982 | *Haemoproteus* sp. | *Sula nebouxii* | Ecuador | Galápagos Islands | SE2M | 184 | NAN | (Levin *et al*., 2011) |
| JF833060 | -0,829 | -90,982 | *Haemoproteus* sp. | *Sula nebouxii* | Ecuador | Galápagos Islands | NA | 184 | NAN | (Lee-Cruz *et al*., 2016) |
| JF833061 | -0,829 | -90,982 | *Haemoproteus* sp. | *Sula nebouxii* | Ecuador | Galápagos Islands | SE11M | 184 | NAN | (Levin *et al*., 2011) |
| KF537325 | 4,804 | -75,713 | *Haemoproteus* sp. | *Vireo olivaceus* | Colombia | Pereira | TANIG01 | 185 | NAN | (González *et al*., 2015) |
| JF833065 | -0,829 | -90,982 | *Haemoproteus* sp. | *Creagrus furcatus* | Ecuador | Galápagos Islands | STG2 | 186 | NAN | (Levin *et al*., 2011) |
| HQ287550 | -10,249 | -48,324 | *Haemoproteus* sp. | *Galbula ruficauda* | Brazil | Palmas,Tocantins | Toc-26 | 187 | CSA | (Belo *et al*., 2011) |
| JQ314226 | NA | NA | *Haemosporida* sp. | *Cygnus columbianus* | North America Continental | NA | CYGNUS01 | 188 | NA | (Galen and Witt, 2014) |
| KJ527071 | -9,190 | -75,015 | *Haemoproteus* sp. | *Oxyura jamaicensis* | Peru | NA | NA | 188 | NAN | (Smith and Ramey 2015) |
| KJ527072 | -9,190 | -75,015 | *Haemoproteus* sp. | *Anas georgica* | Peru | NA | NA | 188 | NAN | (Smith and Ramey 2015) |
| KJ527070 | -9,190 | -75,015 | *Haemoproteus* sp. | *Anas cyanoptera* | Peru | NA | NA | 188 | NAN | (Smith and Ramey 2015) |
| KJ527077 | -9,190 | -75,015 | *Plasmodium* sp. | *Anas cyanoptera* | Peru | NA | NA | 189 | NAN | (Smith and Ramey 2015) |
| AY455656 | 16,235 | -61,488 | *Haemoproteus* sp. | *Margarops fuscus* | Antilles | NA | HF | 190* | LAN | (Fallon *et al*., 2005) |
| AY841000 | 16,235 | -61,488 | *Plasmodium* sp. | *Sin registro* | Antilles | NA | PH | 191* | LAN | (Fallon *et al*., 2005) |
| AY455659 | 16,235 | -61,488 | *Haemoproteus* sp. | *Dendroica adelaidae* | Antilles | NA | HI | 192* | LAN | (Fallon *et al*., 2005) |
| AY455657 | 16,235 | -61,488 | *Haemoproteus* sp. | *Margarops fuscus* | Antilles | NA | HG | 193* | LAN | (Fallon *et al*., 2005) |
| DQ241557 | 4,86 | -58,93 | *Haemoproteus* sp. | *Columbina talpacoti* | Guyana | NA | 50 | 194* | AMN | (Durrant *et al*., 2006) |
| GQ141576 | NA | NA | *Parahaemoproteus* sp. | *Dumetella carolinensis* | North America Continental | NA | MAFUS02 | 195 | NA | (Galen and Witt, 2014) |
| GQ141579 | NA | NA | *Parahaemoproteus* sp. | *Margarops fuscus* | Antilles | NA | MAFUS04 | 196 | NA | (Galen and Witt, 2014) |
| AF465565 | NA | NA | *Haemoproteus* sp. | *Margarops fuscus* | North America Continental | NA | MAFUS03 | 196 | NA | (Galen and Witt, 2014) |
| AY167246 | 16,742 | -62,187 | *Haemoproteus* sp. | *Coereba flaveola* | Antilles | Lesser Antilles - Montserrat Island | HU3 | 196 | LAN | (Fallon *et al*., 2005; Fallon *et al*., 2003) |
| GQ141597 | NA | NA | *Parahaemoproteus* sp. | *Dendroica adelaidae* | Antilles | NA | NA | 197 | NA | (Galen and Witt, 2014) |
| HM222461 | 37,09 | -95,712 | *Haemoproteus* sp. | *Loxigilla violacea* | United States of America | NA | NA | 198 | NA | (Ricklefs *et al*., 2017) |
| AF465569 | NA | NA | *Haemoproteus* sp. | *Loxigilla noctis* | Antilles | NA | LOXNOC01 | 198 | NA | (Galen and Witt, 2014) |
| AY167245 | 17,075 | -61,817 | *Haemoproteus* sp. | *Loxigilla noctis* | Antilles | Lesser Antilles - Antigua Island | HE | 198 | LAN | (Fallon *et al*., 2005; Fallon *et al*., 2003) |
| AY167245 | 17,075 | -61,817 | *Haemoproteus* sp. | *Tiaris bicolor* | Antilles | Lesser Antilles - Antigua Island | HE | 198 | LAN | (Fallon *et al*., 2005; Fallon *et al*., 2003) |
| AY167245 | 17,626 | -61,77 | *Haemoproteus* sp. | *Loxigilla noctis* | Antilles | Antillas menores - Barbuda Island | HE | 198 | LAN | (Fallon *et al*., 2005; Fallon *et al*., 2003) |
| AY167245 | 13,909 | -60,978 | *Haemoproteus* sp. | *Loxigilla noctis* | Antilles | Lesser Antilles - Saint Lucia Island | HE | 198 | LAN | (Fallon *et al*., 2005; Fallon *et al*., 2003) |
| AY167245 | 13,909 | -60,978 | *Haemoproteus* sp. | *Vireo altiloquus* | Antilles | Lesser Antilles - Saint Lucia Island | HE | 198 | LAN | (Fallon *et al*., 2005; Fallon *et al*., 2003) |
| AY167245 | 16,742 | -62,187 | *Haemoproteus* sp. | *Loxigilla noctis* | Antilles | Lesser Antilles - Montserrat Island | HE | 198 | LAN | (Fallon *et al*., 2005; Fallon *et al*., 2003) |
| AF465590 | NA | NA | *Haemoproteus* sp. | *Melanerpes erythrocephalus* | North America Continental | NA | MALERY01 | 199 | NA | (Galen and Witt, 2014) |
| HM222472 | NA | NA | *Haemoproteus* sp. | *Melanerpes striatus* | North America Continental y Antilles | NA | NA | 199 | NA | (Galen and Witt, 2014) |
| AF465582 | NA | NA | *Haemoproteus* sp. | *Piranga rubra* | North America Continental | NA | PIRUB01 | 200 | NA | (Galen and Witt, 2014) |
| GU252006 | NA | NA | *Parahaemoproteus* sp. | *Vireo olivaceus* | North America Continental | NA | NA | 201 | NA | (Galen and Witt, 2014) |
| AY167240 | 14,609 | -61,072 | *Haemoproteus* sp. | *Vireo altiloquus* | Antilles | Lesser Antilles - Martinique Island | HB | 201 | LAN | (Fallon *et al*., 2005; Fallon *et al*., 2003) |
| AY167240 | 17,626 | -61,77 | *Haemoproteus* sp. | *Vireo altiloquus* | Antilles | Antillas menores - Barbuda Island | HB | 201 | LAN | (Fallon *et al*., 2005; Fallon *et al*., 2003) |
| AY167240 | 13,909 | -60,978 | *Haemoproteus* sp. | *Vireo altiloquus* | Antilles | Lesser Antilles - Saint Lucia Island | HB | 201 | LAN | (Fallon *et al*., 2005; Fallon *et al*., 2003) |
| AY167240 | 15,439 | -61,346 | *Haemoproteus* sp. | *Vireo altiloquus* | Antilles | Lesser Antilles - Dominica Island | HB | 201 | LAN | (Fallon *et al*., 2005; Fallon *et al*., 2003) |
| AY167240 | 16,129 | -61,653 | *Haemoproteus* sp. | *Vireo altiloquus* | Antilles | Lesser Antilles - Guadeloupe Island | HB | 201 | LAN | (Fallon *et al*., 2005; Fallon *et al*., 2003) |
| AY167240 | 13,251 | -61,186 | *Haemoproteus* sp. | *Vireo altiloquus* | Antilles | Lesser Antilles - Saint Vincent Island | HB | 201 | LAN | (Fallon *et al*., 2005; Fallon *et al*., 2003) |
| AY817748 | 37,964 | -91,832 | *Haemoproteus* sp. | *Vireo olivaceus* | United States of America | Misuri | NA | 201 | NA | (Ricklefs *et al*., 2017) |
| GU252005 | NA | NA | *Parahaemoproteus* sp. | *Vireo olivaceus* | North America Continental | NA | NA | 202 | NA | (Galen and Witt, 2014) |
| AY099034 | 44,558 | -72,577 | *Haemoproteus* sp. | *Vireo olivaceus* | United States of America | Vermont | VIOLI01 | 202 | NA | (Galen and Witt, 2014) |
| JN792140 | 36,778 | -119,418 | *Haemoproteus* sp. | *Catharus ustulatus* | United States of America | California | NA | 202 | NA | (Galen and Witt, 2014) |
| GU251992 | NA | NA | *Parahaemoproteus* sp. | *Vireo crassirostris* | Antilles | NA | NA | 203 | NA | (Galen and Witt, 2014) |
| GU252009 | NA | NA | *Parahaemoproteus* sp. | *Vireo pallens* | North America Continental | NA | NA | 203 | NA | (Galen and Witt, 2014) |
| GU251991 | NA | NA | *Parahaemoproteus* sp. | *Falco sparverius* | North America Continental | NA | SALAUR01 | 204 | NA | (Galen and Witt, 2014) |
| AF465594 | NA | NA | *Haemoproteus* sp. | *Polyborus plancus* | North America Continental | NA | POLPLA01 | 204 | NA | (Galen and Witt, 2014) |
| DQ241555 | -32,522 | -55,765 | *Haemoproteus* sp. | *Saltator aurantiirostris* | Uruguay | NA | 48 | 204 | PAM | (Durrant *et al*., 2006) |
| EU627830 | 38,837 | -120,895 | *Haemoproteus* sp. | *Tyto alba* | United States of America | Northern California | BNOW03 | 204 | NA | (Galen and Witt, 2014) |
| AF465577 | NA | NA | *Haemoproteus* sp. | *Vireo griseus* | North America Continental y Antilles | NA | VIGRI02 | 205 | NA | (Galen and Witt, 2014) |
| GU256263 | NA | NA | *Parahaemoproteus* sp. | *Loxigilla portoricensis* | Antilles | NA | LOXPOR01 | 206 | NA | (Galen and Witt, 2014) |
| AF465579 | NA | NA | *Haemoproteus* sp. | *Coereba flaveola* | Antilles | NA | COFLA01 | 206 | NA | (Galen and Witt, 2014) |
| AF465579 | NA | NA | *Haemoproteus* sp. | *Loxigilla noctis* | Antilles | NA | COFLA01 | 206 | NA | (Galen and Witt, 2014) |
| AY167242 | 14,609 | -61,072 | *Haemoproteus* sp. | *Loxigilla noctis* | Antilles | Lesser Antilles - Martinique Island | HC | 206 | LAN | (Fallon *et al*., 2005; Fallon *et al*., 2003) |
| AY167242 | 17,075 | -61,817 | *Haemoproteus* sp. | *Coereba flaveola* | Antilles | Lesser Antilles - Antigua Island | HC | 206 | LAN | (Fallon *et al*., 2005; Fallon *et al*., 2003) |
| AY167242 | 17,626 | -61,77 | *Haemoproteus* sp. | *Coereba flaveola* | Antilles | Antillas menores - Barbuda Island | HC | 206 | LAN | (Fallon *et al*., 2005; Fallon *et al*., 2003) |
| AY167242 | 13,909 | -60,978 | *Haemoproteus* sp. | *Loxigilla noctis* | Antilles | Lesser Antilles - Saint Lucia Island | HC | 206 | LAN | (Fallon *et al*., 2005; Fallon *et al*., 2003) |
| AY167242 | 13,909 | -60,978 | *Haemoproteus* sp. | *Coereba flaveola* | Antilles | Lesser Antilles - Saint Lucia Island | HC | 206 | LAN | (Fallon *et al*., 2005; Fallon *et al*., 2003) |
| AY167242 | 13,909 | -60,978 | *Haemoproteus* sp. | *Tiaris bicolor* | Antilles | Lesser Antilles - Saint Lucia Island | HC | 206 | LAN | (Fallon *et al*., 2005; Fallon *et al*., 2003) |
| AY167242 | 13,909 | -60,978 | *Haemoproteus* sp. | *Vireo altiloquus* | Antilles | Lesser Antilles - Saint Lucia Island | HC | 206 | LAN | (Fallon *et al*., 2005; Fallon *et al*., 2003) |
| AY167242 | 16,742 | -62,187 | *Haemoproteus* sp. | *Loxigilla noctis* | Antilles | Lesser Antilles - Montserrat Island | HC | 206 | LAN | (Fallon *et al*., 2005; Fallon *et al*., 2003) |
| AY167242 | 16,742 | -62,187 | *Haemoproteus* sp. | *Coereba flaveola* | Antilles | Lesser Antilles - Montserrat Island | HC | 206 | LAN | (Fallon *et al*., 2005; Fallon *et al*., 2003) |
| AY167242 | 15,439 | -61,346 | *Haemoproteus* sp. | *Loxigilla noctis* | Antilles | Lesser Antilles - Dominica Island | HC | 206 | LAN | (Fallon *et al*., 2005; Fallon *et al*., 2003) |
| AY167242 | 13,194 | -59,543 | *Haemoproteus* sp. | *Loxigilla noctis* | Antilles | Lesser Antilles - Barbados | HC | 206 | LAN | (Fallon *et al*., 2005; Fallon *et al*., 2003) |
| AY167242 | 15,439 | -61,346 | *Haemoproteus* sp. | *Coereba flaveola* | Antilles | Lesser Antilles - Dominica Island | HC | 206 | LAN | (Fallon *et al*., 2005; Fallon *et al*., 2003) |
| AY167242 | 13,194 | -59,543 | *Haemoproteus* sp. | *Tiaris bicolor* | Antilles | Lesser Antilles - Barbados | HC | 206 | LAN | (Fallon *et al*., 2005; Fallon *et al*., 2003) |
| AY167242 | 15,439 | -61,346 | *Haemoproteus* sp. | *Vireo altiloquus* | Antilles | Lesser Antilles - Dominica Island | HC | 206 | LAN | (Fallon *et al*., 2005; Fallon *et al*., 2003) |
| AY167242 | 16,129 | -61,653 | *Haemoproteus* sp. | *Loxigilla noctis* | Antilles | Lesser Antilles - Guadeloupe Island | HC | 206 | LAN | (Fallon *et al*., 2005; Fallon *et al*., 2003) |
| AY167242 | 16,129 | -61,653 | *Haemoproteus* sp. | *Coereba flaveola* | Antilles | Lesser Antilles - Guadeloupe Island | HC | 206 | LAN | (Fallon *et al*., 2005; Fallon *et al*., 2003) |
| AY167242 | 13,251 | -61,186 | *Haemoproteus* sp. | *Loxigilla noctis* | Antilles | Lesser Antilles - Saint Vincent Island | HC | 206 | LAN | (Fallon *et al*., 2005; Fallon *et al*., 2003) |
| AY167242 | 13,251 | -61,186 | *Haemoproteus* sp. | *Coereba flaveola* | Antilles | Lesser Antilles - Saint Vincent Island | HC | 206 | LAN | (Fallon *et al*., 2005; Fallon *et al*., 2003) |
| GQ141606 | NA | NA | *Parahaemoproteus* sp. | *Buteo jamaicensis* | North America Continental | NA | BUTJAM12 | 206 | NA | (Galen and Witt, 2014) |
| HQ287540 | -10,249 | -48,324 | *Haemoproteus* sp. | *Elaenia chiriquensis* | Brazil | Palmas,Tocantins | Toc5 | 206 | CSA | (Belo *et al*., 2011) |
| JN819369 | 9,748 | -83,753 | *Haemoproteus* sp. | *Tangara icterocephala* | Costa Rica | NA | NA | 206 | CDH | (Galen and Witt, 2014) |
| JN819373 | 9,748 | -83,753 | *Haemoproteus* sp. | *Tangara icterocephala* | Costa Rica | NA | NA | 206 | CDH | (Galen and Witt, 2014) |
| JN819370 | 9,748 | -83,753 | *Haemoproteus* sp. | *Tangara icterocephala* | Costa Rica | NA | NA | 207 | CDH | (Galen and Witt, 2014) |
| KF537282 | 4,804 | -75,713 | *Haemoproteus* sp. | *Hemispingus superciliaris* | Colombia | Pereira | TANIG01 | 207 | NAN | (González *et al*., 2015) |
| KF537285 | 4,804 | -75,713 | *Haemoproteus coatneyi* | *Tangara vassorii* | Colombia | Pereira | TANIG01 | 207 | NAN | (González *et al*., 2015) |
| KF537295 | 4,804 | -75,713 | *Haemoproteus coatneyi* | *Tangara nigroviridis* | Colombia | Pereira | TANIG01 | 207 | NAN | (González *et al*., 2015) |
| EU627829 | 38,837 | -120,895 | *Haemoproteus* sp. | *Tyto alba* | United States of America | Northern California | BNOW01 | 208 | NA | (Galen and Witt, 2014) |
| JX501823 | -15,53 | -47,55 | *Haemoproteus* sp. | *Volatinia jacarina* | Brazil | Águas Emendadas Station Ecologic, Distrito Federal | NA | 209 | CSA | (Ricklefs *et al*., 2017) |
| KC680685 | -0,633 | -76,133 | *Haemoproteus* sp. | *Glyphorynchus spirurus* | Ecuador | Tiputini Biodiversity Station, Orellana Province | H18 | 210 | AMN | (Svensson-Coelho *et al*. 2016) |
| JX501907 | -15,53 | -47,55 | *Haemoproteus* sp. | *Nystalus chacuru* | Brazil | Águas Emendadas Station Ecologic, Distrito Federal | NA | 210 | CSA | (Ricklefs *et al*., 2017) |
| KC680694 | -0,633 | -76,133 | *Plasmodium* sp. | *Automolus infuscatus* | Ecuador | Tiputini Biodiversity Station, Orellana Province | P36 | 211 | AMN | (Svensson-Coelho *et al*. 2016) |
| HQ287556 | -10,249 | -48,324 | *Plasmodium* sp. | *Volatinia jacarina* | Brazil | Palmas,Tocantins | Toc-24 | 212 | CSA | (Belo *et al*., 2011) |
| KC680706 | -0,633 | -76,133 | *Plasmodium* sp. | *Myrmelastes humaythae* | Ecuador | Tiputini Biodiversity Station, Orellana Province | NA | 212 | AMN | (Ricklefs *et al*., 2017) |
| KC680708 | -0,633 | -76,133 | *Plasmodium* sp. | *Thamnomanes ardesiacus* | Ecuador | Tiputini Biodiversity Station, Orellana Province | P12L | 212 | AMN | (Svensson-Coelho *et al*. 2016) |
| KC680702 | -0,633 | -76,133 | *Plasmodium* sp. | *Thamnophilus schistaceus* | Ecuador | Tiputini Biodiversity Station, Orellana Province | P25L | 212 | AMN | (Svensson-Coelho *et al*. 2016) |
| KC680716 | -0,633 | -76,133 | *Plasmodium* sp. | *Pipra filicauda* | Ecuador | Tiputini Biodiversity Station, Orellana Province | NA | 213 | AMN | (Ricklefs *et al*., 2017) |
| KC680716 | -0,633 | -76,133 | *Plasmodium* sp. | *Pipra filicauda* | Ecuador | Tiputini Biodiversity Station, Orellana Province | P20 | 213 | AMN | (Svensson-Coelho *et al*. 2016) |
| KC680718 | -0,633 | -76,133 | *Haemoproteus* sp. | *Glyphorynchus spirurus* | Ecuador | Tiputini Biodiversity Station, Orellana Province | NA | 214 | AMN | (Ricklefs *et al*., 2017) |
| KC680718 | -0,633 | -76,133 | *Haemoproteus* sp. | *Glyphorynchus spirurus* | Ecuador | Tiputini Biodiversity Station, Orellana Province | H13 | 214 | AMN | (Svensson-Coelho *et al*. 2016) |
| KC680665 | -0,633 | -76,133 | *Plasmodium* sp. | *Willisornis poecilinotus* | Ecuador | Tiputini Biodiversity Station, Orellana Province | NA | 215 | AMN | (Ricklefs *et al*., 2017) |
| KC680665 | -0,633 | -76,133 | *Plasmodium* sp. | *Willisornis poecilinotus* | Ecuador | Tiputini Biodiversity Station, Orellana Province | P29 | 215 | AMN | (Svensson-Coelho *et al*. 2016) |
| HQ287552 | -10,249 | -48,324 | *Plasmodium* sp. | *Monasa nigrifrons* | Brazil | Palmas,Tocantins | Toc-14 | 216 | CSA | (Belo *et al*., 2011) |
| KC680657 | -0,633 | -76,133 | *Plasmodium* sp. | *Formicarius colma* | Ecuador | Tiputini Biodiversity Station, Orellana Province | P4L | 216 | AMN | (Svensson-Coelho *et al*. 2016) |
| KC680670 | -0,633 | -76,133 | *Plasmodium* sp. | *Formicarius colma* | Ecuador | Tiputini Biodiversity Station, Orellana Province | NA | 216 | AMN | (Ricklefs *et al*., 2017) |
| KC680712 | -0,633 | -76,133 | *Plasmodium* sp. | *Hafferia fortis* | Ecuador | Tiputini Biodiversity Station, Orellana Province | P27 | 217 | AMN | (Svensson-Coelho *et al*. 2016) |
| KC680666 | -0,633 | -76,133 | *Haemoproteus* sp. | *Automolus infuscatus* | Ecuador | Tiputini Biodiversity Station, Orellana Province | H17L | 218 | AMN | (Svensson-Coelho *et al*. 2016) |
| KC680677 | -0,633 | -76,133 | *Haemoproteus* sp. | *Xiphorhynchus ocellatus* | Ecuador | Tiputini Biodiversity Station, Orellana Province | NA | 218 | AMN | (Ricklefs *et al*., 2017) |
| KC680720 | -0,633 | -76,133 | *Plasmodium* sp. | *Myiobius barbatus* | Ecuador | Tiputini Biodiversity Station, Orellana Province | P9 | 219 | AMN | (Svensson-Coelho *et al*. 2016) |
| DQ241541 | 4,86 | -58,93 | *Haemoproteus* sp. | *Psarocolius viridis* | Guyana | NA | PSAVIR02 | 220 | AMN | (Galen and Witt, 2014; Durrant *et al*., 2006) |
| DQ241514 | -32,522 | -55,765 | *Plasmodium* sp. | *Sturnella superciliaris* | Uruguay | NA | 7 | 221 | PAM | (Durrant *et al*., 2006) |
| DQ241514 | -32,522 | -55,765 | *Plasmodium* sp. | *Sturnella superciliaris* | Uruguay | NA | 7 | 221 | PAM | (Durrant *et al*., 2006) |
| DQ241542 | 4,86 | -58,93 | *Haemoproteus* sp. | *Lamprospiza melanoleuca* | Guyana | NA | LAMMEL01 | 222 | AMN | (Galen and Witt, 2014; Durrant *et al*., 2006) |
| DQ241544 | -32,522 | -55,765 | *Haemoproteus* sp. | *Molothrus badius* | Uruguay | NA | MOLBAD01 | 223 | PAM | (Galen and Witt, 2014; Durrant *et al*., 2006) |
| DQ241544 | -32,522 | -55,765 | *Haemoproteus* sp. | *Pardirallus sanguinolentus* | Uruguay | NA | MOLBAD01 | 223 | PAM | (Galen and Witt, 2014; Durrant *et al*., 2006) |
| DQ241546 | -32,522 | -55,765 | *Haemoproteus* sp. | *Icterus cayanensis* | Uruguay | NA | ICTCAY02 | 224 | PAM | (Galen and Witt, 2014; Durrant *et al*., 2006) |
| DQ241543 | 4,86 | -58,93 | *Haemoproteus* sp. | *Leptotila rufaxilla* | Guyana | NA | 36 | 224 | AMN | (Durrant *et al*., 2006) |
| HQ287547 | -10,249 | -48,324 | *Haemoproteus* sp. | *Vireo olivaceus* | Brazil | Palmas,Tocantins | Toc-22 | 225 | CSA | (Belo *et al*., 2011) |
| KC680688 | -0,633 | -76,133 | *Plasmodium* sp. | *Philydor pyrrhodes* | Ecuador | Tiputini Biodiversity Station, Orellana Province | P19 | 226 | AMN | (Svensson-Coelho *et al*. 2016) |
| KC680707 | -0,633 | -76,133 | *Plasmodium* sp. | *Myrmotherula axillaris* | Ecuador | Tiputini Biodiversity Station, Orellana Province | P10 | 227 | AMN | (Svensson-Coelho *et al*. 2016) |
| KC680659 | -0,633 | -76,133 | *Plasmodium* sp. | *Thamnophilus schistaceus* | Ecuador | Tiputini Biodiversity Station, Orellana Province | P42 | 227 | AMN | (Svensson-Coelho *et al*. 2016) |
| KC680671 | -0,633 | -76,133 | *Plasmodium* sp. | *Lepidothrix coronata* | Ecuador | Tiputini Biodiversity Station, Orellana Province | P16 | 228 | AMN | (Svensson-Coelho *et al*. 2016) |
| KJ644778 | 4,638 | -74,084 | *Haemoproteus columbae* | *Columba livia* | Colombia | Bogotá, Universidad Nacional de Colombia | NA | 229 | NAN | (Coral *et al*., 2015) |
| KU131583 | -23,65 | -46,617 | *Haemoproteus columbae* | *Columba livia* | Brazil | São Paulo Zoo | NA | 229 | ATL | (Chagas *et al*., 2016) |
| KU131585 | -23,65 | -46,617 | *Haemoproteus columbae* | *Columba livia* | Brazil | São Paulo Zoo | NA | 229 | ATL | (Chagas *et al*., 2016) |
| KC680690 | -0,633 | -76,133 | *Plasmodium* sp. | *Chamaeza nobilis* | Ecuador | Tiputini Biodiversity Station, Orellana Province | P5L | 230 | AMN | (Svensson-Coelho *et al*. 2016) |
| KX171628 | -23,65 | -46,617 | *Plasmodium* sp. | *Ramphastos vitellinus* | Brazil | São Paulo Zoo | NA | 231 | ATL | (Chagas *et al*., 2017) |
| KX171628 | -23,65 | -46,617 | *Plasmodium* sp. | *Ramphastos toco* | Brazil | São Paulo Zoo | NA | 231 | ATL | (Chagas *et al*., 2017) |
| KC789821 | 37,09 | -95,712 | *Plasmodium* sp. | *Turdus migratorius* | United States of America | NA | NA | 232 | NA | (Ricklefs *et al*., 2017) |
| KX171627 | -23,65 | -46,617 | *Parahaemoproteus* sp. | *Penelope obscura* | Brazil | São Paulo Zoo | NA | 233 | ATL | (Chagas *et al*., 2017) |
| KF537326 | 4,804 | -75,713 | *Haemoproteus coatneyi* | *Piranga olivacea* | Colombia | Pereira | PIOLI03 | 234 | NAN | (González *et al*., 2015) |
| MF990711 | 5,349 | -67,861 | *Haemoproteus* sp. | *Coereba flaveola* | Colombia | Vichada,Corregimiento Santa Rita, NNP El Tuparro | NA | 235 | NSA | In this studio |
| KJ175078 | 5,683 | -71,083 | *Haemoproteus macrovacuolatus* | *Dendrocygna autumnalis* | Colombia | Casanare, Paz de Ariporo | NA | 236 | NSA | (Matta *et al*., 2014) |
| KJ469133 | -1,817 | -65,7 | *Haemoproteus* sp. | *Rynchops niger* | Brazil | Amazonas | NA | 236 | AMN | (Roos *et al*. 2015) |
| KJ592828 | 5,758 | -71,573 | *Haemoproteus macrovacuolatus* | *Dendrocygna autumnalis* | Colombia | Casanare | CA1014 | 236 | NSA | (Bensch *et al*., 2009) |
| KJ592828 | 5,683 | -71,083 | *Haemoproteus macrovacuolatus* | *Dendrocygna autumnalis* | Colombia | Casanare, Paz de Ariporo | NA | 236 | NSA | (Matta *et al*., 2014) |
| KJ175078 | 5,758 | -71,573 | *Haemoproteus macrovacuolatus* | *Dendrocygna autumnalis* | Colombia | Casanare | CA1013 | 236 | NSA | (Bensch *et al*., 2009) |
| JN819389 | 9,748 | -83,753 | *Haemoproteus* sp. | *Tangara icterocephala* | Costa Rica | NA | STTA17H | 237 | CDH | (Benson *et al*., 2011) |
| JN819389 | -12,217 | -76,985 | *Haemoproteus* sp. | *Sicalis luteola* | Peru | Pantanos de Villa wetland Reserve | STTA17H | 237 | STP | (Marzal *et al*., 2015) |
| JN819389 | -9,916 | -76,233 | *Haemoproteus* sp. | *Thraupis episcopus* | Peru | Huánuco | STTA17H | 237 | CAN | (Marzal *et al*., 2015) |
| KJ661310 | 0,362 | -79,716 | *Haemoproteus* sp. | *Euphonia xanthogaster* | Ecuador | Bilsa | NA | 237 | EPC | (Harrigan *et al*., 2014) |
| KM065797 | 37,09 | -95,712 | *Haemoproteus* sp. | *Pipilo erythrophthalmus* | United States of America | NA | NA | 238 | NA | (Ricklefs *et al*., 2017) |
| HQ287541 | -10,249 | -48,324 | *Haemoproteus* sp. | *Turdus leucomelas* | Brazil | Palmas,Tocantins | Toc-7 | 239 | CSA | (Belo *et al*., 2011) |
| KF537328 | 4,804 | -75,713 | *Plasmodium* sp. | *Catharus ustulatus* | Colombia | Pereira | CATUST21 | 240 | NAN | (González *et al*., 2015) |
| KF482344 | -9,916 | -76,233 | *Haemoproteus* sp. | *Serpophaga cinerea* | Peru | Huánuco | SERCIN01 | 241 | CAN | (Marzal *et al*., 2015) |
| KC867673 | 44,451 | -73,122 | *Plasmodium* sp. | *Dolichonyx oryzivorus* | United States of America | Vermont - northwestern Vermont | RWB01 | 242 | NA | (Levin *et al*., 2013) |
| JF833051 | -0,829 | -90,982 | *Haemoproteus multipigmentatus* | *Zenaida galapagoensis* | Ecuador | Galápagos Islands | HMULTIPIGMENTATUS1 | 243 | NAN | (Levin *et al*., 2011) |
| JF833054 | -0,829 | -90,982 | *Haemoproteus multipigmentatus* | *Zenaida galapagoensis* | Ecuador | Galápagos Islands | HMULTIPIGMENTATUS4 | 243 | NAN | (Levin *et al*., 2011) |
| JF833055 | -1,831 | -78,183 | *Haemoproteus multipigmentatus* | *Zenaida galapagoensis* | Ecuador | Tiputini Biodiversity Station, Orellana Province | HMULTIPIGMENTATUS5 | 243 | AMN | (Levin *et al*., 2011) |
| JF833056 | -1,831 | -78,183 | *Haemoproteus multipigmentatus* | *Zenaida galapagoensis* | Ecuador | Tiputini Biodiversity Station, Orellana Province | HMULTIPIGMENTATUS6 | 243 | AMN | (Levin *et al*., 2011) |
| JF833059 | -0,829 | -90,982 | *Haemoproteus* sp. | *Sula granti* | Ecuador | Galápagos Islands | NZB9 | 243 | NAN | (Levin *et al*., 2011) |
| JF833059 | -0,829 | -90,982 | *Haemoproteus* sp. | *Creagrus furcatus* | Ecuador | Galápagos Islands | NZB9 | 243 | NAN | (Levin *et al*., 2011) |
| JF833066 | -0,829 | -90,982 | *Haemoproteus* sp. | *Creagrus furcatus* | Ecuador | Galápagos Islands | STG14 | 243 | NAN | (Levin *et al*., 2011) |
| KC867665 | -0,829 | -90,982 | *Plasmodium* sp. | *Setophaga petechia* | Ecuador | Galápagos Islands | SETPET01 | 244 | NAN | (Levin *et al*., 2013) |
| KT373868 | -2,117 | -77,733 | *Plasmodium* sp. | *Gymnopithys leucaspis* | Ecuador | Morona-Santiago Province, Wisui | MYRMAX01 | 245 | AMN | (Moens & Pérez-Tris, 2016) |
| KT373868 | -2,087 | -77,751 | *Plasmodium* sp. | *Myrmotherula axillaris* | Ecuador | Wisui reserve | NA | 245 | AMN | (Moens *et al*., 2017) |
| KU562285 | -4,7 | -56,53 | *Plasmodium* sp. | *Rhegmatorhina berlepschi* | Brazil | Tapajόs River | NA | 245 | AMS | (Fecchio *et al*., 2017) |
| KU562286 | -0,4 | -64,8 | *Plasmodium* sp. | *Myrmotherula axillaris* | Brazil | Negro River | NA | 245 | AMN | (Fecchio *et al*., 2017) |
| KU562287 | -4,983 | -62,13 | *Plasmodium* sp. | *Phlegopsis nigromaculata* | Brazil | Purus River | NA | 245 | AMN | (Fecchio *et al*., 2017) |
| KU562288 | -4,983 | -62,13 | *Plasmodium* sp. | *Isleria hauxwelli* | Brazil | Purus River | NA | 245 | AMN | (Fecchio *et al*., 2017) |
| KU562289 | -4,983 | -62,13 | *Plasmodium* sp. | *Gymnopithys salvini* | Brazil | Purus River | NA | 245 | AMN | (Fecchio *et al*., 2017) |
| KU562290 | -4,983 | -62,13 | *Plasmodium* sp. | *Hafferia fortis* | Brazil | Purus River | NA | 245 | AMN | (Fecchio *et al*., 2017) |
| KU562291 | -4,983 | -62,13 | *Plasmodium* sp. | *Myrmelastes humaythae* | Brazil | Purus River | NA | 245 | AMN | (Fecchio *et al*., 2017) |
| KU562292 | -4,983 | -62,13 | *Plasmodium* sp. | *Myrmotherula axillaris* | Brazil | Purus River | NA | 245 | AMN | (Fecchio *et al*., 2017) |
| KU562293 | -1,35 | -56,367 | *Plasmodium* sp. | *Gymnopithys rufigula* | Brazil | Porto Trombetas | NA | 245 | AMN | (Fecchio *et al*., 2017) |
| KU562294 | -1,35 | -56,367 | *Plasmodium* sp. | *Gymnopithys rufigula* | Brazil | Porto Trombetas | NA | 245 | AMN | (Fecchio *et al*., 2017) |
| KU562295 | -1,35 | -56,367 | *Plasmodium* sp. | *Gymnopithys rufigula* | Brazil | Porto Trombetas | NA | 245 | AMN | (Fecchio *et al*., 2017) |
| KU562296 | -8,8 | -64,083 | *Plasmodium* sp. | *Gymnopithys salvini* | Brazil | Madeira River | NA | 245 | AMS | (Fecchio *et al*., 2017) |
| KU562347 | -5,1 | -56,43 | *Plasmodium* sp. | *Phlegopsis nigromaculata* | Brazil | Jamanxim River | NA | 245 | AMS | (Fecchio *et al*., 2017) |
| KU562361 | -4,5 | -56,283 | *Plasmodium* sp. | *Thamnomanes saturninus* | Brazil | Tapajόs River | NA | 245 | AMS | (Fecchio *et al*., 2017) |
| KU562362 | -4,5 | -56,283 | *Plasmodium* sp. | *Myrmornis torquata* | Brazil | Tapajόs River | NA | 245 | AMS | (Fecchio *et al*., 2017) |
| KU562363 | -4,683 | -56,63 | *Plasmodium* sp. | *Hylophylax naevius* | Brazil | Tapajόs River | NA | 245 | AMS | (Fecchio *et al*., 2017) |
| KU562364 | -4,683 | -56,63 | *Plasmodium* sp. | *Myrmotherula hauxwelli* | Brazil | Tapajόs River | NA | 245 | AMS | (Fecchio *et al*., 2017) |
| KU562371 | -5,217 | -56,917 | *Plasmodium* sp. | *Hypocnemis striata* | Brazil | Tapajόs River | NA | 245 | AMS | (Fecchio *et al*., 2017) |
| KU562373 | -5,217 | -56,917 | *Plasmodium* sp. | *Willisornis poecilinotus* | Brazil | Tapajόs River | NA | 245 | AMS | (Fecchio *et al*., 2017) |
| KU562374 | -5,217 | -56,917 | *Plasmodium* sp. | *Phlegopsis nigromaculata* | Brazil | Tapajόs River | NA | 245 | AMS | (Fecchio *et al*., 2017) |
| KU562391 | -4,5 | -56,283 | *Plasmodium* sp. | *Myrmoborus myotherinus* | Brazil | Tapajόs River | NA | 245 | AMS | (Fecchio *et al*., 2017) |
| KU562392 | -4,683 | -56,63 | *Plasmodium* sp. | *Hypocnemis striata* | Brazil | Tapajόs River | NA | 245 | AMS | (Fecchio *et al*., 2017) |
| KU562393 | -4,683 | -56,63 | *Plasmodium* sp. | *Myrmoborus myotherinus* | Brazil | Tapajόs River | NA | 245 | AMS | (Fecchio *et al*., 2017) |
| KU562394 | -4,683 | -56,63 | *Plasmodium* sp. | *Myrmelastes humaythae* | Brazil | Tapajόs River | NA | 245 | AMS | (Fecchio *et al*., 2017) |
| KU562502 | -4,5 | -56,283 | *Plasmodium* sp. | *Dichrozona cincta* | Brazil | Tapajόs River | NA | 245 | AMS | (Fecchio *et al*., 2017) |
| KT373868 | -2,117 | -77,733 | *Plasmodium* sp. | *Myrmotherula axillaris* | Ecuador | Morona-Santiago Province, Wisui | MYRMAX01 | 245 | AMN | (Moens & Pérez-Tris, 2016) |
| AY393806 | 60,128 | 18,643 | *Haemoproteus* sp. | *Carduelis spinus* | Sweden | NA | SISKIN1 | 246 | NA | (Galen and Witt, 2014) |
| AY393806 | 60,129 | 18,643 | *Haemoproteus* sp. | *Sylvia communis* | Sweden | NA | SISKIN1 | 246 | NA | (Galen and Witt, 2014) |
| JN792143 | 9,748 | -83,753 | *Haemoproteus* sp. | *Catharus ustulatus* | Costa Rica | NA | NA | 246 | CDH | (Galen and Witt, 2014) |
| MF077657 | 35,827 | -106,896 | *Haemoproteus* sp. | *Cyanocitta stelleri* | United States of America | Nuevo Mexico, Elk Springs | CYASTE05 | 247 | NA | (Marroquin-Flores *et al*., 2017) |
| MF077658 | 35,570 | -107,244 | *Plasmodium* sp. | *Aphelocoma woodhouseii* | United States of America | Nuevo Mexico, Mesa Chivato | TROAED24 | 248 | NA | (Marroquin-Flores *et al*., 2017) |
| MF077658 | 34,813 | -108,211 | *Plasmodium* sp. | *Thryomanes bewickii* | United States of America | Nuevo Mexico, El Malpais | TROAED24 | 248 | NA | (Marroquin-Flores *et al*., 2017) |
| JQ988462 | -6,649 | -76,072 | *Parahaemoproteus* sp. | *Bucco macrodactylus* | Peru | San Martín | H-T091 | 249 | CAN | (Galen and Witt, 2014) |
| JQ988446 | -11,983 | -74,933 | *Parahaemoproteus* sp. | *Heliangelus amethysticollis* | Peru | Junín | NA | 250 | CAN | (Galen and Witt, 2014) |
| MF077668 | 35,535 | -107,350 | *Haemoproteus* sp. | *Piranga ludoviciana* | United States of America | Nuevo Mexico, Mesa Chivato | PIRLUD01 | 251 | NA | (Marroquin-Flores *et al*., 2017) |
| JX021457 | -17,111 | -43,82 | *Plasmodium* sp. | *Thamnophilus ambiguus* | Brazil | Bocaiúva | THCAE01 | 252 | CSA | (Lacorte *et al*., 2013) |
| JX021457 | -22,959 | -44,041 | *Plasmodium* sp. | *Sakesphorus cristatus* | Brazil | Manga | THCAE01 | 252 | ATL | (Lacorte *et al*., 2013) |
| JX021457 | -19,993 | -43,848 | *Plasmodium* sp. | *Thamnophilus caerulescens* | Brazil | Nova Lima | THCAE01 | 252 | CSA | (Lacorte *et al*., 2013) |
| JX021457 | -3,944 | -73,607 | *Plasmodium* sp. | *Myrmotherula axillaris* | Peru | Allpahuayo Mishana National Reserve | NA | 252 | AMN | (Ricopa & Villa, 2016) |
| JX021457 | -3,944 | -73,607 | *Plasmodium* sp. | *Lepidotrix coronata* | Peru | Allpahuayo Mishana National Reserve | NA | 252 | AMN | (Ricopa & Villa, 2016) |
| JX021457 | -17,111 | -43,82 | *Plasmodium* sp. | *Sakesphorus cristatus* | Brazil | Bocaiúva | THCAE01 | 252 | CSA | (Lacorte *et al*., 2013) |
| MF077665 | 34,957 | -107,970 | *Plasmodium* sp. | *Baeolophus ridgwayi* | United States of America | Nuevo Mexico, El Malpais | MOLATE01 | 253 | NA | (Marroquin-Flores *et al*., 2017) |
| KF767421 | -12,029 | -76,65 | *Haemoproteus* sp. | *Troglodytes aedon* | Peru | Lima | TROAED12 | 254 | STP | (Galen and Witt, 2014) |
| KX130086 | 10,829 | -73,692 | *Parahaemoproteus* sp. | *Vireo leucophrys* | Colombia | Sierra Nevada de Santa Marta, San Lorenzo ridge | NA | 254 | NAN | (Gonzalez-Quevedo, Rivera-Gutierrez & Pabón, 2016) |
| MF077670 | 35,827 | -106,896 | *Haemoproteus* sp. | *Vireo plumbeus* | United States of America | Nuevo Mexico, Elk Springs | TROAED12 | 254 | NA | (Marroquin-Flores *et al*., 2017) |
| MF077670 | 34,837 | -108,215 | *Haemoproteus* sp. | *Vireo plumbeus* | United States of America | Nuevo Mexico, El Malpais | TROAED12 | 254 | NA | (Marroquin-Flores *et al*., 2017) |
| MF077670 | 35,533 | -107,348 | *Haemoproteus* sp. | *Vireo plumbeus* | United States of America | Nuevo Mexico, Mesa Chivato | TROAED12 | 254 | NA | (Marroquin-Flores *et al*., 2017) |
| KF767422 | -12,029 | -76,65 | *Haemoproteus* sp. | *Troglodytes aedon* | Peru | Lima | TROAED12 | 254 | STP | (Galen and Witt, 2014) |
| JQ988220 | -6,649 | -76,072 | *Parahaemoproteus* sp. | *Thamnomanes ardesiacus* | Peru | San Martín | NA | 255 | CAN | (Galen and Witt, 2014) |
| MF077648 | 34,836 | -108,223 | *Haemoproteus* sp. | *Tyrannus vociferans* | United States of America | Nuevo Mexico, El Malpais | TYRVOC01 | 256 | NA | (Marroquin-Flores *et al*., 2017) |
| JX029886 | -16,437 | -41,012 | *Plasmodium* sp. | *Turdus leucomelas* | Brazil | Jequitinhonha | TULEU08 | 257 | CSA | (Lacorte *et al*., 2013) |
| JX029879 | -17,0003 | -46,008 | *Plasmodium* sp. | *Gnorimopsar chopi* | Brazil | Brasilandia de Minas | CAOBS01 | 258 | CSA | (Lacorte *et al*., 2013) |
| JX029879 | -18,713 | -44,925 | *Plasmodium* sp. | *Camptostoma obsoletum* | Brazil | Felixlândia | CAOBS01 | 258 | CSA | (Lacorte *et al*., 2013) |
| JX029879 | -19,993 | -43,848 | *Plasmodium* sp. | *Thamnophilus caerulescens* | Brazil | Nova Lima | CAOBS01 | 258 | CSA | (Lacorte *et al*., 2013) |
| JX029879 | -17,0003 | -46,008 | *Plasmodium* sp. | *Camptostoma obsoletum* | Brazil | Brasilandia de Minas | CAOBS01 | 258 | CSA | (Lacorte *et al*., 2013) |
| KU562749 | -1,35 | -56,367 | *Plasmodium* sp. | *Glyphorynchus spirurus* | Brazil | Porto Trombetas | NA | 258 | AMN | (Fecchio *et al*., 2017) |
| KU562750 | -1,35 | -56,367 | *Plasmodium* sp. | *Glyphorynchus spirurus* | Brazil | Porto Trombetas | NA | 258 | AMN | (Fecchio *et al*., 2017) |
| KU562751 | -1,35 | -56,367 | *Plasmodium* sp. | *Glyphorynchus spirurus* | Brazil | Porto Trombetas | NA | 258 | AMN | (Fecchio *et al*., 2017) |
| KU562752 | -13,8 | -59,683 | *Plasmodium* sp. | *Turdus amaurochalinus* | Brazil | Comodoro | NA | 258 | CSA | (Fecchio *et al*., 2017) |
| KU562753 | -13,8 | -59,683 | *Plasmodium* sp. | *Ceratopipra rubrocapilla* | Brazil | Comodoro | NA | 258 | CSA | (Fecchio *et al*., 2017) |
| KU562754 | -13,8 | -59,683 | *Plasmodium* sp. | *Dendrocincla fuliginosa* | Brazil | Comodoro | NA | 258 | CSA | (Fecchio *et al*., 2017) |
| KU562755 | -13,8 | -59,683 | *Plasmodium* sp. | *Dendrocincla fuliginosa* | Brazil | Comodoro | NA | 258 | CSA | (Fecchio *et al*., 2017) |
| KU562756 | -13,8 | -59,683 | *Plasmodium* sp. | *Dendrocincla fuliginosa* | Brazil | Comodoro | NA | 258 | CSA | (Fecchio *et al*., 2017) |
| KU562757 | -13,8 | -59,683 | *Plasmodium* sp. | *Ceratopipra rubrocapilla* | Brazil | Comodoro | NA | 258 | CSA | (Fecchio *et al*., 2017) |
| KU562758 | -13,8 | -59,683 | *Plasmodium* sp. | *Myrmoborus myotherinus* | Brazil | Comodoro | NA | 258 | CSA | (Fecchio *et al*., 2017) |
| KU562759 | -13,8 | -59,683 | *Plasmodium* sp. | *Ceratopipra rubrocapilla* | Brazil | Comodoro | NA | 258 | CSA | (Fecchio *et al*., 2017) |
| KU562760 | -13,8 | -59,683 | *Plasmodium* sp. | *Glyphorynchus spirurus* | Brazil | Comodoro | NA | 258 | CSA | (Fecchio *et al*., 2017) |
| KU562761 | -13,8 | -59,683 | *Plasmodium* sp. | *Turdus amaurochalinus* | Brazil | Comodoro | NA | 258 | CSA | (Fecchio *et al*., 2017) |
| KU562762 | -13,8 | -59,683 | *Plasmodium* sp. | *Turdus amaurochalinus* | Brazil | Comodoro | NA | 258 | CSA | (Fecchio *et al*., 2017) |
| JX029918 | -19,82 | -40,276 | *Haemoproteus* sp. | *Troglodytes musculus* | Brazil | Aracruz | TRMUS01 | 259 | ATL | (Lacorte *et al*., 2013) |
| KU562138 | -4,683 | -56,63 | *Haemoproteus* sp. | *Tachyphonus cristatus* | Brazil | Tapajόs River | NA | 259 | AMS | (Fecchio *et al*., 2017) |
| KU562139 | -0,583 | -64,917 | *Haemoproteus* sp. | *Dendrocincla fuliginosa* | Brazil | Negro River | NA | 259 | AMN | (Fecchio *et al*., 2017) |
| KU562140 | -3,7 | -46,75 | *Haemoproteus* sp. | *Tachyphonus luctuosus* | Brazil | Gurupi | NA | 259 | AMS | (Fecchio *et al*., 2017) |
| KU562141 | -1,35 | -56,367 | *Haemoproteus* sp. | *Tachyphonus cristatus* | Brazil | Porto Trombetas | NA | 259 | AMN | (Fecchio *et al*., 2017) |
| JX029906 | -16,11 | -40,022 | *Haemoproteus* sp. | *Nemosia pileata* | Brazil | Salto da Divisa | NEPIL01 | 260 | CSA | (Lacorte *et al*., 2013) |
| JX029906 | -16,11 | -40,022 | *Haemoproteus* sp. | *Pheugopedius genibarbis* | Brazil | Salto da Divisa | NEPIL01 | 260 | CSA | (Lacorte *et al*., 2013) |
| AF254962 | 60,128 | 18,643 | *Plasmodium ashfordi* | *Acrocephalus arundinaceus* | Sweden | NA | GRW02 | 261 | NA | (Walther *et al*., 2014; Bensch *et al*., 2000) |
| JX029904 | -16,11 | -40,022 | *Haemoproteus* sp. | *Columbina talpacoti* | Brazil | Salto da Divisa | COTAL01 | 262 | CSA | (Lacorte *et al*., 2013) |
| JX029904 | -16,11 | -40,022 | *Haemoproteus* sp. | *Coccyzus melacoryphus* | Brazil | Salto da Divisa | COTAL01 | 262 | CSA | (Lacorte *et al*., 2013) |
| JX029904 | -22,959 | -44,041 | *Haemoproteus* sp. | *Columbina talpacoti* | Brazil | Manga | COTAL01 | 262 | ATL | (Lacorte *et al*., 2013) |
| KU562218 | -19,567 | -57,017 | *Haemoproteus multipigmentatus* | *Columbina talpacoti* | Brazil | Corumbá | NA | 262 | CSA | (Fecchio *et al*., 2017) |
| JX021477 | -17,111 | -43,82 | *Plasmodium* sp. | *Turdus albicollis* | Brazil | Bocaiúva | TUAMA01 | 263 | CSA | (Lacorte *et al*., 2013) |
| JX021477 | -17,111 | -43,82 | *Plasmodium* sp. | *Turdus amaurochalinus* | Brazil | Bocaiúva | TUAMA01 | 263 | CSA | (Lacorte *et al*., 2013) |
| JX021477 | -16,437 | -41,012 | *Plasmodium* sp. | *Turdus amaurochalinus* | Brazil | Jequitinhonha | TUAMA01 | 263 | CSA | (Lacorte *et al*., 2013) |
| JX021477 | -22,959 | -44,041 | *Plasmodium* sp. | *Turdus amaurochalinus* | Brazil | Manga | TUAMA01 | 263 | ATL | (Lacorte *et al*., 2013) |
| JX021477 | -16,11 | -40,022 | *Plasmodium* sp. | *Turdus amaurochalinus* | Brazil | Salto da Divisa | TUAMA01 | 263 | CSA | (Lacorte *et al*., 2013) |
| JX021477 | -3,944 | -73,607 | *Plasmodium* sp. | *Turdus ignobilis* | Peru | Allpahuayo Mishana National Reserve | NA | 263 | AMN | (Ricopa & Villa, 2016) |
| JX021477 | -19,82 | -40,276 | *Plasmodium* sp. | *Turdus leucomelas* | Brazil | Aracruz | TUAMA01 | 263 | ATL | (Lacorte *et al*., 2013) |
| KU562569 | -15,53 | -47,55 | *Plasmodium* sp. | *Turdus amaurochalinus* | Brazil | Planaltina | NA | 263 | CSA | (Fecchio *et al*., 2017) |
| KU562771 | -12,217 | -60,73 | *Plasmodium* sp. | *Turdus amaurochalinus* | Brazil | Chupinguaia | NA | 263 | AMS | (Fecchio *et al*., 2017) |
| KU562772 | -13,8 | -59,683 | *Plasmodium* sp. | *Turdus amaurochalinus* | Brazil | Comodoro | NA | 263 | CSA | (Fecchio *et al*., 2017) |
| MF077659 | 35,827 | -106,896 | *Haemoproteus* sp. | *Empidonax oberholseri* | United States of America | Nuevo Mexico, Elk Springs | EMPOBE02 | 264 | NA | (Marroquin-Flores *et al*., 2017) |
| MF077672 | 34,837 | -108,219 | *Haemoproteus* sp. | *Tyrannus vociferans* | United States of America | Nuevo Mexico, El Malpais | TYRVOC03 | 264 | NA | (Marroquin-Flores *et al*., 2017) |
| JX029867 | -17,111 | -43,82 | *Plasmodium* sp. | *Thamnophilus ambiguus* | Brazil | Bocaiúva | THAMB06 | 265 | CSA | (Lacorte *et al*., 2013) |
| KU562727 | -3,7 | -46,75 | *Plasmodium* sp. | *Thamnophilus aethiops* | Brazil | Gurupi | NA | 265 | AMS | (Fecchio *et al*., 2017) |
| JX029865 | -17,111 | -43,82 | *Plasmodium* sp. | *Thamnophilus ambiguus* | Brazil | Bocaiúva | THAMB05 | 266 | CSA | (Lacorte *et al*., 2013) |
| JX021485 | -19,993 | -43,848 | *Plasmodium* sp. | *Pyriglena leucoptera* | Brazil | Nova Lima | PYLEU02 | 267 | CSA | (Lacorte *et al*., 2013) |
| JX021459 | -16,11 | -40,022 | *Plasmodium* sp. | *Thamnophilus ambiguus* | Brazil | Salto da Divisa | THAMB03 | 267 | CSA | (Lacorte *et al*., 2013) |
| KU562730 | -3,7 | -46,75 | *Plasmodium* sp. | *Willisornis poecilinotus* | Brazil | Gurupi | NA | 267 | AMS | (Fecchio *et al*., 2017) |
| KU562731 | -3,7 | -46,75 | *Plasmodium* sp. | *Poecilotriccus fumifrons* | Brazil | Gurupi | NA | 267 | AMS | (Fecchio *et al*., 2017) |
| JX021492 | -19,993 | -43,848 | *Plasmodium* sp. | *Conopophaga lineata* | Brazil | Nova Lima | COLIN08 | 268 | CSA | (Lacorte *et al*., 2013) |
| KU562513 | -6,602 | -40,124 | *Plasmodium* sp. | *Coryphospingus pileatus* | Brazil | Aiuaba | NA | 269 | CSA | (Fecchio *et al*., 2017) |
| KU562514 | -6,602 | -40,124 | *Plasmodium* sp. | *Coryphospingus pileatus* | Brazil | Aiuaba | NA | 269 | CSA | (Fecchio *et al*., 2017) |
| KU562515 | -6,602 | -40,124 | *Plasmodium* sp. | *Veniliornis affinis* | Brazil | Aiuaba | NA | 269 | CSA | (Fecchio *et al*., 2017) |
| KU562516 | -6,602 | -40,124 | *Plasmodium* sp. | *Coryphospingus pileatus* | Brazil | Aiuaba | NA | 269 | CSA | (Fecchio *et al*., 2017) |
| KU562517 | -6,582 | -37,267 | *Plasmodium* sp. | *Coryphospingus pileatus* | Brazil | Serra Negra do Norte | NA | 269 | ATL | (Fecchio *et al*., 2017) |
| KU562518 | -6,582 | -37,267 | *Plasmodium* sp. | *Coryphospingus pileatus* | Brazil | Serra Negra do Norte | NA | 269 | ATL | (Fecchio *et al*., 2017) |
| KU562520 | -6,582 | -37,267 | *Plasmodium* sp. | *Coryphospingus pileatus* | Brazil | Serra Negra do Norte | NA | 269 | ATL | (Fecchio *et al*., 2017) |
| KU562521 | -6,582 | -37,267 | *Plasmodium* sp. | *Coryphospingus pileatus* | Brazil | Serra Negra do Norte | NA | 269 | ATL | (Fecchio *et al*., 2017) |
| KU562522 | -6,582 | -37,267 | *Plasmodium* sp. | *Coryphospingus pileatus* | Brazil | Serra Negra do Norte | NA | 269 | ATL | (Fecchio *et al*., 2017) |
| KU562523 | -6,582 | -37,267 | *Plasmodium* sp. | *Coryphospingus pileatus* | Brazil | Serra Negra do Norte | NA | 269 | ATL | (Fecchio *et al*., 2017) |
| KU562524 | -6,582 | -37,267 | *Plasmodium* sp. | *Coryphospingus pileatus* | Brazil | Serra Negra do Norte | NA | 269 | ATL | (Fecchio *et al*., 2017) |
| KU562525 | -6,582 | -37,267 | *Plasmodium* sp. | *Coryphospingus pileatus* | Brazil | Serra Negra do Norte | NA | 269 | ATL | (Fecchio *et al*., 2017) |
| KU562706 | -6,582 | -37,267 | *Plasmodium* sp. | *Coryphospingus pileatus* | Brazil | Serra Negra do Norte | NA | 269 | ATL | (Fecchio *et al*., 2017) |
| KU562710 | -6,582 | -37,267 | *Plasmodium* sp. | *Coryphospingus pileatus* | Brazil | Serra Negra do Norte | NA | 269 | ATL | (Fecchio *et al*., 2017) |
| KU562519 | -6,582 | -37,267 | *Plasmodium* sp. | *Veniliornis passerinus* | Brazil | Serra Negra do Norte | NA | 269 | ATL | (Fecchio *et al*., 2017) |
| KU562596 | -0,583 | -64,917 | *Plasmodium* sp. | *Willisornis poecilinotus* | Brazil | Negro River | NA | 270 | AMN | (Fecchio *et al*., 2017) |
| KU562600 | -0,583 | -64,917 | *Plasmodium* sp. | *Hylophylax naevius* | Brazil | Negro River | NA | 271 | AMN | (Fecchio *et al*., 2017) |
| KU562614 | -0,583 | -64,917 | *Plasmodium* sp. | *Gymnopithys leucaspis* | Brazil | Negro River | NA | 272 | AMN | (Fecchio *et al*., 2017) |
| KT373878 | -2,117 | -77,733 | *Plasmodium* sp. | *Thamnophilus aethiops* | Ecuador | Morona-Santiago Province, Wisui | THAMAE01 | 273 | AMN | (Moens & Pérez-Tris, 2016) |
| KT373878 | -2,117 | -77,733 | *Plasmodium* sp. | *Willisornis poecilinotus* | Ecuador | Morona-Santiago Province, Wisui | THAMAE01 | 273 | AMN | (Moens & Pérez-Tris, 2016) |
| KT373878 | -2,087 | -77,751 | *Plasmodium* sp. | *Thamnophilus aethiops* | Ecuador | Wisui reserve | NA | 273 | AMN | (Moens *et al*., 2017) |
| KU562298 | -5,1 | -56,43 | *Plasmodium* sp. | *Myrmotherula menetriesii* | Brazil | Jamanxim River | NA | 273 | AMS | (Fecchio *et al*., 2017) |
| KU562299 | -5,1 | -56,43 | *Plasmodium* sp. | *Myrmotherula axillaris* | Brazil | Jamanxim River | NA | 273 | AMS | (Fecchio *et al*., 2017) |
| KU562300 | -5,1 | -56,43 | *Plasmodium* sp. | *Rhegmatorhina gymnops* | Brazil | Jamanxim River | NA | 273 | AMS | (Fecchio *et al*., 2017) |
| KU562301 | -4,5 | -56,283 | *Plasmodium* sp. | *Phlegopsis nigromaculata* | Brazil | Tapajόs River | NA | 273 | AMS | (Fecchio *et al*., 2017) |
| KU562302 | -4,5 | -56,283 | *Plasmodium* sp. | *Thamnomanes saturninus* | Brazil | Tapajόs River | NA | 273 | AMS | (Fecchio *et al*., 2017) |
| KU562303 | -4,7 | -56,53 | *Plasmodium* sp. | *Myrmoborus myotherinus* | Brazil | Tapajόs River | NA | 273 | AMS | (Fecchio *et al*., 2017) |
| KU562304 | -4,683 | -56,63 | *Plasmodium* sp. | *Myrmoborus myotherinus* | Brazil | Tapajόs River | NA | 273 | AMS | (Fecchio *et al*., 2017) |
| KU562305 | -4,683 | -56,63 | *Plasmodium* sp. | *Willisornis poecilinotus* | Brazil | Tapajόs River | NA | 273 | AMS | (Fecchio *et al*., 2017) |
| KU562306 | -4,683 | -56,63 | *Plasmodium* sp. | *Hypocnemis striata* | Brazil | Tapajόs River | NA | 273 | AMS | (Fecchio *et al*., 2017) |
| KU562307 | -4,7 | -56,53 | *Plasmodium* sp. | *Isleria hauxwelli* | Brazil | Tapajόs River | NA | 273 | AMS | (Fecchio *et al*., 2017) |
| KU562308 | -4,683 | -56,63 | *Plasmodium* sp. | *Phlegopsis nigromaculata* | Brazil | Tapajόs River | NA | 273 | AMS | (Fecchio *et al*., 2017) |
| KU562309 | -4,5 | -56,283 | *Plasmodium* sp. | *Galbula cyanicollis* | Brazil | Tapajόs River | NA | 273 | AMS | (Fecchio *et al*., 2017) |
| KU562310 | -4,5 | -56,283 | *Plasmodium* sp. | *Hypocnemis striata* | Brazil | Tapajόs River | NA | 273 | AMS | (Fecchio *et al*., 2017) |
| KU562311 | -4,5 | -56,283 | *Plasmodium* sp. | *Dichrozona cincta* | Brazil | Tapajόs River | NA | 273 | AMS | (Fecchio *et al*., 2017) |
| KU562312 | -4,5 | -56,283 | *Plasmodium* sp. | *Thamnophilus nigrocinereus* | Brazil | Tapajόs River | NA | 273 | AMS | (Fecchio *et al*., 2017) |
| KU562313 | -5,717 | -63,2 | *Plasmodium* sp. | *Gymnopithys salvini* | Brazil | Purus River | NA | 273 | AMN | (Fecchio *et al*., 2017) |
| KU562314 | -5,717 | -63,2 | *Plasmodium* sp. | *Dichrozona cincta* | Brazil | Purus River | NA | 273 | AMN | (Fecchio *et al*., 2017) |
| KU562315 | -3,7 | -46,75 | *Plasmodium* sp. | *Isleria hauxwelli* | Brazil | Gurupi | NA | 273 | AMS | (Fecchio *et al*., 2017) |
| KU562316 | -3,7 | -46,75 | *Plasmodium* sp. | *Phlegopsis nigromaculata* | Brazil | Gurupi | NA | 273 | AMS | (Fecchio *et al*., 2017) |
| KU562317 | -9,1167 | -64,483 | *Plasmodium* sp. | *Dichrozona cincta* | Brazil | Madeira River | NA | 273 | AMS | (Fecchio *et al*., 2017) |
| KU562318 | -9,1167 | -64,483 | *Plasmodium* sp. | *Gymnopithys salvini* | Brazil | Madeira River | NA | 273 | AMS | (Fecchio *et al*., 2017) |
| KU562319 | -9,33 | -64,683 | *Plasmodium* sp. | *Rhegmatorhina hoffmannsi* | Brazil | Madeira River | NA | 273 | AMS | (Fecchio *et al*., 2017) |
| KU562320 | -9,133 | -64,633 | *Plasmodium* sp. | *Xiphorhynchus elegans* | Brazil | Madeira River | NA | 273 | AMS | (Fecchio *et al*., 2017) |
| KU562611 | -0,583 | -64,917 | *Plasmodium* sp. | *Glyphorynchus spirurus* | Brazil | Negro River | NA | 273 | AMN | (Fecchio *et al*., 2017) |
| KU562572 | -15,53 | -47,55 | *Plasmodium* sp. | *Neothraupis fasciata* | Brazil | Planaltina | NA | 274 | CSA | (Fecchio *et al*., 2017) |
| KU562573 | -15,53 | -47,55 | *Plasmodium* sp. | *Neothraupis fasciata* | Brazil | Planaltina | NA | 274 | CSA | (Fecchio *et al*., 2017) |
| KU562575 | -15,53 | -47,55 | *Plasmodium* sp. | *Neothraupis fasciata* | Brazil | Planaltina | NA | 274 | CSA | (Fecchio *et al*., 2017) |
| KU562571 | -15,53 | -47,55 | *Plasmodium* sp. | *Neothraupis fasciata* | Brazil | Planaltina | NA | 274 | CSA | (Fecchio *et al*., 2017) |
| KU562580 | -0,4 | -64,8 | *Plasmodium* sp. | *Xiphorhynchus pardalotus* | Brazil | Negro River | NA | 275 | AMN | (Fecchio *et al*., 2017) |
| KU562428 | -4,683 | -56,63 | *Plasmodium* sp. | *Hypocnemis striata* | Brazil | Tapajόs River | NA | 276 | AMS | (Fecchio *et al*., 2017) |
| KU562645 | -4,983 | -62,13 | *Plasmodium* sp. | *Formicarius colma* | Brazil | Purus River | NA | 277 | AMN | (Fecchio *et al*., 2017) |
| KU562450 | -4,5 | -56,283 | *Plasmodium* sp. | *Thamnomanes caesius* | Brazil | Tapajόs River | NA | 277 | AMS | (Fecchio *et al*., 2017) |
| KU562409 | -4,683 | -56,63 | *Plasmodium* sp. | *Hypocnemis striata* | Brazil | Tapajόs River | NA | 278 | AMS | (Fecchio *et al*., 2017) |
| KU562495 | -4,683 | -56,63 | *Plasmodium* sp. | *Thamnomanes caesius* | Brazil | Tapajόs River | NA | 279 | AMS | (Fecchio *et al*., 2017) |
| KU562496 | -4,683 | -56,63 | *Plasmodium* sp. | *Thamnomanes saturninus* | Brazil | Tapajόs River | NA | 280 | AMS | (Fecchio *et al*., 2017) |
| KU562500 | -4,5 | -56,283 | *Plasmodium* sp. | *Thamnomanes saturninus* | Brazil | Tapajόs River | NA | 281 | AMS | (Fecchio *et al*., 2017) |
| KU562411 | -4,683 | -56,63 | *Plasmodium* sp. | *Willisornis poecilinotus* | Brazil | Tapajόs River | NA | 282 | AMS | (Fecchio *et al*., 2017) |
| KU562460 | -4,5 | -56,283 | *Plasmodium* sp. | *Automolus ochrolaemus* | Brazil | Tapajόs River | NA | 282 | AMS | (Fecchio *et al*., 2017) |
| KU562461 | -12,567 | -70,083 | *Plasmodium* sp. | *Automolus infuscatus* | Peru | Manu | NA | 282 | CAN | (Fecchio *et al*., 2017) |
| KU562462 | -12,567 | -70,083 | *Plasmodium* sp. | *Cercomacra nigrescens* | Peru | Manu | NA | 282 | CAN | (Fecchio *et al*., 2017) |
| KU562610 | -0,583 | -64,917 | *Plasmodium* sp. | *Automolus infuscatus* | Brazil | Negro River | NA | 282 | AMN | (Fecchio *et al*., 2017) |
| KU562463 | -12,567 | -70,083 | *Plasmodium* sp. | *Automolus rufipileatus* | Peru | Manu | NA | 282 | CAN | (Fecchio *et al*., 2017) |
| KU562457 | -4,5 | -56,283 | *Plasmodium* sp. | *Myrmotherula longipennis* | Brazil | Tapajόs River | NA | 283 | AMS | (Fecchio *et al*., 2017) |
| KU562783 | -13,8 | -59,683 | *Plasmodium* sp. | *Turdus amaurochalinus* | Brazil | Comodoro | NA | 284 | CSA | (Fecchio *et al*., 2017) |
| KU562782 | -13,8 | -59,683 | *Plasmodium* sp. | *Hypocnemis cantator* | Brazil | Comodoro | NA | 285 | CSA | (Fecchio *et al*., 2017) |
| KU562781 | -13,8 | -59,683 | *Plasmodium* sp. | *Xenopipo atronitens* | Brazil | Comodoro | NA | 286 | CSA | (Fecchio *et al*., 2017) |
| KU562785 | -13,8 | -59,683 | *Plasmodium* sp. | *Willisornis poecilinotus* | Brazil | Comodoro | NA | 287 | CSA | (Fecchio *et al*., 2017) |
| KU562786 | -13,8 | -59,683 | *Plasmodium* sp. | *Willisornis poecilinotus* | Brazil | Comodoro | NA | 287 | CSA | (Fecchio *et al*., 2017) |
| KU562741 | -3,7 | -46,75 | *Plasmodium* sp. | *Pyrrhura lepida* | Brazil | Gurupi | NA | 288 | AMS | (Fecchio *et al*., 2017) |
| KU562740 | -3,7 | -46,75 | *Plasmodium* sp. | *Aratinga jandaya* | Brazil | Gurupi | NA | 289 | AMS | (Fecchio *et al*., 2017) |
| KU562742 | -3,7 | -46,75 | *Plasmodium* sp. | *Sporophila americana* | Brazil | Gurupi | NA | 290 | AMS | (Fecchio *et al*., 2017) |
| KU562826 | -12,567 | -70,083 | *Plasmodium* sp. | *Turdus hauxwelli* | Peru | Manu | NA | 291 | CAN | (Fecchio *et al*., 2017) |
| KU562825 | -12,567 | -70,083 | *Plasmodium* sp. | *Myrmoborus leucophrys* | Peru | Manu | NA | 291 | CAN | (Fecchio *et al*., 2017) |
| KU562823 | -12,567 | -70,083 | *Plasmodium* sp. | *Automolus ochrolaemus* | Peru | Manu | NA | 292 | CAN | (Fecchio *et al*., 2017) |
| KU562838 | -12,567 | -70,083 | *Plasmodium* sp. | *Saltator maximus* | Peru | Manu | NA | 293 | CAN | (Fecchio *et al*., 2017) |
| KU562830 | -12,567 | -70,083 | *Plasmodium* sp. | *Dendrocincla fuliginosa* | Peru | Manu | NA | 294 | CAN | (Fecchio *et al*., 2017) |
| KU562595 | -0,583 | -64,917 | *Plasmodium* sp. | *Phlegopsis erythroptera* | Brazil | Negro River | NA | 295 | AMN | (Fecchio *et al*., 2017) |
| KU562833 | -12,567 | -70,083 | *Plasmodium* sp. | *Tangara schrankii* | Peru | Manu | NA | 295 | CAN | (Fecchio *et al*., 2017) |
| KU562803 | -9,45 | -64,383 | *Plasmodium* sp. | *Sciaphylax hemimelaena* | Brazil | Porto Velho | NA | 296 | AMS | (Fecchio *et al*., 2017) |
| KU562661 | -5,717 | -63,2 | *Plasmodium* sp. | *Myrmotherula axillaris* | Brazil | Purus River | NA | 297 | AMN | (Fecchio *et al*., 2017) |
| KU562403 | -4,7 | -56,53 | *Plasmodium* sp. | *Thamnomanes saturninus* | Brazil | Tapajόs River | NA | 298 | AMS | (Fecchio *et al*., 2017) |
| KU562404 | -12,217 | -60,73 | *Plasmodium* sp. | *Willisornis poecilinotus* | Brazil | Chupinguaia | NA | 298 | AMS | (Fecchio *et al*., 2017) |
| KU562405 | -12,217 | -60,73 | *Plasmodium* sp. | *Willisornis poecilinotus* | Brazil | Chupinguaia | NA | 298 | AMS | (Fecchio *et al*., 2017) |
| KU562406 | -13,8 | -59,683 | *Plasmodium* sp. | *Willisornis poecilinotus* | Brazil | Comodoro | NA | 298 | CSA | (Fecchio *et al*., 2017) |
| KU562407 | -12,217 | -60,73 | *Plasmodium* sp. | *Willisornis poecilinotus* | Brazil | Chupinguaia | NA | 298 | AMS | (Fecchio *et al*., 2017) |
| KU562408 | -9,017 | -64,23 | *Plasmodium* sp. | *Pipra fasciicauda* | Brazil | Madeira River | NA | 298 | AMS | (Fecchio *et al*., 2017) |
| KU562660 | -5,717 | -63,2 | *Plasmodium* sp. | *Willisornis poecilinotus* | Brazil | Purus River | NA | 298 | AMN | (Fecchio *et al*., 2017) |
| KU562695 | -16,467 | -58,13 | *Plasmodium* sp. | *Pyriglena leuconota* | Brazil | Cáceres | NA | 299 | CSA | (Fecchio *et al*., 2017) |
| KU562696 | -16,467 | -58,13 | *Plasmodium* sp. | *Pyriglena leuconota* | Brazil | Cáceres | NA | 299 | CSA | (Fecchio *et al*., 2017) |
| KU562697 | -16,467 | -58,13 | *Plasmodium* sp. | *Pyriglena leuconota* | Brazil | Cáceres | NA | 299 | CSA | (Fecchio *et al*., 2017) |
| KU562653 | -4,983 | -62,13 | *Plasmodium* sp. | *Hafferia fortis* | Brazil | Purus River | NA | 299 | AMN | (Fecchio *et al*., 2017) |
| KU562674 | -5,717 | -63,2 | *Plasmodium* sp. | *Myrmotherula axillaris* | Brazil | Purus River | NA | 300 | AMN | (Fecchio *et al*., 2017) |
| KU562712 | -3,7 | -46,75 | *Plasmodium* sp. | *Dysithamnus mentalis* | Brazil | Gurupi | NA | 301 | AMS | (Fecchio *et al*., 2017) |
| KU562709 | -6,582 | -37,267 | *Plasmodium* sp. | *Coryphospingus pileatus* | Brazil | Serra Negra do Norte | NA | 302 | ATL | (Fecchio *et al*., 2017) |
| KU562590 | -0,4 | -64,8 | *Plasmodium* sp. | *Isleria guttata* | Brazil | Negro River | NA | 303 | AMN | (Fecchio *et al*., 2017) |
| KU562694 | -16,467 | -58,13 | *Plasmodium* sp. | *Pyriglena leuconota* | Brazil | Cáceres | NA | 303 | CSA | (Fecchio *et al*., 2017) |
| KU562217 | -19,567 | -57,017 | *Haemoproteus* sp. | *Paroaria capitata* | Brazil | Corumbá | NA | 304 | CSA | (Fecchio *et al*., 2017) |
| KU562203 | -0,4 | -64,8 | *Haemoproteus* sp. | *Chloroceryle inda* | Brazil | Santa Isabel do Rio Negro | NA | 305 | AMN | (Fecchio *et al*., 2017) |
| KU562200 | -15,53 | -47,55 | *Haemoproteus* sp. | *Cypsnagra hirundinacea* | Brazil | Planaltina | NA | 306 | CSA | (Fecchio *et al*., 2017) |
| KU562199 | -15,53 | -47,55 | *Haemoproteus* sp. | *Cypsnagra hirundinacea* | Brazil | Planaltina | NA | 306 | CSA | (Fecchio *et al*., 2017) |
| KU562232 | -3,7 | -46,75 | *Haemoproteus* sp. | *Thalurania furcata* | Brazil | Gurupi | NA | 307 | AMS | (Fecchio *et al*., 2017) |
| KU562234 | -3,7 | -46,75 | *Haemoproteus* sp. | *Campephilus rubricollis* | Brazil | Gurupi | NA | 307 | AMS | (Fecchio *et al*., 2017) |
| KU562235 | -3,7 | -46,75 | *Haemoproteus* sp. | *Campephilus rubricollis* | Brazil | Gurupi | NA | 307 | AMS | (Fecchio *et al*., 2017) |
| KU562149 | -4,683 | -56,63 | *Haemoproteus* sp. | *Phlegopsis nigromaculata* | Brazil | Tapajόs River | NA | 308 | AMS | (Fecchio *et al*., 2017) |
| KU562150 | -4,683 | -56,63 | *Haemoproteus* sp. | *Phlegopsis nigromaculata* | Brazil | Tapajόs River | NA | 308 | AMS | (Fecchio *et al*., 2017) |
| KU562148 | -4,683 | -56,63 | *Haemoproteus* sp. | *Phlegopsis nigromaculata* | Brazil | Tapajόs River | NA | 309 | AMS | (Fecchio *et al*., 2017) |
| KU562163 | -4,5 | -56,267 | *Haemoproteus* sp. | *Cantorchilus leucotis* | Brazil | Tapajόs River | NA | 310 | AMS | (Fecchio *et al*., 2017) |
| KU562346 | -5,1 | -56,43 | *Plasmodium* sp. | *Thamnomanes caesius* | Brazil | Jamanxim River | NA | 311 | AMS | (Fecchio *et al*., 2017) |
| KU562395 | -4,5 | -56,283 | *Plasmodium* sp. | *Isleria hauxwelli* | Brazil | Tapajόs River | NA | 312 | AMS | (Fecchio *et al*., 2017) |
| KU562366 | -4,7 | -56,53 | *Plasmodium* sp. | *Thamnomanes saturninus* | Brazil | Tapajόs River | NA | 313 | AMS | (Fecchio *et al*., 2017) |
| KU562380 | -5,1 | -56,43 | *Plasmodium* sp. | *Willisornis poecilinotus* | Brazil | Jamanxim River | NA | 314 | AMS | (Fecchio *et al*., 2017) |
| KU562379 | -4,7 | -56,53 | *Plasmodium* sp. | *Thamnomanes saturninus* | Brazil | Tapajόs River | NA | 315 | AMS | (Fecchio *et al*., 2017) |
| KC867672 | 0,318 | -90,469 | *Plasmodium* sp. | *Geospiza fuliginosa* | Ecuador | Galápagos Island- Marchena Island | GEOFUL01 | 316 | NAN | (Levin *et al*., 2013) |
| HM146901 | 26,82 | 30,802 | *Plasmodium* sp. | *Passer domesticus* | Egypt | NA | NA | 317 | NA | (Walther *et al*., 2014) |
| EU770151 | 7,946 | -1,023 | *Plasmodium globularis* | *Eurillas latirostris* | Ghana | NA | NA | 318 | NA | (Walther *et al*., 2014) |
| KC680701 | -0,633 | -76,133 | *Plasmodium* sp. | *Myrmoborus myotherinus* | Ecuador | Tiputini Biodiversity Station, Orellana Province | P15 | 319 | AMN | (Svensson-Coelho *et al*. 2016) |
| KF537292 | 4,804 | -75,713 | *Haemoproteus coatneyi* | *Arremon brunneinucha* | Colombia | Pereira | ARBRU01 | 320 | NAN | (González *et al*., 2015) |
| KJ661308 | 0,362 | -79,716 | *Haemoproteus* sp. | *Thalurania fannyi* | Ecuador | Bilsa | NA | 321 | EPC | (Harrigan *et al*., 2014) |
| KJ661333 | -1,477 | -78,16 | *Haemoproteus* sp. | *Glyphorynchus spirurus* | Ecuador | Cumandá | NA | 322 | NAN | (Harrigan *et al*., 2014) |
| KJ661332 | -1,477 | -78,16 | *Plasmodium* sp. | *Glyphorynchus spirurus* | Ecuador | Cumandá | NA | 323 | NAN | (Harrigan *et al*., 2014) |
| JQ988623 | -12,029 | -76,65 | *Parahaemoproteus* sp. | *Leptasthenura pileata* | Peru | Lima | NA | 324 | STP | (Galen and Witt, 2014) |
| AY817751 | 37,964 | -91,832 | *Haemoproteus* sp. | *Vireo olivaceus* | United States of America | Misuri | NA | 325* | NA | (Ricklefs *et al*., 2017) |
| AY167239 | 14,609 | -61,072 | *Haemoproteus* sp. | *Loxigilla noctis* | Antilles | Lesser Antilles - Martinique Island | HA | 325* | LAN | (Fallon *et al*., 2005; Fallon *et al*., 2003) |
| AY167239 | 17,075 | -61,817 | *Haemoproteus* sp. | *Vireo altiloquus* | Antilles | Lesser Antilles - Antigua Island | HA | 325* | LAN | (Fallon *et al*., 2005; Fallon *et al*., 2003) |
| AY817750 | 37,964 | -91,832 | *Haemoproteus* sp. | *Vireo griseus* | United States of America | Misuri | NA | 326* | NA | (Ricklefs *et al*., 2017) |
| AY455661 | 16,235 | -61,488 | *Plasmodium* sp. | *Coereba flaveola* | Antilles | NA | PE | 327* | LAN | (Fallon *et al*., 2005) |
| AY841003 | 16,235 | -61,488 | *Apicomplexa* sp. | *Columbina passerina* | Antilles | NA | CPA2 | 328* | LAN | (Fallon *et al*., 2005) |
| AY840998 | 16,235 | -61,488 | *Haemoproteus* sp. | *Sin registro* | Antilles | NA | HK | 328* | LAN | (Fallon *et al*., 2005) |
| AY840999 | 16,235 | -61,488 | *Haemoproteus* sp. | *Sin registro* | Antilles | NA | HL | 328* | LAN | (Fallon *et al*., 2005) |
| AY841004 | 16,235 | -61,488 | *Apicomplexa* sp. | *Zenaida aurita* | Antilles | NA | Zed | 328* | LAN | (Fallon *et al*., 2005) |
| AY455663 | 16,235 | -61,488 | *Plasmodium* sp. | *Seiurus aurocapillus* | Antilles | NA | PG | 329* | LAN | (Fallon *et al*., 2005) |
| AY167244 | 17,626 | -61,77 | *Haemoproteus* sp. | *Vireo altiloquus* | Antilles | Antillas menores - Barbuda Island | HU2 | 330* | LAN | (Fallon *et al*., 2005; Fallon *et al*., 2003) |
| DQ241540 | 4,86 | -58,93 | *Haemoproteus* sp. | *Psarocolius viridis* | Guyana | NA | 33 | 331 | AMN | (Durrant *et al*., 2006) |
| DQ241536 | -32,522 | -55,765 | *Plasmodium* sp. | *Ardea alba* | Uruguay | NA | 29 | 332 | PAM | (Durrant *et al*., 2006) |
| DQ241536 | -32,522 | -55,765 | *Plasmodium* sp. | *Megascops choliba* | Uruguay | NA | 29 | 332 | PAM | (Durrant *et al*., 2006) |
| KC680673 | -0,633 | -76,133 | *Plasmodium* sp. | *Myrmoborus myotherinus* | Ecuador | Tiputini Biodiversity Station, Orellana Province | P8 | 333* | AMN | (Svensson-Coelho *et al*. 2016) |
| AB250415 | -32,522 | -55,765 | *Plasmodium juxtanucleare* | NA | Uruguay | NA | NA | 334 | PAM | (Mantilla *et al*., 2013) |
| KC138226 | 4,804 | -75,713 | *Plasmodium lutzi* | *Turdus fuscater* | Colombia | Pereira | TFUS05 | 335 | NAN | (González *et al*., 2015; Mantilla *et al*., 2013) |
| AY841001 | 16,235 | -61,488 | *Plasmodium* sp. | *Sin registro* | Antilles | NA | PI | 335 | LAN | (Fallon *et al*., 2005) |
| KC789828 | 37,09 | -95,712 | *Plasmodium* sp. | *Turdus migratorius* | United States of America | NA | NA | 335 | NA | (Ricklefs *et al*., 2017) |
| KF537310 | 4,804 | -75,713 | *Plasmodium lutzi* | *Diglossa cyanea* | Colombia | Pereira | DICYA02 | 335 | NAN | (González *et al*., 2015) |
| HM031936 | -23,65 | -46,617 | *Plasmodium* sp. | *Musophaga violacea* | Brazil | São Paulo Zoo | NA | 336 | ATL | (Chagas *et al*., 2017) |
| DQ241522 | 4,86 | -58,93 | *Plasmodium* sp. | *Diopsittaca nobilis* | Guyana | NA | 15 | 336 | AMN | (Durrant *et al*., 2006) |
| KU562808 | -9,1167 | -64,467 | *Plasmodium* sp. | *Turdus albicollis* | Brazil | Porto Velho | NA | 336 | AMS | (Fecchio *et al*., 2017) |
| GQ395636 | -0,829 | -90,982 | *Haemoproteus* sp. | *Spheniscus mendiculus* | Ecuador | Galápagos Islands | NA | 337 | NAN | (Ricklefs *et al*., 2017) |
| GQ141575 | NA | NA | *Parahaemoproteus* sp. | *Dendroica magnolia* | North America Continental | NA | DENMAG01 | 338 | NA | (Galen and Witt, 2014) |
| GQ395658 | -0,829 | -90,982 | *Haemoproteus* sp. | *Spheniscus mendiculus* | Ecuador | Galápagos Islands | NA | 339 | NAN | (Ricklefs *et al*., 2017) |
| GQ141571 | 37,09 | -95,712 | *Parahaemoproteus* sp. | *Icterus leucopteryx* | United States of America | NA | NA | 339 | NA | (Ricklefs *et al*., 2017) |
| GQ141571 | NA | NA | *Parahaemoproteus* sp. | *Icterus leucopteryx* | Antilles | NA | ICTLEU01 | 339 | NA | (Galen and Witt, 2014) |
| AF069611 | NA | NA | *Plasmodium elongatum* | *Sin registro* | Antilles | NA | NA | 340 | NA | (Fallon *et al*., 2005) |
| AF069611 | -23,65 | -46,617 | *Plasmodium* sp. | *Anser cygnoides* | Brazil | São Paulo Zoo | NA | 340 | ATL | (Chagas *et al*., 2017) |
| DQ659550 | 4,86 | -58,93 | *Plasmodium* sp. | *Emberizoides herbicola* | Guyana | NA | NA | 340 | AMN | (Ricklefs *et al*., 2017) |
| DQ838997 | -32,522 | -55,765 | *Plasmodium* sp. | *Troglodytes aedon* | Uruguay | NA | NA | 340 | PAM | (Ricklefs *et al*., 2017) |
| KF537316 | 4,804 | -75,713 | *Plasmodium* sp. | *Leiothlypis peregrina* | Colombia | Pereira | PACPEC02 | 340 | NAN | (González *et al*., 2015) |
| KC771248 | 4,711 | -74,072 | *Plasmodium unalis* | *Turdus fuscater* | Colombia | Bogotá, D.C. | TFUS06 | 341 | NAN | (Mantilla *et al*., 2013) |
| HM222477 | 41,603 | -73,087 | *Plasmodium* sp. | *Turdus rufiventris* | United States of America | Connecticut | OZ35 | 341 | NA | (Mantilla *et al*., 2013; Marzal *et al*., 2015) |
| KF537323 | 4,804 | -75,713 | *Plasmodium unalis* | *Turdus fuscater* | Colombia | Pereira | TFUS06 | 341 | NAN | (González *et al*., 2015) |
| GQ141557 | NA | NA | *Parahaemoproteus* sp. | *Anas crecca* | North America Continental | NA | ANACRE01 | 342 | NA | (Galen and Witt, 2014) |
| GQ141573 | NA | NA | *Parahaemoproteus* sp. | *Coereba flaveola* | Antilles | NA | COFLA08 | 343 | NA | (Galen and Witt, 2014) |
| AF465573 | NA | NA | *Haemoproteus* sp. | *Corvus brachyrhynchos* | North America Continental | NA | COBRA01 | 344 | NA | (Galen and Witt, 2014) |
| KF537315 | 4,804 | -75,713 | *Haemoproteus* sp. | *Zonotrichia capensis* | Colombia | Pereira | ZOCAP09 | 345 | NAN | (González *et al*., 2015) |
| KF537315 | 4,711 | -74,072 | *Haemoproteus* sp. | *Zonotrichia capensis* | Colombia | Bogotá, D.C. | ZOCAP09 | 345 | NAN | (Mantilla *et al*., 2016) |
| AF465564 | NA | NA | *Haemoproteus* sp. | *Toxostoma rufum* | North America Continental y Antilles | NA | TOXRUF01 | 346 | NA | (Galen and Witt, 2014) |
| GQ141599 | NA | NA | *Parahaemoproteus* sp. | *Mimus gilvus* | Antilles | NA | MIMGIL01 | 346 | NA | (Galen and Witt, 2014) |
| HQ287546 | -10,249 | -48,324 | *Haemoproteus* sp. | *Myiopagis viridicata* | Brazil | Palmas,Tocantins | Toc-20 | 346 | CSA | (Belo *et al*., 2011) |
| GU256262 | NA | NA | *Parahaemoproteus* sp. | *Vireo olivaceus* | North America Continental | NA | NA | 347 | NA | (Galen and Witt, 2014) |
| EU627834 | 46,729 | -94,685 | *Haemoproteus* sp. | *Strix varia* | United States of America | Minnesota | STVAR03 | 348 | NA | (Galen and Witt, 2014) |
| AF465589 | NA | NA | *Haemoproteus* sp. | *Strix varia* | North America Continental | NA | STVAR01 | 348 | NA | (Galen and Witt, 2014) |
| AF465589 | NA | NA | *Haemoproteus* sp. | *Bubo virginianus* | North America Continental | NA | STVAR01 | 348 | NA | (Galen and Witt, 2014) |
| EU627838 | 38,837 | -120,895 | *Haemoproteus* sp. | *Tyto alba* | United States of America | Northern California | BNOW02 | 349 | NA | (Galen and Witt, 2014) |
| JN819379 | 9,748 | -83,753 | *Haemoproteus* sp. | *Tangara icterocephala* | Costa Rica | NA | NA | 350 | CDH | (Galen and Witt, 2014) |
| KC680689 | -0,633 | -76,133 | *Plasmodium* sp. | *Formicarius analis* | Ecuador | Tiputini Biodiversity Station, Orellana Province | NA | 351 | AMN | (Ricklefs *et al*., 2017) |
| KC680704 | -0,633 | -76,133 | *Plasmodium* sp. | *Glyphorynchus spirurus* | Ecuador | Tiputini Biodiversity Station, Orellana Province | P41L | 352 | AMN | (Svensson-Coelho *et al*. 2016) |
| DQ241537 | 4,86 | -58,93 | *Plasmodium* sp. | *Cyanocompsa cyanoides* | Guyana | NA | 30 | 353 | AMN | (Durrant *et al*., 2006) |
| DQ241509 | -32,522 | -55,765 | *Plasmodium* sp. | *Microspingus lateralis* | Uruguay | NA | 2 | 354 | PAM | (Durrant *et al*., 2006) |
| DQ241509 | -32,522 | -55,765 | *Plasmodium* sp. | *Icterus cayanensis* | Uruguay | NA | 2 | 354 | PAM | (Durrant *et al*., 2006) |
| DQ241525 | 4,86 | -58,93 | *Plasmodium* sp. | *Agamia agami* | Guyana | NA | 18 | 355 | AMN | (Durrant *et al*., 2006) |
| HQ287548 | -10,249 | -48,324 | *Haemoproteus* sp. | *Cantorchilus leucotis* | Brazil | Palmas,Tocantins | Toc-29 | 356 | CSA | (Belo *et al*., 2011) |
| HQ287551 | -10,249 | -48,324 | *Plasmodium* sp. | *Hylophilus pectoralis* | Brazil | Palmas,Tocantins | Toc-19 | 357 | CSA | (Belo *et al*., 2011) |
| KC680683 | -0,633 | -76,133 | *Plasmodium* sp. | *Formicarius analis* | Ecuador | Tiputini Biodiversity Station, Orellana Province | P2 | 358 | AMN | (Svensson-Coelho *et al*. 2016) |
| KC680661 | -0,633 | -76,133 | *Plasmodium* sp. | *Myrmoborus myotherinus* | Ecuador | Tiputini Biodiversity Station, Orellana Province | P26 | 359 | AMN | (Svensson-Coelho *et al*. 2016) |
| KU562567 | -15,53 | -47,55 | *Plasmodium* sp. | *Saltator atricollis* | Brazil | Planaltina | NA | 360 | CSA | (Fecchio *et al*., 2017) |
| KU562568 | -15,53 | -47,55 | *Plasmodium* sp. | *Saltator atricollis* | Brazil | Planaltina | NA | 360 | CSA | (Fecchio *et al*., 2017) |
| KX171629 | -23,65 | -46,617 | *Plasmodium* sp. | *Saltator atricollis* | Brazil | São Paulo Zoo | NA | 360 | ATL | (Chagas *et al*., 2017) |
| JN788938 | 18,797 | -110,975 | *Haemoproteus* sp. | *Mimus polyglottos* | Mexico | Socorro Island | NA | 361 | PAS | (Galen and Witt, 2014) |
| KC789824 | 37,09 | -95,712 | *Plasmodium* sp. | *Passer domesticus* | United States of America | NA | NA | 362 | NA | (Ricklefs *et al*., 2017) |
| HQ287543 | -10,249 | -48,324 | *Plasmodium* sp. | *Cantorchilus leucotis* | Brazil | Palmas,Tocantins | Toc-11 | 363 | CSA | (Belo *et al*., 2011) |
| HQ287544 | -10,249 | -48,324 | *Haemoproteus* sp. | *Pipra fasciicauda* | Brazil | Palmas,Tocantins | Toc-13 | 364 | CSA | (Belo *et al*., 2011) |
| KJ469131 | -1,817 | -65,7 | *Plasmodium* sp. | *Rynchops niger* | Brazil | Amazonas | NA | 365 | AMN | (Roos *et al*. 2015) |
| EF153642 | -9,916 | -76,233 | *Plasmodium* sp. | *Sayornis nigricans* | Peru | Huánuco | PHPAT01 | 365 | CAN | (Marzal *et al*., 2015) |
| EF153642 | -53,163 | -70,917 | *Plasmodium* sp. | *Phrygilus patagonicus* | Chile | Punta Arenas | ChL5 | 365 | SAN | (Merino *et al*., 2008) |
| JX025077 | -17,0003 | -46,008 | *Plasmodium* sp. | *Phaeomyias murina* | Brazil | Brasilandia de Minas | PHPAT01 | 365 | CSA | (Lacorte *et al*., 2013) |
| JX025077 | -18,713 | -44,925 | *Plasmodium* sp. | *Myiothlypis flaveola* | Brazil | Felixlândia | PHPAT01 | 365 | CSA | (Lacorte *et al*., 2013) |
| JX025077 | -16,11 | -40,022 | *Plasmodium* sp. | *Ammodramus humeralis* | Brazil | Salto da Divisa | PHPAT01 | 365 | CSA | (Lacorte *et al*., 2013) |
| JX025077 | -16,11 | -40,022 | *Plasmodium* sp. | *Phaeomyias murina* | Brazil | Salto da Divisa | PHPAT01 | 365 | CSA | (Lacorte *et al*., 2013) |
| KC867663 | 43,193 | -71,572 | *Plasmodium* sp. | *Dolichonyx oryzivorus* | United States of America | New Hampshire - Platte River | PHPAT01 | 365 | NA | (Levin *et al*., 2013) |
| KC680662 | -0,633 | -76,133 | *Haemoproteus* sp. | *Lepidothrix coronata* | Ecuador | Tiputini Biodiversity Station, Orellana Province | H9L | 366 | AMN | (Svensson-Coelho *et al*. 2016) |
| MF077654 | 35,827 | -106,896 | *Haemoproteus* sp. | *Vireo gilvus* | United States of America | Nuevo Mexico, Elk Springs | VIGIL07 | 367 | NA | (Marroquin-Flores *et al*., 2017) |
| KM211351 | 4,804 | -75,713 | *Haemoproteus* sp. | *Leiothlypis peregrina* | Colombia | Pereira | VEPER02 | 367 | NAN | (González *et al*., 2015) |
| KM065800 | 37,09 | -95,712 | *Haemoproteus* sp. | *Mimus polyglottos* | United States of America | NA | NA | 368 | NA | (Ricklefs *et al*., 2017) |
| KF537297 | 4,804 | -75,713 | *Haemoproteus coatneyi* | *Hemispingus atropileus* | Colombia | Pereira | HEATR02 | 369 | NAN | (González *et al*., 2015) |
| KF537283 | 4,804 | -75,713 | *Haemoproteus coatneyi* | *Tangara vassorii* | Colombia | Pereira | HEATR02 | 369 | NAN | (González *et al*., 2015) |
| KF537298 | 4,804 | -75,713 | *Haemoproteus coatneyi* | *Hemispingus atropileus* | Colombia | Pereira | HEATR02 | 369 | NAN | (González *et al*., 2015) |
| KF537308 | 4,804 | -75,713 | *Haemoproteus coatneyi* | *Hemispingus atropileus* | Colombia | Pereira | HEATR02 | 369 | NAN | (González *et al*., 2015) |
| KF482358 | -12,217 | -76,985 | *Plasmodium* sp. | *Troglodytes aedon* | Peru | Pantanos de Villa wetland Reserve | TROGLODY01 | 370 | STP | (Marzal *et al*., 2015) |
| KT373873 | -2,117 | -77,733 | *Plasmodium* sp. | *Hypocnemis cantator* | Ecuador | Morona-Santiago Province, Wisui | HYPCAN01 | 370 | AMN | (Moens & Pérez-Tris, 2016) |
| KT373873 | -2,087 | -77,751 | *Plasmodium* sp. | *Hypocnemis cantator* | Ecuador | Wisui reserve | NA | 370 | AMN | (Moens *et al*., 2017) |
| KC680714 | -0,633 | -76,133 | *Plasmodium* sp. | *Hylophilus ochraceiceps* | Ecuador | Tiputini Biodiversity Station, Orellana Province | P18 | 371 | AMN | (Svensson-Coelho *et al*. 2016) |
| KC680660 | -0,633 | -76,133 | *Haemoproteus* sp. | *Pipra filicauda* | Ecuador | Tiputini Biodiversity Station, Orellana Province | H2 | 372 | AMN | (Svensson-Coelho *et al*. 2016) |
| KC867653 | -0,829 | -91,135 | *Plasmodium* sp. | *Setophaga petechia* | Ecuador | Galápagos Island -Isabela Island | MYITYR01 | 373 | NAN | (Levin *et al*., 2013) |
| JX029905 | -16,11 | -40,022 | *Haemoproteus* sp. | *Nemosia pileata* | Brazil | Salto da Divisa | PAPOL01 | 374 | CSA | (Lacorte *et al*., 2013; Galen & Witt, 2014) |
| JX029905 | -16,11 | -40,022 | *Haemoproteus* sp. | *Nyctidromus albicollis* | Brazil | Salto da Divisa | PAPOL01 | 374 | CSA | (Lacorte *et al*., 2013; Galen & Witt, 2014) |
| JX029905 | -16,11 | -40,022 | *Haemoproteus* sp. | *Pachyramphus polychopterus* | Brazil | Salto da Divisa | PAPOL01 | 374 | CSA | (Lacorte *et al*., 2013; Galen & Witt, 2014) |
| JX029905 | -16,11 | -40,022 | *Haemoproteus* sp. | *Paroaria dominicana* | Brazil | Salto da Divisa | PAPOL01 | 374 | CSA | (Lacorte *et al*., 2013; Galen & Witt, 2014) |
| KU364576 | -4,235 | -79,174 | *Haemoproteus witti* | *Adelomyia melanogenys* | Ecuador | Podocarpus National Park | TROAED20 | 374 | NAN | (Moens *et al*., 2016) |
| KU364577 | -4,387 | -79,146 | *Haemoproteus witti* | *Coeligena torquata* | Ecuador | Podocarpus National Park | TROAED20 | 374 | NAN | (Moens *et al*., 2016) |
| KU364578 | -4,235 | -79,174 | *Haemoproteus witti* | *Diglossa albilatera* | Ecuador | Podocarpus National Park | TROAED20 | 374 | NAN | (Moens *et al*., 2016) |
| KU364579 | -4,235 | -79,174 | *Haemoproteus witti* | *Diglossa cyanea* | Ecuador | Podocarpus National Park | TROAED20 | 374 | NAN | (Moens *et al*., 2016) |
| DQ847271 | NA | NA | *Plasmodium* sp. | Sp. | Europe, Australia, North America, Pacific | NA | SYAT05 | 375 | NA | (Mantilla *et al*., 2013) |
| DQ847271 | NA | NA | *Plasmodium* sp. | Sp. | Europe, Australia, North America, Pacific | NA | SYAT05 | 375 | NA | (Mantilla *et al*., 2013) |
| JQ988527 | -11,983 | -74,933 | *Parahaemoproteus* sp. | *Synallaxis azarae* | Peru | Junín | NA | 376 | CAN | (Galen and Witt, 2014) |
| JQ988404 | -13,249 | -72,169 | *Parahaemoproteus* sp. | *Patagona gigas* | Peru | Cusco | NA | 377 | CAN | (Galen and Witt, 2014) |
| KJ661260 | -0,599 | -77,89 | *Haemoproteus* sp. | *Adelomyia melanogenys* | Ecuador | Yanayacu | NA | 377 | NAN | (Harrigan *et al*., 2014) |
| MF077655 | 35,827 | -106,896 | *Plasmodium* sp. | *Empidonax hammondii* | United States of America | Nuevo Mexico, Elk Springs | EMPHAM02 | 378 | NA | (Marroquin-Flores *et al*., 2017) |
| JX021454 | -18,713 | -44,925 | *Plasmodium* sp. | *Leptopogon amaurocephalus* | Brazil | Felixlândia | LEAMA01 | 379 | CSA | (Lacorte *et al*., 2013) |
| JX021454 | -16,437 | -41,012 | *Plasmodium* sp. | *Conopophaga lineata* | Brazil | Jequitinhonha | LEAMA01 | 379 | CSA | (Lacorte *et al*., 2013) |
| JX021454 | -19,789 | -42,141 | *Plasmodium* sp. | *Leptopogon amaurocephalus* | Brazil | Caratinga | LEAMA01 | 379 | CSA | (Lacorte *et al*., 2013) |
| JQ988206 | -6,104 | -78,341 | *Parahaemoproteus* sp. | *Adelomyia melanogenys* | Peru | Amazonas | NA | 380 | CAN | (Galen and Witt, 2014) |
| JQ988414 | -5,896 | -79,785 | *Parahaemoproteus* sp. | *Chalcostigma herrani* | Peru | Lambayeque | NA | 380 | CAN | (Galen and Witt, 2014) |
| KF767423 | -8,386 | -78,645 | *Haemoproteus* sp. | *Troglodytes aedon* | Peru | La Libertad | TROAED18 | 380 | CAN | (Galen and Witt, 2014) |
| KU131584 | -23,65 | -46,617 | *Haemoproteus columbae* | *Columba livia* | Brazil | São Paulo Zoo | NA | 381 | ATL | (Chagas *et al*., 2016) |
| MF077651 | 35,533 | -107,349 | *Haemoproteus* sp. | *Spinus psaltria* | United States of America | Nuevo Mexico, Mesa Chivato | SPISAL01 | 382 | NA | (Marroquin-Flores *et al*., 2017) |
| JQ988355 | -13,163 | -74,223 | *Parahaemoproteus* sp. | *Coeligena torquata* | Peru | Ayacucho | NA | 383 | CAN | (Galen and Witt, 2014) |
| MF077653 | 35,827 | -106,896 | *Haemoproteus* sp. | *Cyanocitta stelleri* | United States of America | Nuevo Mexico, Elk Springs | CYASTE06 | 384 | NA | (Marroquin-Flores *et al*., 2017) |
| KF767412 | -12,651 | -72,323 | *Plasmodium* sp. | *Troglodytes aedon* | Peru | Cusco | TROAED22 | 385 | CAN | (Galen and Witt, 2014) |
| JX029884 | -16,11 | -40,022 | *Plasmodium* sp. | *Pachyramphus polychopterus* | Brazil | Salto da Divisa | PAPOL05 | 386 | CSA | (Lacorte *et al*., 2013) |
| JX029882 | -17,111 | -43,82 | *Plasmodium* sp. | *Euphonia chlorotica* | Brazil | Bocaiúva | EUCHL01 | 387 | CSA | (Lacorte *et al*., 2013) |
| JX029889 | -17,0003 | -46,008 | *Plasmodium* sp. | *Molothrus bonariensis* | Brazil | Brasilandia de Minas | MOBON01 | 388 | CSA | (Lacorte *et al*., 2013) |
| AF495571 | -9,916 | -76,233 | *Plasmodium* sp. | *Colibri coruscans* | Peru | Huánuco | SERCIN01 | 389 | CAN | (Marzal *et al*., 2015) |
| AF495571 | -9,916 | -76,233 | *Plasmodium* sp. | *Sayornis nigricans* | Peru | Huánuco | SERCIN01 | 389 | CAN | (Marzal *et al*., 2015) |
| AF495571 | -9,916 | -76,233 | *Plasmodium* sp. | *Serpophaga cinerea* | Peru | Huánuco | SERCIN01 | 389 | CAN | (Marzal *et al*., 2015) |
| AF495571 | -9,916 | -76,233 | *Plasmodium* sp. | *Zonotrichia capensis* | Peru | Huánuco | SERCIN01 | 389 | CAN | (Marzal *et al*., 2015) |
| AF495571 | -12,217 | -76,985 | *Plasmodium* sp. | *Conirostrum cinereum* | Peru | Pantanos de Villa wetland Reserve | SERCIN01 | 389 | STP | (Marzal *et al*., 2015) |
| AF495571 | -12,217 | -76,985 | *Plasmodium* sp. | *Phleocryptes melanops* | Peru | Pantanos de Villa wetland Reserve | SERCIN01 | 389 | STP | (Marzal *et al*., 2015) |
| AF495571 | -12,217 | -76,985 | *Plasmodium* sp. | *Troglodytes aedon* | Peru | Pantanos de Villa wetland Reserve | SERCIN01 | 389 | STP | (Marzal *et al*., 2015) |
| AF495571 | -9,916 | -76,233 | *Plasmodium* sp. | *Amazilia chionogaster* | Peru | Huánuco | SERCIN01 | 389 | CAN | (Marzal *et al*., 2015) |
| JX029871 | -18,713 | -44,925 | *Plasmodium* sp. | *Turdus leucomelas* | Brazil | Felixlândia | TULEU03 | 390 | CSA | (Lacorte *et al*., 2013) |
| KC680669 | -0,633 | -76,133 | *Haemoproteus* sp. | *Tachyphonus cristatus* | Ecuador | Tiputini Biodiversity Station, Orellana Province | H3 | 391 | AMN | (Svensson-Coelho *et al*. 2016) |
| AF254977 | NA | NA | *Haemoproteus majoris* | *Parus caeruleus* | North America Continental | NA | PARUS1 | 392 | NA | (Galen and Witt, 2014) |
| JX029896 | -19,82 | -40,276 | *Plasmodium* sp. | *Thamnophilus ambiguus* | Brazil | Aracruz | THAMB10 | 393 | ATL | (Lacorte *et al*., 2013) |
| JX029901 | -16,11 | -40,022 | *Haemoproteus* sp. | *Pachyramphus polychopterus* | Brazil | Salto da Divisa | FULEU01 | 394 | CSA | (Lacorte *et al*., 2013) |
| JX029901 | -16,11 | -40,022 | *Haemoproteus* sp. | *Furnarius leucopus* | Brazil | Salto da Divisa | FULEU01 | 394 | CSA | (Lacorte *et al*., 2013) |
| JX029901 | -22,959 | -44,041 | *Haemoproteus* sp. | *Furnarius leucopus* | Brazil | Manga | FULEU01 | 394 | ATL | (Lacorte *et al*., 2013) |
| JX021472 | -16,437 | -41,012 | *Plasmodium* sp. | *Tolmomyias flaviventris* | Brazil | Jequitinhonha | TOFLA01 | 395 | CSA | (Lacorte *et al*., 2013) |
| JX021472 | -22,959 | -44,041 | *Plasmodium* sp. | *Tolmomyias flaviventris* | Brazil | Manga | TOFLA01 | 395 | ATL | (Lacorte *et al*., 2013) |
| JX021472 | -16,11 | -40,022 | *Plasmodium* sp. | *Tolmomyias flaviventris* | Brazil | Salto da Divisa | TOFLA01 | 395 | CSA | (Lacorte *et al*., 2013) |
| JX021472 | -18,713 | -44,925 | *Plasmodium* sp. | *Phaeomyias murina* | Brazil | Felixlândia | TOFLA01 | 395 | CSA | (Lacorte *et al*., 2013) |
| KU562543 | -6,602 | -40,124 | *Plasmodium* sp. | *Pachyramphus validus* | Brazil | Aiuaba | NA | 395 | CSA | (Fecchio *et al*., 2017) |
| KU562544 | -3,7 | -46,75 | *Plasmodium* sp. | *Tolmomyias flaviventris* | Brazil | Gurupi | NA | 395 | AMS | (Fecchio *et al*., 2017) |
| JX021479 | -19,993 | -43,848 | *Plasmodium* sp. | *Conopophaga lineata* | Brazil | Nova Lima | COLIN01 | 396 | CSA | (Lacorte *et al*., 2013) |
| JX021479 | -19,789 | -42,141 | *Plasmodium* sp. | *Conopophaga lineata* | Brazil | Caratinga | COLIN01 | 396 | CSA | (Lacorte *et al*., 2013) |
| MF077673 | 35,533 | -107,349 | *Haemoproteus* sp. | *Vireo plumbeus* | United States of America | Nuevo Mexico, Mesa Chivato | VIRPLU01 | 397 | NA | (Marroquin-Flores *et al*., 2017) |
| JX021466 | -18,713 | -44,925 | *Plasmodium* sp. | *Turdus leucomelas* | Brazil | Felixlândia | TULEU02 | 398 | CSA | (Lacorte *et al*., 2013) |
| JX021466 | -16,437 | -41,012 | *Plasmodium* sp. | *Coryphospingus pileatus* | Brazil | Jequitinhonha | TULEU02 | 398 | CSA | (Lacorte *et al*., 2013) |
| JX021466 | -19,789 | -42,141 | *Plasmodium* sp. | *Turdus leucomelas* | Brazil | Caratinga | TULEU02 | 398 | CSA | (Lacorte *et al*., 2013) |
| JX021469 | -16,11 | -40,022 | *Plasmodium* sp. | *Euphonia violacea* | Brazil | Salto da Divisa | EUVIO01 | 399 | CSA | (Lacorte *et al*., 2013) |
| JX029866 | -17,0003 | -46,008 | *Plasmodium* sp. | *Hylocryptus rectirostris* | Brazil | Brasilandia de Minas | HYREC01 | 400 | CSA | (Lacorte *et al*., 2013) |
| JX029869 | -19,789 | -42,141 | *Plasmodium* sp. | *Thamnophilus ambiguus* | Brazil | Caratinga | THAMB08 | 401 | CSA | (Lacorte *et al*., 2013) |
| JX029869 | -19,098 | -40,186 | *Plasmodium* sp. | *Thamnophilus ambiguus* | Brazil | Sooretama | THAMB08 | 401 | ATL | (Lacorte *et al*., 2013) |
| JX029869 | -17,111 | -43,82 | *Plasmodium* sp. | *Thamnophilus ambiguus* | Brazil | Bocaiúva | THAMB08 | 401 | CSA | (Lacorte *et al*., 2013) |
| JX029868 | -17,111 | -43,82 | *Plasmodium* sp. | *Thamnophilus ambiguus* | Brazil | Bocaiúva | THAMB07 | 402 | CSA | (Lacorte *et al*., 2013) |
| JX021488 | -16,11 | -40,022 | *Plasmodium* sp. | *Pyriglena leucoptera* | Brazil | Salto da Divisa | PYLEU05 | 403 | CSA | (Lacorte *et al*., 2013) |
| JX021493 | -19,993 | -43,848 | *Plasmodium* sp. | *Conopophaga lineata* | Brazil | Nova Lima | COLIN09 | 404 | CSA | (Lacorte *et al*., 2013) |
| KU562597 | -0,583 | -64,917 | *Plasmodium* sp. | *Willisornis poecilinotus* | Brazil | Negro River | NA | 405 | AMN | (Fecchio *et al*., 2017) |
| KU562615 | -0,583 | -64,917 | *Plasmodium* sp. | *Thamnophilus murinus* | Brazil | Negro River | NA | 405 | AMN | (Fecchio *et al*., 2017) |
| KU562616 | -4,983 | -62,13 | *Plasmodium* sp. | *Willisornis poecilinotus* | Brazil | Purus River | NA | 405 | AMN | (Fecchio *et al*., 2017) |
| KU562617 | -4,983 | -62,13 | *Plasmodium* sp. | *Willisornis poecilinotus* | Brazil | Purus River | NA | 405 | AMN | (Fecchio *et al*., 2017) |
| KU562618 | -5,717 | -63,2 | *Plasmodium* sp. | *Willisornis poecilinotus* | Brazil | Purus River | NA | 405 | AMN | (Fecchio *et al*., 2017) |
| KU562619 | -5,717 | -63,2 | *Plasmodium* sp. | *Willisornis poecilinotus* | Brazil | Purus River | NA | 405 | AMN | (Fecchio *et al*., 2017) |
| KU562620 | -5,717 | -63,2 | *Plasmodium* sp. | *Isleria hauxwelli* | Brazil | Purus River | NA | 405 | AMN | (Fecchio *et al*., 2017) |
| KU562621 | -5,717 | -63,2 | *Plasmodium* sp. | *Myrmotherula longipennis* | Brazil | Purus River | NA | 405 | AMN | (Fecchio *et al*., 2017) |
| KU562622 | -9,1167 | -64,467 | *Plasmodium* sp. | *Willisornis poecilinotus* | Brazil | Porto Velho | NA | 405 | AMS | (Fecchio *et al*., 2017) |
| KU562598 | -0,583 | -64,917 | *Plasmodium* sp. | *Willisornis poecilinotus* | Brazil | Negro River | NA | 405 | AMN | (Fecchio *et al*., 2017) |
| KU562599 | -0,583 | -64,917 | *Plasmodium* sp. | *Willisornis poecilinotus* | Brazil | Negro River | NA | 406 | AMN | (Fecchio *et al*., 2017) |
| KU562602 | -0,583 | -64,917 | *Plasmodium* sp. | *Thamnomanes caesius* | Brazil | Negro River | NA | 407 | AMN | (Fecchio *et al*., 2017) |
| KU562592 | -0,4 | -64,8 | *Plasmodium* sp. | *Isleria guttata* | Brazil | Negro River | NA | 408 | AMN | (Fecchio *et al*., 2017) |
| KU562604 | -0,583 | -64,917 | *Plasmodium* sp. | *Willisornis poecilinotus* | Brazil | Negro River | NA | 409 | AMN | (Fecchio *et al*., 2017) |
| JX021456 | -16,437 | -41,012 | *Plasmodium* sp. | *Thamnophilus ambiguus* | Brazil | Jequitinhonha | THAMB01 | 410 | CSA | (Lacorte *et al*., 2013) |
| JX021456 | -3,944 | -73,607 | *Plasmodium* sp. | *Attila spadiceus* | Peru | Allpahuayo Mishana National Reserve | NA | 410 | AMN | (Ricopa & Villa, 2016) |
| KU562605 | -0,583 | -64,917 | *Plasmodium* sp. | *Hypocnemis hypoxantha* | Brazil | Negro River | NA | 410 | AMN | (Fecchio *et al*., 2017) |
| KU562579 | -0,4 | -64,8 | *Plasmodium* sp. | *Galbula albirostris* | Brazil | Negro River | NA | 411 | AMN | (Fecchio *et al*., 2017) |
| KU562576 | -15,53 | -47,55 | *Plasmodium* sp. | *Neothraupis fasciata* | Brazil | Planaltina | NA | 412 | CSA | (Fecchio *et al*., 2017) |
| KU562439 | -4,683 | -56,63 | *Plasmodium* sp. | *Myrmotherula axillaris* | Brazil | Tapajόs River | NA | 413 | AMS | (Fecchio *et al*., 2017) |
| KU562441 | -3,7 | -46,75 | *Plasmodium* sp. | *Myrmotherula axillaris* | Brazil | Gurupi | NA | 413 | AMS | (Fecchio *et al*., 2017) |
| KU562442 | -3,7 | -46,75 | *Plasmodium* sp. | *Myrmotherula axillaris* | Brazil | Gurupi | NA | 413 | AMS | (Fecchio *et al*., 2017) |
| KU562443 | -12,217 | -60,73 | *Plasmodium* sp. | *Myrmotherula axillaris* | Brazil | Chupinguaia | NA | 413 | AMS | (Fecchio *et al*., 2017) |
| KU562444 | -12,217 | -60,73 | *Plasmodium* sp. | *Myrmotherula axillaris* | Brazil | Chupinguaia | NA | 413 | AMS | (Fecchio *et al*., 2017) |
| KU562497 | -4,683 | -56,63 | *Plasmodium* sp. | *Galbula cyanicollis* | Brazil | Tapajόs River | NA | 413 | AMS | (Fecchio *et al*., 2017) |
| KU562440 | -4,683 | -56,63 | *Plasmodium* sp. | *Myrmotherula axillaris* | Brazil | Tapajόs River | NA | 413 | AMS | (Fecchio *et al*., 2017) |
| KU562449 | -4,5 | -56,283 | *Plasmodium* sp. | *Formicarius colma* | Brazil | Tapajόs River | NA | 414 | AMS | (Fecchio *et al*., 2017) |
| KU562445 | -4,683 | -56,63 | *Plasmodium* sp. | *Hypocnemis striata* | Brazil | Tapajόs River | NA | 415 | AMS | (Fecchio *et al*., 2017) |
| KU562410 | -4,683 | -56,63 | *Plasmodium* sp. | *Automolus ochrolaemus* | Brazil | Tapajόs River | NA | 416 | AMS | (Fecchio *et al*., 2017) |
| KU562509 | -4,5 | -56,267 | *Plasmodium* sp. | *Thamnophilus nigrocinereus* | Brazil | Tapajόs River | NA | 417 | AMS | (Fecchio *et al*., 2017) |
| KU562456 | -4,5 | -56,283 | *Plasmodium* sp. | *Myrmoborus myotherinus* | Brazil | Tapajόs River | NA | 418 | AMS | (Fecchio *et al*., 2017) |
| KU562763 | -1,35 | -56,367 | *Plasmodium* sp. | *Formicarius colma* | Brazil | Porto Trombetas | NA | 419 | AMN | (Fecchio *et al*., 2017) |
| KU562764 | -1,35 | -56,367 | *Plasmodium* sp. | *Formicarius colma* | Brazil | Porto Trombetas | NA | 419 | AMN | (Fecchio *et al*., 2017) |
| KU562765 | -1,35 | -56,367 | *Plasmodium* sp. | *Formicarius colma* | Brazil | Porto Trombetas | NA | 419 | AMN | (Fecchio *et al*., 2017) |
| KU562774 | -13,8 | -59,683 | *Plasmodium* sp. | *Lepidothrix nattereri* | Brazil | Comodoro | NA | 420 | CSA | (Fecchio *et al*., 2017) |
| KU562773 | -13,8 | -59,683 | *Plasmodium* sp. | *Lepidothrix nattereri* | Brazil | Comodoro | NA | 420 | CSA | (Fecchio *et al*., 2017) |
| KU562775 | -13,8 | -59,683 | *Plasmodium* sp. | *Machaeropterus pyrocephalus* | Brazil | Comodoro | NA | 421 | CSA | (Fecchio *et al*., 2017) |
| KU562777 | -13,8 | -59,683 | *Plasmodium* sp. | *Xenopipo atronitens* | Brazil | Comodoro | NA | 421 | CSA | (Fecchio *et al*., 2017) |
| KU562778 | -13,8 | -59,683 | *Plasmodium* sp. | *Hemitriccus margaritaceiventer* | Brazil | Comodoro | NA | 421 | CSA | (Fecchio *et al*., 2017) |
| KU562776 | -13,8 | -59,683 | *Plasmodium* sp. | *Hypocnemis cantator* | Brazil | Comodoro | NA | 421 | CSA | (Fecchio *et al*., 2017) |
| KU562744 | -3,7 | -46,75 | *Plasmodium* sp. | *Attila cinnamomeus* | Brazil | Gurupi | NA | 422 | AMS | (Fecchio *et al*., 2017) |
| KU562733 | -3,7 | -46,75 | *Plasmodium* sp. | *Pyriglena leuconota* | Brazil | Gurupi | NA | 423 | AMS | (Fecchio *et al*., 2017) |
| KU562736 | -3,7 | -46,75 | *Plasmodium* sp. | *Cercomacra cinerascens* | Brazil | Gurupi | NA | 424 | AMS | (Fecchio *et al*., 2017) |
| KU562623 | -0,583 | -64,917 | *Plasmodium* sp. | *Myrmotherula longipennis* | Brazil | Negro River | NA | 425 | AMN | (Fecchio *et al*., 2017) |
| KU562735 | -3,7 | -46,75 | *Plasmodium* sp. | *Micrastur mintoni* | Brazil | Gurupi | NA | 425 | AMS | (Fecchio *et al*., 2017) |
| KU562817 | -12,567 | -70,083 | *Plasmodium* sp. | *Automolus infuscatus* | Peru | Manu | NA | 426 | CAN | (Fecchio *et al*., 2017) |
| KU562816 | -12,567 | -70,083 | *Plasmodium* sp. | *Pipra fasciicauda* | Peru | Manu | NA | 427 | CAN | (Fecchio *et al*., 2017) |
| KU562819 | -12,567 | -70,083 | *Plasmodium* sp. | *Gymnopithys salvini* | Peru | Manu | NA | 428 | CAN | (Fecchio *et al*., 2017) |
| KU562842 | -12,567 | -70,083 | *Plasmodium* sp. | *Arremon taciturnus* | Peru | Manu | NA | 429 | CAN | (Ricklefs *et al*., 2017) |
| KU562841 | -12,567 | -70,083 | *Plasmodium* sp. | *Automolus ochrolaemus* | Peru | Manu | NA | 430 | CAN | (Fecchio *et al*., 2017) |
| KU562840 | -12,567 | -70,083 | *Plasmodium* sp. | *Cymbilaimus sanctaemariae* | Peru | Manu | NA | 431 | CAN | (Fecchio *et al*., 2017) |
| KU562815 | -12,567 | -70,083 | *Plasmodium* sp. | *Myrmoborus myotherinus* | Peru | Manu | NA | 432 | CAN | (Fecchio *et al*., 2017) |
| KU562797 | -9,133 | -64,617 | *Plasmodium* sp. | *Hylophylax naevius* | Brazil | Porto Velho | NA | 433 | AMS | (Fecchio *et al*., 2017) |
| KU562795 | -9,283 | -64,73 | *Plasmodium* sp. | *Formicarius colma* | Brazil | Porto Velho | NA | 434 | AMS | (Fecchio *et al*., 2017) |
| KU562791 | -9,283 | -64,73 | *Plasmodium* sp. | *Cercomacra cinerascens* | Brazil | Porto Velho | NA | 435 | AMS | (Fecchio *et al*., 2017) |
| DQ241527 | 4,86 | -58,93 | *Plasmodium* sp. | *Saltator maximus* | Guyana | NA | 20 | 436 | AMN | (Durrant *et al*., 2006) |
| KU562790 | -9,283 | -64,717 | *Plasmodium* sp. | *Cercomacra cinerascens* | Brazil | Porto Velho | NA | 436 | AMS | (Fecchio *et al*., 2017) |
| KU562651 | -4,983 | -62,13 | *Plasmodium* sp. | *Cercomacra serva* | Brazil | Purus River | NA | 436 | AMN | (Fecchio *et al*., 2017) |
| KU562652 | -4,983 | -62,13 | *Plasmodium* sp. | *Myrmoborus myotherinus* | Brazil | Purus River | NA | 437 | AMN | (Fecchio *et al*., 2017) |
| KU562707 | -6,582 | -37,267 | *Plasmodium* sp. | *Coryphospingus pileatus* | Brazil | Serra Negra do Norte | NA | 438 | ATL | (Fecchio *et al*., 2017) |
| KU562729 | -3,7 | -46,75 | *Plasmodium* sp. | *Philydor erythropterum* | Brazil | Gurupi | NA | 439 | AMS | (Fecchio *et al*., 2017) |
| KU562723 | -3,7 | -46,75 | *Plasmodium* sp. | *Myrmotherula axillaris* | Brazil | Gurupi | NA | 440 | AMS | (Fecchio *et al*., 2017) |
| KU562724 | -3,7 | -46,75 | *Plasmodium* sp. | *Myrmotherula axillaris* | Brazil | Gurupi | NA | 440 | AMS | (Fecchio *et al*., 2017) |
| KU562725 | -3,7 | -46,75 | *Plasmodium* sp. | *Myrmotherula axillaris* | Brazil | Gurupi | NA | 440 | AMS | (Fecchio *et al*., 2017) |
| KU562677 | -5,717 | -63,2 | *Plasmodium* sp. | *Ramphocelus carbo* | Brazil | Purus River | NA | 441 | AMN | (Fecchio *et al*., 2017) |
| KU562682 | -19,567 | -57,017 | *Plasmodium* sp. | *Paroaria capitata* | Brazil | Corumbá | NA | 442 | CSA | (Fecchio *et al*., 2017) |
| KU562221 | -6,582 | -37,267 | *Haemoproteus sp* | *Coryphospingus pileatus* | Brazil | Serra Negra do Norte | NA | 443 | ATL | (Fecchio *et al*., 2017) |
| KU562198 | -15,53 | -47,55 | *Haemoproteus* sp. | *Cypsnagra hirundinacea* | Brazil | Planaltina | NA | 444 | CSA | (Fecchio *et al*., 2017) |
| KU562201 | -15,53 | -47,55 | *Haemoproteus* sp. | *Neothraupis fasciata* | Brazil | Planaltina | NA | 445 | CSA | (Fecchio *et al*., 2017) |
| KU562245 | -12,567 | -70,083 | *Haemoproteus* sp. | *Myrmoborus myotherinus* | Peru | Manu | NA | 446 | CAN | (Fecchio *et al*., 2017) |
| KU562242 | -13,8 | -59,683 | *Haemoproteus* sp. | *Claravis pretiosa* | Brazil | Comodoro | NA | 447 | CSA | (Fecchio *et al*., 2017) |
| KU562254 | -5,067 | -56,85 | *Plasmodium* sp. | *Terenotriccus erythrurus* | Brazil | Tapajόs River | NA | 448 | AMS | (Fecchio *et al*., 2017) |
| KU562231 | -3,7 | -46,75 | *Haemoproteus* sp. | *Tachyphonus cristatus* | Brazil | Gurupi | NA | 449 | AMS | (Fecchio *et al*., 2017) |
| KU562233 | -3,7 | -46,75 | *Haemoproteus* sp. | *Psarocolius bifasciatus* | Brazil | Gurupi | NA | 450 | AMS | (Fecchio *et al*., 2017) |
| KU562188 | -15,53 | -47,55 | *Haemoproteus* sp. | *Suiriri suiriri* | Brazil | Planaltina | NA | 451 | CSA | (Fecchio *et al*., 2017) |
| KU562143 | -4,5 | -56,283 | *Haemoproteus* sp. | *Hylophylax punctulatus* | Brazil | Tapajόs River | NA | 452 | AMS | (Fecchio *et al*., 2017) |
| KU562142 | -4,5 | -56,283 | *Haemoproteus* sp. | *Glyphorynchus spirurus* | Brazil | Tapajόs River | NA | 453 | AMS | (Fecchio *et al*., 2017) |
| KU562157 | -4,683 | -56,63 | *Haemoproteus* sp. | *Phlegopsis nigromaculata* | Brazil | Tapajόs River | NA | 454 | AMS | (Fecchio *et al*., 2017) |
| KU562351 | -4,7 | -56,53 | *Plasmodium* sp. | *Phlegopsis nigromaculata* | Brazil | Tapajόs River | NA | 455 | AMS | (Fecchio *et al*., 2017) |
| KU562349 | -4,5 | -56,283 | *Plasmodium* sp. | *Phlegopsis nigromaculata* | Brazil | Tapajόs River | NA | 455 | AMS | (Fecchio *et al*., 2017) |
| KU562350 | -4,5 | -56,283 | *Plasmodium* sp. | *Thamnomanes saturninus* | Brazil | Tapajόs River | NA | 455 | AMS | (Fecchio *et al*., 2017) |
| KU562352 | -4,7 | -56,53 | *Plasmodium* sp. | *Phlegopsis nigromaculata* | Brazil | Tapajόs River | NA | 455 | AMS | (Fecchio *et al*., 2017) |
| KU562353 | -4,983 | -62,13 | *Plasmodium* sp. | *Synallaxis rutilans* | Brazil | Purus River | NA | 455 | AMN | (Fecchio *et al*., 2017) |
| KU562354 | -16,467 | -58,13 | *Plasmodium* sp. | *Pyriglena leuconota* | Brazil | Cáceres | NA | 455 | CSA | (Fecchio *et al*., 2017) |
| KU562355 | -3,7 | -46,75 | *Plasmodium* sp. | *Thamnophilus aethiops* | Brazil | Gurupi | NA | 455 | AMS | (Fecchio *et al*., 2017) |
| KU562356 | -3,7 | -46,75 | *Plasmodium* sp. | *Phlegopsis nigromaculata* | Brazil | Gurupi | NA | 455 | AMS | (Fecchio *et al*., 2017) |
| KU562348 | -5,1 | -56,43 | *Plasmodium* sp. | *Hylophilus ochraceiceps* | Brazil | Jamanxim River | NA | 456 | AMS | (Fecchio *et al*., 2017) |
| KU562284 | -5,217 | -56,917 | *Plasmodium* sp. | *Lepidothrix vilasboasi* | Brazil | Tapajόs River | NA | 457 | AMS | (Fecchio *et al*., 2017) |
| KU562368 | -5,067 | -56,85 | *Plasmodium* sp. | *Pipra fasciicauda* | Brazil | Tapajόs River | NA | 457 | AMS | (Fecchio *et al*., 2017) |
| KU562369 | -5,717 | -63,2 | *Plasmodium* sp. | *Lepidothrix coronata* | Brazil | Purus River | NA | 457 | AMN | (Fecchio *et al*., 2017) |
| KU562370 | -5,717 | -63,2 | *Plasmodium* sp. | *Ceratopipra rubrocapilla* | Brazil | Purus River | NA | 457 | AMN | (Fecchio *et al*., 2017) |
| KU562321 | -5,1 | -56,43 | *Plasmodium* sp. | *Myrmotherula menetriesii* | Brazil | Jamanxim River | NA | 458 | AMS | (Fecchio *et al*., 2017) |
| JQ764619 | 10,231 | -67,285 | *Haemoproteus* sp. | *Geotrygon linearis* | Venezuela | Aragua | HVE2 | 459 | NAN | (Mijares *et al*., 2012) |
| FJ389157 | 4,217 | 9,173 | *Plasmodium multivacuolaris* | *Eurillas latirostris* | Africa | Mount Cameroon | NA | 460 | NA | (Walther *et al*., 2014) |
| JN792136 | 36,778 | -119,418 | *Haemoproteus* sp. | *Catharus ustulatus* | United States of America | California | NA | 461 | NA | (Galen and Witt, 2014) |
| KJ661299 | -1,477 | -78,16 | *Haemoproteus* sp. | *Threnetes niger* | Ecuador | Cumandá | NA | 462 | NAN | (Harrigan *et al*., 2014) |
| KJ661248 | -0,676 | -77,254 | *Haemoproteus* sp. | *Phaethornis malaris* | Ecuador | Loreto | NA | 462 | AMN | (Harrigan *et al*., 2014) |
| KJ661249 | -0,676 | -77,254 | *Haemoproteus* sp. | *Ceratopipra erythrocephala* | Ecuador | Loreto | NA | 462 | AMN | (Harrigan *et al*., 2014) |
| KJ661250 | -0,676 | -77,254 | *Haemoproteus* sp. | *Thamnophilus schistaceus* | Ecuador | Loreto | NA | 462 | AMN | (Harrigan *et al*., 2014) |
| KJ661252 | -0,676 | -77,254 | *Haemoproteus* sp. | *Phaethornis malaris* | Ecuador | Loreto | NA | 462 | AMN | (Harrigan *et al*., 2014) |
| KC680696 | -0,633 | -76,133 | *Plasmodium* sp. | *Hylophylax naevius* | Ecuador | Tiputini Biodiversity Station, Orellana Province | P14 | 463 | AMN | (Svensson-Coelho *et al*. 2016) |
| JQ988255 | -6,649 | -76,072 | *Parahaemoproteus* sp. | *Thalurania furcata* | Peru | San Martín | NA | 464 | CAN | (Galen and Witt, 2014) |
| JQ988257 | -6,649 | -76,072 | *Parahaemoproteus* sp. | *Thalurania furcata* | Peru | San Martín | NA | 464 | CAN | (Galen and Witt, 2014) |
| KJ561806 | -9,190 | -75,015 | *Haemoproteus* sp. | *Spheniscus humboldti* | Peru | NA | NA | 465 | NAN | (Sallaberry-Pincheira *et al*. 2015) |
| DQ241517 | 4,86 | -58,93 | *Plasmodium* sp. | *Streptoprocne zonaris* | Guyana | NA | 10 | 466 | AMN | (Durrant *et al*., 2006) |
| DQ241531 | 4,86 | -58,93 | *Plasmodium* sp. | *Cacicus cela* | Guyana | NA | 24 | 467 | AMN | (Durrant *et al*., 2006) |
| DQ241533 | 4,86 | -58,93 | *Plasmodium* sp. | *Molothrus oryzivorus* | Guyana | NA | 26 | 468 | AMN | (Durrant *et al*., 2006) |
| DQ241533 | 4,86 | -58,93 | *Plasmodium* sp. | *Molothrus oryzivorus* | Guyana | NA | 26 | 468 | AMN | (Durrant *et al*., 2006) |
| AY167248 | 15,439 | -61,346 | *Plasmodium* sp. | *Vireo altiloquus* | Antilles | Lesser Antilles - Dominica Island | PB | 469* | LAN | (Fallon *et al*., 2005; Fallon *et al*., 2003) |
| AY167248 | 13,909 | -60,978 | *Plasmodium* sp. | *Vireo altiloquus* | Antilles | Lesser Antilles - Saint Lucia Island | PB | 469* | LAN | (Fallon *et al*., 2005; Fallon *et al*., 2003) |
| AY455662 | 16,235 | -61,488 | *Plasmodium* sp. | *Turdus plumbeus* | Antilles | NA | PF | 470* | LAN | (Fallon *et al*., 2005) |
| DQ241511 | 4,86 | -58,93 | *Plasmodium* sp. | *Saltator maximus* | Guyana | NA | 4 | 471 | AMN | (Durrant *et al*., 2006) |
| GQ395662 | -0,829 | -90,982 | *Plasmodium* sp. | *Spheniscus mendiculus* | Ecuador | Galápagos Islands | NA | 472 | NAN | (Ricklefs *et al*., 2017) |
| GQ141561 | NA | NA | *Parahaemoproteus* sp. | *Phaenicophilus palmarum* | Antilles | NA | PHAPAL01 | 473 | NA | (Galen and Witt, 2014) |
| GQ141584 | NA | NA | *Parahaemoproteus* sp. | *Turdus migratorius* | North America Continental | NA | TUMIG06 | 474 | NA | (Galen and Witt, 2014) |
| GQ141562 | NA | NA | *Parahaemoproteus* sp. | *Coereba flaveola* | Antilles | NA | COFLA03 | 475 | NA | (Galen and Witt, 2014) |
| AY840997 | 16,235 | -61,488 | *Haemoproteus* sp. | *Sin registro* | Antilles | NA | HJ | 475 | LAN | (Fallon *et al*., 2005) |
| AY841002 | 16,235 | -61,488 | *Apicomplexa* sp. | *Columbina passerina* | Antilles | NA | CPA1 | 475 | LAN | (Fallon *et al*., 2005) |
| AF465583 | NA | NA | *Haemoproteus* sp. | *Piranga olivacea* | North America Continental | NA | PIOLI01 | 476 | NA | (Galen and Witt, 2014) |
| AF465583 | NA | NA | *Haemoproteus* sp. | *Piranga rubra* | North America Continental | NA | PIOLI01 | 476 | NA | (Galen and Witt, 2014) |
| KC680700 | -0,633 | -76,133 | *Haemoproteus* sp. | *Pipra pipra* | Ecuador | Tiputini Biodiversity Station, Orellana Province | H12 | 477 | AMN | (Svensson-Coelho *et al*. 2016) |
| KC680676 | -0,633 | -76,133 | *Plasmodium* sp. | *Hylophylax naevius* | Ecuador | Tiputini Biodiversity Station, Orellana Province | P28 | 478 | AMN | (Svensson-Coelho *et al*. 2016) |
| KC680672 | -0,633 | -76,133 | *Plasmodium* sp. | *Hylophilus ochraceiceps* | Ecuador | Tiputini Biodiversity Station, Orellana Province | P17 | 479 | AMN | (Svensson-Coelho *et al*. 2016) |
| JX501908 | -15,53 | -47,55 | *Plasmodium* sp. | *Neothraupis fasciata* | Brazil | Águas Emendadas Station Ecologic, Distrito Federal | NA | 480 | CSA | (Ricklefs *et al*., 2017) |
| KF537314 | 4,804 | -75,713 | *Haemoproteus columbae* | *Columba livia* | Colombia | Pereira | HAECOL1 | 481 | NAN | (González *et al*., 2015) |
| KC680705 | -0,633 | -76,133 | *Plasmodium* sp. | *Habia rubica* | Ecuador | Tiputini Biodiversity Station, Orellana Province | P43L | 482 | AMN | (Svensson-Coelho *et al*. 2016) |
| AY167249 | 14,609 | -61,072 | *Plasmodium* sp. | *Tiaris bicolor* | Antilles | Lesser Antilles - Martinique Island | PC | 482 | LAN | (Fallon *et al*., 2005; Fallon *et al*., 2003) |
| AY167249 | 14,609 | -61,072 | *Plasmodium* sp. | *Coereba flaveola* | Antilles | Lesser Antilles - Martinique Island | PC | 482 | LAN | (Fallon *et al*., 2005; Fallon *et al*., 2003) |
| AY167249 | 17,075 | -61,817 | *Plasmodium* sp. | *Loxigilla noctis* | Antilles | Lesser Antilles - Antigua Island | PC | 482 | LAN | (Fallon *et al*., 2005; Fallon *et al*., 2003) |
| AY167249 | 13,909 | -60,978 | *Plasmodium* sp. | *Tiaris bicolor* | Antilles | Lesser Antilles - Saint Lucia Island | PC | 482 | LAN | (Fallon *et al*., 2005; Fallon *et al*., 2003) |
| AY167249 | 13,909 | -60,978 | *Plasmodium* sp. | *Loxigilla noctis* | Antilles | Lesser Antilles - Saint Lucia Island | PC | 482 | LAN | (Fallon *et al*., 2005; Fallon *et al*., 2003) |
| AY167249 | 13,909 | -60,978 | *Plasmodium* sp. | *Coereba flaveola* | Antilles | Lesser Antilles - Saint Lucia Island | PC | 482 | LAN | (Fallon *et al*., 2005; Fallon *et al*., 2003) |
| AY167249 | 15,439 | -61,346 | *Plasmodium* sp. | *Tiaris bicolor* | Antilles | Lesser Antilles - Dominica Island | PC | 482 | LAN | (Fallon *et al*., 2005; Fallon *et al*., 2003) |
| AY167249 | 15,439 | -61,346 | *Plasmodium* sp. | *Loxigilla noctis* | Antilles | Lesser Antilles - Dominica Island | PC | 482 | LAN | (Fallon *et al*., 2005; Fallon *et al*., 2003) |
| AY167249 | 16,129 | -61,653 | *Plasmodium* sp. | *Tiaris bicolor* | Antilles | Lesser Antilles - Guadeloupe Island | PC | 482 | LAN | (Fallon *et al*., 2005; Fallon *et al*., 2003) |
| AY167249 | 16,129 | -61,653 | *Plasmodium* sp. | *Loxigilla noctis* | Antilles | Lesser Antilles - Guadeloupe Island | PC | 482 | LAN | (Fallon *et al*., 2005; Fallon *et al*., 2003) |
| AY167249 | 16,129 | -61,653 | *Plasmodium* sp. | *Coereba flaveola* | Antilles | Lesser Antilles - Guadeloupe Island | PC | 482 | LAN | (Fallon *et al*., 2005; Fallon *et al*., 2003) |
| DQ241539 | 4,86 | -58,93 | *Haemoproteus* sp. | *Psarocolius viridis* | Guyana | NA | CHASPI01 | 483 | AMN | (Galen and Witt, 2014; Durrant *et al*., 2006) |
| DQ241539 | 4,86 | -58,93 | *Haemoproteus* sp. | *Chaetura spinicauda* | Guyana | NA | CHASPI01 | 483 | AMN | (Galen and Witt, 2014; Durrant *et al*., 2006) |
| DQ241535 | 4,86 | -58,93 | *Plasmodium* sp. | *Icterus nigrogularis* | Guyana | NA | 28 | 484 | AMN | (Durrant *et al*., 2006) |
| DQ241535 | 4,86 | -58,93 | *Plasmodium* sp. | *Icterus nigrogularis* | Guyana | NA | 28 | 484 | AMN | (Durrant *et al*., 2006) |
| DQ241535 | 4,86 | -58,93 | *Plasmodium* sp. | *Sturnella superciliaris* | Guyana | NA | 28 | 484 | AMN | (Durrant *et al*., 2006) |
| DQ241535 | 4,86 | -58,93 | *Plasmodium* sp. | *Icterus nigrogularis* | Guyana | NA | 28 | 484 | AMN | (Durrant *et al*., 2006) |
| EU328179 | 37,09 | -95,712 | *Haemoproteus* sp. | *Geothlypis trichas* | United States of America | NA | SIAMEX01 | 485 | NA | (Galen and Witt, 2014) |
| EU328178 | 37,09 | -95,712 | *Haemoproteus* sp. | *Geothlypis trichas* | United States of America | NA | GEOTRI04 | 486 | NA | (Galen and Witt, 2014) |
| EU810634 | -0,803 | 11,609 | *Plasmodium* sp. | *Alethe diademata castanea* | Gabón | NA | WA19 | 487 | NA | (Mantilla *et al*., 2013) |
| EF153646 | -0,0013 | -78,355 | *Haemoproteus* sp. | *Zonotrichia capensis* | Ecuador | Quito | ChH1 | 488 | NAN | (Cadena *et al*., 2015) |
| EF153646 | -36,833 | -72,55 | *Haemoproteus* sp. | *Aphrastura spinicauda* | Chile | Pantanillo | ChH1 | 488 | SAN | (Galen and Witt, 2014) |
| KC680719 | -0,633 | -76,133 | *Plasmodium* sp. | *Myrmotherula axillaris* | Ecuador | Tiputini Biodiversity Station, Orellana Province | NA | 489 | AMN | (Ricklefs *et al*., 2017) |
| KC680719 | -0,633 | -76,133 | *Plasmodium* sp. | *Myrmotherula axillaris* | Ecuador | Tiputini Biodiversity Station, Orellana Province | P33L | 489 | AMN | (Svensson-Coelho *et al*. 2016) |
| KC680717 | -0,633 | -76,133 | *Plasmodium* sp. | *Automolus infuscatus* | Ecuador | Tiputini Biodiversity Station, Orellana Province | P22 | 490 | AMN | (Svensson-Coelho *et al*. 2016) |
| EF153653 | -32,835 | -70,701 | *Haemoproteus* sp. | *Phrygilus fruticeti* | Chile | Rinconada | PHALA01 | 491 | SAN | (Galen and Witt, 2014) |
| KU057964 | -23,65 | -46,617 | *Haemoproteus* sp. | *Columba livia* | Brazil | São Paulo Zoo | NA | 492 | ATL | (Chagas *et al*., 2016) |
| KX171624 | -23,65 | -46,617 | *Haemoproteus* sp. | *Eudocimus ruber* | Brazil | São Paulo Zoo | NA | 493 | ATL | (Chagas *et al*., 2017) |
| KX171625 | -23,65 | -46,617 | *Plasmodium* sp. | *Mitu tomentosum* | Brazil | São Paulo Zoo | NA | 494 | ATL | (Chagas *et al*., 2017) |
| KX171625 | -23,65 | -46,617 | *Plasmodium* sp. | *Phoenicopterus chilensis* | Brazil | São Paulo Zoo | NA | 494 | ATL | (Chagas *et al*., 2017) |
| KC680709 | -0,633 | -76,133 | *Plasmodium* sp. | *Hypocnemis hypoxantha* | Ecuador | Tiputini Biodiversity Station, Orellana Province | P7 | 495 | AMN | (Svensson-Coelho *et al*. 2016) |
| KX171623 | -23,65 | -46,617 | *Plasmodium* sp. | *Cereopsis novaehollandiae* | Brazil | São Paulo Zoo | NA | 496 | ATL | (Chagas *et al*., 2017) |
| KC480268 | -0,0013 | -78,355 | *Plasmodium* sp. | *Zonotrichia capensis* | Ecuador | Quito | ZOCAP10 | 497 | NAN | (Cadena *et al*., 2015) |
| KC480268 | -13,937 | -75,8 | *Plasmodium* sp. | *Zonotrichia capensis* | Peru | Ica | ZOCAP10 | 497 | STP | (Jones *et al*., 2013) |
| KC480267 | -0,0013 | -78,355 | *Plasmodium* sp. | *Zonotrichia capensis* | Ecuador | Quito | ZOCAP09 | 498 | NAN | (Cadena *et al*., 2015) |
| KC480267 | -13,937 | -75,8 | *Plasmodium* sp. | *Zonotrichia capensis* | Peru | Ica | ZOCAP09 | 498 | STP | (Jones *et al*., 2013) |
| KF537327 | 4,804 | -75,713 | *Haemoproteus coatneyi* | *Zonotrichia capensis* | Colombia | Pereira | ZOCAP08 | 499 | NAN | (González *et al*., 2015) |
| JN819387 | 9,748 | -83,753 | *Haemoproteus* sp. | *Tangara icterocephala* | Costa Rica | NA | NA | 500 | CDH | (Galen and Witt, 2014) |
| HQ287553 | -10,249 | -48,324 | *Plasmodium* sp. | *Formicivora grisea* | Brazil | Palmas,Tocantins | Toc-16 | 501 | CSA | (Belo *et al*., 2011) |
| MF990727 | 2,967 | -78,184 | *Haemoproteus* sp. | *Sporophila luctuosa* | Colombia | Cauca, PNN Gorgona, El Poblado | NA | 502 | CHO | In this studio |
| JF833042 | 19,313 | -81,255 | *Haemoproteus* sp. | *Fregata magnificens* | Cayman Islands | NA | CY18 | 503 | GAN | (Levin *et al*., 2011) |
| JF833057 | -1,831 | -78,183 | *Haemoproteus multipigmentatus* | *Zenaida galapagoensis* | Ecuador | Tiputini Biodiversity Station, Orellana Province | HMULTIPIGMENTATUS7 | 503 | AMN | (Levin *et al*., 2011) |
| JN792142 | 36,778 | -119,418 | *Haemoproteus* sp. | *Catharus ustulatus* | United States of America | California | NA | 504 | NA | (Galen and Witt, 2014) |
| KT373872 | -2,117 | -77,733 | *Plasmodium* sp. | *Syndactyla ruficollis* | Ecuador | Morona-Santiago Province, Wisui | HYLSUB01 | 505 | AMN | (Moens & Pérez-Tris, 2016) |
| KT373872 | -2,087 | -77,751 | *Plasmodium* sp. | *Automolus subulatus* | Ecuador | Wisui reserve | NA | 505 | AMN | (Moens *et al*., 2017) |
| KT373872 | -2,117 | -77,733 | *Plasmodium* sp. | *Automolus subulatus* | Ecuador | Morona-Santiago Province, Wisui | HYLSUB01 | 505 | AMN | (Moens & Pérez-Tris, 2016) |
| KT373867 | -2,117 | -77,733 | *Plasmodium* sp. | *Xenopipo holochlora* | Ecuador | Morona-Santiago Province, Wisui | LEPCOR02 | 506 | AMN | (Moens & Pérez-Tris, 2016) |
| KT373867 | -2,117 | -77,733 | *Plasmodium* sp. | *Myiobius atricaudus* | Ecuador | Morona-Santiago Province, Wisui | LEPCOR02 | 506 | AMN | (Moens & Pérez-Tris, 2016) |
| KT373867 | -2,087 | -77,751 | *Plasmodium* sp. | *Lepidothrix coronata* | Ecuador | Wisui reserve | NA | 506 | AMN | (Moens *et al*., 2017) |
| KT373867 | -2,117 | -77,733 | *Plasmodium* sp. | *Lepidothrix coronata* | Ecuador | Morona-Santiago Province, Wisui | LEPCOR02 | 506 | AMN | (Moens & Pérez-Tris, 2016) |
| KT373860 | -2,117 | -77,733 | *Haemoproteus* sp. | *Euphonia laniirostris* | Ecuador | Morona-Santiago Province, Wisui | EUXAN01 | 507 | AMN | (Moens & Pérez-Tris, 2016) |
| KT373860 | -2,087 | -77,751 | *Parahaemoproteus* sp. | *Euphonia xanthogaster* | Ecuador | Wisui reserve | NA | 507 | AMN | (Moens *et al*., 2017) |
| KT373860 | -2,117 | -77,733 | *Haemoproteus* sp. | *Euphonia xanthogaster* | Ecuador | Morona-Santiago Province, Wisui | EUXAN01 | 507 | AMN | (Moens & Pérez-Tris, 2016) |
| GU085190 | NA | NA | *Haemoproteus* sp. | *Emberiza cirlus* | Sur America | NA | EMCIR01 | 508 | NA | (Galen and Witt, 2014) |
| JF833062 | -0,829 | -90,982 | *Haemoproteus* sp. | *Sula nebouxii* | Ecuador | Galápagos Islands | SE22F | 508 | NAN | (Levin *et al*., 2011) |
| JF833062 | -0,829 | -90,982 | *Haemoproteus* sp. | *Sula nebouxii* | Ecuador | Galápagos Islands | NA | 508 | NAN | (Lee-Cruz *et al*., 2016) |
| JF833063 | -0,829 | -90,982 | *Haemoproteus* sp. | *Sula nebouxii* | Ecuador | Galápagos Islands | SE26F | 508 | NAN | (Levin *et al*., 2011) |
| JF833063 | -0,829 | -90,982 | *Haemoproteus* sp. | *Sula nebouxii* | Ecuador | Galápagos Islands | NA | 508 | NAN | (Lee-Cruz *et al*., 2016) |
| JF833064 | -0,829 | -90,982 | *Haemoproteus* sp. | *Sula nebouxii* | Ecuador | Galápagos Islands | SE26M | 508 | NAN | (Levin *et al*., 2011) |
| JF833064 | -0,829 | -90,982 | *Haemoproteus* sp. | *Sula nebouxii* | Ecuador | Galápagos Islands | NA | 508 | NAN | (Lee-Cruz *et al*., 2016) |
| JQ988516 | -11,983 | -74,933 | *Parahaemoproteus* sp. | *Diglossa cyanea* | Peru | Junín | NA | 509 | CAN | (Galen and Witt, 2014) |
| JQ988508 | -5,896 | -79,785 | *Parahaemoproteus* sp. | *Atlapetes latinuchus* | Peru | Lambayeque | NA | 510 | CAN | (Galen and Witt, 2014) |
| MF077660 | 35,534 | -107,350 | *Haemoproteus* sp. | *Setophaga graciae* | United States of America | Nuevo Mexico, Mesa Chivato | SETGRA01 | 511 | NA | (Marroquin-Flores *et al*., 2017) |
| JX021458 | -16,11 | -40,022 | *Plasmodium* sp. | *Thamnophilus ambiguus* | Brazil | Salto da Divisa | THAMB02 | 512 | CSA | (Lacorte *et al*., 2013) |
| JX021458 | -19,098 | -40,186 | *Plasmodium* sp. | *Thamnophilus ambiguus* | Brazil | Sooretama | THAMB02 | 512 | ATL | (Lacorte *et al*., 2013) |
| JX021458 | -19,789 | -42,141 | *Plasmodium* sp. | *Thamnophilus ambiguus* | Brazil | Caratinga | THAMB02 | 512 | CSA | (Lacorte *et al*., 2013) |
| JQ988544 | -13,249 | -72,169 | *Parahaemoproteus* sp. | *Amazilia viridicauda* | Peru | Cusco | NA | 513 | CAN | (Galen and Witt, 2014) |
| MF077666 | 34,958 | -107,967 | *Plasmodium* sp. | *Melozone fusca* | United States of America | Nuevo Mexico, El Malpais | MELFUS05 | 514 | NA | (Marroquin-Flores *et al*., 2017) |
| KF767424 | -11,983 | -74,933 | *Haemoproteus* sp. | *Troglodytes aedon* | Peru | Junín | TROAED16 | 515 | CAN | (Galen and Witt, 2014) |
| JQ988305 | -6,104 | -78,341 | *Parahaemoproteus* sp. | *Coeligena coeligena* | Peru | Amazonas | NA | 516 | CAN | (Galen and Witt, 2014) |
| JQ988342 | -13,163 | -74,223 | *Parahaemoproteus* sp. | *Coeligena coeligena* | Peru | Ayacucho | NA | 517 | CAN | (Galen and Witt, 2014) |
| KF767418 | -9,342 | -77,508 | *Haemoproteus* sp. | *Troglodytes aedon* | Peru | Ancash | TROAED14 | 518 | CAN | (Galen and Witt, 2014) |
| AF254975 | NA | NA | *Plasmodium* sp. | *Acrocephalus arundinaceus* | Antilles | NA | GRW4 | 519 | NA | (Fallon *et al*., 2005) |
| AY172842 | -9,916 | -76,233 | *Haemoproteus* sp. | *Carduelis magellanica* | Peru | Huánuco | PYERY01 | 520 | CAN | (Marzal *et al*., 2015) |
| JX029913 | -16,11 | -40,022 | *Haemoproteus* sp. | *Pachyramphus polychopterus* | Brazil | Salto da Divisa | PAPOL04 | 521 | CSA | (Lacorte *et al*., 2013) |
| JX029919 | -16,11 | -40,022 | *Haemoproteus* sp. | *Pitangus sulphuratus* | Brazil | Salto da Divisa | PISUL01 | 522 | CSA | (Lacorte *et al*., 2013) |
| JX029919 | -16,11 | -40,022 | *Haemoproteus* sp. | *Thamnophilus ambiguus* | Brazil | Salto da Divisa | PISUL01 | 522 | CSA | (Lacorte *et al*., 2013) |
| JX029919 | -17,0003 | -46,008 | *Haemoproteus* sp. | *Dendrocolaptes platyrostris* | Brazil | Brasilandia de Minas | PISUL01 | 522 | CSA | (Lacorte *et al*., 2013) |
| JX029916 | -19,82 | -40,276 | *Haemoproteus* sp. | *Tolmomyias flaviventris* | Brazil | Aracruz | TOFLA03 | 523 | ATL | (Lacorte *et al*., 2013) |
| JX021481 | -19,789 | -42,141 | *Plasmodium* sp. | *Conopophaga lineata* | Brazil | Caratinga | COLIN03 | 524 | CSA | (Lacorte *et al*., 2013) |
| JX021468 | -19,82 | -40,276 | *Plasmodium* sp. | *Zonotrichia capensis* | Brazil | Aracruz | TRMEL02 | 525 | ATL | (Lacorte *et al*., 2013) |
| JX021468 | -19,789 | -42,141 | *Plasmodium* sp. | *Trichothraupis melanops* | Brazil | Caratinga | TRMEL02 | 525 | CSA | (Lacorte *et al*., 2013) |
| JX021468 | -19,789 | -42,141 | *Plasmodium* sp. | *Anabazenops fuscus* | Brazil | Caratinga | TRMEL02 | 525 | CSA | (Lacorte *et al*., 2013) |
| JX021468 | -0,0013 | -78,355 | *Plasmodium* sp. | *Zonotrichia capensis* | Ecuador | Quito | TRMEL02 | 525 | NAN | (Cadena *et al*., 2015) |
| JX021468 | -19,82 | -40,276 | *Plasmodium* sp. | *Vireo olivaceus* | Brazil | Aracruz | TRMEL02 | 525 | ATL | (Lacorte *et al*., 2013) |
| JX021496 | -19,993 | -43,848 | *Plasmodium* sp. | *Conopophaga lineata* | Brazil | Nova Lima | COLIN13 | 526 | CSA | (Lacorte *et al*., 2013) |
| JX021497 | -19,993 | -43,848 | *Plasmodium* sp. | *Conopophaga lineata* | Brazil | Nova Lima | COLIN14 | 527 | CSA | (Lacorte *et al*., 2013) |
| JX029864 | -17,111 | -43,82 | *Plasmodium* sp. | *Thamnophilus ambiguus* | Brazil | Bocaiúva | THAMB04 | 528 | CSA | (Lacorte *et al*., 2013) |
| JX029863 | -16,437 | -41,012 | *Plasmodium* sp. | *Coryphospingus pileatus* | Brazil | Jequitinhonha | COPIL02 | 529 | CSA | (Lacorte *et al*., 2013) |
| JX021484 | -19,789 | -42,141 | *Plasmodium* sp. | *Pyriglena leucoptera* | Brazil | Caratinga | PYLEU01 | 530 | CSA | (Lacorte *et al*., 2013) |
| JX021484 | -19,789 | -42,141 | *Plasmodium* sp. | *Dysithamnus plumbeus* | Brazil | Caratinga | PYLEU01 | 530 | CSA | (Lacorte *et al*., 2013) |
| JX021484 | -19,993 | -43,848 | *Plasmodium* sp. | *Pyriglena leucoptera* | Brazil | Nova Lima | PYLEU01 | 530 | CSA | (Lacorte *et al*., 2013) |
| JX021484 | -16,11 | -40,022 | *Plasmodium* sp. | *Pyriglena leucoptera* | Brazil | Salto da Divisa | PYLEU01 | 530 | CSA | (Lacorte *et al*., 2013) |
| JX021484 | -19,098 | -40,186 | *Plasmodium* sp. | *Pyriglena leucoptera* | Brazil | Sooretama | PYLEU01 | 530 | ATL | (Lacorte *et al*., 2013) |
| JX021484 | -17,111 | -43,82 | *Plasmodium* sp. | *Pyriglena leucoptera* | Brazil | Bocaiúva | PYLEU01 | 530 | CSA | (Lacorte *et al*., 2013) |
| JX021486 | -19,993 | -43,848 | *Plasmodium* sp. | *Pyriglena leucoptera* | Brazil | Nova Lima | PYLEU03 | 531 | CSA | (Lacorte *et al*., 2013) |
| KU562542 | -6,602 | -40,124 | *Plasmodium* sp. | *Casiornis fuscus* | Brazil | Aiuaba | NA | 532 | CSA | (Fecchio *et al*., 2017) |
| KU562606 | -0,583 | -64,917 | *Plasmodium* sp. | *Psophia ochroptera* | Brazil | Negro River | NA | 533 | AMN | (Fecchio *et al*., 2017) |
| KU562607 | -0,583 | -64,917 | *Plasmodium* sp. | *Psophia ochroptera* | Brazil | Negro River | NA | 533 | AMN | (Fecchio *et al*., 2017) |
| KU562401 | -4,7 | -56,53 | *Plasmodium* sp. | *Willisornis poecilinotus* | Brazil | Tapajόs River | NA | 534 | AMS | (Fecchio *et al*., 2017) |
| KU562511 | -4,5 | -56,267 | *Plasmodium* sp. | *Thamnophilus nigrocinereus* | Brazil | Tapajόs River | NA | 535 | AMS | (Fecchio *et al*., 2017) |
| KU562499 | -4,5 | -56,283 | *Plasmodium* sp. | *Xiphorhynchus elegans* | Brazil | Tapajόs River | NA | 536 | AMS | (Fecchio *et al*., 2017) |
| KU562766 | -1,35 | -56,367 | *Plasmodium* sp. | *Thamnomanes caesius* | Brazil | Porto Trombetas | NA | 537 | AMN | (Fecchio *et al*., 2017) |
| KU562767 | -1,35 | -56,367 | *Plasmodium* sp. | *Thamnomanes caesius* | Brazil | Porto Trombetas | NA | 537 | AMN | (Fecchio *et al*., 2017) |
| KU562738 | -3,7 | -46,75 | *Plasmodium* sp. | *Thraupis episcopus* | Brazil | Gurupi | NA | 538 | AMS | (Fecchio *et al*., 2017) |
| KU562737 | -3,7 | -46,75 | *Plasmodium* sp. | *Ramphocelus carbo* | Brazil | Gurupi | NA | 538 | AMS | (Fecchio *et al*., 2017) |
| KU562837 | -12,567 | -70,083 | *Plasmodium* sp. | *Saltator maximus* | Peru | Manu | NA | 539 | CAN | (Fecchio *et al*., 2017) |
| KU562796 | -9,283 | -64,73 | *Plasmodium* sp. | *Gymnopithys salvini* | Brazil | Porto Velho | NA | 540 | AMS | (Fecchio *et al*., 2017) |
| KU562799 | -9,283 | -64,717 | *Plasmodium* sp. | *Lepidothrix coronata* | Brazil | Porto Velho | NA | 541 | AMS | (Fecchio *et al*., 2017) |
| KU562789 | -9,33 | -64,67 | *Plasmodium* sp. | *Arremon taciturnus* | Brazil | Porto Velho | NA | 542 | AMS | (Fecchio *et al*., 2017) |
| KU562812 | -12,567 | -70,083 | *Plasmodium* sp. | *Pipra fasciicauda* | Peru | Manu | NA | 543 | CAN | (Fecchio *et al*., 2017) |
| KU562804 | -9,283 | -64,73 | *Plasmodium* sp. | *Myrmoborus myotherinus* | Brazil | Porto Velho | NA | 544 | AMS | (Fecchio *et al*., 2017) |
| KU562664 | -5,717 | -63,2 | *Plasmodium* sp. | *Willisornis poecilinotus* | Brazil | Purus River | NA | 545 | AMN | (Fecchio *et al*., 2017) |
| KU562714 | -3,7 | -46,75 | *Plasmodium* sp. | *Thamnomanes caesius* | Brazil | Gurupi | NA | 546 | AMS | (Fecchio *et al*., 2017) |
| KU562715 | -3,7 | -46,75 | *Plasmodium* sp. | *Thamnomanes caesius* | Brazil | Gurupi | NA | 546 | AMS | (Fecchio *et al*., 2017) |
| KU562705 | -6,582 | -37,267 | *Plasmodium* sp. | *Coryphospingus pileatus* | Brazil | Serra Negra do Norte | NA | 547 | ATL | (Fecchio *et al*., 2017) |
| KU562684 | -19,567 | -57,017 | *Plasmodium* sp. | *Paroaria capitata* | Brazil | Corumbá | NA | 548 | CSA | (Fecchio *et al*., 2017) |
| KU562197 | -15,53 | -47,55 | *Haemoproteus* sp. | *Cypsnagra hirundinacea* | Brazil | Planaltina | NA | 549 | CSA | (Fecchio *et al*., 2017) |
| KU562249 | -12,567 | -70,083 | *Haemoproteus* sp. | *Automolus ochrolaemus* | Peru | Manu | NA | 550 | CAN | (Fecchio *et al*., 2017) |
| KU562255 | -5,067 | -56,85 | *Plasmodium* sp. | *Cantorchilus leucotis* | Brazil | Tapajόs River | NA | 551 | AMS | (Fecchio *et al*., 2017) |
| KU562256 | -5,067 | -56,85 | *Plasmodium* sp. | *Pheugopedius genibarbis* | Brazil | Tapajόs River | NA | 551 | AMS | (Fecchio *et al*., 2017) |
| KU562257 | -4,5 | -56,267 | *Plasmodium* sp. | *Cantorchilus leucotis* | Brazil | Tapajόs River | NA | 551 | AMS | (Fecchio *et al*., 2017) |
| KU562258 | -5,067 | -56,85 | *Plasmodium* sp. | *Myiarchus ferox* | Brazil | Tapajόs River | NA | 551 | AMS | (Fecchio *et al*., 2017) |
| KU562225 | -6,582 | -37,267 | *Haemoproteus* sp. | *Coryphospingus pileatus* | Brazil | Serra Negra do Norte | NA | 552 | ATL | (Fecchio *et al*., 2017) |
| KU562144 | -4,5 | -56,283 | *Haemoproteus* sp. | *Isleria hauxwelli* | Brazil | Tapajόs River | NA | 553 | AMS | (Fecchio *et al*., 2017) |
| KU562147 | -4,683 | -56,63 | *Haemoproteus* sp. | *Thamnomanes caesius* | Brazil | Tapajόs River | NA | 554 | AMS | (Fecchio *et al*., 2017) |
| KU562120 | -5,066 | -56,851 | *Haemoproteus* sp. | *Cyanocompsa cyanoides* | Brazil | Tapajόs River | NA | 555 | AMS | (Fecchio *et al*., 2017) |
| KU562135 | -4,7 | -56,63 | *Haemoproteus* sp. | *Phlegopsis nigromaculata* | Brazil | Tapajόs River | NA | 556 | AMS | (Fecchio *et al*., 2017) |
| KU562159 | -4,5 | -56,283 | *Haemoproteus* sp. | *Thamnomanes saturninus* | Brazil | Tapajόs River | NA | 557 | AMS | (Fecchio *et al*., 2017) |
| KU562367 | -4,7 | -56,53 | *Plasmodium* sp. | *Myrmoborus myotherinus* | Brazil | Tapajόs River | NA | 558 | AMS | (Fecchio *et al*., 2017) |
| KX130088 | 10,829 | -73,692 | *Plasmodium* sp. | *Turdus olivater* | Colombia | Sierra Nevada de Santa Marta, San Lorenzo ridge | NA | 559 | NAN | (Gonzalez-Quevedo, Rivera-Gutierrez & Pabón, 2016) |
| JF833052 | -0,829 | -90,982 | *Haemoproteus multipigmentatus* | *Zenaida galapagoensis* | Ecuador | Galápagos Islands | HMULTIPIGMENTATUS2 | 560 | NAN | (Levin *et al*., 2011) |
| JF833053 | -0,829 | -90,982 | *Haemoproteus multipigmentatus* | *Zenaida galapagoensis* | Ecuador | Galápagos Islands | HMULTIPIGMENTATUS3 | 560 | NAN | (Levin *et al*., 2011) |
| JX073258 | 38,837 | -120,895 | *Haemoproteus sacharovi* | *Zenaida macroura* | United States of America | Northern California | MODO1 | 561 | NA | (Galen and Witt, 2014) |
| KJ145051 | 10,829 | -73,692 | *Plasmodium* sp. | *Arremon basilicus* | Colombia | Sierra Nevada de Santa Marta, San Lorenzo ridge | NA | 562 | NAN | (Gonzalez-Quevedo, Rivera-Gutierrez & Pabón, 2016) |
| KJ661306 | -0,676 | -77,254 | *Haemoproteus* sp. | *Phaethornis malaris* | Ecuador | Loreto | NA | 563 | AMN | (Harrigan *et al*., 2014) |
| HQ287555 | -10,249 | -48,324 | *Plasmodium* sp. | *Turdus leucomelas* | Brazil | Palmas,Tocantins | Toc-21 | 564 | CSA | (Belo *et al*., 2011) |
| KC680675 | -0,633 | -76,133 | *Haemoproteus* sp. | *Myrmoborus myotherinus* | Ecuador | Tiputini Biodiversity Station, Orellana Province | H5 | 565 | AMN | (Svensson-Coelho *et al*. 2016) |
| HQ287554 | -10,249 | -48,324 | *Plasmodium* sp. | *Ramphocelus carbo* | Brazil | Palmas,Tocantins | Toc-28 | 566 | CSA | (Belo *et al*., 2011) |
| DQ241516 | -32,522 | -55,765 | *Plasmodium* sp. | *Pseudoleistes virescens* | Uruguay | NA | 9 | 567 | PAM | (Durrant *et al*., 2006) |
| AY167241 | 13,251 | -61,186 | *Haemoproteus* sp. | *Vireo altiloquus* | Antilles | Lesser Antilles - Saint Vincent Island | HU1 | 568* | LAN | (Fallon *et al*., 2005; Fallon *et al*., 2003) |
| DQ241551 | -32,522 | -55,765 | *Haemoproteus* sp. | *Tachycineta leucorrhoa* | Uruguay | NA | 44 | 569 | PAM | (Durrant *et al*., 2006) |
| DQ241518 | -32,522 | -55,765 | *Plasmodium* sp. | *Aramides ypecaha* | Uruguay | NA | 11 | 570 | PAM | (Durrant *et al*., 2006) |
| KF767430 | -7,161 | -78,512 | *Leucocytozoon* sp. | *Troglodytes aedon* | Peru | Cajamarca | TROAED09 | 571 | CAN | (Galen and Witt, 2014) |
| MF077683 | 34,836 | -108,215 | *Leucocytozoon* sp. | *Piranga flava* | United States of America | Nuevo Mexico, El Malpais | PIRFLA02 | 571 | NA | (Marroquin-Flores *et al*., 2017) |
| KF767440 | -17,39 | -70,345 | *Leucocytozoon* sp. | *Troglodytes aedon* | Peru | Tacna | TROAED05 | 572 | CAN | (Galen and Witt, 2014) |
| KF717065 | 4,683 | -73,833 | *Leucocytozoon* sp. | *Atlapetes pallidinucha* | Colombia | Chingaza NNP, Palacio Forest | L_ATPA_01 | 573 | NAN | (Bensch *et al*., 2009) |
| KF717062 | 4,683 | -73,833 | *Leucocytozoon* sp. | *Hemispingus verticalis* | Colombia | Chingaza NNP, Palacio Forest | HEVE01 | 573 | NAN | (Bensch *et al*., 2009; Lotta *et al*., 2016) |
| KF717062 | 4,717 | -75,45 | *Leucocytozoon* sp. | *Hemispingus verticalis* | Colombia | Los Nevados National Natural Park, El Bosque station | HEVE01 | 573 | NAN | (Lotta *et al*., 2016) |
| KF717066 | 4,57 | -74,297 | *Leucocytozoon* sp. | *Diglossa cyanea* | Colombia | NA | L_DICYA_01 | 574 | NAN | (Bensch *et al*., 2009) |
| KF699312 | 4,683 | -73,833 | *Leucocytozoon* sp. | *Mecocerculus leucophrys* | Colombia | Chingaza NNP, Palacio Forest | METYR01 | 574 | NAN | (Bensch *et al*., 2009; Lotta *et al*., 2016) |
| KF699312 | 4,683 | -73,833 | *Leucocytozoon* sp. | *Arremon torquatus* | Colombia | Chingaza NNP, Palacio Forest | METYR01 | 574 | NAN | (Bensch *et al*., 2009; Lotta *et al*., 2016) |
| KF699312 | 4,683 | -73,833 | *Leucocytozoon* sp. | *Metallura tyrianthina* | Colombia | Chingaza NNP, Palacio Forest | METYR01 | 574 | NAN | (Bensch *et al*., 2009; Lotta *et al*., 2016) |
| KF699312 | 4,617 | -73,717 | *Leucocytozoon* sp. | *Arremon torquatus* | Colombia | Chingaza NNP, Monter Redondo Station | METHY01 | 574 | NAN | (Lotta *et al*., 2016) |
| KF699312 | 4,617 | -73,717 | *Leucocytozoon* sp. | *Metallura tyrianthinae* | Colombia | Chingaza NNP, Monter Redondo Station | METHY01 | 574 | NAN | (Lotta *et al*., 2016) |
| KF699312 | 4,617 | -73,717 | *Leucocytozoon* sp. | *Mecocerculus leucophrys* | Colombia | Chingaza NNP, Monter Redondo Station | METHY01 | 574 | NAN | (Lotta *et al*., 2016) |
| KF699313 | 4,683 | -73,833 | *Leucocytozoon majoris* | *Turdus fuscater* | Colombia | Chingaza NNP, Palacio Forest | TFUS11 | 574 | NAN | (Bensch *et al*., 2009; Lotta *et al*., 2016) |
| KF699313 | 4,616 | -73,722 | *Leucocytozoon majoris* | *Turdus fuscater* | Colombia | Chingaza NNP, Monter Redondo Station | TFUS11 | 574 | NAN | (Bensch *et al*., 2009; Lotta *et al*., 2016) |
| KF717066 | 4,683 | -73,833 | *Leucocytozoon* sp. | *Diglossa cyanea* | Colombia | Chingaza NNP, Palacio Forest | DICYA01 | 574 | NAN | (Lotta *et al*., 2016) |
| MF077681 | 35,533 | -107,349 | *Leucocytozoon* sp. | *Vireo plumbeus* | United States of America | Nuevo Mexico, Mesa Chivato | VIRPLU05 | 575 | NA | (Marroquin-Flores *et al*., 2017) |
| MF077681 | 35,827 | -106,896 | *Leucocytozoon* sp. | *Vireo plumbeus* | United States of America | Nuevo Mexico, Elk Springs | VIRPLU05 | 575 | NA | (Marroquin-Flores *et al*., 2017) |
| KF309188 | 4,683 | -73,833 | *Leucocytozoon quynzae* | *Heliangelus amethysticollis* | Colombia | Chingaza NNP, Palacio Forest | HELIAM01 | 576* | NAN | (Bensch *et al*., 2009; Lotta *et al*., 2016) |
| KF309188 | 4,683 | -73,833 | *Leucocytozoon quynzae* | *Metallura tyrianthina* | Colombia | Chingaza NNP, Palacio Forest | HELIAM01 | 576* | NAN | (Bensch *et al*., 2009; Lotta *et al*., 2016) |
| KF309189 | 4,617 | -73,717 | *Leucocytozoon quynzae* | *Coeligena helianthea* | Colombia | Chingaza NNP, Monter Redondo Station | COHEL01 | 577 | NAN | (Bensch *et al*., 2009; Lotta *et al*., 2016) |
| MF077685 | 35,533 | -107,346 | *Leucocytozoon* sp. | *Nucifraga columbiana* | United States of America | Nuevo Mexico, Mesa Chivato | NUCCOL01 | 578 | NA | (Marroquin-Flores *et al*., 2017) |
| MF077690 | 34,834 | -108,225 | *Leucocytozoon* sp. | *Piranga flava* | United States of America | Nuevo Mexico, El Malpais | PIRFLA03 | 579 | NA | (Marroquin-Flores *et al*., 2017) |
| MF077686 | 35,533 | -107,350 | *Leucocytozoon* sp. | *Setophaga graciae* | United States of America | Nuevo Mexico, Mesa Chivato | SETGRA02 | 579 | NA | (Marroquin-Flores *et al*., 2017) |
| KF717054 | 4,717 | -75,45 | *Leucocytozoon* sp. | *Zonotrichia capensis* | Colombia | Los Nevados National Natural Park, El Bosque station | ZOCAP07 | 580 | NAN | (Bensch *et al*., 2009; Lotta *et al*., 2016) |
| EF153656 | -33,408 | -70,567 | *Leucocytozoon* sp. | *Aphrastura spinicauda* | Chile | Navarino | APSPI05 | 580 | SAN | (Martínez *et al*., 2015) |
| EF153656 | -33,408 | -70,567 | *Leucocytozoon* sp. | *Carduelis barbata* | Chile | Navarino | APSPI05 | 580 | SAN | (Martínez *et al*., 2015) |
| EF153656 | -33,408 | -70,567 | *Leucocytozoon* sp. | *Curaeus curaeus* | Chile | Navarino | APSPI05 | 580 | SAN | (Martínez *et al*., 2015) |
| EF153656 | -33,408 | -70,567 | *Leucocytozoon* sp. | *Phrygilus patagonicus* | Chile | Navarino | APSPI05 | 580 | SAN | (Martínez *et al*., 2015) |
| EF153656 | -33,408 | -70,567 | *Leucocytozoon* sp. | *Troglodytes musculus* | Chile | Navarino | APSPI05 | 580 | SAN | (Martínez *et al*., 2015) |
| EF153656 | -33,408 | -70,567 | *Leucocytozoon* sp. | *Anairetes fernandezianus* | Chile | Navarino | APSPI05 | 580 | SAN | (Martínez *et al*., 2015) |
| EF153656 | -33,408 | -70,567 | *Leucocytozoon* sp. | *Zonotrichia capensis* | Chile | Navarino | APSPI05 | 580 | SAN | (Martínez *et al*., 2015) |
| KF717054 | 4,717 | -75,45 | *Leucocytozoon* sp. | *Catamenia inornata* | Colombia | Los Nevados National Natural Park, El Bosque station | ZOCAP07 | 580 | NAN | (Lotta *et al*., 2016) |
| KF717060 | 4,57 | -74,297 | *Leucocytozoon* sp. | *Zonotrichia capensis* | Colombia | NA | L_ZOCAP_08 | 580 | NAN | (Bensch *et al*., 2009) |
| KF717060 | 4,683 | -73,833 | *Leucocytozoon* sp. | *Hemispingus superciliaris* | Colombia | Chingaza NNP, Palacio Forest | ZOCAP08 | 580 | NAN | (Lotta *et al*., 2016) |
| KF717060 | 4,683 | -73,833 | *Leucocytozoon* sp. | *Catamenia inornata* | Colombia | Chingaza NNP, Palacio Forest | ZOCAP08 | 580 | NAN | (Lotta *et al*., 2016) |
| KF717060 | 4,683 | -73,833 | *Leucocytozoon* sp. | *Zonotrichia capensis* | Colombia | Chingaza NNP, Palacio Forest | ZOCAP08 | 580 | NAN | (Lotta *et al*., 2016) |
| KF717060 | 4,717 | -75,45 | *Leucocytozoon* sp. | *Hemispingus superciliaris* | Colombia | Los Nevados National Natural Park, El Bosque station | ZOCAP08 | 580 | NAN | (Lotta *et al*., 2016) |
| KF717060 | 4,717 | -75,45 | *Leucocytozoon* sp. | *Catamenia inornata* | Colombia | Los Nevados National Natural Park, El Bosque station | ZOCAP08 | 580 | NAN | (Lotta *et al*., 2016) |
| KF717060 | 4,717 | -75,45 | *Leucocytozoon* sp. | *Zonotrichia capensis* | Colombia | Los Nevados National Natural Park, El Bosque station | ZOCAP08 | 580 | NAN | (Lotta *et al*., 2016) |
| KF767426 | -9,342 | -77,508 | *Leucocytozoon* sp. | *Troglodytes aedon* | Peru | Ancash | TROAED01 | 580 | CAN | (Galen and Witt, 2014) |
| KF767431 | -9,018 | -77,539 | *Leucocytozoon* sp. | *Troglodytes aedon* | Peru | Ancash | TROAED02 | 580 | CAN | (Galen and Witt, 2014) |
| KF767432 | -14,06 | -73,008 | *Leucocytozoon* sp. | *Troglodytes aedon* | Peru | Apurímac | TROAED02 | 580 | CAN | (Galen and Witt, 2014) |
| KF767433 | -9,342 | -77,508 | *Leucocytozoon* sp. | *Troglodytes aedon* | Peru | Ancash | NA | 580 | CAN | (Galen and Witt, 2014) |
| KF767434 | -9,342 | -77,508 | *Leucocytozoon* sp. | *Troglodytes aedon* | Peru | Ancash | TROAED01 | 580 | CAN | (Galen and Witt, 2014) |
| KF767435 | -13,249 | -72,169 | *Leucocytozoon* sp. | *Troglodytes aedon* | Peru | Yanah | TROAED01 | 580 | CAN | (Galen and Witt, 2014) |
| KF767436 | -7,161 | -78,512 | *Leucocytozoon* sp. | *Troglodytes aedon* | Peru | Cajamarca | TROAED02 | 580 | CAN | (Galen and Witt, 2014) |
| KF717059 | 4,717 | -75,45 | *Leucocytozoon* sp. | *Myioborus ornatus* | Colombia | Los Nevados National Natural Park, El Bosque station | MYIOR01 | 581 | NAN | (Bensch *et al*., 2009; Lotta *et al*., 2016) |
| KF717050 | 4,57 | -74,297 | *Leucocytozoon* sp. | *Catamenia inornata* | Colombia | NA | L_CINOR_01 | 582 | NAN | (Bensch *et al*., 2009) |
| KF717050 | 4,717 | -75,45 | *Leucocytozoon* sp. | *Catamenia inornata* | Colombia | Los Nevados National Natural Park, El Bosque station | CINOR01 | 582 | NAN | (Lotta *et al*., 2016) |
| KJ661323 | -2,887 | -79,427 | *Leucocytozoon* sp. | *Diglossa cyanea* | Ecuador | Chuacha | NA | 582 | NAN | (Harrigan *et al*., 2014) |
| KF717047 | 4,7 | -75,483 | *Leucocytozoon* sp. | *Atlapetes albinucha* | Colombia | Ucumarí Regional Natural Park | MYFUM01 | 583 | NAN | (Lotta *et al*., 2016) |
| JQ988120 | 10,829 | -73,692 | *Leucocytozoon* sp. | *Arremon basilicus* | Colombia | Sierra Nevada de Santa Marta, San Lorenzo ridge | NA | 583 | NAN | (Gonzalez-Quevedo, Rivera-Gutierrez & Pabón, 2016) |
| JQ988120 | 10,829 | -73,692 | *Leucocytozoon* sp. | *Diglossa humeralis* | Colombia | Sierra Nevada de Santa Marta, San Lorenzo ridge | NA | 583 | NAN | (Gonzalez-Quevedo, Rivera-Gutierrez & Pabón, 2016) |
| JQ988120 | 10,829 | -73,692 | *Leucocytozoon* sp. | *Atlapetes melanocephalus* | Colombia | Sierra Nevada de Santa Marta, San Lorenzo ridge | NA | 583 | NAN | (Gonzalez-Quevedo, Rivera-Gutierrez & Pabón, 2016) |
| KF717047 | 4,57 | -74,297 | *Leucocytozoon* sp. | *Myiotheretes fumigatus* | Colombia | NA | L_MYOFU_01 | 583 | NAN | (Bensch *et al*., 2009) |
| KF717047 | 4,617 | -73,717 | *Leucocytozoon* sp. | *Myiotheretes fumigatus* | Colombia | Chingaza NNP, Monter Redondo Station | MYFUM01 | 583 | NAN | (Lotta *et al*., 2016) |
| KF717047 | 4,717 | -75,45 | *Leucocytozoon* sp. | *Atlapetes schistaceus* | Colombia | Los Nevados National Natural Park, El Bosque station | MYFUM01 | 583 | NAN | (Lotta *et al*., 2016) |
| KF717047 | 4,717 | -75,45 | *Leucocytozoon* sp. | *Diglossa albilatera* | Colombia | Los Nevados National Natural Park, El Bosque station | MYFUM01 | 583 | NAN | (Lotta *et al*., 2016) |
| KF717048 | 4,683 | -73,833 | *Leucocytozoon* sp. | *Buthraupis montana* | Colombia | Chingaza NNP, Palacio Forest | BUTMO01 | 583 | NAN | (Lotta *et al*., 2016) |
| KF962962 | 4,717 | -75,45 | *Leucocytozoon* sp. | *Hemispingus superciliaris* | Colombia | Los Nevados National Natural Park, El Bosque station | HESUP01 | 583 | NAN | (Lotta *et al*., 2016) |
| KF717052 | 4,717 | -75,45 | *Leucocytozoon* sp. | *Hemispingus atropileus* | Colombia | Los Nevados National Natural Park, El Bosque station | HATR01 | 584 | NAN | (Bensch *et al*., 2009; Lotta *et al*., 2016) |
| KF717052 | 4,717 | -75,45 | *Leucocytozoon* sp. | *Hemispingus superciliaris* | Colombia | Los Nevados National Natural Park, El Bosque station | HATR01 | 584 | NAN | (Lotta *et al*., 2016) |
| KF767442 | -13,555 | -70,875 | *Leucocytozoon* sp. | *Troglodytes aedon* | Peru | Cusco | TROAED10 | 584 | CAN | (Galen and Witt, 2014) |
| KF717051 | 4,57 | -74,297 | *Leucocytozoon* sp. | *Arremon brunneinucha* | Colombia | NA | L_ARBRU_01 | 585 | NAN | (Bensch *et al*., 2009) |
| KF717051 | 4,6 | -73,717 | *Leucocytozoon* sp. | *Arremon brunneinucha* | Colombia | Chingaza NNP, Encenillo Forest | ARBRU01 | 585 | NAN | (Lotta *et al*., 2016) |
| KF717051 | 4,6 | -73,717 | *Leucocytozoon* sp. | *Arremon brunneinucha* | Colombia | Chingaza NNP, Encenillo Forest | ARBRU01 | 585 | NAN | (Lotta *et al*., 2016) |
| KJ661320 | -3,003 | -78,51 | *Leucocytozoon* sp. | *Diglossa cyanea* | Ecuador | Cerro Bosco | NA | 586 | NAN | (Harrigan *et al*., 2014) |
| KF767427 | -5,896 | -79,785 | *Leucocytozoon* sp. | *Troglodytes aedon* | Peru | Olmos | TROAED08 | 587 | CAN | (Galen and Witt, 2014) |
| KF699310 | 4,617 | -73,717 | *Leucocytozoon* sp. | *Turdus fuscater* | Colombia | Chingaza NNP, Monter Redondo Station | TFUS12 | 587 | NAN | (Bensch *et al*., 2009; Lotta *et al*., 2016) |
| KF717056 | 4,717 | -75,45 | *Leucocytozoon* sp. | *Turdus fuscater* | Colombia | Los Nevados National Natural Park, El Bosque station | TFUS10 | 587 | NAN | (Bensch *et al*., 2009; Lotta *et al*., 2016) |
| KJ527075 | -9,190 | -75,015 | *Leucocytozoon* sp. | *Oxyura jamaicensis* | Peru | NA | NA | 588 | CAN | (Smith and Ramey 2015) |
| KJ527074 | -9,190 | -75,015 | *Leucocytozoon* sp. | *Oxyura jamaicensis* | Peru | NA | NA | 588 | CAN | (Smith and Ramey 2015) |
| JX984671 | -33,641 | -78,846 | *Leucocytozoon* sp. | *Turdus falcklandii* | Chile | Robinson Crusoe Island | TUFAL04 | 589 | STP | (Martínez *et al*., 2015) |
| JX984672 | -33,641 | -78,846 | *Leucocytozoon* sp. | *Turdus falcklandii* | Chile | Robinson Crusoe Island | TUFAL05 | 589 | STP | (Martínez *et al*., 2015) |
| EU627802 | 10,829 | -73,692 | *Leucocytozoon* sp. | *Vireo leucophrys* | Colombia | Sierra Nevada de Santa Marta, San Lorenzo ridge | NA | 590 | NAN | (Gonzalez-Quevedo, Rivera-Gutierrez & Pabón, 2016) |
| EU627802 | 10,829 | -73,692 | *Leucocytozoon* sp. | *Turdus flavipes* | Colombia | Sierra Nevada de Santa Marta, San Lorenzo ridge | NA | 590 | NAN | (Gonzalez-Quevedo, Rivera-Gutierrez & Pabón, 2016) |
| EU627802 | 10,829 | -73,692 | *Leucocytozoon* sp. | *Myioborus flavivertex* | Colombia | Sierra Nevada de Santa Marta, San Lorenzo ridge | NA | 590 | NAN | (Gonzalez-Quevedo, Rivera-Gutierrez & Pabón, 2016) |
| MF077679 | 34,958 | -107,967 | *Leucocytozoon* sp. | *Vireo plumbeus* | United States of America | Nuevo Mexico, El Malpais | COLBF21 | 590 | NA | (Marroquin-Flores *et al*., 2017) |
| MF077679 | 35,827 | -106,896 | *Leucocytozoon* sp. | *Baeolophus ridgwayi* | United States of America | Nuevo Mexico, Elk Springs | COLBF21 | 590 | NA | (Marroquin-Flores *et al*., 2017) |
| MF077679 | 34,805 | -108,207 | *Leucocytozoon* sp. | *Piranga flava* | United States of America | Nuevo Mexico, El Malpais | COLBF21 | 590 | NA | (Marroquin-Flores *et al*., 2017) |
| MF077679 | 34,805 | -108,209 | *Leucocytozoon* sp. | *Poecile gambeli* | United States of America | Nuevo Mexico, El Malpais | COLBF21 | 590 | NA | (Marroquin-Flores *et al*., 2017) |
| MF077679 | 35,533 | -107,348 | *Leucocytozoon* sp. | *Vireo plumbeus* | United States of America | Nuevo Mexico, Mesa Chivato | COLBF21 | 590 | NA | (Marroquin-Flores *et al*., 2017) |
| AY393796 | 55,169 | 23,881 | *Leucocytozoon* sp. | *Carduelis spinus* | Lithuanian | NA | NA | 591 | _ | (Walther *et al*., 2014) |
| JQ988531 | -11,983 | -74,933 | *Leucocytozoon* sp. | *Troglodytes aedon* | Peru | Junín | TROAED06 | 592 | CAN | (Galen and Witt, 2014) |
| EF153665 | 10,829 | -73,692 | *Leucocytozoon* sp. | *Anabacerthia striaticollis* | Colombia | Sierra Nevada de Santa Marta, San Lorenzo ridge | NA | 592 | NAN | (Gonzalez-Quevedo, Rivera-Gutierrez & Pabón, 2016) |
| EF153665 | 10,829 | -73,692 | *Leucocytozoon* sp. | *Mionectes olivaceus* | Colombia | Sierra Nevada de Santa Marta, San Lorenzo ridge | NA | 592 | NAN | (Gonzalez-Quevedo, Rivera-Gutierrez & Pabón, 2016) |
| KF767437 | -11,767 | -76,528 | *Leucocytozoon* sp. | *Troglodytes aedon* | Peru | Lima | TROAED06 | 592 | CAN | (Galen and Witt, 2014) |
| KF767438 | -14,173 | -73,323 | *Leucocytozoon* sp. | *Troglodytes aedon* | Peru | Apurímac | TROAED07 | 592 | CAN | (Galen and Witt, 2014) |
| KF767441 | -11,767 | -76,528 | *Leucocytozoon* sp. | *Troglodytes aedon* | Peru | Lima | TROAED06 | 592 | CAN | (Galen and Witt, 2014) |
| MF077688 | 35,827 | -106,896 | *Leucocytozoon* sp. | *Cyanocitta stelleri* | United States of America | Nuevo Mexico, Elk Springs | CYASTE07 | 593 | NA | (Marroquin-Flores *et al*., 2017) |
| MF077682 | 35,827 | -106,896 | *Leucocytozoon* sp. | *Cyanocitta stelleri* | United States of America | Nuevo Mexico, Elk Springs | CYASTE04 | 593 | NA | (Marroquin-Flores *et al*., 2017) |
| MF077680 | 35,570 | -107,244 | *Leucocytozoon* sp. | *Aphelocoma woodhouseii* | United States of America | Nuevo Mexico, Mesa Chivato | APHWOO01 | 594 | NA | (Marroquin-Flores *et al*., 2017) |
| MF077689 | 34,958 | -107,969 | *Leucocytozoon* sp. | *Aphelocoma woodhouseii* | United States of America | Nuevo Mexico, El Malpais | APHCAL01 | 594 | NA | (Marroquin-Flores *et al*., 2017) |
| JQ764623 | 10,231 | -67,285 | *Leucocytozoon* sp. | *Seiurus noveboracensis* | Venezuela | Aragua | LVE6 | 595 | NAN | (Mijares *et al*., 2012) |
| MF077687 | 35,526 | -107,387 | *Leucocytozoon* sp. | *Vireo gilvus* | United States of America | Nuevo Mexico, Mesa Chivato | VIGIL02 | 595 | NA | (Marroquin-Flores *et al*., 2017) |
| MF077684 | 35,535 | -107,350 | *Leucocytozoon* sp. | *Setophaga coronata* | United States of America | Nuevo Mexico, Mesa Chivato | CNEORN01 | 596 | NA | (Marroquin-Flores *et al*., 2017) |
| MF077684 | 35,533 | -107,350 | *Leucocytozoon* sp. | *Spizella passerina* | United States of America | Nuevo Mexico, Mesa Chivato | CNEORN01 | 596 | NA | (Marroquin-Flores *et al*., 2017) |
| KF717055 | 4,717 | -75,45 | *Leucocytozoon* sp. | *Turdus fuscater* | Colombia | Los Nevados National Natural Park, El Bosque station | TFUS09 | 597 | NAN | (Bensch *et al*., 2009; Lotta *et al*., 2016) |
| JQ815435 | 4,617 | -73,717 | *Leucocytozoon fringillinarum* | *Turdus fuscater* | Colombia | Chingaza NNP, Monter Redondo Station | TFUS04 | 597 | NAN | (Lotta *et al*., 2013) |
| JQ815435 | 4,617 | -73,717 | *Leucocytozoon* sp. | *Turdus fuscater* | Colombia | Chingaza NNP, Monter Redondo Station | TFUS04 | 597 | NAN | (Lotta *et al*., 2016) |
| KF717053 | 4,717 | -75,45 | *Leucocytozoon* sp. | *Turdus fuscater* | Colombia | Los Nevados National Natural Park, El Bosque station | TFUS08 | 597 | NAN | (Bensch *et al*., 2009; Lotta *et al*., 2016) |
| KF717058 | 4,717 | -75,45 | *Leucocytozoon* sp. | *Hemispingus superciliaris* | Colombia | Los Nevados National Natural Park, El Bosque station | HESUP02 | 598 | NAN | (Bensch *et al*., 2009; Lotta *et al*., 2016) |
| KF717058 | 4,767 | -75,45 | *Leucocytozoon* sp. | *Hemispingus superciliaris* | Colombia | Otun Lagoon | HESUP02 | 598 | NAN | (Bensch *et al*., 2009; Lotta *et al*., 2016) |
| KJ661321 | 0,655 | -78,032 | *Leucocytozoon* sp. | *Diglossa cyanea* | Ecuador | Guandera | NA | 599 | NAN | (Harrigan *et al*., 2014) |
| JQ815434 | 4,617 | -73,717 | *Leucocytozoon fringillinarum* | *Turdus fuscater* | Colombia | Chingaza NNP, Monter Redondo Station | TFUS03 | 600 | NAN | (Lotta *et al*., 2013) |
| KX130090 | 10,829 | -73,692 | *Leucocytozoon* sp. | *Turdus flavipes* | Colombia | Sierra Nevada de Santa Marta, San Lorenzo ridge | NA | 601 | NAN | (Gonzalez-Quevedo, Rivera-Gutierrez & Pabón, 2016) |
| KX130089 | 10,829 | -73,692 | *Leucocytozoon* sp. | *Myioborus flavivertex* | Colombia | Sierra Nevada de Santa Marta, San Lorenzo ridge | NA | 601 | NAN | (Gonzalez-Quevedo, Rivera-Gutierrez & Pabón, 2016) |
| KM610045 | 4,783 | -75,414 | *Leucocytozoon* sp. | *Grallaria quitensis* | Colombia | Otun Lagoon | GRAQUI01 | 602* | NAN | (Lotta *et al*., 2015) |
| KM272251 | 0,9 | -77,65 | *Leucocytozoon* sp. | *Grallaria quitensis* | Colombia | Chingaza NNP, Pupiales | GRQUI01 | 602* | NAN | (Lotta *et al*., 2016) |
| KM272251 | 4,783 | -75,414 | *Leucocytozoon* sp. | *Grallaria quitensis* | Colombia | Otun Lagoon | GRAQUI02 | 602* | NAN | (Lotta *et al*., 2015) |
| KM272251 | 4,717 | -75,45 | *Leucocytozoon* sp. | *Grallaria quitensis* | Colombia | Los Nevados National Natural Park, El Bosque station | GRAQUI02 | 602* | NAN | (Lotta *et al*., 2015) |
| KM610046 | 4,683 | -73,833 | *Leucocytozoon pterotenuis* | *Grallaria ruficapilla* | Colombia | Chingaza NNP, Palacio Forest | GRARUF02 | 603* | NAN | (Lotta *et al*., 2015) |
| KM272250 | 4,683 | -73,833 | *Leucocytozoon pterotenuis* | *Grallaria ruficapilla* | Colombia | Chingaza NNP, Palacio Forest | GRRUF01 | 603* | NAN | (Lotta *et al*., 2016) |
| KM272250 | 4,683 | -73,833 | *Leucocytozoon pterotenuis* | *Grallaria ruficapilla* | Colombia | Chingaza NNP, Palacio Forest | GRARUF01 | 603* | NAN | (Lotta *et al*., 2015) |
| KF717067 | 4,683 | -73,833 | *Leucocytozoon* sp. | *Atlapetes schistaceus* | Colombia | Chingaza NNP, Palacio Forest | L_ATSCHI_01 | 604 | NAN | (Bensch *et al*., 2009) |
| KF699311 | 4,617 | -73,717 | *Leucocytozoon* sp. | *Turdus fuscater* | Colombia | Chingaza NNP, Monter Redondo Station | TFUS07 | 605 | NAN | (Bensch *et al*., 2009; Lotta *et al*., 2016) |
| KF717057 | 4,7 | -75,483 | *Leucocytozoon* sp. | *Pachyramphus versicolor* | Colombia | Ucumarí Regional Natural Park | L_PAVER_01 | 606 | NAN | (Bensch *et al*., 2009; Lotta *et al*., 2016) |
| KF767439 | -14,407 | -73,087 | *Leucocytozoon* sp. | *Troglodytes aedon* | Peru | Apurímac | TROAED03 | 606 | CAN | (Galen and Witt, 2014) |
| KJ661319 | -3,003 | -78,51 | *Leucocytozoon* sp. | *Diglossa cyanea* | Ecuador | Cerro Bosco | NA | 607 | NAN | (Harrigan *et al*., 2014) |
| KF717061 | 4,57 | -74,297 | *Leucocytozoon* sp. | *Dubusia taeniata* | Colombia | NA | L_DUTAE_01 | 607 | NAN | (Bensch *et al*., 2009) |
| KF717061 | 4,683 | -73,833 | *Leucocytozoon* sp. | *Dubusia taeniata* | Colombia | Chingaza NNP, Palacio Forest | DUTAE01 | 607 | NAN | (Lotta *et al*., 2016) |
| KJ527076 | -9,190 | -75,015 | *Leucocytozoon* sp. | *Anas cyanoptera* | Peru | NA | NA | 608 | CAN | (Smith and Ramey 2015) |
| JX984673 | -33,641 | -78,846 | *Leucocytozoon* sp. | *Turdus falcklandii* | Chile | Robinson Crusoe Island | TUFAL06 | 609 | STP | (Martínez *et al*., 2015) |
| KF767429 | -11,767 | -76,528 | *Leucocytozoon* sp. | *Troglodytes aedon* | Peru | Lima | TROAED04 | 610 | CAN | (Galen and Witt, 2014) |
| KF767428 | -14,173 | -73,323 | *Leucocytozoon* sp. | *Troglodytes aedon* | Peru | Apurímac | TROAED11 | 611 | CAN | (Galen and Witt, 2014) |
| KF717064 | 4,7 | -75,483 | *Leucocytozoon* sp. | *Atlapetes albinucha* | Colombia | Ucumarí Regional Natural Park | ATAL02 | 612 | NAN | (Lotta *et al*., 2016) |
| KF717049 | 4,617 | -73,717 | *Leucocytozoon* sp. | *Phyllomyias nigrocapillus* | Colombia | Chingaza NNP, Monter Redondo Station | PHINIGR01 | 612 | NAN | (Bensch *et al*., 2009; Lotta *et al*., 2016) |
| KF717064 | 4,57 | -74,297 | *Leucocytozoon* sp. | *Atlapetes albinucha* | Colombia | NA | L_ATAL_02 | 612 | NAN | (Bensch *et al*., 2009) |
| KF717063 | 4,7 | -75,483 | *Leucocytozoon* sp. | *Atlapetes albinucha* | Colombia | Ucumarí Regional Natural Park | ATAL01 | 613 | NAN | (Lotta *et al*., 2016) |
| KF717063 | 4,57 | -74,297 | *Leucocytozoon* sp. | *Atlapetes albinucha* | Colombia | NA | L_ATAL_01 | 613 | NAN | (Bensch *et al*., 2009) |
| KF962961 | 4,717 | -75,45 | *Leucocytozoon* sp. | *Anisognathus lacrymosus* | Colombia | Los Nevados National Natural Park, El Bosque station | ANILA01 | 614 | NAN | (Lotta *et al*., 2016) |
| KJ661322 | 0,655 | -78,032 | *Leucocytozoon* sp. | *Diglossa cyanea* | Ecuador | Guandera | NA | 615 | NAN | (Harrigan *et al*., 2014) |
| EF153663 | -36,833 | -72,55 | *Leucocytozoon* sp. | *Turdus falcklandii* | Chile | Pantanillo | TUFAL01 | 616 | SAN | (Martínez *et al*., 2015) |
| JQ815432 | 4,617 | -73,717 | *Leucocytozoon fringillinarum* | *Turdus fuscater* | Colombia | Chingaza NNP, Monter Redondo Station | TFUS01 | 617 | NAN | (Lotta *et al*., 2013) |
| JQ815433 | 4,617 | -73,717 | *Leucocytozoon fringillinarum* | *Turdus fuscater* | Colombia | Chingaza NNP, Monter Redondo Station | TFUS02 | 618 | NAN | (Lotta *et al*., 2013) |
| NA | -9,190 | -75,015 | *Plasmodium* sp. | *Troglodytes aedon* | Peru | NA | BAEBIC02 P1 | NA | AMS | (Galen and Witt, 2014) |
