# Supplementary material for: Genetic variation of avian malaria in the tropical Andes: a relationship with the spatial distribution of hosts
Source: Malar J. 2019 Apr 11;18:129. doi: 10.1186/s12936-019-2699-9 (PMC6458820; doi:10.1186/s12936-019-2699-9)
Supplement: Supplementary file 4 — Additional file 4. Summary of clustering accessions of avian haemosporidia cytochrome b into haplotype groups. Haplotypes were determined using USEARCH v8.1 [22], implementing a cut-off level criterion > 99.3% similarity between sequences, for 1686 unique accessions in the database and further detailed in Additional file 3. The number of accessions refers to the unique sequences in the database used for the haplotype determination. [file 12936_2019_2699_MOESM4_ESM.docx]

**Additional file 4. Summary of clustering accessions of avian haemosporidia cytochrome *b* into haplotype groups.** Haplotypes were determined using USEARCH v8.1 (22), implementing a cut-off level criterion >99.3% similarity between sequences, for 1686 unique accessions in the database and further detailed in the Additional file 3. The number of accessions refers to the unique sequences in the database used for the haplotype determination.

| Sequences | *Plasmodium*/ *Haemoproteus* | *Leucocytozoon* |
| --- | --- | --- |
| Number of GenBank Accessions | 1599 | 87 |
| Multiple Database Sequences | 123 | 4 |
| Unique Database Sequences | 977 | 77 |
| Haplotypic groups identified | 571 | 48 |
| Single-sequence haplotypes | 385 | 23 |
| Number of sequences per haplotypic group (Maximum/average/minimum) | 62/2.8/1 | 10/1.9/1 |
