# Supplementary material for: Genetic variation of avian malaria in the tropical Andes: a relationship with the spatial distribution of hosts
Source: Malar J. 2019 Apr 11;18:129. doi: 10.1186/s12936-019-2699-9 (PMC6458820; doi:10.1186/s12936-019-2699-9)
Supplement: Supplementary file 5 — Additional file 5. Rarefaction plot of avian haemosporidia haplotype richness on the number of GenBank accessions. Rarefaction of the number of haplotype groups of avian haemosporidia as a function of the number of cyt b sequences in the dataset using EstimateS v 9.1.0 [32]. The grey area represents the 95% level of confidence interval. A total of 619 haplotypes (black circle) of 1686 cyt b sequences from avian malaria parasites was obtained; the dotted line represents the extrapolated rarefaction value up to 6000 sequences. [file 12936_2019_2699_MOESM5_ESM.docx]

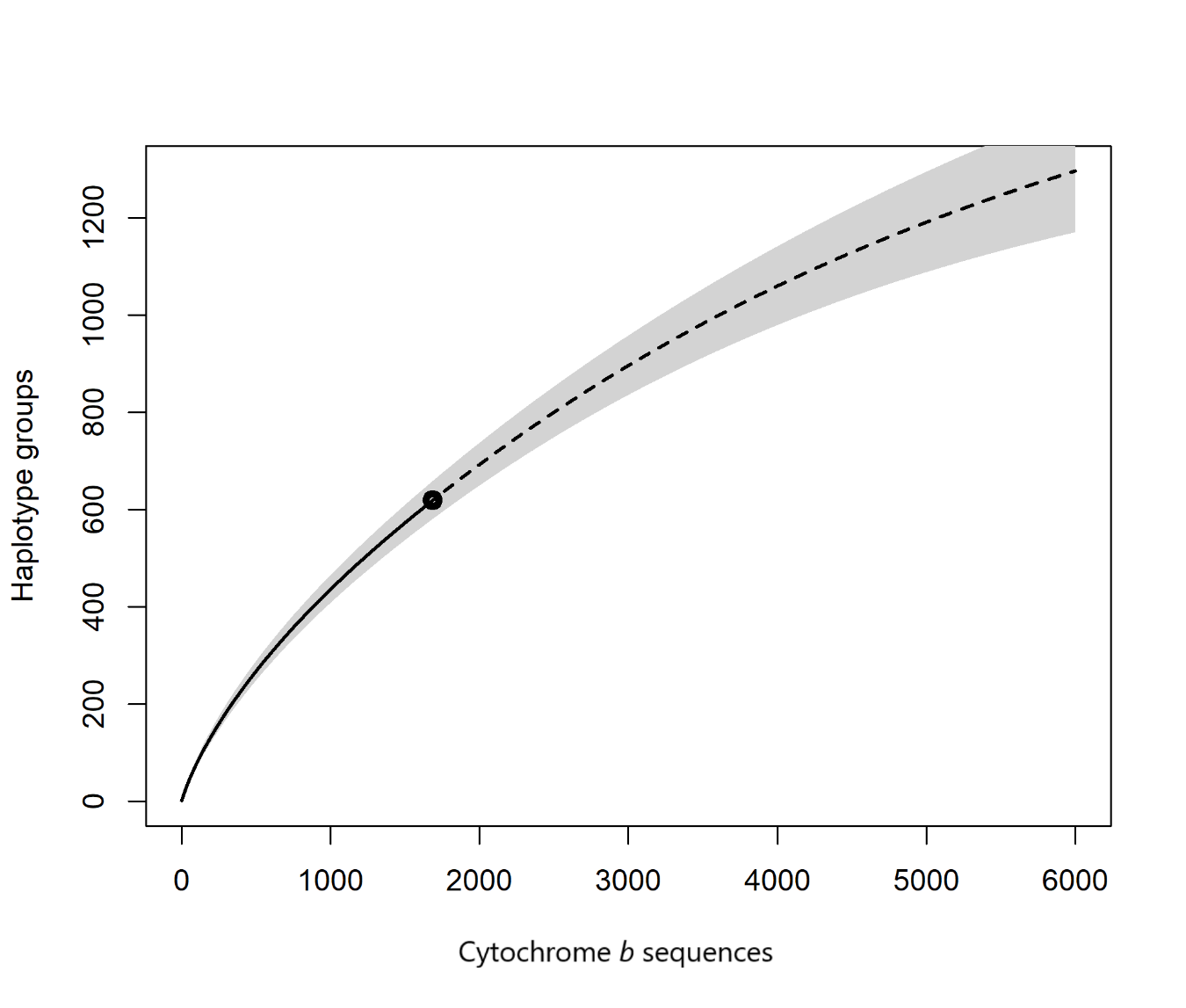


**Additional file 5. Rarefaction plot of avian haemosporidia haplotype richness on the number of GenBank accessions.** Rarefaction of the number of haplotype groups of avian haemosporidia as a function of the number of cyt *b* sequences in the dataset using EstimateS v 9.1.0 (32). The grey area represents the 95% level of confidence interval. A total of 619 haplotypes (black circle) of 1686 cyt *b* sequences from avian malaria parasites was obtained; the dotted line represents the extrapolated rarefaction value up to 6000 sequences.
